# Investigating EGF and PAG1 as necroptosis-related biomarkers for diabetic nephropathy: an *in silico* and *in vitro* validation study

**DOI:** 10.18632/aging.205233

**Published:** 2023-11-20

**Authors:** Yuejun Wang, Linlin Zhang, Zhiping Peng

**Affiliations:** 1 Department of Geriatrics, Zhejiang Aged Care Hospital, Hangzhou Normal University, Hangzhou 310000, Zhejiang, China; 2 Zhejiang Institute for Food and Drug Control, Hangzhou 310012, Zhejiang, China; 3 Department of Gerontology, Hangzhou TCM Hospital Affiliated to Zhejiang Chinese Medical University, Hangzhou 310007, Zhejiang, China

**Keywords:** regulated cell death, diabetic nephropathy, biomarkers

## Abstract

The current study aims to understand the mechanisms behind regulated cell death (RCD) in diabetic nephropathy and identify related biomarkers through bioinformatics and experimental validation. Datasets of bulk and single-cell RNA sequencing were obtained from public databases and analyzed using gene set variation analysis (GSVA) with gene sets related to RCD, including autophagy, necroptosis, pyroptosis, apoptosis, and ferroptosis. RCD-related gene biomarkers were identified using weighted gene correlation network analysis (WGCNA). The results were verified through experiments with an independent cohort and *in vitro* experiments. The GSVA revealed higher necroptosis scores in diabetic nephropathy. Three necroptosis-related biomarkers, EGF, PAG1, and ZFP36, were identified and showed strong diagnostic ability for diabetic kidney disease. *In vitro* experiments showed high levels of necroptotic markers in HK-2 cells treated with high glucose. Bioinformatics and experimental validation have thus identified EGF and PAG1 as necroptosis-related biomarkers for diabetic nephropathy.

## INTRODUCTION

Diabetic nephropathy (DN) is a prevalent complication of diabetes that leads to structural alterations in the glomeruli, including mesangial hyperplasia, hypertrophy, and nodular sclerosis. According to estimates, DN affects 30 to 40% of diabetic patients [1]. The literature establishes the significance of DN as a leading cause of end-stage renal disease [2, 3]. Renal intrinsic cells undergo regulated cell death (RCD) upon chronic exposure to high glucose levels, thereby instigating an auto-amplifying cycle of inflammation that exacerbates the progression of DN [4]. RCD is a gene-driven process that eliminates unwanted, redundant, and cancerous cells, and encompasses several distinct patterns, including autophagy, pyroptosis, necroptosis, apoptosis, and ferroptosis [5]. Endoplasmic reticulum stress, oxidative damage, and glycosylation end-product damage are underlying mechanisms of RCD abnormalities in DN, affecting different cells, including renal tubular epithelial cells and podocytes [6].

Apoptosis in renal intrinsic cells has been identified as a fundamental mechanism underlying DN in previous research [7]. Animal and cell experiments suggest that high glucose conditions significantly increase apoptosis in renal tubular epithelial cells, which are intrinsic renal cells. This effect may be linked to the oxidative stress induced by hyperglycemia in renal tubular cells [8], as reactive oxygen species are known to promote cell apoptosis. Autophagy has also emerged as a key research area in DN, with impaired autophagy detected in early diabetic models, especially in the renal cortical tubule fraction [9]. Such impairment may contribute to mitochondrial damage, which is associated with renal fibrosis and sclerosis [10]. Another mode of regulated death, cell pyroptosis, has been linked to DN through Gasdermin D-mediated renal tubular epithelial cell pyroptosis activated by the TLR4/NF-κB signaling pathway [11]. Ferroptosis, characterized by intracellular iron overload, has been associated with DN through increased ACSL4 expression in DN mouse tissues and *in vitro* studies confirming that ferroptosis inducers can lead to cell death in kidney tubules. Furthermore, iron and ACSL4 increase the sensitivity of cells to ferroptosis [12]. Hence, investigating the mechanisms underlying RCD is diagnostically and therapeutically important for DN.

Necroptosis is a form of regulated cell death mediated by receptor-interacting protein kinase 1 (RIPK1), RIPK3, and mixed lineage kinase domain-like pseudokinase (MLKL). It is morphologically characterized by plasma membrane rupture and release of intracellular contents, distinguishing it from apoptosis [13]. The necroptotic pathway is activated when caspase-8 activity is inhibited, preventing apoptosis. This results in the formation of the necrosome complex containing RIPK1, RIPK3, and MLKL. RIPK3 phosphorylates MLKL, causing its oligomerization and translocation to the plasma membrane. This disrupts membrane integrity and leads to necroptotic cell death [14]. Several initiating stimuli can trigger necroptosis, including tumor necrosis factor α (TNFα), Fas ligand, Toll-like receptor activation, and interferons. These lead to assembly of the necrosome via RIPK1 and RIPK3. Executioner MLKL then induces lytic cell death [15].

Necroptosis has been implicated as a key mechanism of cellular demise in several diseases, including ischemic injury, neurodegeneration, and viral infection. Emerging evidence suggests it may also play a role in the pathogenesis of diabetes and its complications. Clinical trials have reported elevated RIPK1/RIPK3/MLKL expression in DN renal tissue [16], while experimental studies have shown high expression of RIPK1/RIPK3 in DN rat‘s models and glomerular endothelial cells stimulated by high glucose. Adiponectin has been found to inhibit necroptosis mediated by RIPK1/RIPK3 and reduce the inflammatory response and renal damage induced by DN [17]. Hyperglycaemia induces metabolic stress and inflammation, activating necroptotic signaling in renal cells like podocytes, tubular epithelial cells and endothelial cells [18]. Animal models have shown heightened renal expression of key necroptotic mediators RIPK1, RIPK3 and MLKL in diabetic mice compared to controls. Morphological signs of necroptosis are also observed [19]. Hyperglycaemia causes mitochondrial dysfunction and oxidative stress in renal cells. This results in depletion of ATP and inhibition of apoptosis, shunting cells towards necroptosis. Excess glucose also upregulates expression of cyclophilin D, which sensitizes the mitochondrial permeability transition pore (mPTP) to open. This leads to loss of mitochondrial membrane potential, ROS production and eventual necroptotic cell death [20].

Previous research on DN has been limited by low-throughput experimental validation, resulting in a lack of studies that investigate the role of RCD mechanisms in DN using high-throughput technologies. Single-cell sequencing technology provides an opportunity for high-throughput sequencing of the transcriptome at the individual cell level, allowing for the identification of intercellular heterogeneity and the genetic expression status of a single cell [21]. However, the renal tissue contains various intrinsic and interstitial cells, and traditional RNA sequencing methods only explore the average level of variation in cells. Therefore, the use of scRNA technology can reveal the cellular profile of the kidney and improve our understanding of the physiological and pathological changes in DN.

The aim of this study was to identify biomarkers related to necroptosis in DN using a combination of bioinformatics methodology and RNA sequencing techniques. To achieve this, we utilized bulk RNA-seq and scRNA-seq data to examine the transcriptomic profiles of cells from renal tissues affected by DN. By comparing the expression profiles of these cells to those of healthy controls, we were able to identify genes and pathways associated with necroptosis in DN. To further validate our findings, we conducted *in vitro* experiments to investigate the effects of specific genes on cell death and other cellular processes. Overall, our approach allowed us to gain a better understanding of the molecular mechanisms underlying necroptosis in DN and provided insights into potential therapeutic targets for this disease.

## RESULTS

### scRNA-seq data analysis

**Figure 1:**
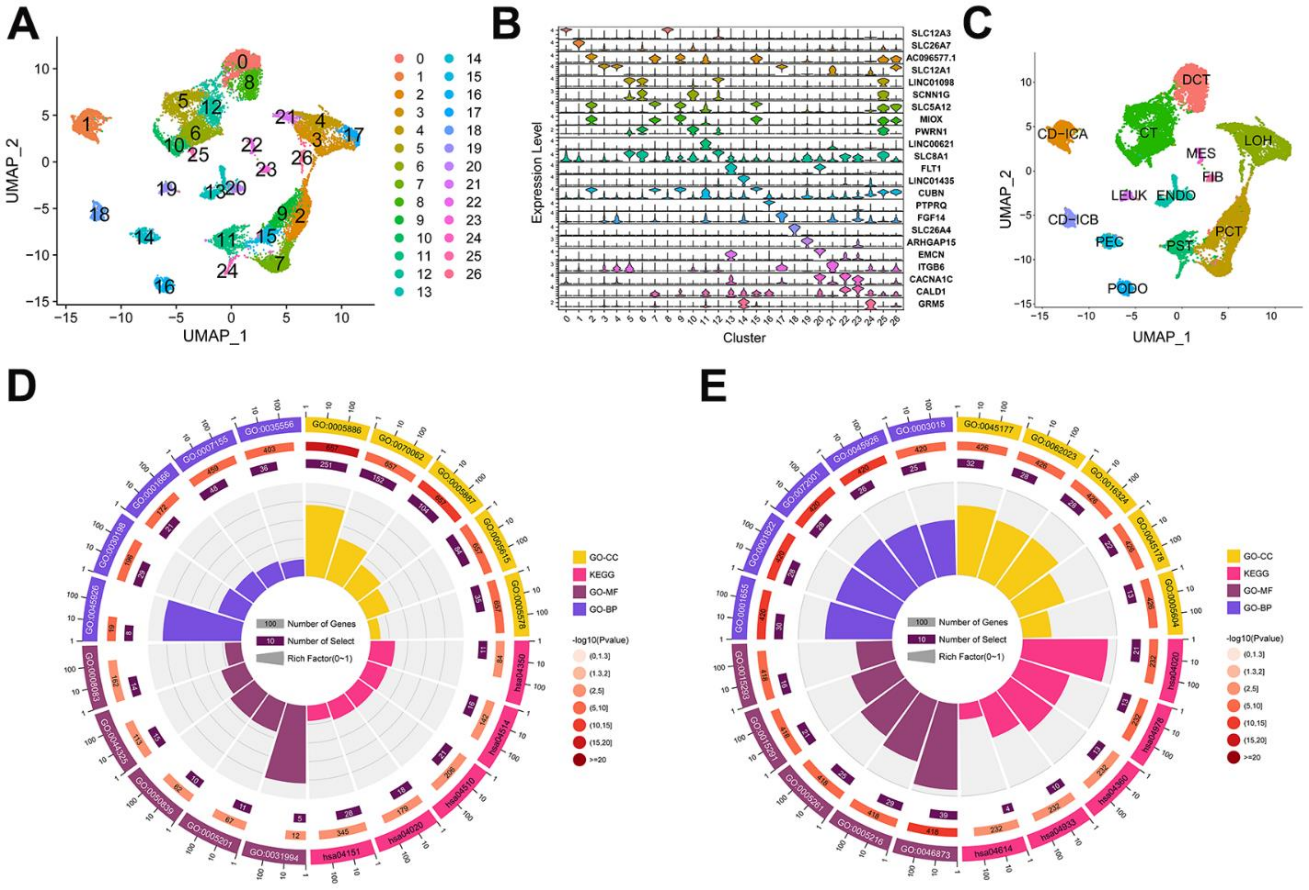
**Cell type and the identification of differentially expression genes (DEGs) in the scRNA-seq dataset.** (**A**) The distribution of 27 cell clusters (0-26) in the GSE131882 dataset as determined by the UMAP algorithm. (**B**) Violin plot showing the expression levels of highly variable gene markers in different cell clusters. (**C**) Thirteen cell types were identified in the GSE131882 scRNA-seq data. (**D**) Circle plot of the top 5 GO and KEGG enrichments in the up-regulated DEGs. (**E**) Circle plot of the top 5 GO and KEGG enrichments in the down-regulated DEGs. Abbreviations: LOH, loop of Henle cell; CD-ICA, collecting duct type A intercalated cell; CT, connecting tubule cell; PEC, parietal epithelial cell; CD-ICB, collecting duct type B intercalated cell; PST, proximal straight tubule cell; DCT, distal convoluted tubule cell; PODO, podocyte cell; ENDO, endothelium cell; PCT, proximal convoluted tubule cell; FIB, fibroblast cell; MES, mesangial cell; LEUK, leukocyte cell; KEGG, the Kyoto Encyclopaedia of Genes and Genomes; GO, gene ontology; UMAP: uniform manifold approximation and projection.

The Seurat package was utilized for integration and quality control processes, which identified a total of 23,980 cells. Subsequently, the UMAP algorithm revealed twenty-seven distinct clusters, as depicted in Figure 1A. The top 20 most highly variable markers were visualized in Figure 1B. Using gene markers identified from literature relating to kidneys (Supplementary Table 1), we identified 13 cell types from all clusters, as shown in Figure 1C. We then identified 698 down-regulated DEGs and 511 up-regulated DEGs (Supplementary Table 2).

### Functional enrichment of DEGs in scRNA-seq data

The results of the GO analysis indicated that the majority of up-regulated DEGs were enriched in the plasma membrane, playing a role in cell adhesion and extracellular matrix organization (Figure 1D and Table 1). In contrast, the down-regulated DEGs were mostly associated with urogenital system and kidney development in the apical regions of cells, as determined by GO enrichment analysis (Figure 1E and Table 1). Additionally, KEGG enrichment analysis revealed that the up-regulated genes were primarily enriched in pathways related to cell adhesion (Figure 1D and Table 1), while the down-regulated genes were mainly associated with the AGE-RAGE pathway and mineral absorption (Figure 1E and Table 1).

**Table 1:** Top5 GO and top5 KEGG enrichment in differentially expressed genes of sing-cell RNA seq dataset.

**ID**	**Classification**	**Classification**	**adj. *p*-value**	**Count**	**Regulation**
GO:0007155	GO-BP	cell adhesion	4.25E-10	48	Up
GO:0030198	GO-BP	extracellular matrix organization	1.17E-09	29	Up
GO:0045926	GO-BP	negative regulation of growth	3.44E-06	8	Up
GO:0035556	GO-BP	intracellular signal transduction	3.71E-06	36	Up
GO:0001666	GO-BP	response to hypoxia	7.29E-06	21	Up
GO:0005886	GO-CC	plasma membrane	3.27E-20	251	Up
GO:0005887	GO-CC	integral component of plasma membrane	3.33E-12	104	Up
GO:0005578	GO-CC	proteinaceous extracellular matrix	1.75E-10	35	Up
GO:0070062	GO-CC	extracellular exosome	1.28E-07	152	Up
GO:0005615	GO-CC	extracellular space	9.13E-07	84	Up
GO:0044325	GO-MF	ion channel binding	6.86E-05	15	Up
GO:0005201	GO-MF	extracellular matrix structural constituent	1.58E-04	11	Up
GO:0050839	GO-MF	cell adhesion molecule binding	4.14E-04	10	Up
GO:0031994	GO-MF	insulin-like growth factor I binding	7.09E-04	5	Up
GO:0008083	GO-MF	growth factor activity	0.006930406	14	Up
hsa04510	KEGG	Focal adhesion	0.001431117	21	Up
hsa04514	KEGG	Cell adhesion molecules (CAMs)	0.002452309	16	Up
hsa04020	KEGG	Calcium signaling pathway	0.004017716	18	Up
hsa04151	KEGG	PI3K-Akt signaling pathway	0.005444036	28	Up
hsa04350	KEGG	TGF-beta signaling pathway	0.005445321	11	Up
GO:0001655	GO-BP	urogenital system development	3.47E-11	30	Down
GO:0001822	GO-BP	kidney development	3.51E-11	28	Down
GO:0045926	GO-BP	negative regulation of growth	4.78E-11	26	Down
GO:0072001	GO-BP	renal system development	6.82E-11	28	Down
GO:0003018	GO-BP	vascular process in circulatory system	2.63E-10	25	Down
GO:0045177	GO-CC	apical part of cell	6.69E-10	32	Down
GO:0062023	GO-CC	collagen-containing extracellular matrix	1.14E-09	32	Down
GO:0016324	GO-CC	apical plasma membrane	4.03E-09	28	Down
GO:0045178	GO-CC	basal part of cell	5.94E-08	22	Down
GO:0005604	GO-CC	basement membrane	1.29E-07	13	Down
GO:0046873	GO-MF	metal ion transmembrane transporter activity	1.41E-13	39	Down
GO:0015291	GO-MF	secondary active transmembrane transporter activity	1.46E-07	21	Down
GO:0015293	GO-MF	symporter activity	1.74E-07	16	Down
GO:0005216	GO-MF	ion channel activity	2.03E-07	29	Down
GO:0005261	GO-MF	cation channel activity	2.04E-07	25	Down
hsa04978	KEGG	Mineral absorption	2.76E-06	13	Down
hsa04020	KEGG	Calcium signaling pathway	6.64E-04	21	Down
hsa04933	KEGG	AGE-RAGE signaling pathway in diabetic complications	4.20E-02	10	Down
hsa04360	KEGG	Axon guidance	1.51E-02	13	Down
hsa04614	KEGG	Renin-angiotensin system	1.66E-02	4	Down

### Identification of five different patterns of RCD gene markers and the assessment of RCD enrichment

**Figure 2:**
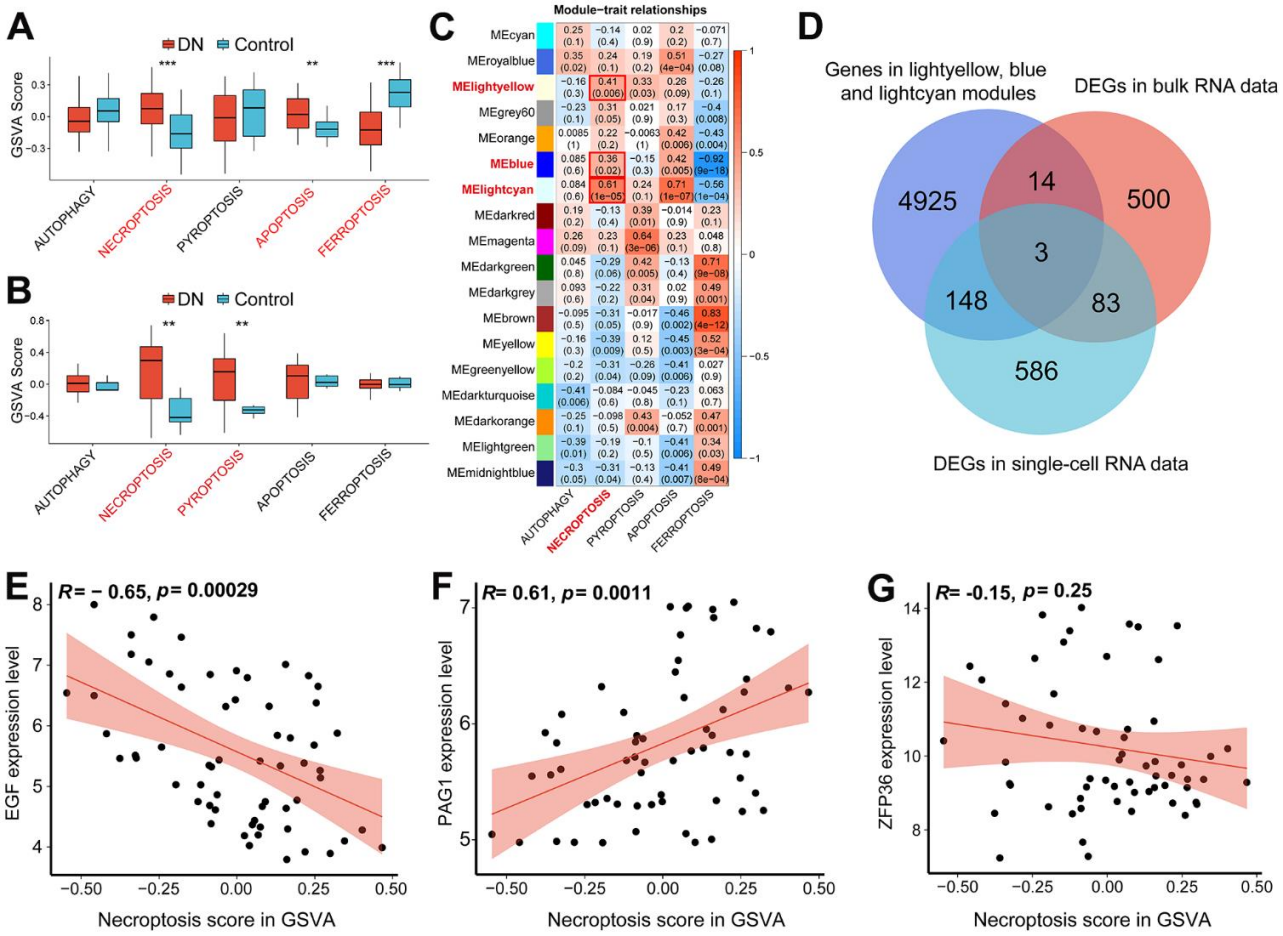
**The identification of necroptosis-related biomarkers.** (**A**) GSVA score of five different types of programmed cell death (PCD) in the bulk RNA-seq data (GSE96804). (**B**) GSVA score of five different types of programmed cell death based on the scRNA-seq data (GSE131882). (**C**) Heatmap showing the correlation between PCD traits and gene modules. (**D**) Venn diagram demonstrating the necroptosis-related biomarkers by intersecting necroptotic gene modules arising from WGCNA analysis, DEGs in the bulk RNA-seq data and DEGs in the scRNA-seq data. Scatter plots showing Pearson’s correlation analysis between necroptotic GSVA scores and EGF (**E**), PAG1 (**F**) and ZFP36 (**G**) respectively. (**P<0.01; ***P<0.001). Abbreviations: DN: diabetic nephropathy; GSVA: gene set variation analysis; WGCNA: weighted gene correlation network analysis; DEGs: differentially expression genes.

We compiled a list of gene biomarkers associated with five different patterns of regulated cell death by utilizing the GO, KEGG, and FerrDb v2 databases, as presented in Table 2. Next, we utilized these RCD-related gene signatures to conduct gene set variation analysis on RNA-seq datasets, including single-cell and bulk data. In the bulk dataset, higher GSVA scores for necroptosis and apoptosis were observed in DN samples, while the score for ferroptosis was relatively low (Figure 2A). Interestingly, the scRNA-seq dataset also demonstrated higher necroptosis scores in DN samples. Moreover, we identified a higher pyroptosis score in DN samples based on the scRNA-seq data (Figure 2B).

**Table 2:** A collection of biomarkers for five different types of regulated cell death (RCD).

**Types of RCD**	**Sources**	**Gene markers**
Autophagy	KEGG_REGULATION_OF_AUTOPHAGY v7.5.1	ATG12 ATG3 ATG4A ATG4B ATG4C ATG4D ATG5 ATG7 BECN1 BECN2 GABARAP GABARAPL1 GABARAPL2 IFNA1 IFNA10 IFNA13 IFNA14 IFNA16 IFNA17 IFNA2 IFNA21 IFNA4 IFNA5 IFNA6 IFNA7 IFNA8 IFNG INS PIK3C3 PIK3R4 PRKAA1 PRKAA2 ULK1 ULK2 ULK3
Necroptosis	GOBP_NECROPTOTIC_SIGNALING_PATHWAY.v7.5.1	RIPK1 RIPK3 MLKL ZBP1 CASP8 TNF CYLD ITPK1 IPMK MAP3K7 CASP6 TRPM7 FADD PELI1 PGLYRP1 SPATA2 SIRT3 HMGB1 TP53 TNFRSF1A
Pyroptosis	GOBP_PYROPTOSIS.v7.5.1	AIM2 APIP CASP1 CASP4 CASP6 CASP8 DHX9 ELANE GSDMA GSDMB GSDMC GSDMD GSDME GZMA GZMB NAIP NLRC4 NLRP1 NLRP6 NLRP9 TREM2 ZBP1
Apoptosis	KEGG_APOPTOSIS v7.5.1	AIFM1 AKT1 AKT2 AKT3 APAF1 ATM BAD BAX BCL2 BCL2L1 BID BIRC2 BIRC3 CAPN1 CAPN2 CASP10 CASP3 CASP6 CASP7 CASP8 CASP9 CFLAR CHP1 CHP2 CHUK CSF2RB CYCS DFFA DFFB ENDOD1 ENDOG EXOG FADD FAS FASLG IKBKB IKBKG IL1A IL1B IL1R1 IL1RAP IL3 IL3RA IRAK1 IRAK2 IRAK3 IRAK4 MAP3K14 MYD88 NFKB1 NFKBIA NGF NTRK1 PIK3CA PIK3CB PIK3CD PIK3CG PIK3R1 PIK3R2 PIK3R3 PIK3R5 PPP3CA PPP3CB PPP3CC PPP3R1 PPP3R2 PRKACA PRKACB PRKACG PRKAR1A PRKAR1B PRKAR2A PRKAR2B PRKX RELA RIPK1 TNF TNFRSF10A TNFRSF10B TNFRSF10C TNFRSF10D TNFRSF1A TNFSF10 TP53 TRADD TRAF2 XIAP
Ferroptosis	FerrDb V2	DECR1 ZEB1 PIR SIRT6 CD82 TF ADAM23 AGPS GPX4 SNCA HRAS ALOX12 MDM2 KLHDC3 EGFR NDRG1 CREB1 ACSL1 ABCC5 BRDT PEDS1 CHP1 BRD3 NEDD4 EZH2 HDDC3 MTF1 YY1AP1 MTCH1 RRM2 BEX1 AKT1S1 AMN CIRBP DDR2 PGD HCAR1 HSPB1 GDF15 FADS1 MICU1 KDM5A MGST1 NCOA3 CCDC6 CTSB ZFP36 NEDD4L PARK7 PTGS2 ALOXE3 EMC2 MAPK1 BRD7 ATF2 MAPK8 MYCN EGLN2 OSBPL9 LAMP2 LCE2C ABCC1 IFNA21 ACSL4 SOX2 SLC39A7 LYRM1 CGAS AGPAT3 PARP14 PARP11 FURIN SMAD7 PARP6 NOX4 ACO1 TYRO3 DUOX1 PARP1 PML MLLT1 ACADSB CDCA3 CYGB GLRX5 CISD1 KIF20A TTPA TMSB4X ANO6 PRKCA IFNA8 NFE2L2 MT1G CHMP6 TOR2A GCH1 VCP SIAH2 PTPN6 INTS2 CDKN1A PEBP1 HMGB1 PHKG2 SLC40A1 PRDX1 AEBP2 IREB2 FTL TRIM26 ATM PARP2 ALOX15B AIFM2 CYB5R1 NR5A2 FAR1 HELLS ALOX12B SIRT3 TLR4 NOX1 MMD LCN2 CDKN2A JUN AR SESN2 ATP5MC3 ARF6 PLIN2 MIOX FXN CREB3 USP11 POR SREBF2 PDK4 HMOX1 IFNA7 METTL14 GSTM1 TSC1 GPAT4 SOCS1 FBXW7 TP53 OTUB1 RPL8 IL1B PEX12 PARP9 PANX1 SQSTM1 ATF3 DPP4 MUC1 IDH2 SLC1A5 MPC1 ETV4 GRIA3 ARNTL KDM3B CHAC1 TMBIM4 SUV39H1 CDH1 FNDC5 PARP10 DLD P4HB PDSS2 IFNA10 CP NCOA4 TP63 LONP1 FTMT PAQR3 YTHDC2 FH G6PD BCAT2 IFNA14 CISD3 SAT1 RBMS1 MEF2C MYB RPTOR CHMP5 CS PRKAA1 FADS2 AHCY RNF113A SMPD1 SCD STAT3 DNAJB6 KEAP1 PEX2 NQO1 IFNG PANX2 ACVR1B WWTR1 SLC38A1 PARP3 FABP4 SLC25A28 NF2 MIB1 MDM4 GOT1 PARP16 POM121L12 PLA2G6 ATG7 SLC7A11 PARP8 BRPF1 CYBB SRC NFS1 AKR1C1 CARS1 BRD2 ELAVL1 IDH1 IFNA6 CLTRN SREBF1 SLC16A1 PRDX6 KDM5C IFNA2 KRAS STING1 TFRC ATF4 SLC11A2 RARRES2 MAPK3 LIG3 SMG9 TRIB2 VDAC2 CD44 ECH1 ABHD12 BRD4 KLF2 ISCU CA9 CBS PARP4 KDM6B GSK3B TNFAIP3 GCLC KDM4A NR4A1 NOX3 TAZ GSTZ1 TRIM46 CDC25A ALOX15 NUPR1 FZD7 TGFBR1 DCAF7 IFNA5 PIEZO1 IL6 IFNA16 PRKAA2 TIMM9 CDO1 PIK3CA PEX3 IFNA13 CAV1 HIF1A IFNA4 SRSF9 CAMKK2 AKR1C3 ELOVL5 ACSL3 CPEB1 ACSF2 FTH1 USP35 TGFB1 PARP15 TFAM PARP12 NOX5 MTDH GALNT14 RB1 TFAP2A STK11 SIRT1 PTEN PTPN18 PROM2 ALOX5 GJA1 COPZ1 DUOX2 CYP4F8 AKR1C2 NRAS MTOR ATG5 CISD2 PEX6 CREB5 TMSB4Y PPARA IFNA17 LIFR HSPA5 MLST8 HSF1 BAP1 PEX10 IFNA1 QSOX1 LPCAT3 BECN1 DHODH PHF21A

### Identification of necroptosis-related gene modules

We employed the WGCNA algorithm on the bulk RNA-seq dataset to investigate the gene modules associated with necroptosis. As a result, we identified three significant gene modules, indicated as the yellow, blue, and cyan modules in Figure 2C. By combining these modules, we identified 5090 genes that are associated with necroptosis (Supplementary Table 3).

### Identification of necroptosis-related biomarkers

We aimed to identify necroptosis-related gene markers in our study. Firstly, we generated Venn diagrams for the scRNA-seq DEGs, the DEGs obtained from the bulk data (Supplementary Table 4), and the necroptosis-related gene modules (Figure 2D). By overlapping these gene lists, we identified three necroptosis-related gene markers: EGF, PAG1, and ZFP36. Next, we assessed the correlation between the expression levels of these biomarkers and necroptosis scores using Pearson’s correlation test. We found that EGF was negatively correlated with the necroptosis score (Figure 2E), whereas PAG1 was positively associated with the necroptosis score (Figure 2F). However, no statistically significant association was found between the expression levels of ZFP36 and the necroptosis score (Figure 2G).

### Biomarker expression profiles

**Figure 3:**
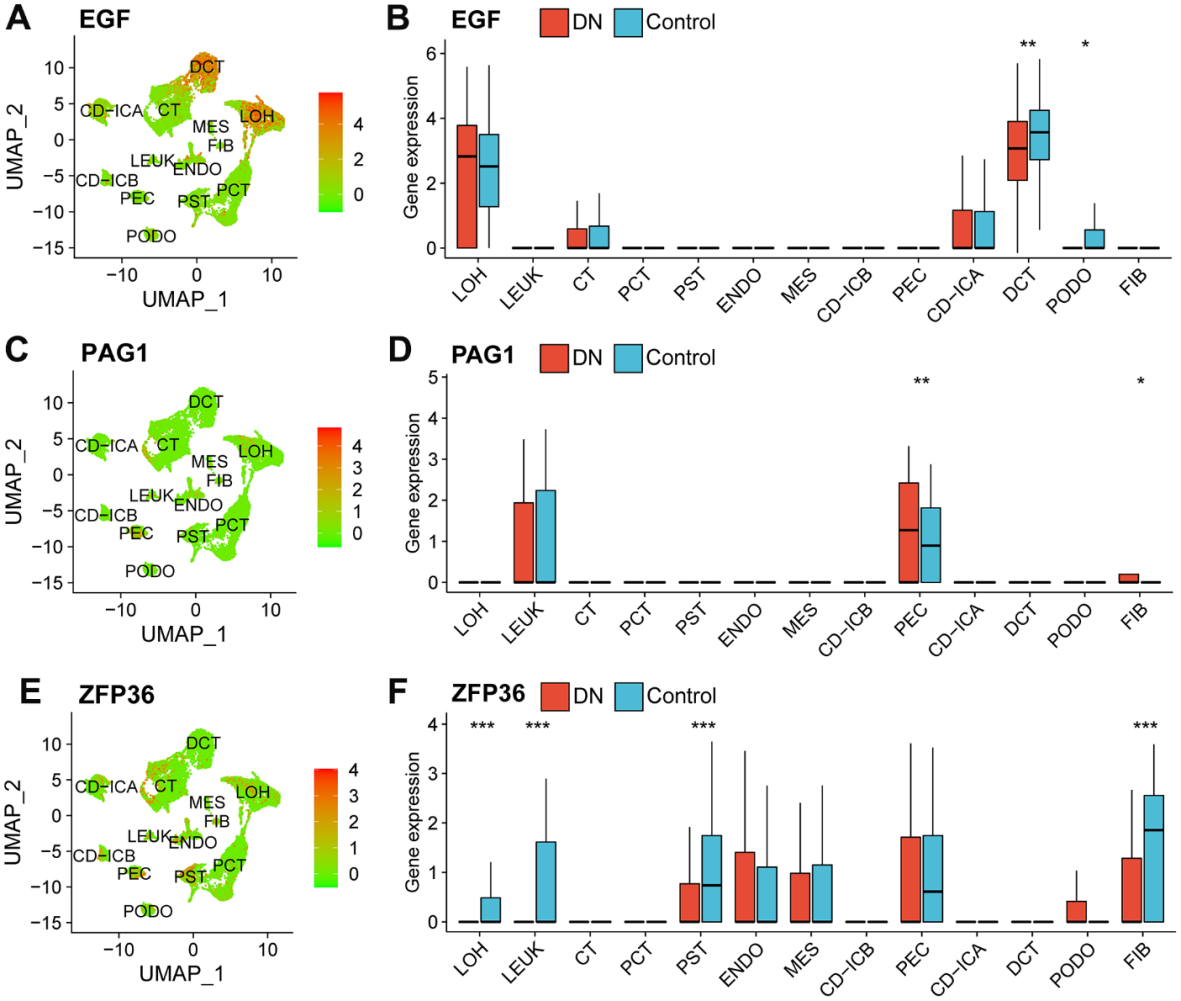
**Expression profiles of necroptosis-related biomarkers in the scRNA-seq data.** A dimension reduction plot (**A**) and boxplot (**B**) showing the profiles of EGF expression in different cell types. The expression levels of PAG1 in scRNA-seq data as shown as a dimension reduction plot (**C**) and boxplot (**D**). ZFP36 expression profile in different types of cells in the GSE131882 as shown in a dimension reduction plot (**E**) and boxplot (**F**). (Wilcoxon’s test; *p<0.05; **p<0.01; ***p<0.001). Abbreviations: LOH, loop of Henle cell; CD-ICA, collecting duct type A intercalated cell; CT, connecting tubule cell; PEC, parietal epithelial cell; CD-ICB, collecting duct type B intercalated cell; PST, proximal straight tubule cell; DCT, distal convoluted tubule cell; PODO, podocyte cell; ENDO, endothelium cell; PCT, proximal convoluted tubule cell; FIB, fibroblast cell; MES, mesangial cell; LEUK, leukocyte cell; DN: diabetic nephropathy; UMAP: uniform manifold approximation and projection.

In the scRNA-seq dataset, EGF was mainly expressed at higher levels in distal convoluted tubule (DCT) cells of healthy subjects (Figure 3A, 3B). PAG1 was predominantly expressed in parietal epithelial cells (PEC) of patients with DN (Figure 3C, 3D). The expression levels of ZFP36 were significantly higher in cells of the proximal straight tubule (PST), fibroblasts (FIB), loop of Henle (LOH), and leukocytes (LEUK) of healthy subjects (Figure 3E, 3F).

**Figure 4:**
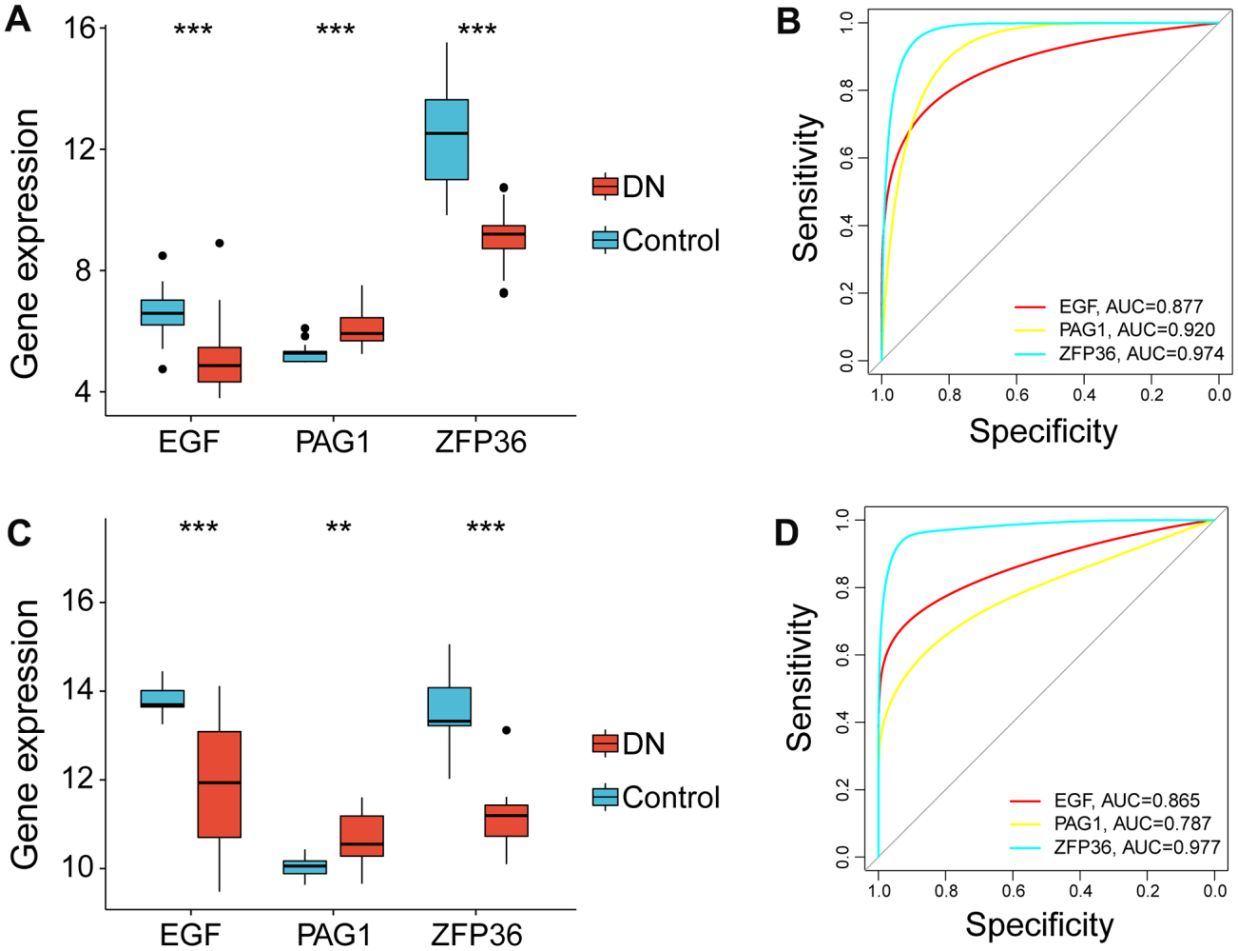
**Expression profile of necroptosis-related gene markers in the bulk RNA-seq data (GSE96804) and validation in the test cohort (GSE142025).** (**A**) Violin plot showing the expression levels of genes related to necroptosis in the bulk RNA-seq data. (**B**) Receiver operating characteristic curves of EGF, PAG1 and ZFP36 in the bulk RNA-seq data. (**C**) The expression levels of necroptosis-related biomarkers as validated by the external independent dataset and shown as a violin plot. (**D**) The performance of EGF, PAG1 and ZFP36 in the external independent dataset as determined by receiver operating characteristic curves. (**p<0.01; ***p<0.001, compared DN and control group).

Moreover, based on the analysis of the bulk RNA-seq datasets, EGF and ZFP36 were expressed at significantly higher levels in the control group (p-value<0.001), whereas PAG1 was expressed at higher levels in subjects with DN (p-value<0.001) (Figure 4A). The ROC analysis results revealed that EGF, PAG1, and ZFP36 had high accuracy in discriminating between normal patients and those with DN, with AUC values of 0.877, 0.920, and 0.974, respectively (Figure 4B).

### Validation in the external independent cohort

In the external test cohort (GSE142025), we observed that EGF and ZFP36 were significantly overexpressed in normal subjects (p-value<0.001), while PAG1 was expressed at high levels in DN patients (p-value<0.01) with expression levels similar to those in the training cohort (Figure 4C). ROC analysis demonstrated that EGF and ZFP36 had strong discriminatory abilities for distinguishing between normal controls and DN patients (AUC = 0.865 for EGF; AUC = 0.977 for ZFP36), while PAG1 had a moderate discriminatory ability (AUC = 0.787) (Figure 4D).

### Clinical features associations with the necroptosis-related gene markers

**Figure 5:**
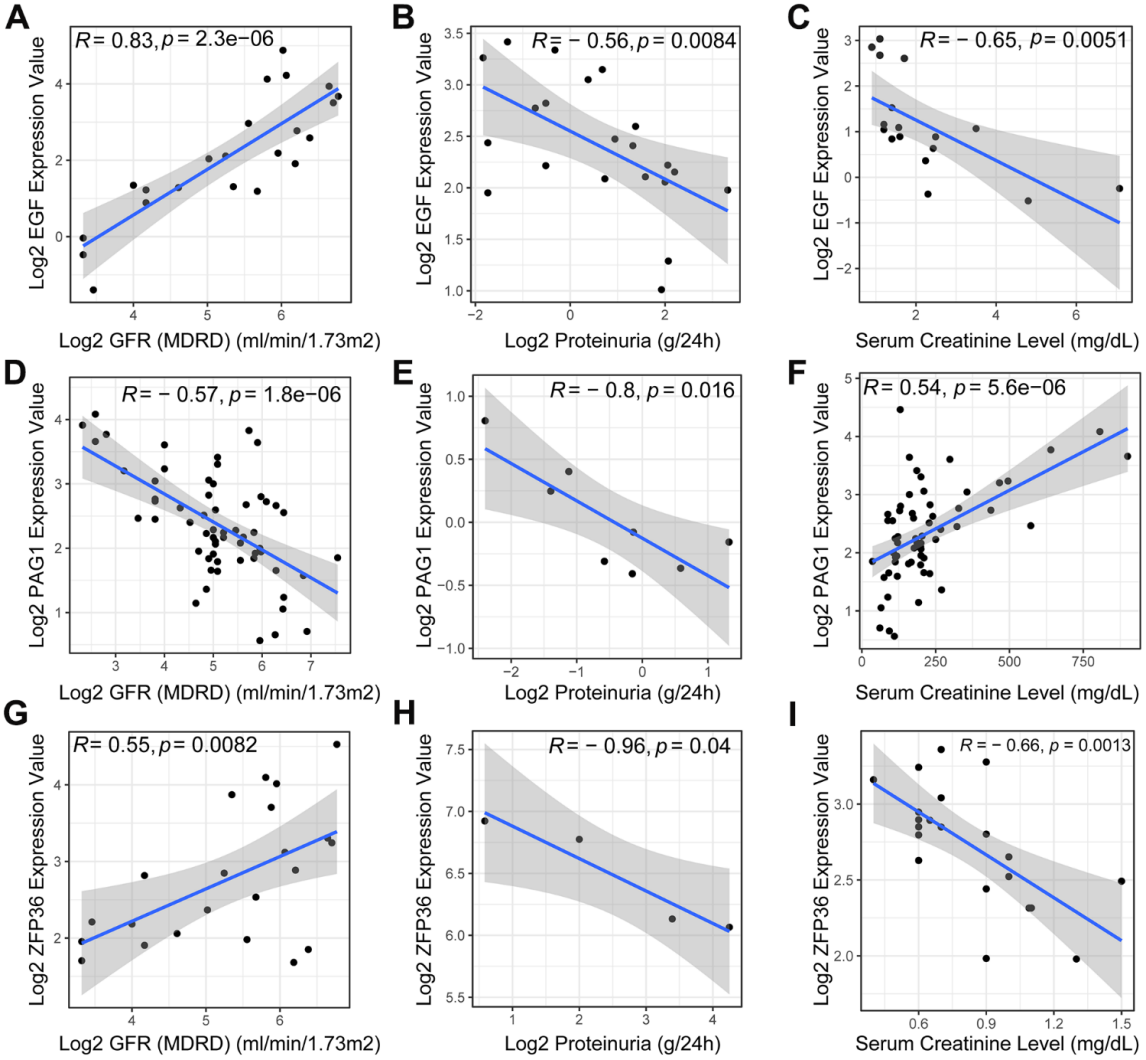
**Pearson correlation analysis of necroptosis-related biomarkers and clinical features.** The scatter plots revealed a positive correlation between the expression level of the EGF gene (**A**) in DN patients and the glomerular filtration rate (GFR), while exhibiting a negative correlation with proteinuria (**B**) and serum creatinine (**C**). Additionally, the expression level of PAG1 was negatively correlated with GFR (**D**) and proteinuria (**E**), but positively correlated with serum creatinine (**F**). ZFP36, on the other hand, exhibited a positive correlation with GFR (**G**) but a negative correlation with proteinuria (**H**) and serum creatinine (**I**) levels.

In this study, we investigated the potential of EGF, ZFP36, and PAG1 as biomarkers for chronic kidney disease (CKD). We found that EGF and ZFP36 were positively correlated with the estimated rate of glomerular filtration (P=2.3e-06 and R=0.83 for EGF; P=0.0082 and R=0.55 for ZFP36) (Figure 5A, 5G), indicating their potential to estimate the extent and progression of functional renal loss. However, PAG1 expression levels had no significant relationship with the glomerular filtration rate (P=1.8e-06 and R=-0.57 for PAG1) (Figure 5D).

Proteinuria, a known indicator of CKD, was negatively correlated with all three biomarkers (R=-0.56 for EGF; R=-0.8 for PAG1; R=-0.96 for ZFP36, all p-value <0.05) (Figure 5B, 5E, 5H). Furthermore, we found that the expression levels of EGF and ZFP36 were negatively correlated with serum creatinine levels in DN patients (P=0.0051 and R=-0.65 for EGF; P=0.0013 and R=-0.66 for ZFP36) (Figure 5C, 5I), whereas the levels of blood creatinine showed an opposite trend with regards to the expression of PAG1 in patients with DN (P=5.6e-06 and R=0.54 for PAG1) (Figure 5F).

### Activation of necroptosis in HK-2 cells with high-glucose treatment

**Figure 6:**
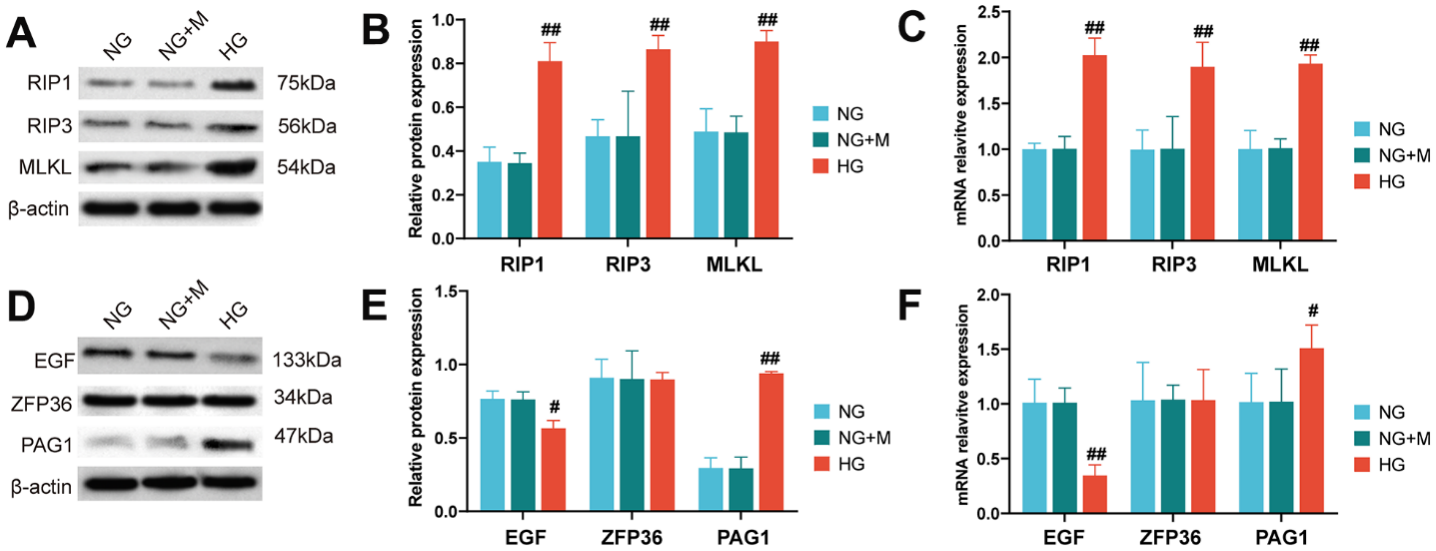
**Activation of necroptotic and necroptosis-related biomarkers in HK-2 cells stimulated by high glucose.** (**A**) Western blot assays were used to detect the expression levels of necroptotic proteins in HK-2 cells under different treatments. (**B**) Bar charts show the grey values obtained from the western blots shown in (**A**). (**C**) Reverse transcription quantitative PCR analysis was used to quantify the expression levels of necroptotic gene markers in HK-2 cells treated with NG, NG+M and HG, respectively. The protein and mRNA expression levels of EGF, PAG1 and ZFP36 were detected by western blot analysis (**D**, **E**) and reverse transcription quantitative PCR (**F**) in HK-cells when treated with NG, NG+M and HG. (#P < 0.05, ##P < 0.01, compared with NG group). Abbreviations: NG: normal glucose; NG+M: NG + Hyperosmotic medium; HG: high glucose; DN: diabetic nephropathy.

The necroptotic activation of HK-2 cells under high-glucose conditions was evaluated by western blotting and RT-qPCR analysis of RIP1, RIP3, and MLKL expression at the protein and mRNA levels. Our findings indicated that high glucose exposure led to a significant upregulation of RIP1, RIP3, and MLKL at both the protein (Figure 6A, 6B) and mRNA (Figure 6C) levels.

### Expression of necroptosis-related biomarkers in HG-stimulated HK-2 cells

To evaluate the expression of EGF, ZFP36, and PAG1, we employed western blotting and RT-qPCR methods (Figure 6D–6F). We found that the expression levels of EGF were significantly lower in the HG group at both the mRNA and protein level when compared to the normal glucose treatment. Conversely, the expression levels of PAG1 were significantly higher in the HG group than in the NG group. However, there was no statistically significant difference in the expression levels of ZFP26 between the NG and HG groups.

## DISCUSSION

In this study, we first investigated the scRNA-seq dataset using bioinformatics methods and identified twenty-seven clusters and thirteen cell types, highlighting the diversity and heterogeneity of cells in kidney tissue. We identified 1209 DEGs in the scRNA-seq data, which were mostly upregulated in DN patients and enriched in cell adhesion and organization of the extracellular matrix. Previous studies have demonstrated that the kidney tissues of DN patients are affected by various stimuli, including high glucose, which leads to increased cell adhesion and infiltration, ultimately resulting in the extensive accumulation of monocytes and macrophages in the kidney tissue and exacerbating endothelial cell damage [22, 23].

To evaluate the enrichment of regulated cell death (RCD) in RNA-seq datasets at both single-cell and bulk levels, we collected RCD-related gene sets for gene set variation analysis. Our findings revealed that t DN samples had a higher enrichment score for necroptosis in both the single-cell and bulk RNA-seq datasets. While previous studies have shown that different RCD patterns can be observed in diabetic kidney disease, they have not identified which pattern plays the most critical role in the disease [4, 24]. In our study, we found that necroptosis had a higher enrichment score than other RCD patterns, including autophagy, pyroptosis, apoptosis, and ferroptosis. Our results suggest that necroptosis may play a primary role in the progression of diabetic nephropathy (DN). Necroptosis is a distinct RCD pattern that is not dependent on the caspase pathway and is distinguishable from apoptosis and necrosis. Necroptotic cells display swollen morphology, cytoplasmic contents spill out, mitochondria dysfunction, and cell disintegration [25]. Studies have shown that RIP1 and RIP3 are critical for necroptotic signaling [26]. Recent *in vivo* and *in vitro* studies have indicated that necroptosis contributes to podocyte injury induced by high glucose levels, as well as high expression levels of RIP1, RIP3, and MLKL in podocytes, and necroptosis-related morphological features in DN groups were also described [27]. Moreover, kidney biopsies from DN patients and animal models have revealed that monocytes and macrophages are activated and recruited to the injury area to defend against and clear damaged cells, causing damage to the cells in the kidney tubular. M1 macrophages secrete a large number of inflammatory cytokines, which exacerbates the extent of necroptosis in kidney cells [28].

Therefore, we focused on the role of necroptosis in diabetic nephropathy (DN) and identified EGF, PAG1, and ZFP36 as potential biomarkers associated with necroptosis using the WGCNA algorithm. EGF is mainly generated from the loop of Henle and the renal distal convoluted tubule, and a previous study suggested that EGF may protect against HG-induced podocyte injury by regulating autophagy, promoting cell proliferation, and inhibiting apoptosis [29]. Tenascin-C exerts a direct stimulatory effect on the epidermal growth factor (EGF) receptor signaling pathway within muscle stem cells. This observation implies that necroptosis serves as a catalyst for the proliferation of muscle stem cells by activating EGF signaling, thereby facilitating muscle tissue regeneration [30]. Furthermore, it is noteworthy that within the white matter of the central nervous system, an excessive activation of ErbB receptors, which mediate the EGF signaling pathway, can precipitate deleterious outcomes [31]. Another significant insight pertains to the regulatory role of ZFP36 in the orchestration of the death complex known as the Ripoptosome. ZFP36’s action leads to the induction of RIP1-dependent necroptosis, underscoring its pivotal role in facilitating RIP1-dependent cell death under conditions marked by a depletion of inhibitor of apoptosis proteins (IAPs) [32]. Moreover, it is pertinent to mention that tumor necrosis factor alpha (TNFα) plays a promoting role in necroptosis. The expression of TNFα is augmented by mitogen-activated protein kinase-activated protein kinase 2, while ZFP36 serves as an inhibitor of this process [33].

This study observed a downregulation of EGF in the context of diabetic nephropathy (DN), which is consistent with prior research indicating a reduction in urinary EGF levels associated with a decline in glomerular filtration rate (GFR) among DN patients [34, 35]. Intriguingly, our investigation revealed a negative correlation between EGF expression levels and both proteinuria and serum creatinine levels. Nonetheless, additional research is warranted to thoroughly elucidate this relationship. Although there is limited existing literature connecting PAG1 to kidney disease, our findings demonstrated an upregulation of PAG1 in DN, aligning with its recognized pro-inflammatory role in conditions such as asthma [36] and nasopharyngeal carcinoma [37]. Nevertheless, certain contradictions in clinical correlations warrant further exploration. In the case of ZFP36, although its role in ferroptosis regulation in liver fibrosis is well-documented [38], its association with diabetic kidney disease remains poorly understood and requires further investigation. Our study notably identified the predominant expression of these biomarkers within renal tubular cells, specifically in the convoluted tubule and proximal straight tubule.

Furthermore, our *in vitro* experiments using the HK-2 cell line validated the expression patterns of the necroptotic biomarkers, such as RIP1, RIP3, and MLKL, under high glucose conditions. We also found that EGF and PAG1 expression levels were consistent with the results obtained *in silico*, suggesting their potential as therapeutic targets for DN. However, we did not find any significant changes in the expression levels of ZFP36 under different treatments, which needs further investigation.

However, there are still some limitations in this study. First, only three biomarkers were characterized, providing an incomplete representation of necroptotic signaling. Analysis of broader pathways is needed. Second, lack of validation in human DN samples limits clinical applicability. Animal models do not fully recapitulate human disease. Third, potential confounders like medications, comorbidities were not addressed. Larger, longitudinal clinical studies are required. Overall, our study provides important insights into the pathogenesis of DN and highlights potential biomarkers for diagnosis and treatment.

## CONCLUSIONS

In summary, our comprehensive investigation, which involved an integration of both single-cell and bulk RNA-seq analyses, in conjunction with experimental validation, has uncovered the critical role played by necroptosis in the progression of diabetic nephropathy (DN). Specifically, our results have highlighted the involvement of two essential biomarkers, EGF and PAG1, in the necroptotic pathway in DN. These findings represent an important step towards elucidating the underlying molecular mechanisms that drive the pathogenesis of DN, and offer valuable insights into the identification of potential therapeutic targets for this debilitating disease.

## MATERIALS AND METHODS

### Data collection and processing

To identify relevant datasets for our study, we used the keywords “Homo sapiens,” “diabetic kidney disease,” and “diabetic nephropathy” to search the Gene Expression Omnibus (GEO) database. We then carefully selected only those datasets that exclusively included patients with nephropathy caused by type 2 diabetes. Ultimately, we identified three datasets that met our criteria. The first was a single-cell RNA sequencing dataset (GSE131882), which included kidney tissue samples from three patients with DN and three healthy controls [39]. The second was a bulk RNA-seq dataset (GSE96804) that contained kidney tissue samples obtained from 40 patients with DN and 21 healthy controls [40]. Finally, we used the GSE142025 dataset as an external validation cohort, which consisted of 28 samples from patients with DN and 9 samples from healthy controls [41]. In Supplementary Table 5, we provide detailed clinical profiles of all the patients included in the three datasets.

In this study, we employed the Seurat package to analyze the single-cell sequencing data, as per the established guidelines [42]. We applied the following quality control standards to ensure the reliability of our analysis: (1) exclusion of cells with a gene count of less than 200 or more than 7500, (2) removal of cells with more than 25% of mitochondrial genes, and (3) elimination of double cells. The integrated samples were subjected to the Harmony algorithm and principal component analysis for linear downscaling and cell clustering. To visualize the data, we applied the non-linear downscaling method, “uniform manifold approximation and projection” (UMAP) algorithm. The function “FindAllMarkers” was utilized to identify specific genes in each cluster. Further, cell-specific markers mentioned in previous literature were used to annotate the clusters.

To analyze the bulk-RNA seq data, we first aligned the reads to the hg38 reference genome, and then transformed the raw read counts data to transcripts per million (TPM) for further analysis. Differential gene expression analysis was performed using the “limma” package [43]. We considered genes with a |log2-fold change (FC)| ≥ 1 and Benjamini and Hochberg adjusted p-value < 0.05 as differentially expressed genes (DEGs). These DEGs were subsequently used for downstream analyses.

### DEGs in scRNA-Seq and functional enrichment

In accordance with the cluster annotation results, we used the ‘FindMarkers’ function to investigate the DEGs between DN and control individuals in various clusters. Genes with a p-value < 0.05 were identified as DEGs. Afterward, we employed the online enrichment analysis tool DAVID (http://david.ncifcrf.gov) and Gene Ontology (GO) and Kyoto Encyclopedia of Genes and Genomes (KEGG) databases to identify the biological functions and pathways of the DEGs [44]. We regarded the enrichment terms with a Benjamini and Hochberg adjusted p-value of less than 0.05 as statistically significant.

### Identification of regulated cell death biomarkers and gene set variation analysis (GSVA)

To investigate the mechanisms underlying regulated cell death (RCD) in diabetic nephropathy (DN) samples, we searched the KEGG, GO and FerrDb v2 databases to identify RCD markers of five types, including autophagy, necroptosis, pyroptosis, apoptosis and ferroptosis [45]. To assess the enrichment of these RCD gene sets in the RNA sequencing datasets, we used gene set variation analysis (GSVA) to generate GSVA scores for each gene set, which evaluate the change in activity of the associated gene set [46]. We then created RCD gene sets from the collected RCD-related key genes and used them to assess the enrichment of various types of RCD. After calculating the GSVA scores of different RCD patterns, we selected RCD patterns with statistical differences (p-value<0.05) in RNA-seq data (both single-cell and bulk) for further investigation.

### Identification of key RCD-related gene modules

We employed the weighted gene co-expression network analysis (WGCNA) algorithm, a data reduction and unsupervised classification method, to build a gene co-expression network and confirm co-expressed genes and modules associated with RCD traits [47]. Gene modules were identified using hierarchical clustering trees, and topological overlap matrix-based hierarchical clustering was used to detect them. Pearson correlation coefficients were calculated to determine the correlation of each module with RCD features, and modules significantly associated with RCD traits (p-value<0.05) were identified. The genes within these modules were then exported for further analysis.

### Identification of key RCD-related biomarkers

In order to identify RCD-related biomarkers in DN samples, we first intersected the DEGs identified in both the single-cell and bulk RNA-seq data with the gene modules acquired by WGCNA. Subsequently, we investigated the expression profiles of these biomarkers in different types of renal cells using the single-cell sequencing dataset. Additionally, we also investigated the expression profiles of these candidate gene markers in bulk RNA-seq data. To assess the diagnostic ability of these gene markers, we employed the “ROCR” package to generate receiver operating characteristic (ROC) curves.

### Validation in the external test cohort

To further evaluate the robustness of the biomarkers obtained from the training cohort, we assessed their expression levels in different groups and generated receiver operating characteristic (ROC) curves based on an independent external dataset (GSE142025).

### Association between gene markers and clinical features

The Nephroseq database (http://www.nephroseq.org) is a valuable resource for investigating the association between key genes and important clinical renal parameters such as serum levels of creatine (SCr), glomerular filtration rate (GFR), and proteinuria (PRO) in DN patients. Therefore, we utilized the Nephroseq database to assess the correlation between key genes and these clinical parameters. Pearson correlation analysis was performed to investigate the relationship between key genes and GFR, SCr, and PRO in DN patients.

### Cell culture and treatment

HK-2 cells were obtained from the National Collection of Authenticated Cell Cultures and cultured in DMEM (HyClone; Cytiva, USA) supplemented with 10% FBS (HyClone; Cytiva, USA) at 37° C and 5% CO2. When cells reached 70%-80% confluency, the medium was replaced with serum-free culture medium and incubated for 24h. The cells were then divided into three groups: high glucose (HG, 30 mmol/L glucose), normal glucose (NG, 5.5 mmol/L glucose), and normal glucose with hyperosmotic medium (NG+M, 5.5 mmol/L glucose + 24.5 mmol/L mannitol). These groups were cultured for 48h, and the proteins were extracted for subsequent experiments. The cell assays were performed in triplicate.

### Western blot analysis

Protein was extracted from HK-2 cells using RIPA lysate (cat. no. P0013 Beyotime, China), and the protein concentration was quantified using the BCA kit (cat. no. P0010; Beyotime, China). A total of 50μg of protein was mixed with loading buffer in a 1:5 ratio, denatured at 100° C for 10 min, and then separated by SDS-PAGE and transferred onto a PVDF membrane (Bio-Rad Laboratories, Inc., USA) at 90V for 2h. The membranes were blocked using 5% skimmed milk powder for 2h at 37° C and then incubated overnight at 4° C with various primary antibodies, including anti-MLKL (cat. no. ab184718, Abcam, USA), anti-RIP3 (cat. no. ab209384, Abcam, USA), anti-RIP1 (cat. no. ab202985, Abcam, USA), anti-EGF (cat. no. ab218831, Abcam, USA), anti-ZFP36 (cat. no. orb39206, Biorbyt, UK), anti-PAG1 (cat. no. orb67086, Biorbyt, UK), and anti-beta actin (cat. no. ab115777, Abcam, USA), all diluted by 1:1000. The membranes were washed three times with TBST for 10 min each and then incubated with HRP-labelled secondary antibodies (cat.no. ab97051, Abcam, USA) diluted by 1:1000 for 1h. Finally, the ECL method was used for visualization, and the optical density of the bands was quantified using ImageJ (version 1.8). The experiments were performed in triplicate.

### Reverse transcription quantitative PCR (RT-qPCR) analysis

Lastly, we evaluated the mRNA expression levels of RIP1, RIP3, MLKL, EGF and ZFP36 in HK-2 cells using specific primers (see Supplementary Table 6). Total RNA was extracted from HK-2 cells using TRIzol reagent (Invitrogen, USA), and cDNA was synthesized using PrimeScript RT Master Mix (Takara, Japan). Real-time PCR was performed using SYBR Premix Ex Taq II (Takara, Japan) under the following conditions: (1) initial denaturation at 95° C for 30 s; (2) annealing at 95° C for 55 s and 60° C for 40 s, for 40 cycles; and (3) melting at 95° C for 15 s, 60° C for 60 s, and 95° C for 15 s. The endogenous control was β-actin, and data were analyzed using the 2-∆∆Cq method.

### Statistical analysis

In this study, all statistical analyses were performed using R software (version 4.2). For microarray data, we applied a screening threshold of |log2-fold change (FC)| ≥ 1 and a Benjamini and Hochberg adjusted p-value < 0.05. For single cell data, we considered a p-value < 0.05 and an absolute value of log2FC greater than 0.25 as screening conditions. The GSVA scores were compared between DN and control groups for each RCD gene set using appropriate statistical tests like Wilcoxon rank sum test. Only those RCD types showing significantly different GSVA scores between groups (p<0.05) were considered enriched and taken for further analysis. In WGCNA, only gene modules showing significant correlation with regulated cell death (p<0.05) were considered associated with the phenotype. To compare the expression of hub biomarkers between diabetic nephropathy and control groups, we used the Wilcoxon test.

### Data availability statement

All public datasets used in this study can be downloaded in GEO by searching the accession ID provided in this article. The data that support this finding are available within the article or its supplementary materials.

## Supplementary Material

Supplementary Table 1

Supplementary Table 2

Supplementary Table 3

Supplementary Table 4

Supplementary Tables 5 and 6
