# Supplementary material for: Investigating EGF and PAG1 as necroptosis-related biomarkers for diabetic nephropathy: an in silico and in vitro validation study
Source: Aging (Albany NY). 2023 Nov 20;15(22):13176–93. doi: 10.18632/aging.205233 (PMC10713428; doi:10.18632/aging.205233)
Supplement: Supplementary Table 1 [file aging-15-205233-s001.pdf]

SUPPLEMENTARY TABLE

Supplementary Table 1. Identification of cell types in scRNA-Seq data via highly variable markers.

Cell type	Cluster	Markers
Collecting duct Type A intercalated (CD-ICA) cell	1	SLC26A7, CLNK, ADGRF5
Collecting duct Type B intercalated (CD-ICB) cell	18	SLC26A4, SLC4A9, CELF2
Connecting tubule (CT) cell	5,6,10,12,25	SLC8A1, SNTG1, LSAMP
Distal convoluted tubule (DCT) cell	0,8	SLC12A3, TRPM6, CNNM2
Endothelium (ENDO) cell	13,20	LDB2, EMCN, MEIS2
Fibroblasts (FIB) cell	23	C7, NEGR1, TSHZ2, SVEP1
Leukocyte (LEUK) cell	19	ARHGAP15, PTPRC, PRKCB
Loop of Henle (LOH) cell	3,4,17,21,26	SLC12A1, PLCB1, RP1
Mesangial (MES) cell	22	CACNA1C, EBF1, NTRK3
Proximal convoluted tubule (PCT) cell	2,7,9,15	SORCS1, SLC5A12, UGT2B7
Parietal epithelial (PEC) cell	14	ALDH1A2, CFH, KCNT2
Podocyte (PODO) cell	16	PTPRQ, PTPRO, PLA2R1
Proximal straight tubule (PST) cell	11,24	ITGB8, VCAM1
