# Supplementary material for: Investigating EGF and PAG1 as necroptosis-related biomarkers for diabetic nephropathy: an in silico and in vitro validation study
Source: Aging (Albany NY). 2023 Nov 20;15(22):13176–93. doi: 10.18632/aging.205233 (PMC10713428; doi:10.18632/aging.205233)
Supplement: Supplementary Table 2 [file aging-15-205233-s002.docx]

**Supplementary Table 2. Differentially expressed genes in scRNA-seq data (log2|fold change|>0.25 and p-value<0.05).**

| **gene** | **P-value** | **avg log2FC** | **pct.1** | **pct.2** | **adjust p-value** | **cell type** |
| --- | --- | --- | --- | --- | --- | --- |
| ACSM2B | 1.16E-97 | -0.556156267 | 0.888 | 0.192 | 2.33E-94 | DCT |
| ACSM2A | 5.18E-66 | -0.529094763 | 0.86 | 0.216 | 1.04E-62 | DCT |
| MIOX | 8.60E-60 | -0.453187398 | 0.751 | 0.112 | 1.72E-56 | DCT |
| PRODH2 | 5.34E-140 | -0.41803197 | 0.808 | 0.082 | 1.07E-136 | DCT |
| AC092078.2 | 4.23E-24 | -0.417270988 | 0.95 | 0.412 | 8.47E-21 | DCT |
| AC093895.1 | 8.61E-124 | -0.400733976 | 0.95 | 0.213 | 1.72E-120 | DCT |
| LRRK2 | 2.61E-22 | -0.365316622 | 0.909 | 0.385 | 5.21E-19 | DCT |
| SLC5A12 | 1.78E-138 | -0.33764276 | 0.91 | 0.164 | 3.55E-135 | DCT |
| PTCHD1-AS | 1.77E-10 | -0.311990193 | 0.794 | 0.344 | 3.54E-07 | DCT |
| MEPE | 1.11E-66 | -0.311168517 | 0.862 | 0.219 | 2.22E-63 | DCT |
| RGS6 | 3.36E-12 | -0.311020527 | 0.472 | 0.113 | 6.72E-09 | DCT |
| SLC16A9 | 2.16E-42 | -0.29341221 | 0.772 | 0.174 | 4.31E-39 | DCT |
| UGT1A8 | 4.54E-09 | -0.287200015 | 0.524 | 0.189 | 9.09E-06 | DCT |
| KCNH7 | 3.54E-14 | -0.285596369 | 0.703 | 0.195 | 7.09E-11 | DCT |
| FKBP5 | 1.14E-95 | -0.275789806 | 0.919 | 0.224 | 2.27E-92 | DCT |
| SLC2A9 | 1.11E-23 | -0.265631391 | 0.444 | 0.116 | 2.22E-20 | DCT |
| SLC4A4 | 2.91E-42 | -0.265330508 | 0.414 | 0.164 | 5.83E-39 | DCT |
| LINC01505 | 5.39E-10 | -0.254179864 | 0.848 | 0.398 | 1.08E-06 | DCT |
| ZNF331 | 2.99E-24 | 0.265591394 | 0.448 | 0.172 | 5.98E-21 | DCT |
| USP43 | 2.73E-11 | 0.28054717 | 0.772 | 0.363 | 5.45E-08 | DCT |
| SLC20A1 | 1.21E-14 | 0.371671315 | 0.869 | 0.473 | 2.42E-11 | DCT |
| KCNIP4 | 1.93E-11 | 0.394253244 | 0.981 | 0.836 | 3.87E-08 | DCT |
| SLC8A1 | 1.18E-12 | 0.453672912 | 0.922 | 0.561 | 2.37E-09 | DCT |
| DNER | 1.60E-19 | 0.476193082 | 0.832 | 0.413 | 3.21E-16 | DCT |
| ALAS1 | 3.98E-252 | -0.664688777 | 0.942 | 0.162 | 7.96E-249 | CT |
| PRODH2 | 2.05E-246 | -0.252436125 | 0.86 | 0.087 | 4.10E-243 | CT |
| MIOX | 8.67E-224 | -0.497014849 | 0.866 | 0.108 | 1.73E-220 | CT |
| SLC12A3 | 1.84E-200 | -0.467518169 | 0.899 | 0.162 | 3.67E-197 | CT |
| SLC5A12 | 2.13E-188 | -0.255399274 | 0.898 | 0.167 | 4.27E-185 | CT |
| ZFP36 | 1.24E-165 | -0.36447354 | 0.947 | 0.237 | 2.47E-162 | CT |
| ACSM2B | 6.75E-118 | -0.534593228 | 0.844 | 0.178 | 1.35E-114 | CT |
| ACSM2A | 7.05E-88 | -0.638009577 | 0.846 | 0.218 | 1.41E-84 | CT |
| LTBP1 | 1.59E-39 | -0.3806629 | 0.737 | 0.182 | 3.18E-36 | CT |
| SGCD | 2.90E-39 | -0.619901743 | 0.829 | 0.296 | 5.81E-36 | CT |
| CPM | 8.40E-29 | 0.293132458 | 0.698 | 0.175 | 1.68E-25 | CT |
| MIR222HG | 3.85E-28 | 0.287017277 | 0.706 | 0.2 | 7.70E-25 | CT |
| FGF13 | 5.54E-25 | 0.333658707 | 0.774 | 0.311 | 1.11E-21 | CT |
| TMEM178A | 7.72E-25 | -0.425936585 | 0.979 | 0.455 | 1.54E-21 | CT |
| HSPA1A | 4.07E-21 | -0.278029762 | 0.748 | 0.246 | 8.14E-18 | CT |
| ITGAV | 1.83E-20 | 0.254249909 | 0.915 | 0.548 | 3.65E-17 | CT |
| HSPA1B | 2.10E-17 | -0.369026329 | 0.862 | 0.386 | 4.19E-14 | CT |
| S100A2 | 8.31E-14 | 0.470459701 | 0.432 | 0.087 | 1.66E-10 | CT |
| KLHL13 | 1.35E-13 | 0.256135278 | 0.814 | 0.435 | 2.70E-10 | CT |
| ITGB6 | 7.56E-13 | 0.401332026 | 0.737 | 0.336 | 1.51E-09 | CT |
| UPP1 | 2.14E-09 | 0.297789754 | 0.485 | 0.098 | 4.28E-06 | CT |
| CYTOR | 1.29E-07 | 0.358590145 | 0.673 | 0.269 | 0.000258643 | CT |
| MET | 3.95E-07 | 0.40558937 | 0.662 | 0.239 | 0.000789002 | CT |
| GLS | 1.25E-06 | 0.330416042 | 0.97 | 0.87 | 0.002508042 | CT |
| CUBN | 1.74E-06 | -0.352104417 | 0.744 | 0.312 | 0.003487297 | CT |
| GADD45B | 1.71E-203 | -0.498504334 | 0.995 | 0.22 | 3.43E-200 | LOH |
| SLC5A12 | 2.10E-193 | -0.317366726 | 0.938 | 0.181 | 4.20E-190 | LOH |
| LINC01055 | 2.76E-192 | -0.326449226 | 0.955 | 0.197 | 5.53E-189 | LOH |
| GRM8 | 3.35E-179 | 0.402290793 | 0.249 | 0.057 | 6.71E-176 | LOH |
| CDH20 | 1.30E-163 | -0.284483139 | 0.977 | 0.244 | 2.60E-160 | LOH |
| SLC12A3 | 1.22E-143 | -0.26354733 | 0.846 | 0.141 | 2.45E-140 | LOH |
| ZFP36 | 1.25E-139 | -0.399547328 | 0.983 | 0.262 | 2.49E-136 | LOH |
| FOS | 1.47E-133 | -0.267799227 | 0.87 | 0.172 | 2.93E-130 | LOH |
| CYTIP | 3.29E-127 | -0.33956011 | 0.979 | 0.268 | 6.58E-124 | LOH |
| MT2A | 1.49E-126 | -0.348007951 | 0.879 | 0.187 | 2.98E-123 | LOH |
| SLC16A9 | 8.24E-123 | -0.272094874 | 0.894 | 0.208 | 1.65E-119 | LOH |
| EGR1 | 4.39E-101 | -0.335585731 | 0.864 | 0.203 | 8.78E-98 | LOH |
| TMEM132C | 6.94E-83 | -0.700424634 | 0.962 | 0.31 | 1.39E-79 | LOH |
| TMEM178B | 1.13E-75 | 0.251597977 | 0.368 | 0.154 | 2.25E-72 | LOH |
| ACSM2B | 1.10E-68 | -0.560517731 | 0.81 | 0.192 | 2.20E-65 | LOH |
| SPP1 | 9.08E-66 | -0.81745367 | 0.866 | 0.263 | 1.82E-62 | LOH |
| S100A2 | 1.17E-64 | -0.250278297 | 0.828 | 0.222 | 2.33E-61 | LOH |
| KCNQ3 | 2.94E-62 | -0.25817011 | 0.729 | 0.113 | 5.89E-59 | LOH |
| TIPARP | 6.34E-62 | -0.512358959 | 0.994 | 0.379 | 1.27E-58 | LOH |
| MIR222HG | 1.84E-61 | -0.411620432 | 0.948 | 0.346 | 3.68E-58 | LOH |
| MIOX | 2.69E-60 | -0.36177236 | 0.72 | 0.113 | 5.38E-57 | LOH |
| CACNA1C | 9.73E-59 | -0.372451061 | 0.911 | 0.308 | 1.95E-55 | LOH |
| AC008415.1 | 6.45E-53 | 0.358592498 | 0.34 | 0.027 | 1.29E-49 | LOH |
| LINC01811 | 3.03E-50 | 0.473641272 | 0.698 | 0.101 | 6.06E-47 | LOH |
| GPR137B | 1.18E-49 | -0.261203346 | 0.937 | 0.34 | 2.36E-46 | LOH |
| COL8A1 | 1.37E-49 | -0.682860136 | 0.987 | 0.402 | 2.74E-46 | LOH |
| CPNE4 | 4.20E-42 | -0.380465872 | 0.821 | 0.256 | 8.40E-39 | LOH |
| ACSM2A | 4.46E-41 | -0.536247144 | 0.783 | 0.22 | 8.92E-38 | LOH |
| ACTN1 | 2.21E-40 | -0.306070243 | 0.895 | 0.331 | 4.42E-37 | LOH |
| RGS6 | 1.75E-38 | 0.439610816 | 0.687 | 0.135 | 3.51E-35 | LOH |
| FBXL7 | 2.69E-27 | 0.275575247 | 0.42 | 0.053 | 5.38E-24 | LOH |
| GS1-24F4.2 | 2.99E-27 | -0.298746699 | 0.868 | 0.356 | 5.99E-24 | LOH |
| CUBN | 4.63E-24 | -0.276240883 | 0.813 | 0.319 | 9.25E-21 | LOH |
| AC005699.1 | 1.54E-20 | 0.267430121 | 0.45 | 0.103 | 3.09E-17 | LOH |
| TMEM207 | 2.46E-20 | -0.278637792 | 0.735 | 0.269 | 4.91E-17 | LOH |
| P4HA1 | 1.21E-17 | -0.308257164 | 0.996 | 0.498 | 2.42E-14 | LOH |
| TENM4 | 1.20E-16 | 0.321174248 | 0.816 | 0.464 | 2.39E-13 | LOH |
| HOMER1 | 2.52E-13 | -0.38453807 | 0.918 | 0.494 | 5.03E-10 | LOH |
| PXDNL | 1.58E-11 | 0.364008431 | 0.663 | 0.225 | 3.15E-08 | LOH |
| PAPPA2 | 4.76E-08 | -0.37460235 | 0.774 | 0.354 | 9.52E-05 | LOH |
| PPFIBP1 | 6.28E-08 | 0.299494961 | 0.763 | 0.412 | 0.000125515 | LOH |
| RP1 | 7.10E-07 | 0.379546258 | 0.999 | 0.864 | 0.001419585 | LOH |
| IL1RAPL2 | 8.56E-07 | -0.325754364 | 0.745 | 0.32 | 0.001712599 | LOH |
| RANBP3L | 2.57E-06 | -0.460974483 | 0.853 | 0.71 | 0.005130356 | LOH |
| SLC8A1-AS1 | 1.40E-299 | -0.276019166 | 0.214 | 0.144 | 2.79E-296 | PCT |
| CYP24A1 | 2.89E-181 | 0.263671231 | 0.281 | 0.035 | 5.79E-178 | PCT |
| AC107220.1 | 4.61E-160 | 0.338995792 | 0.283 | 0.018 | 9.22E-157 | PCT |
| NAV3 | 1.87E-121 | -0.333983299 | 0.863 | 0.201 | 3.73E-118 | PCT |
| DNAH5 | 1.72E-110 | 0.530422698 | 0.333 | 0.065 | 3.45E-107 | PCT |
| TIPARP | 2.41E-100 | -0.423631066 | 0.999 | 0.344 | 4.81E-97 | PCT |
| MEG3 | 4.91E-80 | -0.279979352 | 0.377 | 0.108 | 9.83E-77 | PCT |
| SLC26A7 | 7.91E-73 | -0.466202968 | 0.404 | 0.151 | 1.58E-69 | PCT |
| AC011287.1 | 1.21E-69 | -0.335149693 | 0.873 | 0.275 | 2.42E-66 | PCT |
| FTL | 1.54E-66 | -0.348248339 | 1 | 0.422 | 3.08E-63 | PCT |
| SLC8A1 | 3.09E-58 | -0.671256662 | 0.484 | 0.33 | 6.17E-55 | PCT |
| UNC5D | 6.03E-44 | -0.322986804 | 0.735 | 0.173 | 1.21E-40 | PCT |
| FOXP2 | 2.37E-40 | -0.250070936 | 0.492 | 0.288 | 4.74E-37 | PCT |
| TUBGCP3 | 1.84E-35 | 0.523464525 | 0.792 | 0.326 | 3.69E-32 | PCT |
| RHEX | 1.66E-27 | -0.27416955 | 0.484 | 0.184 | 3.31E-24 | PCT |
| ZNF804B | 3.94E-27 | 0.303292061 | 0.999 | 0.547 | 7.89E-24 | PCT |
| CLSTN2 | 4.16E-27 | 0.349900168 | 0.466 | 0.185 | 8.33E-24 | PCT |
| LDLRAD4 | 1.50E-22 | -0.392244352 | 0.907 | 0.398 | 3.01E-19 | PCT |
| RDX | 1.00E-19 | 0.364824943 | 0.899 | 0.565 | 2.01E-16 | PCT |
| CDH20 | 2.64E-19 | 0.387216915 | 0.765 | 0.338 | 5.29E-16 | PCT |
| LINC01060 | 1.23E-13 | 0.271977997 | 0.99 | 0.743 | 2.46E-10 | PCT |
| SORCS1 | 1.32E-13 | 0.394827573 | 0.997 | 0.79 | 2.64E-10 | PCT |
| UGT1A8 | 1.73E-12 | 0.285098223 | 0.985 | 0.876 | 3.46E-09 | PCT |
| DGKG | 5.29E-09 | 0.26951861 | 0.523 | 0.208 | 1.06E-05 | PCT |
| PLPPR1 | 2.15E-08 | -0.413928504 | 0.694 | 0.508 | 4.30E-05 | PCT |
| LRP2 | 3.68E-08 | -0.254988348 | 0.984 | 0.88 | 7.36E-05 | PCT |
| ERRFI1 | 5.44E-08 | 0.251531158 | 0.998 | 0.811 | 0.000108869 | PCT |
| AC096577.1 | 1.32E-07 | 0.312409637 | 0.976 | 0.798 | 0.000264288 | PCT |
| AL110292.1 | 2.13E-05 | 0.333039741 | 0.765 | 0.512 | 0.0425387 | PCT |
| CAVIN2-AS1 | 4.35E-208 | -0.257976892 | 0.991 | 0.075 | 8.69E-205 | CD-ICA |
| ANO4 | 1.70E-145 | 0.431641894 | 0.16 | 0.047 | 3.40E-142 | CD-ICA |
| RGS6 | 1.28E-130 | -0.304030258 | 0.18 | 0.099 | 2.56E-127 | CD-ICA |
| AL031599.1 | 2.95E-122 | -0.404091709 | 0.994 | 0.169 | 5.90E-119 | CD-ICA |
| MME | 1.48E-104 | -0.288456366 | 0.987 | 0.189 | 2.96E-101 | CD-ICA |
| TRAF3IP3 | 2.43E-100 | -0.268027005 | 0.993 | 0.196 | 4.85E-97 | CD-ICA |
| MIOX | 1.51E-99 | -0.394453816 | 0.918 | 0.139 | 3.02E-96 | CD-ICA |
| ACSM2B | 3.35E-74 | -0.535102755 | 0.955 | 0.214 | 6.71E-71 | CD-ICA |
| PPM1E | 1.02E-73 | 0.427771604 | 0.265 | 0.057 | 2.04E-70 | CD-ICA |
| PCDH15 | 1.61E-69 | -0.251097812 | 0.293 | 0.191 | 3.21E-66 | CD-ICA |
| SOX5 | 8.15E-63 | -0.264278018 | 0.308 | 0.237 | 1.63E-59 | CD-ICA |
| HSPA5 | 2.06E-44 | 0.444281934 | 0.332 | 0.161 | 4.13E-41 | CD-ICA |
| CD83 | 1.30E-43 | 0.251951456 | 0.338 | 0.139 | 2.60E-40 | CD-ICA |
| SLC8A1-AS1 | 9.05E-40 | -0.411538429 | 0.384 | 0.288 | 1.81E-36 | CD-ICA |
| NPSR1-AS1 | 1.78E-39 | -0.31602169 | 0.965 | 0.307 | 3.55E-36 | CD-ICA |
| AC010967.1 | 3.22E-38 | -0.255065275 | 0.742 | 0.093 | 6.43E-35 | CD-ICA |
| MARCHF1 | 2.85E-37 | -0.481558958 | 0.865 | 0.236 | 5.70E-34 | CD-ICA |
| TMTC1 | 2.77E-33 | 0.31122287 | 0.364 | 0.194 | 5.54E-30 | CD-ICA |
| ACSM2A | 5.77E-31 | -0.525788349 | 0.861 | 0.242 | 1.15E-27 | CD-ICA |
| GPR137B | 2.90E-28 | 0.298391371 | 0.8 | 0.215 | 5.81E-25 | CD-ICA |
| SLC16A9 | 2.39E-21 | -0.255158512 | 0.74 | 0.153 | 4.79E-18 | CD-ICA |
| KCNJ15 | 4.54E-21 | -0.306132532 | 0.87 | 0.294 | 9.09E-18 | CD-ICA |
| TEX41 | 6.48E-19 | -0.504845872 | 0.462 | 0.235 | 1.30E-15 | CD-ICA |
| SLC4A1 | 6.52E-17 | 0.256349872 | 0.935 | 0.462 | 1.30E-13 | CD-ICA |
| DDIT3 | 2.16E-16 | 0.412308863 | 0.414 | 0.101 | 4.31E-13 | CD-ICA |
| EYA4 | 4.68E-15 | -0.275903371 | 0.751 | 0.203 | 9.35E-12 | CD-ICA |
| UNC5D | 4.50E-13 | -0.254196964 | 0.902 | 0.39 | 9.00E-10 | CD-ICA |
| TCF7L1 | 4.92E-13 | 0.254151301 | 0.915 | 0.489 | 9.83E-10 | CD-ICA |
| ELF3 | 1.42E-12 | 0.352820965 | 0.44 | 0.134 | 2.85E-09 | CD-ICA |
| SQSTM1 | 2.70E-12 | 0.282749839 | 0.46 | 0.23 | 5.39E-09 | CD-ICA |
| LINC00871 | 4.46E-12 | -0.278893798 | 0.481 | 0.183 | 8.93E-09 | CD-ICA |
| PSD3 | 1.93E-11 | 0.424939768 | 0.996 | 0.886 | 3.85E-08 | CD-ICA |
| LEF1 | 5.72E-11 | 0.38621111 | 0.993 | 0.716 | 1.14E-07 | CD-ICA |
| DMD | 2.04E-10 | 0.29714663 | 0.993 | 0.635 | 4.08E-07 | CD-ICA |
| LBH | 3.30E-10 | 0.330692505 | 0.456 | 0.136 | 6.60E-07 | CD-ICA |
| LINC02343 | 3.08E-09 | 0.412051985 | 0.946 | 0.641 | 6.17E-06 | CD-ICA |
| FNDC3A | 4.62E-09 | 0.296420906 | 1 | 0.914 | 9.23E-06 | CD-ICA |
| SLC8A1 | 9.59E-09 | -0.787530511 | 0.655 | 0.546 | 1.92E-05 | CD-ICA |
| AQP6 | 2.45E-08 | 0.343439629 | 0.991 | 0.692 | 4.90E-05 | CD-ICA |
| CA12 | 1.00E-07 | 0.25475983 | 0.996 | 0.885 | 0.000200429 | CD-ICA |
| KIT | 2.29E-07 | 0.342152198 | 0.994 | 0.829 | 0.00045854 | CD-ICA |
| IPCEF1 | 2.58E-07 | 0.46520596 | 0.983 | 0.692 | 0.000516395 | CD-ICA |
| AL359736.1 | 3.70E-07 | 0.298643278 | 0.959 | 0.789 | 0.000739893 | CD-ICA |
| GULP1 | 2.88E-06 | 0.256755233 | 0.97 | 0.738 | 0.00575185 | CD-ICA |
| PDE3A | 3.17E-06 | 0.475486114 | 0.865 | 0.492 | 0.006333244 | CD-ICA |
| HSP90B1 | 5.06E-06 | 0.74565436 | 0.538 | 0.306 | 0.010111288 | CD-ICA |
| KCTD16 | 1.70E-113 | 0.256171948 | 0.106 | 0.034 | 3.40E-110 | ENDO |
| SLC5A12 | 2.97E-76 | -0.355547844 | 0.993 | 0.175 | 5.93E-73 | ENDO |
| PRODH2 | 1.93E-71 | -0.295539011 | 0.888 | 0.087 | 3.86E-68 | ENDO |
| BMPR1B | 5.25E-55 | -0.367322294 | 0.949 | 0.194 | 1.05E-51 | ENDO |
| AC096577.1 | 1.18E-54 | -0.300287275 | 0.839 | 0.083 | 2.35E-51 | ENDO |
| CXXC4-AS1 | 8.70E-50 | -0.280061523 | 0.886 | 0.149 | 1.74E-46 | ENDO |
| HDAC9 | 3.85E-49 | 0.40064576 | 0.256 | 0.1 | 7.70E-46 | ENDO |
| CPM | 1.01E-48 | -0.440735485 | 0.928 | 0.19 | 2.02E-45 | ENDO |
| RGCC | 5.08E-48 | -0.359321431 | 0.814 | 0.156 | 1.02E-44 | ENDO |
| SLC13A1 | 1.04E-46 | -0.255684835 | 0.827 | 0.094 | 2.08E-43 | ENDO |
| ACSM2B | 1.45E-46 | -0.570826463 | 0.93 | 0.2 | 2.91E-43 | ENDO |
| ACSM2A | 1.16E-42 | -0.566328798 | 0.951 | 0.235 | 2.32E-39 | ENDO |
| DOCK8 | 2.29E-40 | 0.277418972 | 0.282 | 0.058 | 4.58E-37 | ENDO |
| ERRFI1 | 1.30E-39 | -0.341065568 | 0.933 | 0.228 | 2.59E-36 | ENDO |
| SLC16A9 | 1.52E-39 | -0.38640592 | 0.856 | 0.151 | 3.03E-36 | ENDO |
| ADAMTS1 | 2.25E-38 | 0.447564205 | 0.289 | 0.077 | 4.51E-35 | ENDO |
| SLC16A12 | 2.66E-38 | -0.520536361 | 0.872 | 0.171 | 5.31E-35 | ENDO |
| CYP3A5 | 3.01E-37 | -0.571152176 | 0.892 | 0.196 | 6.02E-34 | ENDO |
| PTPRQ | 1.75E-36 | -0.267087749 | 0.778 | 0.077 | 3.50E-33 | ENDO |
| VCAM1 | 6.07E-36 | 0.742775526 | 0.182 | 0.036 | 1.21E-32 | ENDO |
| ENPP2 | 1.57E-35 | 0.708164394 | 0.298 | 0.075 | 3.13E-32 | ENDO |
| LINC00639 | 1.64E-35 | 0.293232498 | 0.155 | 0.034 | 3.28E-32 | ENDO |
| HSPB1 | 1.76E-35 | 0.299243477 | 0.305 | 0.104 | 3.53E-32 | ENDO |
| MT1M | 2.14E-35 | -0.364576678 | 0.814 | 0.134 | 4.29E-32 | ENDO |
| SEMA3A | 2.66E-34 | 0.295456254 | 0.305 | 0.075 | 5.32E-31 | ENDO |
| AC005699.1 | 3.97E-34 | 0.328940165 | 0.74 | 0.043 | 7.93E-31 | ENDO |
| NFATC2 | 6.52E-32 | 0.269417926 | 0.323 | 0.132 | 1.30E-28 | ENDO |
| PDE1C | 1.48E-29 | -0.264071784 | 0.827 | 0.164 | 2.97E-26 | ENDO |
| MIOX | 5.16E-29 | -0.349069509 | 0.783 | 0.115 | 1.03E-25 | ENDO |
| CA4 | 1.13E-28 | -0.386884593 | 0.765 | 0.139 | 2.25E-25 | ENDO |
| SLC6A13 | 1.16E-28 | -0.292406108 | 0.774 | 0.107 | 2.32E-25 | ENDO |
| SLC22A6 | 1.60E-28 | -0.32098881 | 0.767 | 0.098 | 3.19E-25 | ENDO |
| SORCS1 | 2.21E-28 | -0.416111504 | 0.816 | 0.156 | 4.43E-25 | ENDO |
| DEPTOR | 3.11E-28 | -0.339457089 | 0.879 | 0.224 | 6.21E-25 | ENDO |
| SCNN1G | 2.61E-26 | -0.270095811 | 0.632 | 0.072 | 5.22E-23 | ENDO |
| RNF212B | 1.50E-25 | -0.33367149 | 0.776 | 0.124 | 3.01E-22 | ENDO |
| KL | 2.98E-25 | -0.27160281 | 0.356 | 0.119 | 5.97E-22 | ENDO |
| DCDC2 | 4.39E-25 | -0.629597627 | 0.85 | 0.207 | 8.79E-22 | ENDO |
| CCDC3 | 6.97E-25 | 0.353564956 | 0.305 | 0.055 | 1.39E-21 | ENDO |
| SLC26A7 | 1.54E-23 | -0.393516144 | 0.863 | 0.228 | 3.07E-20 | ENDO |
| AL390334.1 | 2.67E-23 | -0.302728975 | 0.801 | 0.166 | 5.34E-20 | ENDO |
| RELN | 9.91E-23 | 0.3888684 | 0.348 | 0.064 | 1.98E-19 | ENDO |
| SLIT2 | 3.51E-22 | -0.309572952 | 0.868 | 0.247 | 7.02E-19 | ENDO |
| UGT1A8 | 1.22E-21 | -0.349678864 | 0.807 | 0.181 | 2.44E-18 | ENDO |
| PCDH17 | 2.28E-21 | 0.256066608 | 0.372 | 0.166 | 4.56E-18 | ENDO |
| AL357507.1 | 1.46E-20 | 0.280657249 | 0.361 | 0.075 | 2.91E-17 | ENDO |
| TM4SF1 | 9.32E-20 | 0.260803787 | 0.357 | 0.09 | 1.86E-16 | ENDO |
| AC087633.2 | 5.63E-19 | 0.321424302 | 0.785 | 0.194 | 1.13E-15 | ENDO |
| EMP1 | 2.00E-18 | 0.360605863 | 0.807 | 0.247 | 3.99E-15 | ENDO |
| H2AC18 | 8.98E-18 | 0.433426584 | 0.323 | 0.041 | 1.80E-14 | ENDO |
| CNTN5 | 3.09E-17 | 0.304730339 | 0.363 | 0.06 | 6.17E-14 | ENDO |
| NRP2 | 1.17E-16 | 0.2973951 | 0.522 | 0.092 | 2.33E-13 | ENDO |
| CDK6 | 3.78E-16 | -0.302514648 | 0.478 | 0.382 | 7.55E-13 | ENDO |
| PIK3C2G | 8.57E-16 | -0.454981267 | 0.764 | 0.168 | 1.71E-12 | ENDO |
| C1orf112 | 3.70E-15 | 0.347499779 | 0.39 | 0.087 | 7.40E-12 | ENDO |
| ESM1 | 1.85E-14 | -0.510640517 | 0.733 | 0.149 | 3.69E-11 | ENDO |
| AC004593.2 | 1.90E-14 | 0.387349808 | 0.444 | 0.279 | 3.81E-11 | ENDO |
| LAMA4 | 7.73E-14 | 0.319000176 | 0.397 | 0.092 | 1.55E-10 | ENDO |
| MGP | 1.07E-13 | 0.279033106 | 0.695 | 0.109 | 2.14E-10 | ENDO |
| FGF13 | 1.93E-13 | -0.586212178 | 0.431 | 0.16 | 3.85E-10 | ENDO |
| GPAT3 | 6.47E-13 | -0.411221832 | 0.745 | 0.168 | 1.29E-09 | ENDO |
| VEGFC | 1.51E-12 | 0.435820404 | 0.426 | 0.211 | 3.02E-09 | ENDO |
| PAH | 4.02E-12 | -0.258049484 | 0.686 | 0.098 | 8.05E-09 | ENDO |
| SLC13A3 | 5.54E-12 | -0.286960614 | 0.704 | 0.124 | 1.11E-08 | ENDO |
| PALMD | 1.76E-11 | 0.263467865 | 0.809 | 0.316 | 3.51E-08 | ENDO |
| MAP2 | 4.43E-11 | 0.450851438 | 0.776 | 0.258 | 8.86E-08 | ENDO |
| ABCA10 | 4.52E-11 | -0.314179387 | 0.69 | 0.115 | 9.05E-08 | ENDO |
| TSPAN5 | 2.43E-10 | 0.283243004 | 0.668 | 0.098 | 4.87E-07 | ENDO |
| DPYSL3 | 7.78E-10 | 0.271856254 | 0.601 | 0.122 | 1.56E-06 | ENDO |
| TCIM | 1.01E-09 | 0.397198365 | 0.448 | 0.19 | 2.02E-06 | ENDO |
| H2AC6 | 2.79E-09 | 0.353877217 | 0.421 | 0.075 | 5.59E-06 | ENDO |
| SOX2-OT | 3.85E-09 | -0.418309675 | 0.449 | 0.13 | 7.71E-06 | ENDO |
| DUSP5 | 5.75E-09 | 0.618019442 | 0.44 | 0.151 | 1.15E-05 | ENDO |
| CACNA2D3 | 8.42E-09 | -0.331333922 | 0.695 | 0.143 | 1.68E-05 | ENDO |
| MCTP1 | 2.04E-08 | 0.375700985 | 0.451 | 0.158 | 4.07E-05 | ENDO |
| LINC02197 | 2.21E-08 | 0.318465419 | 0.247 | 0.032 | 4.41E-05 | ENDO |
| GRAMD1B | 2.42E-08 | -0.363992178 | 0.404 | 0.111 | 4.84E-05 | ENDO |
| PDK4 | 4.86E-08 | -0.779043559 | 0.966 | 0.431 | 9.71E-05 | ENDO |
| GPM6A | 6.38E-08 | 0.277393699 | 0.412 | 0.22 | 0.000127558 | ENDO |
| CNTN1 | 7.55E-08 | 0.347456126 | 0.565 | 0.038 | 0.000151005 | ENDO |
| ID2 | 1.63E-07 | -0.321644021 | 0.704 | 0.168 | 0.00032605 | ENDO |
| KCNMB4 | 1.63E-07 | -0.26995457 | 0.863 | 0.373 | 0.000326614 | ENDO |
| SLC12A3 | 1.76E-07 | -0.660442973 | 0.664 | 0.115 | 0.000352476 | ENDO |
| TOX3 | 1.93E-07 | -0.428818953 | 0.711 | 0.179 | 0.000386228 | ENDO |
| IL1RAPL2 | 5.06E-07 | -0.300056753 | 0.468 | 0.132 | 0.001012146 | ENDO |
| AFF3 | 7.20E-07 | 0.351831632 | 0.661 | 0.134 | 0.001440246 | ENDO |
| RHOBTB3 | 9.60E-07 | -0.360658381 | 0.724 | 0.205 | 0.001920921 | ENDO |
| TRPM7 | 1.01E-06 | -0.484562017 | 0.614 | 0.446 | 0.002021638 | ENDO |
| HLA-DRB5 | 1.12E-06 | -0.425122896 | 0.558 | 0.326 | 0.002245127 | ENDO |
| AC119674.1 | 1.35E-06 | -0.296561773 | 0.653 | 0.58 | 0.002690472 | ENDO |
| FOSB | 2.34E-06 | -0.290461518 | 0.621 | 0.098 | 0.004677151 | ENDO |
| FRY | 3.48E-06 | 0.322279644 | 0.782 | 0.358 | 0.006959418 | ENDO |
| LRP2 | 4.14E-06 | -0.304724663 | 0.717 | 0.205 | 0.008283805 | ENDO |
| ST8SIA6 | 4.54E-06 | 0.344382445 | 0.449 | 0.085 | 0.009085218 | ENDO |
| PTPRO | 5.45E-06 | -0.297471892 | 0.567 | 0.07 | 0.010903203 | ENDO |
| PCED1B | 7.20E-06 | -0.269532541 | 0.623 | 0.514 | 0.01440534 | ENDO |
| SNTG1 | 1.22E-05 | -0.307922708 | 0.484 | 0.136 | 0.024376686 | ENDO |
| LMNA | 1.66E-05 | 0.254232013 | 0.67 | 0.171 | 0.033176753 | ENDO |
| PCK1 | 2.16E-82 | -0.416393925 | 0.943 | 0.124 | 4.31E-79 | PST |
| LINC02740 | 3.14E-82 | 0.354349179 | 0.682 | 0.011 | 6.28E-79 | PST |
| RRAD | 1.42E-81 | -0.680692241 | 0.981 | 0.159 | 2.84E-78 | PST |
| GLUL | 3.72E-80 | -0.340143403 | 0.952 | 0.137 | 7.44E-77 | PST |
| EDN1 | 4.60E-72 | -0.483424732 | 0.985 | 0.183 | 9.20E-69 | PST |
| KCND2 | 1.18E-69 | -0.281733317 | 0.87 | 0.108 | 2.37E-66 | PST |
| AFM | 2.44E-69 | -0.258305784 | 0.982 | 0.194 | 4.88E-66 | PST |
| LINC00511 | 3.28E-67 | 0.291031029 | 0.211 | 0.042 | 6.56E-64 | PST |
| SLC12A3 | 5.75E-67 | -0.405537917 | 0.925 | 0.146 | 1.15E-63 | PST |
| AC079298.3 | 1.06E-62 | 0.363256973 | 0.227 | 0.091 | 2.11E-59 | PST |
| ALDOB | 3.45E-62 | -0.430119775 | 0.99 | 0.214 | 6.89E-59 | PST |
| ADGRF5 | 7.07E-59 | -0.256678902 | 0.881 | 0.121 | 1.41E-55 | PST |
| TIPARP | 8.37E-59 | -0.316030675 | 0.956 | 0.196 | 1.67E-55 | PST |
| SGCD | 9.25E-46 | -0.348686454 | 0.927 | 0.21 | 1.85E-42 | PST |
| BIRC3 | 2.04E-44 | -0.404321812 | 0.899 | 0.19 | 4.09E-41 | PST |
| GDF15 | 7.17E-42 | -0.335764788 | 0.321 | 0.256 | 1.43E-38 | PST |
| SLC34A1 | 2.60E-38 | -0.57616245 | 0.959 | 0.26 | 5.20E-35 | PST |
| FUT9 | 1.80E-37 | 0.39499084 | 0.712 | 0.011 | 3.59E-34 | PST |
| PSD3 | 1.40E-35 | 0.350547879 | 0.309 | 0.124 | 2.81E-32 | PST |
| TRAF1 | 3.16E-34 | -0.405783674 | 0.871 | 0.196 | 6.32E-31 | PST |
| ODC1 | 2.62E-33 | 0.265565844 | 0.738 | 0.051 | 5.24E-30 | PST |
| CCL2 | 4.52E-33 | -0.552222208 | 0.799 | 0.124 | 9.05E-30 | PST |
| SLC4A11 | 7.82E-33 | 0.327555371 | 0.362 | 0.002 | 1.56E-29 | PST |
| ZFPM2 | 1.56E-32 | 0.382301597 | 0.322 | 0.157 | 3.12E-29 | PST |
| UBTD1 | 1.61E-31 | 0.289969852 | 0.94 | 0.331 | 3.23E-28 | PST |
| GADD45B | 2.03E-31 | -0.664137445 | 0.974 | 0.298 | 4.07E-28 | PST |
| ELF3 | 1.41E-29 | -0.436615419 | 0.395 | 0.371 | 2.82E-26 | PST |
| MT1M | 1.42E-29 | -0.325683178 | 0.684 | 0.06 | 2.85E-26 | PST |
| H2AC19 | 4.08E-29 | 0.259534191 | 0.25 | 0.009 | 8.16E-26 | PST |
| ID3 | 2.04E-28 | 0.272040132 | 0.127 | 0.007 | 4.07E-25 | PST |
| GRAMD1B | 8.66E-28 | -0.251369338 | 0.871 | 0.232 | 1.73E-24 | PST |
| ST18 | 3.97E-27 | -0.385971634 | 0.899 | 0.252 | 7.93E-24 | PST |
| PLCXD3 | 6.07E-27 | -0.304738574 | 0.881 | 0.243 | 1.21E-23 | PST |
| FRMD5 | 5.99E-25 | 0.305837617 | 0.35 | 0.11 | 1.20E-21 | PST |
| ADGRL3 | 6.41E-25 | 1.145547646 | 0.725 | 0.084 | 1.28E-21 | PST |
| NOL4 | 4.59E-24 | 0.384287914 | 0.709 | 0.064 | 9.18E-21 | PST |
| AL117190.1 | 2.68E-23 | -0.325635236 | 0.96 | 0.336 | 5.35E-20 | PST |
| SLC44A5 | 3.71E-22 | 0.606430412 | 0.76 | 0.163 | 7.43E-19 | PST |
| TEX41 | 3.78E-22 | 0.321370101 | 0.824 | 0.234 | 7.57E-19 | PST |
| PDK4 | 5.06E-22 | -0.541866823 | 0.99 | 0.362 | 1.01E-18 | PST |
| AC004870.4 | 5.45E-22 | 0.379746054 | 0.76 | 0.15 | 1.09E-18 | PST |
| H2AC18 | 1.22E-21 | 0.603577986 | 0.341 | 0.046 | 2.44E-18 | PST |
| SGK1 | 1.35E-20 | -0.566021817 | 0.924 | 0.298 | 2.69E-17 | PST |
| RGS6 | 2.36E-20 | 1.10269825 | 0.356 | 0.068 | 4.71E-17 | PST |
| MEG8 | 3.29E-20 | -0.325223889 | 0.955 | 0.344 | 6.59E-17 | PST |
| SLC39A14 | 6.63E-20 | -0.293887882 | 0.9 | 0.298 | 1.33E-16 | PST |
| MT1E | 9.88E-20 | -0.984651245 | 0.997 | 0.36 | 1.98E-16 | PST |
| PRAG1 | 2.97E-19 | 0.334377879 | 0.798 | 0.227 | 5.94E-16 | PST |
| SLC36A2 | 1.20E-18 | -0.322387683 | 1 | 0.386 | 2.40E-15 | PST |
| MT1H | 1.81E-18 | -1.018658731 | 0.999 | 0.36 | 3.62E-15 | PST |
| NEBL | 4.61E-18 | 0.544136052 | 0.958 | 0.525 | 9.23E-15 | PST |
| MT1G | 8.10E-18 | -1.205515385 | 0.996 | 0.369 | 1.62E-14 | PST |
| AC003984.1 | 1.31E-17 | 0.408569354 | 0.846 | 0.313 | 2.62E-14 | PST |
| LRRC4C | 1.35E-17 | 0.25173541 | 0.697 | 0.086 | 2.69E-14 | PST |
| ID1 | 1.66E-17 | 0.417633217 | 0.168 | 0.011 | 3.31E-14 | PST |
| SERPINA1 | 2.03E-17 | -0.416562533 | 0.7 | 0.082 | 4.06E-14 | PST |
| BAMBI | 2.39E-17 | -0.293856431 | 0.381 | 0.097 | 4.78E-14 | PST |
| AC069277.1 | 4.43E-17 | -0.266972043 | 0.728 | 0.119 | 8.85E-14 | PST |
| RCAN1 | 5.80E-17 | -0.26140009 | 0.858 | 0.278 | 1.16E-13 | PST |
| SORCS1 | 1.82E-16 | -0.344567622 | 1 | 0.411 | 3.64E-13 | PST |
| IGFBP3 | 2.15E-16 | 0.283859457 | 0.341 | 0.009 | 4.30E-13 | PST |
| NUAK2 | 2.38E-16 | -0.38473401 | 0.947 | 0.366 | 4.76E-13 | PST |
| CNTN5 | 6.86E-16 | 0.982560347 | 0.709 | 0.128 | 1.37E-12 | PST |
| KCNMB2 | 7.06E-16 | 1.24230975 | 0.769 | 0.28 | 1.41E-12 | PST |
| NRK | 9.45E-16 | 0.294831437 | 0.619 | 0.049 | 1.89E-12 | PST |
| AC087482.1 | 2.65E-15 | -0.310866752 | 0.849 | 0.272 | 5.29E-12 | PST |
| AC007906.2 | 7.31E-15 | 0.278546665 | 0.679 | 0.077 | 1.46E-11 | PST |
| PPFIA2 | 8.20E-15 | 0.261964871 | 0.671 | 0.062 | 1.64E-11 | PST |
| PDE1A | 8.69E-15 | -0.251379091 | 0.46 | 0.296 | 1.74E-11 | PST |
| ADGRB3 | 1.47E-14 | -0.256521114 | 0.779 | 0.205 | 2.94E-11 | PST |
| AC005394.2 | 1.58E-14 | -0.343200291 | 0.738 | 0.15 | 3.15E-11 | PST |
| TNC | 3.29E-14 | 0.380947311 | 0.673 | 0.075 | 6.58E-11 | PST |
| PHACTR1 | 4.96E-14 | 0.589390131 | 0.717 | 0.159 | 9.93E-11 | PST |
| SOX5 | 8.78E-14 | -0.447243112 | 0.987 | 0.404 | 1.76E-10 | PST |
| BCAS1 | 1.09E-13 | 0.388923993 | 0.173 | 0.004 | 2.18E-10 | PST |
| MIOX | 3.69E-13 | -0.668283681 | 0.982 | 0.4 | 7.39E-10 | PST |
| KCNIP1 | 3.82E-13 | -0.277790033 | 0.523 | 0.397 | 7.63E-10 | PST |
| MTUS2 | 5.59E-13 | 0.479761957 | 0.635 | 0.038 | 1.12E-09 | PST |
| SLC13A3 | 2.73E-12 | -0.406434542 | 0.903 | 0.358 | 5.47E-09 | PST |
| FREM2 | 4.17E-12 | 0.326456908 | 0.804 | 0.316 | 8.33E-09 | PST |
| SLC8A1-AS1 | 5.73E-12 | -0.294118685 | 0.457 | 0.19 | 1.15E-08 | PST |
| NPAS3 | 1.47E-11 | 0.257179878 | 0.909 | 0.437 | 2.93E-08 | PST |
| AP1S3 | 2.06E-11 | 0.256469965 | 0.426 | 0.146 | 4.12E-08 | PST |
| TMEM178B | 3.66E-11 | -0.38663556 | 0.728 | 0.715 | 7.32E-08 | PST |
| FOSB | 5.23E-11 | -0.332508763 | 0.643 | 0.071 | 1.05E-07 | PST |
| FSTL3 | 6.64E-11 | -0.642294917 | 0.966 | 0.411 | 1.33E-07 | PST |
| GPC3 | 8.32E-11 | 0.516386497 | 0.638 | 0.049 | 1.66E-07 | PST |
| PDE1C | 1.88E-10 | -0.265030798 | 0.451 | 0.148 | 3.75E-07 | PST |
| ACSM2B | 4.47E-10 | -0.291336186 | 1 | 0.956 | 8.94E-07 | PST |
| ST3GAL6 | 7.62E-10 | 0.26415554 | 0.773 | 0.287 | 1.52E-06 | PST |
| SHISA9 | 7.82E-10 | 0.567288818 | 0.714 | 0.201 | 1.56E-06 | PST |
| TSHZ2 | 5.60E-09 | 0.991160983 | 0.425 | 0.106 | 1.12E-05 | PST |
| MT1F | 7.21E-09 | -0.911667956 | 0.736 | 0.19 | 1.44E-05 | PST |
| FYB2 | 9.31E-09 | -0.281697432 | 0.58 | 0.435 | 1.86E-05 | PST |
| ZDHHC14 | 6.26E-08 | 0.298674673 | 0.775 | 0.333 | 0.000125179 | PST |
| CREB5 | 1.02E-07 | -0.357199976 | 0.654 | 0.642 | 0.000204336 | PST |
| ABI3BP | 1.65E-07 | 0.333341279 | 0.826 | 0.428 | 0.000329918 | PST |
| DCC | 1.76E-07 | -0.638986363 | 0.972 | 0.74 | 0.000352263 | PST |
| FIGNL1 | 1.94E-07 | -0.500624089 | 0.925 | 0.406 | 0.000387276 | PST |
| CYP4A22-AS1 | 4.10E-07 | -0.258833599 | 0.716 | 0.19 | 0.000820477 | PST |
| AC012593.1 | 4.66E-07 | 0.370725111 | 0.99 | 0.614 | 0.000931578 | PST |
| EYA1 | 9.19E-07 | 0.429231726 | 0.425 | 0.038 | 0.001837155 | PST |
| EPB41L2 | 1.03E-06 | -0.264124031 | 0.594 | 0.444 | 0.002054026 | PST |
| CADM1 | 1.05E-06 | 0.465437948 | 0.496 | 0.331 | 0.002104351 | PST |
| CCDC91 | 1.12E-06 | 0.251953288 | 0.918 | 0.594 | 0.002246582 | PST |
| IL32 | 1.19E-06 | -0.456782094 | 0.852 | 0.366 | 0.002385287 | PST |
| NRXN3 | 1.33E-06 | 0.483578349 | 0.785 | 0.395 | 0.002651069 | PST |
| AC004593.2 | 1.33E-06 | 0.281463857 | 0.794 | 0.415 | 0.002655115 | PST |
| TRPM7 | 1.41E-06 | -0.272853744 | 0.669 | 0.618 | 0.002811081 | PST |
| ARHGAP10 | 1.63E-06 | 0.305190079 | 0.994 | 0.629 | 0.003259367 | PST |
| SGIP1 | 3.03E-06 | 1.044939428 | 0.608 | 0.068 | 0.006058926 | PST |
| SLCO5A1 | 3.21E-06 | 0.591601239 | 0.924 | 0.625 | 0.00642567 | PST |
| SOD2 | 3.96E-06 | -0.4394066 | 0.706 | 0.592 | 0.007913679 | PST |
| ABCA13 | 3.96E-06 | 0.540187154 | 0.328 | 0.015 | 0.00791936 | PST |
| SDK1 | 4.27E-06 | -0.473575318 | 0.939 | 0.872 | 0.008544044 | PST |
| APBB1IP | 4.39E-06 | -0.267683866 | 0.635 | 0.483 | 0.008772286 | PST |
| LINC01060 | 5.06E-06 | -0.27165977 | 0.975 | 0.488 | 0.01012478 | PST |
| PPP1R14C | 5.28E-06 | 0.264955247 | 0.671 | 0.174 | 0.010556994 | PST |
| PLK2 | 7.27E-06 | -0.252079716 | 0.625 | 0.088 | 0.014549742 | PST |
| DCLK1 | 9.05E-06 | -0.251111258 | 0.786 | 0.316 | 0.018091163 | PST |
| TXNRD1 | 9.07E-06 | 0.276072095 | 0.745 | 0.296 | 0.018136295 | PST |
| FBXL7 | 9.13E-06 | 0.408925339 | 0.682 | 0.208 | 0.018251939 | PST |
| AL050403.2 | 1.25E-05 | 0.267090604 | 0.643 | 0.121 | 0.024967929 | PST |
| PHYHIPL | 1.68E-05 | 0.264417672 | 0.791 | 0.406 | 0.033556392 | PST |
| GABRA2 | 1.86E-05 | 0.393855716 | 0.404 | 0.022 | 0.037130836 | PST |
| MT1E | 6.66E-66 | -0.337975231 | 0.994 | 0.138 | 1.33E-62 | PEC |
| NETO1 | 7.50E-62 | -0.250265243 | 0.966 | 0.133 | 1.50E-58 | PEC |
| SOX4 | 4.61E-56 | 0.396586834 | 0.181 | 0.049 | 9.22E-53 | PEC |
| MIOX | 9.88E-49 | -0.307486261 | 0.911 | 0.119 | 1.98E-45 | PEC |
| GEM | 9.13E-47 | 0.276045668 | 0.212 | 0.051 | 1.83E-43 | PEC |
| KCNQ5 | 1.00E-46 | 0.345078065 | 0.215 | 0.114 | 2.01E-43 | PEC |
| BMP6 | 3.34E-46 | -0.400447289 | 0.908 | 0.125 | 6.68E-43 | PEC |
| ITGA11 | 6.16E-45 | 0.296084386 | 0.798 | 0.027 | 1.23E-41 | PEC |
| MT2A | 9.71E-45 | -0.617766401 | 0.997 | 0.209 | 1.94E-41 | PEC |
| ADCY8 | 5.31E-44 | 0.487394028 | 0.92 | 0.165 | 1.06E-40 | PEC |
| TP63 | 2.96E-43 | 0.499058877 | 0.942 | 0.184 | 5.91E-40 | PEC |
| RASD1 | 2.55E-41 | -0.356567101 | 0.991 | 0.217 | 5.10E-38 | PEC |
| LINC01811 | 1.34E-40 | -0.276990137 | 0.979 | 0.217 | 2.69E-37 | PEC |
| EYA4 | 4.28E-36 | -0.289738081 | 0.972 | 0.222 | 8.55E-33 | PEC |
| AC087633.2 | 6.02E-34 | 0.27685704 | 0.868 | 0.152 | 1.20E-30 | PEC |
| ZFPM2-AS1 | 2.88E-33 | 0.299542817 | 0.264 | 0.089 | 5.76E-30 | PEC |
| NUDT4 | 2.10E-30 | 0.27127286 | 0.994 | 0.322 | 4.19E-27 | PEC |
| SLC5A12 | 3.11E-30 | -0.283277445 | 0.908 | 0.195 | 6.22E-27 | PEC |
| AC011287.1 | 8.55E-30 | 0.272825289 | 0.288 | 0.111 | 1.71E-26 | PEC |
| HBEGF | 1.29E-29 | 0.340058802 | 0.255 | 0.054 | 2.58E-26 | PEC |
| MFSD2A | 5.72E-29 | 0.260096403 | 0.979 | 0.285 | 1.14E-25 | PEC |
| RELN | 2.70E-28 | 0.365759639 | 0.301 | 0.206 | 5.39E-25 | PEC |
| PRLR | 4.72E-28 | 0.273840276 | 0.985 | 0.295 | 9.45E-25 | PEC |
| AC079298.3 | 8.06E-28 | 0.586487238 | 0.291 | 0.149 | 1.61E-24 | PEC |
| AC019117.3 | 1.08E-26 | 0.269729987 | 0.298 | 0.103 | 2.17E-23 | PEC |
| AC008415.1 | 1.63E-26 | -0.893709609 | 0.914 | 0.217 | 3.26E-23 | PEC |
| KCNIP4 | 2.47E-25 | -0.366219833 | 0.34 | 0.241 | 4.93E-22 | PEC |
| SLC5A8 | 3.07E-24 | 0.39024796 | 0.301 | 0.038 | 6.13E-21 | PEC |
| SLC13A3 | 7.98E-23 | -0.301713977 | 0.819 | 0.144 | 1.60E-19 | PEC |
| GADD45B | 2.25E-22 | 0.428221437 | 1 | 0.366 | 4.50E-19 | PEC |
| UGDH | 6.03E-22 | 0.623681387 | 0.322 | 0.103 | 1.21E-18 | PEC |
| LINC01115 | 1.09E-20 | 0.405138937 | 0.313 | 0.033 | 2.17E-17 | PEC |
| BTG2 | 1.09E-20 | 0.614365421 | 0.822 | 0.187 | 2.17E-17 | PEC |
| TENM2 | 1.15E-20 | -0.307814271 | 0.963 | 0.304 | 2.30E-17 | PEC |
| CCN1 | 1.00E-19 | -0.542217312 | 0.896 | 0.244 | 2.00E-16 | PEC |
| ARMC4 | 5.26E-19 | 0.466582259 | 0.334 | 0.084 | 1.05E-15 | PEC |
| IGFBP4 | 5.74E-19 | 0.384914141 | 0.988 | 0.398 | 1.15E-15 | PEC |
| XKR4 | 2.37E-17 | 0.250317257 | 0.371 | 0.244 | 4.74E-14 | PEC |
| NTN4 | 4.29E-17 | -0.574495597 | 0.439 | 0.417 | 8.57E-14 | PEC |
| ATP1B3 | 8.66E-17 | 0.344296803 | 0.847 | 0.244 | 1.73E-13 | PEC |
| AC109466.1 | 1.57E-16 | -1.11752225 | 0.951 | 0.314 | 3.15E-13 | PEC |
| PDK4 | 5.36E-16 | -0.337457518 | 0.997 | 0.371 | 1.07E-12 | PEC |
| APBB1IP | 6.61E-16 | 0.36380651 | 0.359 | 0.16 | 1.32E-12 | PEC |
| KLRG2 | 7.79E-16 | -0.677606768 | 0.488 | 0.423 | 1.56E-12 | PEC |
| CLSTN2 | 8.66E-16 | -0.356926134 | 0.411 | 0.32 | 1.73E-12 | PEC |
| GRIN2A | 1.16E-15 | -0.339700904 | 0.791 | 0.168 | 2.32E-12 | PEC |
| PIEZO2 | 2.92E-15 | 0.288007068 | 0.353 | 0.054 | 5.83E-12 | PEC |
| SNTG2 | 4.29E-15 | 0.254805445 | 0.89 | 0.306 | 8.58E-12 | PEC |
| MICAL2 | 2.15E-14 | 0.336213018 | 0.975 | 0.423 | 4.30E-11 | PEC |
| C3 | 6.75E-14 | 0.377953363 | 0.365 | 0.07 | 1.35E-10 | PEC |
| UGT1A8 | 2.40E-13 | -0.325089495 | 0.791 | 0.184 | 4.80E-10 | PEC |
| FOXP2 | 1.14E-12 | 0.695803441 | 0.377 | 0.136 | 2.27E-09 | PEC |
| EIF2AK3 | 1.46E-12 | 0.596230991 | 0.865 | 0.341 | 2.93E-09 | PEC |
| ELF3 | 1.50E-12 | 0.373244531 | 0.393 | 0.157 | 3.00E-09 | PEC |
| FOSB | 2.82E-11 | -0.288940937 | 0.39 | 0.076 | 5.64E-08 | PEC |
| ACSM3 | 1.31E-10 | 0.388481982 | 0.433 | 0.328 | 2.62E-07 | PEC |
| INHBA | 1.39E-10 | 0.345113018 | 0.39 | 0.081 | 2.79E-07 | PEC |
| FLRT2 | 2.19E-10 | -0.281437147 | 0.782 | 0.209 | 4.37E-07 | PEC |
| ATP10A | 2.65E-10 | -0.4294099 | 1 | 0.407 | 5.30E-07 | PEC |
| KCNJ6 | 3.63E-10 | -0.292115407 | 0.742 | 0.163 | 7.25E-07 | PEC |
| KCTD8 | 4.76E-10 | -0.418468758 | 0.911 | 0.336 | 9.52E-07 | PEC |
| ZFP36 | 5.05E-10 | 0.298315208 | 1 | 0.512 | 1.01E-06 | PEC |
| PLOD2 | 5.35E-10 | 0.711733584 | 0.969 | 0.493 | 1.07E-06 | PEC |
| GLUL | 6.79E-10 | -0.427406584 | 0.957 | 0.388 | 1.36E-06 | PEC |
| TNC | 7.81E-10 | -0.28628006 | 0.966 | 0.407 | 1.56E-06 | PEC |
| SLC2A9 | 1.20E-09 | 0.344767111 | 0.871 | 0.401 | 2.40E-06 | PEC |
| HMGCS1 | 1.26E-09 | -0.327901015 | 0.917 | 0.366 | 2.52E-06 | PEC |
| CRISPLD2 | 1.31E-09 | 0.431166179 | 0.853 | 0.36 | 2.62E-06 | PEC |
| F5 | 1.51E-09 | -0.356255001 | 0.991 | 0.42 | 3.02E-06 | PEC |
| AP1S3 | 2.47E-09 | 0.257206595 | 0.439 | 0.249 | 4.94E-06 | PEC |
| RYR3 | 6.01E-09 | -0.453622196 | 0.969 | 0.401 | 1.20E-05 | PEC |
| MAMLD1 | 4.22E-08 | 0.346889569 | 0.758 | 0.236 | 8.44E-05 | PEC |
| RETREG1 | 4.53E-08 | 0.465781154 | 0.994 | 0.569 | 9.06E-05 | PEC |
| SLC12A1 | 5.16E-08 | -0.269411731 | 0.73 | 0.176 | 0.000103155 | PEC |
| PCDH15 | 6.24E-08 | -0.291433502 | 0.755 | 0.209 | 0.000124789 | PEC |
| TM4SF1 | 8.80E-08 | 0.362067828 | 0.666 | 0.098 | 0.00017599 | PEC |
| CRAT37 | 9.78E-08 | 0.306784258 | 0.393 | 0.065 | 0.000195667 | PEC |
| PTP4A1 | 1.34E-07 | 0.434880805 | 0.436 | 0.195 | 0.00026736 | PEC |
| SLC39A14 | 1.68E-07 | 0.476548509 | 0.966 | 0.528 | 0.00033572 | PEC |
| CXCL2 | 3.35E-07 | 0.583210576 | 0.353 | 0.043 | 0.000669119 | PEC |
| COL4A6 | 3.53E-07 | -0.325938138 | 0.537 | 0.352 | 0.000706081 | PEC |
| SVIL | 2.07E-06 | 0.334018618 | 0.969 | 0.615 | 0.004134496 | PEC |
| LINC01697 | 2.36E-06 | 0.318628391 | 0.742 | 0.257 | 0.004725436 | PEC |
| KHDRBS3 | 2.58E-06 | 0.318459183 | 0.755 | 0.257 | 0.00516116 | PEC |
| LSAMP | 5.29E-06 | 0.46461139 | 0.69 | 0.171 | 0.010580677 | PEC |
| EPS8 | 6.45E-06 | 0.414815437 | 0.81 | 0.439 | 0.012891559 | PEC |
| SYNE1 | 9.00E-06 | -0.365737453 | 0.988 | 0.957 | 0.018001064 | PEC |
| GPAT3 | 9.56E-06 | -0.280141629 | 0.574 | 0.472 | 0.019116215 | PEC |
| MGP | 1.21E-05 | -0.265775992 | 0.451 | 0.111 | 0.02422621 | PEC |
| TNIK | 1.30E-05 | 0.368183516 | 0.883 | 0.623 | 0.026007767 | PEC |
| GRIN2B | 7.76E-67 | 0.307985176 | 0.126 | 0.05 | 1.55E-63 | PODO |
| SLC12A3 | 9.38E-59 | -0.332900824 | 0.964 | 0.118 | 1.88E-55 | PODO |
| SLC6A13 | 7.89E-49 | -0.299873674 | 0.946 | 0.132 | 1.58E-45 | PODO |
| AFM | 2.25E-45 | -0.333587738 | 0.866 | 0.103 | 4.49E-42 | PODO |
| FGF14 | 1.96E-41 | 0.266476784 | 0.96 | 0.188 | 3.93E-38 | PODO |
| ACSM2B | 6.12E-33 | -0.840435006 | 1 | 0.244 | 1.22E-29 | PODO |
| SLIT2 | 2.34E-27 | -0.283696549 | 0.975 | 0.262 | 4.69E-24 | PODO |
| CYP4Z2P | 1.26E-26 | -0.306692223 | 0.953 | 0.238 | 2.52E-23 | PODO |
| LINGO2 | 1.41E-26 | -0.326430536 | 1 | 0.279 | 2.83E-23 | PODO |
| TEX41 | 2.74E-25 | -0.306938417 | 0.31 | 0.162 | 5.48E-22 | PODO |
| KCNIP1 | 8.23E-22 | -0.353534899 | 0.996 | 0.297 | 1.65E-18 | PODO |
| MIOX | 6.73E-18 | -0.371935769 | 0.354 | 0.141 | 1.35E-14 | PODO |
| NALCN | 1.02E-16 | 0.351099025 | 0.996 | 0.4 | 2.05E-13 | PODO |
| AC018742.1 | 3.48E-16 | 0.38795162 | 0.996 | 0.424 | 6.97E-13 | PODO |
| ACTB | 9.42E-16 | 0.641495988 | 0.968 | 0.465 | 1.88E-12 | PODO |
| UNC5D | 2.03E-15 | 0.487873157 | 0.35 | 0.124 | 4.05E-12 | PODO |
| AC096577.1 | 7.82E-14 | -0.270726787 | 0.744 | 0.112 | 1.56E-10 | PODO |
| DCN | 1.43E-13 | -0.339033301 | 0.455 | 0.435 | 2.85E-10 | PODO |
| PMEPA1 | 1.70E-12 | -0.295209593 | 0.458 | 0.365 | 3.41E-09 | PODO |
| PLCB1 | 1.55E-11 | 0.335091474 | 0.404 | 0.206 | 3.10E-08 | PODO |
| ATP6V0D2 | 6.85E-11 | -0.282991811 | 0.762 | 0.162 | 1.37E-07 | PODO |
| LRP2 | 7.67E-10 | -0.328332836 | 0.462 | 0.297 | 1.53E-06 | PODO |
| VIM | 1.45E-09 | 0.401068098 | 0.856 | 0.388 | 2.90E-06 | PODO |
| NELL1 | 4.42E-09 | 0.345402002 | 0.942 | 0.432 | 8.83E-06 | PODO |
| F3 | 1.57E-08 | 0.352333683 | 1 | 0.524 | 3.15E-05 | PODO |
| SLIT3 | 1.66E-07 | 0.276381331 | 0.783 | 0.268 | 0.000331981 | PODO |
| TMEM178A | 1.09E-06 | 0.30691574 | 1 | 0.6 | 0.002187426 | PODO |
| ST6GAL1 | 3.29E-06 | -0.290354025 | 0.563 | 0.456 | 0.006585287 | PODO |
| EXPH5 | 2.30E-05 | 0.449940058 | 0.996 | 0.882 | 0.046091461 | PODO |
| LRP2 | 1.22E-45 | -0.283488262 | 0.183 | 0.141 | 2.44E-42 | CD-ICB |
| SORCS1 | 1.12E-40 | -0.430420333 | 0.993 | 0.185 | 2.24E-37 | CD-ICB |
| AC013652.1 | 1.81E-32 | 0.27689616 | 0.232 | 0.107 | 3.63E-29 | CD-ICB |
| HSPA5 | 6.18E-32 | 0.390880044 | 0.232 | 0.085 | 1.24E-28 | CD-ICB |
| CYTOR | 5.69E-31 | 0.516562465 | 0.236 | 0.074 | 1.14E-27 | CD-ICB |
| FKBP5 | 6.42E-29 | -0.350613014 | 1 | 0.248 | 1.28E-25 | CD-ICB |
| ATP1B3 | 7.10E-27 | 0.329012516 | 0.94 | 0.237 | 1.42E-23 | CD-ICB |
| NRG1 | 1.08E-26 | -0.310667624 | 0.275 | 0.104 | 2.16E-23 | CD-ICB |
| PCDH15 | 4.30E-25 | -0.266433672 | 0.289 | 0.152 | 8.60E-22 | CD-ICB |
| KCNQ3 | 4.97E-24 | -0.329180366 | 1 | 0.293 | 9.95E-21 | CD-ICB |
| SOX4 | 4.66E-23 | 0.289352427 | 0.278 | 0.041 | 9.31E-20 | CD-ICB |
| ALDH1A2 | 1.50E-22 | -0.293071878 | 0.303 | 0.13 | 3.00E-19 | CD-ICB |
| OSBPL6 | 2.53E-22 | -0.432878873 | 0.901 | 0.196 | 5.05E-19 | CD-ICB |
| DDIT3 | 3.44E-22 | 0.256209258 | 0.289 | 0.107 | 6.89E-19 | CD-ICB |
| TMEM101 | 4.73E-20 | -0.52396183 | 0.996 | 0.293 | 9.46E-17 | CD-ICB |
| AC074286.1 | 1.91E-19 | 0.294657908 | 0.31 | 0.126 | 3.82E-16 | CD-ICB |
| ADAMTS18 | 3.36E-19 | 0.29329733 | 0.993 | 0.341 | 6.71E-16 | CD-ICB |
| HPSE | 1.69E-18 | -0.379999602 | 0.887 | 0.215 | 3.39E-15 | CD-ICB |
| MIR4435-2HG | 2.90E-16 | 0.39738698 | 0.338 | 0.2 | 5.80E-13 | CD-ICB |
| S100A2 | 7.47E-15 | 0.595324047 | 0.335 | 0.078 | 1.49E-11 | CD-ICB |
| SLC12A3 | 9.94E-14 | -0.310170981 | 0.373 | 0.174 | 1.99E-10 | CD-ICB |
| GDF15 | 1.98E-11 | 0.306471121 | 0.366 | 0.085 | 3.95E-08 | CD-ICB |
| GPC3 | 6.85E-11 | -0.375174569 | 0.82 | 0.215 | 1.37E-07 | CD-ICB |
| MT-ND6 | 1.22E-10 | -0.295253514 | 0.915 | 0.315 | 2.44E-07 | CD-ICB |
| PDE10A | 3.51E-10 | -0.305923479 | 0.451 | 0.352 | 7.01E-07 | CD-ICB |
| ITPRID1 | 3.77E-10 | 0.311389287 | 0.884 | 0.352 | 7.55E-07 | CD-ICB |
| SLC8A1 | 1.15E-08 | -0.535665503 | 0.475 | 0.367 | 2.29E-05 | CD-ICB |
| DHFR | 2.45E-08 | 0.415900416 | 1 | 0.478 | 4.90E-05 | CD-ICB |
| ERRFI1 | 2.55E-08 | -0.365281496 | 0.993 | 0.426 | 5.10E-05 | CD-ICB |
| FAM102A | 4.23E-08 | 0.35057552 | 0.778 | 0.248 | 8.46E-05 | CD-ICB |
| SLIT2 | 7.24E-08 | -0.309805747 | 0.447 | 0.207 | 0.000144869 | CD-ICB |
| KCNMB2 | 1.12E-07 | -0.418246194 | 0.901 | 0.344 | 0.000223111 | CD-ICB |
| EGF | 1.17E-07 | -0.47879484 | 0.715 | 0.137 | 0.000234451 | CD-ICB |
| UNC13C | 1.35E-07 | -0.306098693 | 0.866 | 0.33 | 0.000270757 | CD-ICB |
| ADARB2 | 3.51E-07 | 0.538406201 | 0.81 | 0.293 | 0.000701011 | CD-ICB |
| AC117386.2 | 5.20E-07 | 0.359076838 | 0.761 | 0.237 | 0.001039171 | CD-ICB |
| GPC6 | 2.52E-06 | -0.344164601 | 0.859 | 0.352 | 0.005040003 | CD-ICB |
| MIR100HG | 4.46E-06 | 0.300914802 | 1 | 0.578 | 0.008925351 | CD-ICB |
| SHOC1 | 8.19E-06 | -0.255070979 | 0.521 | 0.381 | 0.016381779 | CD-ICB |
| MEST | 8.27E-06 | 0.279200024 | 0.412 | 0.048 | 0.016534492 | CD-ICB |
| KCNIP4 | 2.10E-05 | 0.49879739 | 0.454 | 0.248 | 0.042006167 | CD-ICB |
| DPYS | 8.26E-19 | 0.365455732 | 1 | 0 | 1.65E-15 | LEUK |
| SLC47A2 | 1.48E-18 | 0.381733299 | 0.996 | 0 | 2.95E-15 | LEUK |
| MAPT | 1.48E-18 | 0.347720968 | 0.996 | 0 | 2.95E-15 | LEUK |
| ALDH1A1 | 4.65E-18 | 0.272931123 | 0.989 | 0 | 9.29E-15 | LEUK |
| ALDOB | 6.15E-18 | 0.485691545 | 0.985 | 0 | 1.23E-14 | LEUK |
| CD200 | 1.44E-17 | 0.291315203 | 0.982 | 0 | 2.88E-14 | LEUK |
| SLC14A2 | 1.34E-16 | 0.364931726 | 1 | 0.034 | 2.68E-13 | LEUK |
| LBH | 1.39E-16 | 0.385316523 | 1 | 0.034 | 2.79E-13 | LEUK |
| SLC7A8 | 2.63E-16 | 0.326136181 | 0.996 | 0.034 | 5.26E-13 | LEUK |
| ESRRB | 2.78E-16 | -0.279442435 | 0.996 | 0.034 | 5.56E-13 | LEUK |
| COLEC12 | 3.44E-16 | -0.264686383 | 0.989 | 0.034 | 6.88E-13 | LEUK |
| Z93403.1 | 4.26E-16 | 0.518398374 | 0.993 | 0.034 | 8.51E-13 | LEUK |
| AC024901.1 | 4.67E-16 | -0.261342217 | 0.993 | 0.034 | 9.33E-13 | LEUK |
| FYB2 | 9.90E-16 | 0.435916314 | 0.985 | 0.034 | 1.98E-12 | LEUK |
| ALDH1A2 | 5.61E-15 | -0.816690282 | 0.066 | 0.138 | 1.12E-11 | LEUK |
| SLC7A2 | 5.87E-15 | -0.276156408 | 0.974 | 0.034 | 1.17E-11 | LEUK |
| AC087482.1 | 5.87E-15 | -0.285359475 | 0.974 | 0.034 | 1.17E-11 | LEUK |
| PTPRO | 1.15E-14 | 0.395106808 | 0.934 | 0 | 2.31E-11 | LEUK |
| MIR3142HG | 1.63E-14 | 0.260291558 | 0.93 | 0.034 | 3.26E-11 | LEUK |
| CNTN1 | 2.20E-14 | -0.425217941 | 1 | 0.069 | 4.40E-11 | LEUK |
| MYH11 | 3.34E-14 | -0.309016665 | 0.077 | 0.103 | 6.68E-11 | LEUK |
| C1QTNF7-AS1 | 3.89E-14 | 0.423091006 | 0.993 | 0.069 | 7.78E-11 | LEUK |
| PAPPA | 1.37E-13 | 0.334794659 | 0.949 | 0.034 | 2.74E-10 | LEUK |
| AKR1C1 | 1.56E-13 | -0.465757697 | 0.908 | 0.034 | 3.12E-10 | LEUK |
| PRODH2 | 1.67E-13 | 0.367687594 | 0.949 | 0.034 | 3.35E-10 | LEUK |
| AC018742.1 | 2.30E-13 | 0.502651232 | 0.945 | 0.034 | 4.60E-10 | LEUK |
| GAD1 | 3.44E-13 | -0.402656902 | 0.978 | 0.069 | 6.88E-10 | LEUK |
| FRMD5 | 5.20E-13 | -0.315204778 | 0.974 | 0.069 | 1.04E-09 | LEUK |
| TNFRSF19 | 5.55E-13 | -0.329765806 | 0.974 | 0.069 | 1.11E-09 | LEUK |
| AC078923.1 | 8.35E-13 | -0.412049064 | 0.971 | 0.069 | 1.67E-09 | LEUK |
| OTOGL | 1.10E-12 | -0.296882244 | 0.934 | 0.034 | 2.20E-09 | LEUK |
| F5 | 1.25E-12 | -0.52829379 | 0.934 | 0.069 | 2.50E-09 | LEUK |
| PTCHD1-AS | 1.93E-12 | 0.837480588 | 0.103 | 0 | 3.86E-09 | LEUK |
| LRRC9 | 1.93E-12 | 0.3206925 | 0.103 | 0 | 3.86E-09 | LEUK |
| ANKRD36BP2 | 1.93E-12 | 1.151142141 | 0.103 | 0 | 3.86E-09 | LEUK |
| TMEM178A | 2.07E-12 | -0.29843026 | 1 | 0.103 | 4.14E-09 | LEUK |
| LINC01505 | 2.76E-12 | -0.43421606 | 0.996 | 0.103 | 5.52E-09 | LEUK |
| DGKB | 2.95E-12 | -0.682914648 | 0.121 | 0.138 | 5.90E-09 | LEUK |
| SNTG1 | 3.07E-12 | 0.42725468 | 0.894 | 0 | 6.14E-09 | LEUK |
| CACNB2 | 3.07E-12 | 0.504935009 | 0.106 | 0 | 6.14E-09 | LEUK |
| NLGN4Y | 3.07E-12 | 0.492079961 | 0.106 | 0 | 6.14E-09 | LEUK |
| HNF4A | 3.09E-12 | -0.606148338 | 0.996 | 0.103 | 6.17E-09 | LEUK |
| LINGO2 | 3.56E-12 | -0.689498108 | 0.993 | 0.103 | 7.12E-09 | LEUK |
| DACH1 | 4.38E-12 | 0.456500053 | 0.982 | 0.103 | 8.75E-09 | LEUK |
| AC003984.1 | 4.95E-12 | -0.748396756 | 0.989 | 0.103 | 9.89E-09 | LEUK |
| ALPK2 | 6.00E-12 | -0.358696403 | 0.989 | 0.103 | 1.20E-08 | LEUK |
| SPP1 | 6.19E-12 | -0.274532508 | 0.989 | 0.103 | 1.24E-08 | LEUK |
| ABCC3 | 6.28E-12 | -0.271807118 | 0.989 | 0.103 | 1.26E-08 | LEUK |
| KIAA1191 | 7.00E-12 | -0.448508359 | 0.952 | 0.069 | 1.40E-08 | LEUK |
| POU2AF1 | 7.66E-12 | 0.783432591 | 0.114 | 0 | 1.53E-08 | LEUK |
| LINC00621 | 9.11E-12 | 0.293316227 | 0.916 | 0.034 | 1.82E-08 | LEUK |
| HSPA1A | 1.16E-11 | -0.337902248 | 0.128 | 0.138 | 2.31E-08 | LEUK |
| CLEC7A | 1.28E-11 | -0.640145864 | 0.07 | 0.138 | 2.55E-08 | LEUK |
| ITGA2 | 1.35E-11 | -0.283394127 | 0.982 | 0.103 | 2.70E-08 | LEUK |
| ABCA10 | 1.69E-11 | 0.374554328 | 0.121 | 0.034 | 3.38E-08 | LEUK |
| ADAMTSL1 | 1.88E-11 | 0.412919166 | 0.121 | 0 | 3.76E-08 | LEUK |
| LONRF3 | 1.90E-11 | -0.932594403 | 0.971 | 0.103 | 3.80E-08 | LEUK |
| MIR222HG | 2.16E-11 | 0.313935781 | 0.125 | 0.034 | 4.33E-08 | LEUK |
| BBOX1 | 3.36E-11 | 0.50186422 | 0.905 | 0.034 | 6.73E-08 | LEUK |
| SLC35F1 | 3.59E-11 | -0.260173023 | 0.96 | 0.103 | 7.17E-08 | LEUK |
| CCL4 | 4.09E-11 | -1.774668834 | 0.949 | 0.103 | 8.19E-08 | LEUK |
| HMOX1 | 5.70E-11 | -0.428580506 | 0.938 | 0.103 | 1.14E-07 | LEUK |
| CYP3A5 | 6.36E-11 | 0.748944553 | 0.989 | 0.138 | 1.27E-07 | LEUK |
| PRAG1 | 7.01E-11 | 0.394619738 | 0.132 | 0 | 1.40E-07 | LEUK |
| TINAG | 7.60E-11 | -0.572773689 | 0.967 | 0.103 | 1.52E-07 | LEUK |
| SCN1A-AS1 | 8.67E-11 | -0.255540439 | 0.93 | 0.069 | 1.73E-07 | LEUK |
| SRGAP1 | 8.70E-11 | -0.552293888 | 1 | 0.138 | 1.74E-07 | LEUK |
| ST3GAL6 | 9.19E-11 | 0.313257979 | 0.136 | 0.034 | 1.84E-07 | LEUK |
| SPRY1 | 1.03E-10 | -0.28628957 | 0.927 | 0.069 | 2.05E-07 | LEUK |
| NOX4 | 1.12E-10 | -0.410445433 | 1 | 0.138 | 2.24E-07 | LEUK |
| SORCS1 | 1.20E-10 | -0.277590168 | 0.996 | 0.138 | 2.41E-07 | LEUK |
| RNF212B | 1.38E-10 | -0.36274735 | 0.996 | 0.138 | 2.75E-07 | LEUK |
| EGR1 | 1.52E-10 | -0.485601578 | 0.96 | 0.103 | 3.04E-07 | LEUK |
| AL110292.1 | 1.65E-10 | 0.289830388 | 0.861 | 0 | 3.31E-07 | LEUK |
| COL23A1 | 1.74E-10 | -0.558218655 | 0.996 | 0.138 | 3.48E-07 | LEUK |
| MEIS1 | 2.04E-10 | 0.318712713 | 0.857 | 0 | 4.08E-07 | LEUK |
| PLCXD3 | 2.27E-10 | -0.358389412 | 0.993 | 0.138 | 4.55E-07 | LEUK |
| GATA3 | 2.52E-10 | 0.298537927 | 0.143 | 0 | 5.04E-07 | LEUK |
| AC093895.1 | 2.76E-10 | -0.343026652 | 0.839 | 0.069 | 5.51E-07 | LEUK |
| ITPKC | 2.89E-10 | -0.40335467 | 0.993 | 0.138 | 5.78E-07 | LEUK |
| AC244205.1 | 3.10E-10 | 1.207117337 | 0.143 | 0 | 6.21E-07 | LEUK |
| LINC01503 | 3.45E-10 | -0.354144736 | 0.861 | 0.034 | 6.90E-07 | LEUK |
| SMIM35 | 3.69E-10 | -0.591770933 | 0.952 | 0.103 | 7.37E-07 | LEUK |
| FCRL5 | 3.78E-10 | 1.004379322 | 0.136 | 0 | 7.56E-07 | LEUK |
| SLC20A1 | 3.92E-10 | -0.672117994 | 0.179 | 0.241 | 7.85E-07 | LEUK |
| SOX5 | 4.14E-10 | 0.778562776 | 0.938 | 0.103 | 8.27E-07 | LEUK |
| RGS1 | 4.26E-10 | -0.428238514 | 0.161 | 0.103 | 8.51E-07 | LEUK |
| CDH13 | 4.82E-10 | -0.26214574 | 0.154 | 0.034 | 9.65E-07 | LEUK |
| AC104461.1 | 7.04E-10 | -0.3915564 | 0.824 | 0.069 | 1.41E-06 | LEUK |
| PWRN1 | 8.73E-10 | 0.368023668 | 0.154 | 0 | 1.75E-06 | LEUK |
| MSC-AS1 | 1.05E-09 | 0.286688469 | 0.143 | 0 | 2.11E-06 | LEUK |
| LINC01098 | 1.12E-09 | -0.410846796 | 0.179 | 0.172 | 2.23E-06 | LEUK |
| ATF3 | 1.19E-09 | -0.482080917 | 0.172 | 0.103 | 2.39E-06 | LEUK |
| EIF2AK3 | 1.26E-09 | -0.260986733 | 0.172 | 0.103 | 2.53E-06 | LEUK |
| FTL | 1.28E-09 | 0.580174569 | 1 | 0.172 | 2.57E-06 | LEUK |
| MYO1B | 1.84E-09 | -0.386736801 | 0.934 | 0.103 | 3.67E-06 | LEUK |
| SSPN | 1.93E-09 | 0.469663201 | 0.15 | 0 | 3.87E-06 | LEUK |
| AC011287.1 | 1.94E-09 | -0.542053183 | 0.934 | 0.103 | 3.88E-06 | LEUK |
| GNAI1 | 2.10E-09 | -0.250813982 | 0.868 | 0.034 | 4.20E-06 | LEUK |
| CHODL | 2.15E-09 | 0.418193815 | 0.161 | 0.034 | 4.30E-06 | LEUK |
| AC096577.1 | 2.42E-09 | -0.315176683 | 1 | 0.172 | 4.85E-06 | LEUK |
| EPS8 | 2.66E-09 | -0.304211384 | 0.183 | 0.138 | 5.33E-06 | LEUK |
| LINC02196 | 2.73E-09 | -0.451202983 | 0.93 | 0.103 | 5.47E-06 | LEUK |
| PDK4 | 3.01E-09 | -0.81127975 | 0.93 | 0.103 | 6.02E-06 | LEUK |
| CYP4A22 | 3.05E-09 | -0.342920706 | 0.714 | 0.034 | 6.09E-06 | LEUK |
| DEPTOR | 3.46E-09 | -0.366644225 | 1 | 0.172 | 6.91E-06 | LEUK |
| NPL | 3.60E-09 | -0.257361986 | 1 | 0.172 | 7.20E-06 | LEUK |
| LINC00189 | 3.79E-09 | -0.756625102 | 0.853 | 0.103 | 7.59E-06 | LEUK |
| SLC30A8 | 3.84E-09 | -0.311770244 | 0.894 | 0.069 | 7.67E-06 | LEUK |
| LINC00871 | 3.90E-09 | -0.390245079 | 1 | 0.172 | 7.81E-06 | LEUK |
| DUSP5 | 4.27E-09 | 0.610917688 | 0.158 | 0 | 8.54E-06 | LEUK |
| SHISA9 | 4.32E-09 | 0.296630508 | 0.168 | 0 | 8.64E-06 | LEUK |
| CADPS2 | 4.59E-09 | 0.86372686 | 0.941 | 0.138 | 9.17E-06 | LEUK |
| SLC17A1 | 4.78E-09 | -0.551234136 | 1 | 0.172 | 9.56E-06 | LEUK |
| ADGRB3 | 4.89E-09 | -0.265926001 | 0.179 | 0.069 | 9.78E-06 | LEUK |
| CTTNBP2 | 5.11E-09 | -0.558592439 | 1 | 0.172 | 1.02E-05 | LEUK |
| EMP1 | 5.27E-09 | -0.398951302 | 0.799 | 0.069 | 1.05E-05 | LEUK |
| MIOX | 5.39E-09 | -1.234796088 | 1 | 0.172 | 1.08E-05 | LEUK |
| AC113414.1 | 5.90E-09 | 0.261229577 | 0.886 | 0.069 | 1.18E-05 | LEUK |
| H2AC18 | 6.49E-09 | 0.648808333 | 0.183 | 0.103 | 1.30E-05 | LEUK |
| AGXT2 | 6.95E-09 | -0.675928914 | 0.996 | 0.172 | 1.39E-05 | LEUK |
| PLPP1 | 7.13E-09 | 0.307205647 | 0.945 | 0.138 | 1.43E-05 | LEUK |
| LINC02432 | 8.14E-09 | -0.341691593 | 0.919 | 0.103 | 1.63E-05 | LEUK |
| PMEPA1 | 8.22E-09 | 0.302540263 | 0.179 | 0.034 | 1.64E-05 | LEUK |
| PPFIBP1 | 8.91E-09 | 0.408461998 | 0.179 | 0.034 | 1.78E-05 | LEUK |
| SQSTM1 | 9.17E-09 | -0.28047968 | 0.194 | 0.103 | 1.83E-05 | LEUK |
| TRAF1 | 9.34E-09 | 0.780539771 | 0.172 | 0 | 1.87E-05 | LEUK |
| NLRP3 | 1.06E-08 | -0.732832777 | 0.934 | 0.138 | 2.12E-05 | LEUK |
| SLC4A11 | 1.09E-08 | -0.744316109 | 0.696 | 0.034 | 2.18E-05 | LEUK |
| ADGRF5 | 1.11E-08 | 0.364725346 | 0.183 | 0.034 | 2.23E-05 | LEUK |
| LINC00540 | 1.16E-08 | -0.260717737 | 0.19 | 0.069 | 2.32E-05 | LEUK |
| SLC26A7 | 1.17E-08 | -0.424431725 | 0.209 | 0.207 | 2.33E-05 | LEUK |
| LIPA | 1.25E-08 | 0.250965386 | 0.183 | 0.034 | 2.51E-05 | LEUK |
| NEDD4 | 1.37E-08 | 0.300110655 | 0.179 | 0 | 2.75E-05 | LEUK |
| H3-3B | 1.55E-08 | 0.315507076 | 0.19 | 0.069 | 3.10E-05 | LEUK |
| TNFRSF21 | 1.55E-08 | -0.737887022 | 0.985 | 0.172 | 3.11E-05 | LEUK |
| SCNN1B | 1.65E-08 | 0.254763091 | 0.813 | 0 | 3.30E-05 | LEUK |
| HAVCR2 | 1.88E-08 | -0.349929623 | 0.194 | 0.069 | 3.76E-05 | LEUK |
| SLC2A3 | 2.01E-08 | -0.399469079 | 0.212 | 0.172 | 4.03E-05 | LEUK |
| LINC02694 | 2.25E-08 | 0.33979538 | 0.194 | 0.069 | 4.51E-05 | LEUK |
| EGF | 2.31E-08 | -0.289080437 | 0.875 | 0.069 | 4.63E-05 | LEUK |
| ANXA1 | 2.32E-08 | -1.051118313 | 0.176 | 0.069 | 4.64E-05 | LEUK |
| CCN1 | 2.34E-08 | -0.855519787 | 0.733 | 0.034 | 4.68E-05 | LEUK |
| ERO1A | 2.91E-08 | 0.40378547 | 0.187 | 0 | 5.81E-05 | LEUK |
| SIGLEC1 | 3.37E-08 | -1.130673763 | 0.817 | 0.103 | 6.74E-05 | LEUK |
| SLC12A1 | 4.22E-08 | -0.358193739 | 0.934 | 0.138 | 8.45E-05 | LEUK |
| SPSB1 | 4.44E-08 | -0.612545703 | 0.212 | 0.103 | 8.88E-05 | LEUK |
| CPNE4 | 4.55E-08 | -0.524686402 | 0.901 | 0.103 | 9.10E-05 | LEUK |
| FCRL1 | 5.04E-08 | 0.965398355 | 0.19 | 0 | 0.000100787 | LEUK |
| KLF4 | 5.10E-08 | -0.289825335 | 0.799 | 0.103 | 0.000102 | LEUK |
| LY9 | 5.12E-08 | 0.347388793 | 0.15 | 0.034 | 0.000102485 | LEUK |
| NPHS1 | 5.33E-08 | -0.460819219 | 0.729 | 0.069 | 0.000106613 | LEUK |
| ENPEP | 6.08E-08 | -0.375907728 | 0.93 | 0.138 | 0.000121655 | LEUK |
| ACSM2A | 7.26E-08 | -0.391899315 | 1 | 0.207 | 0.000145138 | LEUK |
| FOS | 7.92E-08 | -1.410365641 | 0.242 | 0.207 | 0.000158321 | LEUK |
| CLEC2B | 8.29E-08 | -0.480636215 | 0.216 | 0.103 | 0.000165883 | LEUK |
| LRP2 | 8.42E-08 | -0.782672379 | 1 | 0.207 | 0.000168439 | LEUK |
| BCL11A | 8.68E-08 | 0.521842681 | 0.198 | 0 | 0.000173665 | LEUK |
| LINC02798 | 8.68E-08 | -0.637605541 | 0.718 | 0.069 | 0.000173685 | LEUK |
| BTBD11 | 9.28E-08 | 0.34711795 | 0.916 | 0.138 | 0.000185601 | LEUK |
| LEF1 | 1.02E-07 | 0.359938392 | 0.912 | 0.138 | 0.000204388 | LEUK |
| ZDHHC14 | 1.28E-07 | -0.481894538 | 0.231 | 0.172 | 0.00025564 | LEUK |
| CPM | 1.29E-07 | -0.695369663 | 0.923 | 0.138 | 0.000258538 | LEUK |
| NETO2 | 1.33E-07 | -0.475315188 | 0.773 | 0.103 | 0.000265928 | LEUK |
| ROBO1 | 1.38E-07 | 0.57157224 | 0.114 | 0 | 0.000276462 | LEUK |
| AKAP12 | 1.40E-07 | 0.265470076 | 0.824 | 0.034 | 0.000280648 | LEUK |
| DEPP1 | 1.43E-07 | -0.318918538 | 0.656 | 0.034 | 0.00028515 | LEUK |
| HGF | 1.45E-07 | -0.368642912 | 0.824 | 0.069 | 0.000290375 | LEUK |
| PLAUR | 1.48E-07 | -0.721375169 | 0.993 | 0.207 | 0.000296145 | LEUK |
| LINC00298 | 1.57E-07 | -0.622326728 | 0.85 | 0.138 | 0.000314124 | LEUK |
| TIPARP | 1.59E-07 | -0.663918119 | 1 | 0.207 | 0.000318523 | LEUK |
| AFM | 1.91E-07 | -0.693889568 | 0.996 | 0.207 | 0.000381854 | LEUK |
| FMN1 | 2.08E-07 | -0.328377433 | 0.242 | 0.207 | 0.000415421 | LEUK |
| SLC35F4 | 2.09E-07 | -0.442740408 | 0.879 | 0.103 | 0.000418366 | LEUK |
| CACNA2D3 | 2.26E-07 | -0.900841577 | 0.245 | 0.172 | 0.000451454 | LEUK |
| HSPA5 | 2.74E-07 | -0.57437609 | 0.238 | 0.138 | 0.000548792 | LEUK |
| LINC01320 | 3.33E-07 | -0.38802546 | 0.227 | 0.069 | 0.000666808 | LEUK |
| ACSL4 | 4.09E-07 | -0.25687766 | 0.253 | 0.207 | 0.000817715 | LEUK |
| DGKG | 4.34E-07 | 0.266299054 | 0.147 | 0 | 0.000868447 | LEUK |
| ZBTB7C | 4.40E-07 | -0.403336938 | 0.718 | 0.069 | 0.000880252 | LEUK |
| PFKFB3 | 4.55E-07 | -0.484662187 | 0.271 | 0.31 | 0.000910505 | LEUK |
| H2AC6 | 4.63E-07 | 0.652164465 | 0.22 | 0.034 | 0.000925852 | LEUK |
| HLA-DPB1 | 4.64E-07 | -0.35420717 | 0.227 | 0.138 | 0.000927409 | LEUK |
| COL21A1 | 5.41E-07 | -0.305598113 | 0.608 | 0.034 | 0.001081311 | LEUK |
| SGO1-AS1 | 6.42E-07 | 0.527638127 | 0.227 | 0.069 | 0.001283439 | LEUK |
| VIM | 6.42E-07 | -0.277829929 | 0.242 | 0.103 | 0.001284702 | LEUK |
| ABCC4 | 6.58E-07 | 0.286308744 | 0.238 | 0.138 | 0.001315924 | LEUK |
| BLK | 6.94E-07 | 1.118059963 | 0.22 | 0 | 0.001388383 | LEUK |
| COL19A1 | 6.94E-07 | 0.697718446 | 0.22 | 0 | 0.001388383 | LEUK |
| HLA-DRB5 | 8.31E-07 | -0.605445185 | 0.286 | 0.345 | 0.001662794 | LEUK |
| DCC | 9.12E-07 | 0.649317043 | 0.799 | 0.034 | 0.001823748 | LEUK |
| DLGAP1 | 9.60E-07 | 0.352003227 | 0.861 | 0.103 | 0.00191944 | LEUK |
| KSR2 | 9.68E-07 | 0.362803236 | 0.223 | 0 | 0.001935441 | LEUK |
| ACSM2B | 1.02E-06 | -0.583568276 | 1 | 0.241 | 0.002038124 | LEUK |
| AC109466.1 | 1.15E-06 | -0.37570529 | 0.832 | 0.069 | 0.002299439 | LEUK |
| NFKBIZ | 1.22E-06 | -0.441657426 | 0.26 | 0.172 | 0.00243942 | LEUK |
| TGFBR3 | 1.25E-06 | -0.299702678 | 1 | 0.241 | 0.002498591 | LEUK |
| EBF1 | 1.30E-06 | 1.169777969 | 0.227 | 0.034 | 0.002600709 | LEUK |
| CALD1 | 1.41E-06 | 0.461051135 | 0.799 | 0.034 | 0.002813622 | LEUK |
| DTNA | 1.78E-06 | -0.339486629 | 0.886 | 0.138 | 0.00356743 | LEUK |
| KCNJ15 | 1.83E-06 | -0.342437951 | 1 | 0.241 | 0.003655443 | LEUK |
| ACSF2 | 1.98E-06 | -0.314396364 | 0.996 | 0.241 | 0.003950573 | LEUK |
| UGCG | 2.12E-06 | 0.424111308 | 0.238 | 0.034 | 0.004248072 | LEUK |
| AL590385.2 | 2.77E-06 | -0.695207518 | 0.3 | 0.345 | 0.005535248 | LEUK |
| CD163 | 3.17E-06 | -1.558546244 | 1 | 0.241 | 0.006332062 | LEUK |
| SLC44A1 | 3.51E-06 | 0.884679012 | 0.238 | 0 | 0.007017901 | LEUK |
| TRGC1 | 3.74E-06 | -0.431204473 | 0.78 | 0.103 | 0.007484188 | LEUK |
| BIRC3 | 3.87E-06 | 0.725968079 | 0.253 | 0.069 | 0.007749216 | LEUK |
| RGS2 | 4.10E-06 | -0.717748687 | 0.879 | 0.138 | 0.008191197 | LEUK |
| KCNIP4 | 4.97E-06 | 0.520063979 | 0.886 | 0.172 | 0.009939929 | LEUK |
| SLC4A4 | 5.19E-06 | -0.365545642 | 0.941 | 0.207 | 0.01037957 | LEUK |
| TMEM178B | 5.70E-06 | -0.480587875 | 0.842 | 0.103 | 0.01139702 | LEUK |
| ATP2B4 | 5.96E-06 | -0.39609061 | 0.297 | 0.241 | 0.011922513 | LEUK |
| FBN2 | 6.25E-06 | -0.620298768 | 0.868 | 0.138 | 0.012502962 | LEUK |
| MS4A1 | 6.52E-06 | 1.375772383 | 0.245 | 0 | 0.013042128 | LEUK |
| UNC80 | 8.11E-06 | -0.35949926 | 0.626 | 0.034 | 0.01621161 | LEUK |
| AC007368.1 | 9.12E-06 | 1.056764612 | 0.256 | 0.034 | 0.018248426 | LEUK |
| DRAIC | 1.07E-05 | -0.291469124 | 0.278 | 0.103 | 0.02134904 | LEUK |
| TCF4 | 1.07E-05 | -0.535151915 | 0.289 | 0.172 | 0.021378582 | LEUK |
| RBFOX1 | 1.13E-05 | -0.283957784 | 0.894 | 0.172 | 0.022508798 | LEUK |
| MS4A4E | 1.15E-05 | -0.556278198 | 0.821 | 0.103 | 0.023053703 | LEUK |
| LY75-CD302 | 1.16E-05 | 0.324114796 | 0.234 | 0.034 | 0.023179269 | LEUK |
| LINC01036 | 1.19E-05 | 0.601771129 | 0.253 | 0 | 0.023848333 | LEUK |
| SEMA6D | 1.23E-05 | -0.447514667 | 0.802 | 0.069 | 0.024635867 | LEUK |
| GNLY | 1.40E-05 | -0.605622165 | 0.857 | 0.138 | 0.028014227 | LEUK |
| PPP2R2B | 1.42E-05 | 0.39092806 | 0.267 | 0.069 | 0.028424046 | LEUK |
| SLC25A48 | 1.60E-05 | 0.368855366 | 0.744 | 0 | 0.032053843 | LEUK |
| CHGB | 2.28E-05 | -0.254388699 | 0.762 | 0.034 | 0.045514507 | LEUK |
| VCL | 2.29E-05 | -0.813901637 | 1 | 0.276 | 0.045791645 | LEUK |
| LIN52 | 2.34E-05 | -0.321283837 | 1 | 0.276 | 0.046711948 | LEUK |
| PDGFD | 8.21E-33 | 0.384664456 | 0.955 | 0.04 | 1.64E-29 | MES |
| AC092078.2 | 2.93E-32 | -0.286665402 | 0.929 | 0.056 | 5.85E-29 | MES |
| AC018742.1 | 1.00E-29 | 0.323011532 | 0.92 | 0.032 | 2.01E-26 | MES |
| LINC01098 | 2.73E-28 | 0.284252162 | 0.116 | 0.064 | 5.46E-25 | MES |
| UMOD | 6.49E-28 | -0.262398198 | 0.964 | 0.08 | 1.30E-24 | MES |
| FAT3 | 1.70E-27 | 0.260082332 | 0.982 | 0.104 | 3.40E-24 | MES |
| PDE4B | 4.26E-27 | 0.284576146 | 0.125 | 0.056 | 8.53E-24 | MES |
| LINC01762 | 8.53E-27 | -0.267823747 | 0.946 | 0.072 | 1.71E-23 | MES |
| PRKAR2B | 1.69E-26 | 0.381188976 | 0.964 | 0.096 | 3.38E-23 | MES |
| MT1M | 5.63E-26 | -0.58505268 | 0.973 | 0.112 | 1.13E-22 | MES |
| SPSB1 | 6.47E-26 | -0.265811714 | 0.134 | 0.128 | 1.29E-22 | MES |
| TSHZ2 | 4.60E-25 | -0.320242158 | 0.143 | 0.104 | 9.20E-22 | MES |
| LINC02237 | 1.05E-24 | 0.850114258 | 0.759 | 0.048 | 2.11E-21 | MES |
| EGR3 | 1.12E-24 | 0.353645907 | 0.143 | 0.032 | 2.24E-21 | MES |
| HMCN1 | 1.87E-23 | 0.443269667 | 0.152 | 0.04 | 3.75E-20 | MES |
| MT1X | 3.17E-23 | -0.398807637 | 1 | 0.144 | 6.35E-20 | MES |
| GEM | 4.34E-23 | 0.379023258 | 0.152 | 0.128 | 8.68E-20 | MES |
| AC096577.1 | 7.81E-23 | -0.304820116 | 0.964 | 0.12 | 1.56E-19 | MES |
| AC007744.1 | 1.09E-22 | -0.301742206 | 0.982 | 0.136 | 2.17E-19 | MES |
| MIOX | 2.20E-22 | -0.675577748 | 1 | 0.152 | 4.40E-19 | MES |
| SLC12A3 | 3.07E-22 | -0.768458731 | 0.973 | 0.136 | 6.15E-19 | MES |
| GDF15 | 4.92E-22 | -0.312159027 | 0.161 | 0.056 | 9.83E-19 | MES |
| NKAIN2 | 1.17E-21 | 0.340776534 | 0.17 | 0.08 | 2.34E-18 | MES |
| SLC13A3 | 2.89E-21 | -0.470944977 | 1 | 0.16 | 5.79E-18 | MES |
| MAN1C1 | 4.15E-21 | 0.380209053 | 0.946 | 0.128 | 8.31E-18 | MES |
| KCNJ15 | 7.61E-21 | -0.443102212 | 0.964 | 0.136 | 1.52E-17 | MES |
| C1orf112 | 1.05E-20 | -0.289032669 | 0.964 | 0.136 | 2.10E-17 | MES |
| DGKI | 1.68E-20 | 0.339083082 | 0.179 | 0.048 | 3.36E-17 | MES |
| TINAG | 4.81E-20 | -0.455045552 | 0.964 | 0.144 | 9.61E-17 | MES |
| GRIA4 | 5.31E-20 | 0.298244673 | 0.946 | 0.136 | 1.06E-16 | MES |
| GRIN2A | 1.04E-19 | 0.257524798 | 0.973 | 0.168 | 2.08E-16 | MES |
| SLC26A7 | 1.18E-19 | -0.456008755 | 0.991 | 0.176 | 2.36E-16 | MES |
| AC073941.1 | 3.74E-19 | 0.441240451 | 0.188 | 0.104 | 7.47E-16 | MES |
| FGF14 | 1.18E-18 | 0.296177077 | 0.188 | 0.024 | 2.35E-15 | MES |
| AL359232.1 | 1.76E-18 | 0.365089314 | 0.196 | 0.056 | 3.52E-15 | MES |
| LSAMP | 3.06E-17 | -0.516046197 | 1 | 0.208 | 6.11E-14 | MES |
| ARRDC4 | 5.64E-17 | -0.255667979 | 0.902 | 0.12 | 1.13E-13 | MES |
| CYP3A5 | 7.54E-17 | -0.269260583 | 0.991 | 0.2 | 1.51E-13 | MES |
| GPAT3 | 9.37E-17 | -0.505930412 | 0.991 | 0.2 | 1.87E-13 | MES |
| TGM2 | 2.46E-16 | -0.300811203 | 0.223 | 0.12 | 4.93E-13 | MES |
| TIMP1 | 2.98E-16 | 0.350259884 | 0.821 | 0.048 | 5.96E-13 | MES |
| KBTBD12 | 3.74E-16 | -0.303070856 | 0.946 | 0.168 | 7.48E-13 | MES |
| PTP4A1 | 4.92E-16 | -0.393493937 | 0.973 | 0.192 | 9.85E-13 | MES |
| LINC00871 | 5.28E-16 | -0.405865979 | 0.92 | 0.144 | 1.06E-12 | MES |
| SLC22A6 | 1.86E-15 | -0.275266979 | 0.857 | 0.088 | 3.71E-12 | MES |
| CA12 | 3.62E-15 | -0.500363173 | 0.982 | 0.216 | 7.23E-12 | MES |
| CELF2 | 4.32E-15 | -0.461864515 | 0.982 | 0.208 | 8.64E-12 | MES |
| SLC2A3 | 6.57E-15 | 0.775218605 | 0.232 | 0.08 | 1.31E-11 | MES |
| ACO1 | 9.92E-15 | -0.253052304 | 0.25 | 0.144 | 1.98E-11 | MES |
| SLC12A1 | 1.12E-14 | -0.405172501 | 0.884 | 0.128 | 2.24E-11 | MES |
| ABCC9 | 1.21E-14 | 0.460855679 | 0.241 | 0.128 | 2.42E-11 | MES |
| RHOJ | 1.75E-14 | 0.405795323 | 0.241 | 0.136 | 3.50E-11 | MES |
| EYA1 | 2.32E-14 | -0.272403379 | 0.116 | 0.056 | 4.63E-11 | MES |
| TOX | 2.36E-14 | 0.323830202 | 0.982 | 0.24 | 4.73E-11 | MES |
| AL365295.1 | 2.46E-14 | 0.331570651 | 0.241 | 0.072 | 4.92E-11 | MES |
| ENTPD1 | 2.62E-14 | -0.271207564 | 0.982 | 0.232 | 5.24E-11 | MES |
| RGCC | 6.67E-14 | -0.284226492 | 0.991 | 0.232 | 1.33E-10 | MES |
| LINC01435 | 1.49E-13 | -0.53454646 | 0.982 | 0.232 | 2.98E-10 | MES |
| OPCML | 5.26E-13 | 0.439424675 | 0.991 | 0.272 | 1.05E-09 | MES |
| ZNF804A | 6.90E-13 | 1.029205188 | 0.259 | 0.08 | 1.38E-09 | MES |
| EGF | 9.69E-13 | -0.599558252 | 0.884 | 0.152 | 1.94E-09 | MES |
| IL1RL1 | 1.06E-12 | -0.684640676 | 0.241 | 0.152 | 2.11E-09 | MES |
| ANOS1 | 1.41E-12 | 0.3263146 | 0.875 | 0.152 | 2.82E-09 | MES |
| TMTC2 | 1.00E-11 | 0.398932548 | 0.277 | 0.096 | 2.01E-08 | MES |
| EDIL3-DT | 1.40E-11 | 0.43908609 | 0.277 | 0.128 | 2.80E-08 | MES |
| SLC16A9 | 1.45E-11 | -0.346205815 | 1 | 0.264 | 2.91E-08 | MES |
| ACSM2A | 1.46E-11 | -0.99649713 | 0.982 | 0.256 | 2.93E-08 | MES |
| SLC35F1 | 3.70E-11 | 0.402237398 | 0.946 | 0.264 | 7.41E-08 | MES |
| CREB5 | 6.50E-11 | 0.354917369 | 0.277 | 0.024 | 1.30E-07 | MES |
| CRYAB | 7.99E-11 | -0.330831742 | 0.786 | 0.072 | 1.60E-07 | MES |
| SLCO2A1 | 1.38E-10 | 0.959720572 | 0.286 | 0.112 | 2.76E-07 | MES |
| TNC | 2.07E-10 | 0.348338234 | 0.295 | 0.096 | 4.14E-07 | MES |
| TENT5A | 4.52E-10 | 0.399825138 | 0.312 | 0.208 | 9.03E-07 | MES |
| NLGN4Y | 5.21E-10 | 0.308695686 | 0.295 | 0.064 | 1.04E-06 | MES |
| PTCHD4 | 7.60E-10 | 0.29433084 | 0.777 | 0.08 | 1.52E-06 | MES |
| RAB31 | 9.40E-10 | -0.272038805 | 0.339 | 0.336 | 1.88E-06 | MES |
| CDH6 | 1.31E-09 | 0.296694227 | 0.786 | 0.096 | 2.61E-06 | MES |
| NRK | 1.87E-09 | -0.302339229 | 0.321 | 0.128 | 3.73E-06 | MES |
| PPFIA2 | 2.69E-09 | -0.364948253 | 0.33 | 0.16 | 5.39E-06 | MES |
| CNTN1 | 5.31E-09 | 0.306713694 | 0.955 | 0.312 | 1.06E-05 | MES |
| MT1E | 5.31E-09 | 0.271051399 | 0.982 | 0.312 | 1.06E-05 | MES |
| ERRFI1 | 9.80E-09 | -0.32626527 | 0.964 | 0.288 | 1.96E-05 | MES |
| CASR | 1.02E-08 | -0.31614862 | 0.75 | 0.064 | 2.04E-05 | MES |
| DLGAP1 | 1.14E-08 | -0.263674767 | 0.991 | 0.32 | 2.29E-05 | MES |
| C8orf34 | 1.54E-08 | 0.496553647 | 0.741 | 0.064 | 3.08E-05 | MES |
| SLC16A12 | 1.65E-08 | -0.301036384 | 0.339 | 0.144 | 3.30E-05 | MES |
| NID1 | 2.42E-08 | -0.278873325 | 0.366 | 0.272 | 4.83E-05 | MES |
| KCNQ5 | 4.55E-08 | 0.736701055 | 1 | 0.368 | 9.10E-05 | MES |
| PGAP1 | 5.30E-08 | 0.277801513 | 0.339 | 0.16 | 0.000106081 | MES |
| COL23A1 | 1.14E-07 | 0.686896738 | 0.946 | 0.328 | 0.00022773 | MES |
| SERPINE1 | 1.14E-07 | -1.214171896 | 0.116 | 0.032 | 0.000228848 | MES |
| HSPB1 | 1.43E-07 | -0.567788523 | 0.375 | 0.216 | 0.000286384 | MES |
| P3H2 | 2.77E-07 | -0.260915109 | 0.911 | 0.28 | 0.000553939 | MES |
| KCNIP4 | 3.35E-07 | 0.444727944 | 1 | 0.376 | 0.00066946 | MES |
| STIM2 | 3.95E-07 | -0.329918717 | 0.402 | 0.288 | 0.000790721 | MES |
| GRID2 | 4.60E-07 | 0.500993429 | 0.964 | 0.4 | 0.000920496 | MES |
| ACSM2B | 4.65E-07 | -1.048993291 | 0.911 | 0.256 | 0.000929203 | MES |
| BHLHE40 | 4.91E-07 | 0.615705444 | 0.339 | 0.088 | 0.000982817 | MES |
| AL137782.1 | 5.97E-07 | -0.404575925 | 0.411 | 0.336 | 0.001193741 | MES |
| LRP2 | 7.89E-07 | -0.498206512 | 0.848 | 0.208 | 0.001577261 | MES |
| AGTR1 | 1.18E-06 | -0.647699996 | 0.429 | 0.328 | 0.002363217 | MES |
| TG | 1.46E-06 | 0.272008498 | 0.366 | 0.184 | 0.00292938 | MES |
| DACH1 | 1.77E-06 | 0.308334396 | 0.866 | 0.256 | 0.003540311 | MES |
| GADD45B | 1.87E-06 | 0.524923714 | 0.786 | 0.176 | 0.003744626 | MES |
| AFM | 6.00E-06 | -0.283013456 | 0.723 | 0.088 | 0.012001001 | MES |
| HIVEP3 | 7.10E-06 | 0.378404174 | 0.982 | 0.416 | 0.014198837 | MES |
| TIPARP | 7.16E-06 | -0.405863648 | 1 | 0.384 | 0.014315407 | MES |
| PDZRN4 | 7.34E-06 | 0.252956188 | 0.929 | 0.368 | 0.014670039 | MES |
| CUBN | 1.06E-05 | -0.434652464 | 0.973 | 0.368 | 0.021211664 | MES |
| ZFPM2 | 1.89E-05 | 0.388568012 | 0.982 | 0.496 | 0.037874118 | MES |
| MIR646HG | 2.32E-05 | -0.291115332 | 0.741 | 0.128 | 0.046438158 | MES |
| DCHS2 | 1.43E-30 | 0.408247892 | 0.968 | 0.013 | 2.87E-27 | FIB |
| AC114689.3 | 1.28E-29 | 0.308600334 | 1 | 0.039 | 2.57E-26 | FIB |
| BTC | 5.06E-29 | -0.291407748 | 0.992 | 0.039 | 1.01E-25 | FIB |
| MIOX | 1.59E-27 | -0.295059943 | 0.992 | 0.052 | 3.18E-24 | FIB |
| PHF21B | 5.72E-27 | -0.291688399 | 0.992 | 0.052 | 1.14E-23 | FIB |
| GDF15 | 6.05E-27 | -0.254224544 | 0.992 | 0.052 | 1.21E-23 | FIB |
| PAH | 2.62E-26 | -0.252479756 | 1 | 0.065 | 5.24E-23 | FIB |
| TNFRSF19 | 2.68E-26 | 0.390593671 | 0.984 | 0.052 | 5.35E-23 | FIB |
| AC092078.2 | 4.14E-26 | -0.541391837 | 0.984 | 0.052 | 8.29E-23 | FIB |
| MT1X | 3.14E-25 | -0.265410461 | 0.976 | 0.052 | 6.28E-22 | FIB |
| IL1RL1 | 3.93E-25 | -0.266303217 | 0.944 | 0.026 | 7.86E-22 | FIB |
| FSTL4 | 5.92E-25 | -0.397816709 | 0.96 | 0.039 | 1.18E-21 | FIB |
| HELLPAR | 2.55E-24 | -0.250096038 | 0.935 | 0.039 | 5.09E-21 | FIB |
| LINC01505 | 3.39E-24 | 0.755405261 | 0.952 | 0.039 | 6.78E-21 | FIB |
| BAMBI | 3.57E-24 | -0.350922084 | 0.911 | 0.039 | 7.14E-21 | FIB |
| PGR | 4.46E-24 | 0.366766106 | 0.96 | 0.052 | 8.93E-21 | FIB |
| MT1E | 4.53E-24 | -0.679683366 | 0.992 | 0.078 | 9.07E-21 | FIB |
| GALNT13 | 2.30E-23 | -0.51234785 | 1 | 0.091 | 4.60E-20 | FIB |
| AL160272.2 | 3.12E-23 | 0.326438452 | 0.992 | 0.091 | 6.25E-20 | FIB |
| MT1M | 3.25E-23 | -0.74924106 | 0.911 | 0.039 | 6.50E-20 | FIB |
| TMEM108 | 3.36E-23 | -0.272852779 | 0.952 | 0.052 | 6.73E-20 | FIB |
| FREM2 | 5.26E-23 | -0.271918127 | 0.968 | 0.065 | 1.05E-19 | FIB |
| WT1-AS | 1.20E-22 | -0.27445404 | 0.992 | 0.091 | 2.40E-19 | FIB |
| ADM | 1.29E-22 | -0.274390414 | 0.992 | 0.091 | 2.59E-19 | FIB |
| GALNT15 | 1.33E-22 | -0.325414795 | 0.992 | 0.091 | 2.65E-19 | FIB |
| CNTN6 | 1.33E-22 | -0.295762105 | 0.992 | 0.091 | 2.65E-19 | FIB |
| AC109466.1 | 1.56E-22 | -0.326568424 | 0.976 | 0.078 | 3.11E-19 | FIB |
| PRLR | 2.10E-22 | -0.292719621 | 0.976 | 0.078 | 4.21E-19 | FIB |
| UGDH | 2.14E-22 | -0.389094556 | 0.944 | 0.078 | 4.28E-19 | FIB |
| STAP1 | 2.16E-22 | -0.25658572 | 0.903 | 0.039 | 4.33E-19 | FIB |
| HSPB8 | 2.49E-22 | -0.50545998 | 0.944 | 0.078 | 4.99E-19 | FIB |
| TMC1 | 2.51E-22 | -0.280103826 | 0.984 | 0.091 | 5.01E-19 | FIB |
| CSMD3 | 6.73E-22 | -0.254168097 | 0.984 | 0.091 | 1.35E-18 | FIB |
| FGF13 | 7.62E-22 | 0.675155081 | 0.976 | 0.091 | 1.52E-18 | FIB |
| CACNB4 | 9.92E-22 | 0.322653234 | 0.952 | 0.065 | 1.98E-18 | FIB |
| DKK2 | 1.09E-21 | -0.471673461 | 0.992 | 0.104 | 2.18E-18 | FIB |
| BIRC3 | 1.74E-21 | 0.540378069 | 0.105 | 0.026 | 3.49E-18 | FIB |
| AP005230.1 | 2.40E-21 | -0.317611147 | 0.919 | 0.052 | 4.81E-18 | FIB |
| GNGT1 | 2.51E-21 | -0.37852768 | 0.992 | 0.104 | 5.01E-18 | FIB |
| RAMP1 | 2.57E-21 | 0.310562545 | 0.96 | 0.078 | 5.13E-18 | FIB |
| MMP19 | 2.76E-21 | 0.357882833 | 0.911 | 0.065 | 5.52E-18 | FIB |
| KCTD16 | 2.83E-21 | -0.441576893 | 0.992 | 0.104 | 5.67E-18 | FIB |
| GP2 | 3.02E-21 | -0.281003693 | 0.927 | 0.052 | 6.05E-18 | FIB |
| CD34 | 3.05E-21 | -0.407265209 | 0.992 | 0.104 | 6.09E-18 | FIB |
| AGTR1 | 4.45E-21 | 0.290524323 | 0.871 | 0.039 | 8.90E-18 | FIB |
| SFRP1 | 4.50E-21 | -0.326764347 | 0.121 | 0.078 | 8.99E-18 | FIB |
| ZSCAN31 | 1.09E-20 | -0.306317821 | 0.968 | 0.104 | 2.18E-17 | FIB |
| AC104041.1 | 1.21E-20 | 0.445013354 | 0.903 | 0.026 | 2.42E-17 | FIB |
| KLHL1 | 1.53E-20 | 0.292194033 | 0.121 | 0 | 3.06E-17 | FIB |
| ANPEP | 3.01E-20 | -0.287141311 | 0.952 | 0.078 | 6.01E-17 | FIB |
| CYSLTR1 | 3.87E-20 | -0.623113781 | 0.911 | 0.052 | 7.74E-17 | FIB |
| TTC29 | 4.56E-20 | -0.324848193 | 0.919 | 0.052 | 9.11E-17 | FIB |
| AC002074.1 | 5.43E-20 | 0.351845916 | 0.919 | 0.091 | 1.09E-16 | FIB |
| KCNB2 | 6.57E-20 | -0.661659991 | 0.992 | 0.117 | 1.31E-16 | FIB |
| ARMC4 | 8.11E-20 | -0.438038872 | 0.944 | 0.078 | 1.62E-16 | FIB |
| LRRC9 | 8.94E-20 | -0.280324104 | 0.137 | 0.13 | 1.79E-16 | FIB |
| KCNH1 | 9.34E-20 | -0.31838717 | 0.137 | 0.117 | 1.87E-16 | FIB |
| ST6GALNAC5 | 1.06E-19 | -0.350296774 | 0.96 | 0.091 | 2.12E-16 | FIB |
| RNF212B | 1.60E-19 | -0.341878634 | 1 | 0.13 | 3.20E-16 | FIB |
| BRINP1 | 1.77E-19 | 0.388430077 | 0.121 | 0 | 3.53E-16 | FIB |
| PAG1 | 1.77E-19 | 0.272627484 | 0.952 | 0.091 | 3.55E-16 | FIB |
| DACH2 | 1.98E-19 | 0.995626657 | 0.113 | 0.052 | 3.96E-16 | FIB |
| RRAD | 2.02E-19 | -0.390915925 | 0.944 | 0.078 | 4.04E-16 | FIB |
| LINC02511 | 2.34E-19 | -0.964996714 | 0.879 | 0.078 | 4.68E-16 | FIB |
| SCIN | 2.36E-19 | -0.402798366 | 0.968 | 0.104 | 4.72E-16 | FIB |
| AC093865.1 | 3.58E-19 | -0.327561008 | 0.968 | 0.104 | 7.16E-16 | FIB |
| CLNK | 7.33E-19 | -0.29523046 | 0.992 | 0.13 | 1.47E-15 | FIB |
| IL18 | 8.05E-19 | -0.468728513 | 0.919 | 0.091 | 1.61E-15 | FIB |
| MCTP1 | 2.34E-18 | 0.368825592 | 0.145 | 0.026 | 4.68E-15 | FIB |
| PRR16 | 2.91E-18 | 0.533496079 | 0.976 | 0.13 | 5.83E-15 | FIB |
| DUSP5 | 2.91E-18 | -0.369792622 | 0.984 | 0.13 | 5.83E-15 | FIB |
| KIRREL3 | 3.39E-18 | 0.98169822 | 0.145 | 0 | 6.78E-15 | FIB |
| LINC01187 | 4.43E-18 | -0.258744133 | 0.919 | 0.078 | 8.85E-15 | FIB |
| AC013271.1 | 9.04E-18 | -0.324241722 | 0.992 | 0.143 | 1.81E-14 | FIB |
| MMP16 | 9.45E-18 | -0.608975303 | 0.992 | 0.143 | 1.89E-14 | FIB |
| LINC00871 | 9.48E-18 | -0.432912005 | 0.161 | 0.104 | 1.90E-14 | FIB |
| KLF2 | 9.48E-18 | -0.438934146 | 0.161 | 0.104 | 1.90E-14 | FIB |
| DUSP10 | 1.23E-17 | 0.372935884 | 0.871 | 0.026 | 2.46E-14 | FIB |
| NPR3 | 1.28E-17 | 0.274187166 | 0.153 | 0.039 | 2.55E-14 | FIB |
| FAM198B-AS1 | 1.50E-17 | 0.307314912 | 0.113 | 0 | 3.00E-14 | FIB |
| H19 | 1.61E-17 | -0.618684936 | 0.952 | 0.13 | 3.22E-14 | FIB |
| STXBP6 | 2.44E-17 | -0.621478027 | 0.976 | 0.13 | 4.88E-14 | FIB |
| AC008415.1 | 2.51E-17 | -0.581125341 | 0.871 | 0.052 | 5.01E-14 | FIB |
| GASK1B | 2.72E-17 | 0.387161954 | 0.911 | 0.078 | 5.44E-14 | FIB |
| SLC25A21 | 3.17E-17 | -0.43111172 | 0.169 | 0.117 | 6.35E-14 | FIB |
| KCND2 | 3.43E-17 | 0.262491537 | 0.137 | 0.026 | 6.87E-14 | FIB |
| CHRM3 | 3.57E-17 | -0.442270421 | 1 | 0.156 | 7.14E-14 | FIB |
| EGF | 3.69E-17 | 0.645117688 | 0.968 | 0.13 | 7.37E-14 | FIB |
| ANXA2 | 4.90E-17 | -0.583676835 | 0.177 | 0.195 | 9.80E-14 | FIB |
| SORCS3 | 6.08E-17 | -0.726568067 | 0.185 | 0.195 | 1.22E-13 | FIB |
| ITIH5 | 6.25E-17 | 0.329547228 | 0.161 | 0.026 | 1.25E-13 | FIB |
| NCAM2 | 6.36E-17 | 0.334136356 | 0.887 | 0.091 | 1.27E-13 | FIB |
| MAFF | 6.90E-17 | -0.415908904 | 0.169 | 0.078 | 1.38E-13 | FIB |
| RELL1 | 8.73E-17 | 0.293824841 | 0.161 | 0.013 | 1.75E-13 | FIB |
| MAP3K14 | 9.15E-17 | -0.261954825 | 0.169 | 0.091 | 1.83E-13 | FIB |
| CXXC4-AS1 | 1.02E-16 | 0.267212855 | 0.161 | 0 | 2.04E-13 | FIB |
| KCNB1 | 1.03E-16 | -0.524897046 | 0.903 | 0.091 | 2.06E-13 | FIB |
| ADRA1A | 1.09E-16 | -0.527353241 | 0.992 | 0.156 | 2.18E-13 | FIB |
| AL365295.1 | 1.11E-16 | -0.585291786 | 0.177 | 0.195 | 2.21E-13 | FIB |
| STOM | 1.11E-16 | -0.435249373 | 0.177 | 0.13 | 2.21E-13 | FIB |
| DCDC2 | 1.17E-16 | -0.594623973 | 0.177 | 0.143 | 2.35E-13 | FIB |
| IGFBP6 | 1.38E-16 | -1.022421231 | 0.992 | 0.156 | 2.75E-13 | FIB |
| AMPH | 1.52E-16 | 0.257055624 | 0.145 | 0 | 3.05E-13 | FIB |
| RIPOR2 | 1.69E-16 | -0.576893969 | 0.984 | 0.156 | 3.39E-13 | FIB |
| SPP1 | 1.73E-16 | -0.557379632 | 0.895 | 0.065 | 3.46E-13 | FIB |
| CRYAB | 1.75E-16 | -0.917981972 | 0.992 | 0.156 | 3.49E-13 | FIB |
| HMGCS1 | 2.06E-16 | -0.925657278 | 0.177 | 0.104 | 4.12E-13 | FIB |
| TRAF1 | 2.43E-16 | 0.323812308 | 0.169 | 0.065 | 4.86E-13 | FIB |
| FMN2 | 2.72E-16 | -0.572354974 | 0.992 | 0.156 | 5.44E-13 | FIB |
| CASR | 3.05E-16 | 0.270533333 | 0.879 | 0.052 | 6.10E-13 | FIB |
| PXYLP1 | 4.04E-16 | 0.351650685 | 0.169 | 0.026 | 8.07E-13 | FIB |
| ST6GALNAC3 | 5.09E-16 | 0.267376762 | 0.887 | 0.065 | 1.02E-12 | FIB |
| HOPX | 7.35E-16 | 0.376601052 | 0.121 | 0 | 1.47E-12 | FIB |
| SSPN | 7.74E-16 | -0.331425838 | 0.903 | 0.091 | 1.55E-12 | FIB |
| CP | 7.91E-16 | 0.269631585 | 0.153 | 0 | 1.58E-12 | FIB |
| RETREG1 | 7.99E-16 | -0.324435152 | 1 | 0.169 | 1.60E-12 | FIB |
| RCAN1 | 9.16E-16 | 0.308580179 | 0.177 | 0.078 | 1.83E-12 | FIB |
| PCOLCE2 | 1.38E-15 | -0.50824822 | 0.992 | 0.169 | 2.76E-12 | FIB |
| CD74 | 1.42E-15 | -0.308650021 | 0.121 | 0.026 | 2.85E-12 | FIB |
| FHL5 | 1.48E-15 | 0.299734354 | 0.855 | 0.039 | 2.96E-12 | FIB |
| MGST1 | 1.51E-15 | -0.560505014 | 0.992 | 0.169 | 3.03E-12 | FIB |
| AC105383.1 | 1.84E-15 | 0.275780038 | 0.161 | 0.039 | 3.69E-12 | FIB |
| RAB31 | 2.27E-15 | 0.379957031 | 1 | 0.195 | 4.54E-12 | FIB |
| AC079298.3 | 3.87E-15 | -0.332318666 | 0.879 | 0.065 | 7.74E-12 | FIB |
| GPAT3 | 3.98E-15 | -0.452785144 | 0.944 | 0.13 | 7.96E-12 | FIB |
| MYOCD | 5.60E-15 | 0.349174592 | 0.145 | 0 | 1.12E-11 | FIB |
| ABLIM3 | 6.04E-15 | -0.313983455 | 0.194 | 0.091 | 1.21E-11 | FIB |
| NIBAN1 | 6.56E-15 | -0.356684463 | 0.194 | 0.078 | 1.31E-11 | FIB |
| XKR4 | 7.32E-15 | 0.613622107 | 0.185 | 0.026 | 1.46E-11 | FIB |
| ANKRD33B | 7.73E-15 | -0.805842296 | 1 | 0.182 | 1.55E-11 | FIB |
| C3 | 9.06E-15 | -1.003921855 | 1 | 0.182 | 1.81E-11 | FIB |
| KLF5 | 9.62E-15 | -0.807368943 | 1 | 0.182 | 1.92E-11 | FIB |
| VIM | 1.10E-14 | -0.642099114 | 0.218 | 0.234 | 2.20E-11 | FIB |
| LINC02398 | 3.30E-14 | 0.400569772 | 0.177 | 0.065 | 6.61E-11 | FIB |
| SMS | 3.36E-14 | 0.252502051 | 0.887 | 0.091 | 6.71E-11 | FIB |
| PREX2 | 3.37E-14 | 0.555068478 | 0.879 | 0.117 | 6.73E-11 | FIB |
| KCNE4 | 3.49E-14 | 0.307658673 | 0.153 | 0.026 | 6.97E-11 | FIB |
| H2AC6 | 4.05E-14 | 0.566600059 | 0.194 | 0.013 | 8.11E-11 | FIB |
| PTGS2 | 5.00E-14 | 0.426091712 | 0.185 | 0.026 | 1.00E-10 | FIB |
| NLGN4X | 5.06E-14 | 0.321729781 | 0.194 | 0.039 | 1.01E-10 | FIB |
| RIMKLB | 6.26E-14 | -0.30405087 | 0.226 | 0.234 | 1.25E-10 | FIB |
| LINC02456 | 7.18E-14 | -1.121858324 | 0.976 | 0.182 | 1.44E-10 | FIB |
| FXYD6-FXYD2 | 8.12E-14 | 0.373743329 | 0.847 | 0.052 | 1.62E-10 | FIB |
| SLC16A12 | 9.09E-14 | -0.523058965 | 0.968 | 0.169 | 1.82E-10 | FIB |
| NTM | 1.03E-13 | -0.886815838 | 0.218 | 0.156 | 2.07E-10 | FIB |
| HRH1 | 1.04E-13 | -0.601514005 | 0.935 | 0.143 | 2.07E-10 | FIB |
| AHR | 1.25E-13 | -0.331807703 | 0.218 | 0.208 | 2.50E-10 | FIB |
| ETS1 | 1.61E-13 | 0.357840508 | 0.952 | 0.169 | 3.23E-10 | FIB |
| INHBA | 1.70E-13 | 0.339808492 | 0.161 | 0.013 | 3.40E-10 | FIB |
| ERO1B | 1.80E-13 | -0.274450033 | 0.218 | 0.156 | 3.60E-10 | FIB |
| CREB5 | 2.20E-13 | -0.677754701 | 0.992 | 0.195 | 4.41E-10 | FIB |
| EGFEM1P | 2.50E-13 | -0.384843508 | 0.879 | 0.104 | 5.00E-10 | FIB |
| ZNF385B | 2.51E-13 | -0.574183947 | 0.226 | 0.143 | 5.03E-10 | FIB |
| NTNG1 | 2.60E-13 | -0.282592324 | 0.879 | 0.091 | 5.20E-10 | FIB |
| LINC01950 | 2.61E-13 | -0.449481719 | 0.645 | 0.052 | 5.23E-10 | FIB |
| DCLK2 | 3.95E-13 | 0.264944136 | 0.968 | 0.195 | 7.90E-10 | FIB |
| CDH13 | 4.34E-13 | -0.41213108 | 0.234 | 0.221 | 8.68E-10 | FIB |
| DDIT4 | 5.29E-13 | -0.643098584 | 0.952 | 0.182 | 1.06E-09 | FIB |
| ADGRF5 | 6.30E-13 | 0.464471683 | 0.968 | 0.208 | 1.26E-09 | FIB |
| ZNF385D | 6.30E-13 | -1.742035452 | 1 | 0.208 | 1.26E-09 | FIB |
| MYH11 | 7.00E-13 | 0.44442803 | 0.194 | 0.039 | 1.40E-09 | FIB |
| PIP5K1B | 7.30E-13 | -0.347239868 | 0.992 | 0.208 | 1.46E-09 | FIB |
| CHN1 | 7.53E-13 | 0.284381049 | 0.21 | 0.013 | 1.51E-09 | FIB |
| COL14A1 | 7.57E-13 | 0.292446997 | 0.976 | 0.208 | 1.51E-09 | FIB |
| SQSTM1 | 7.85E-13 | -0.483479265 | 0.234 | 0.195 | 1.57E-09 | FIB |
| CLMP | 1.17E-12 | -0.292661119 | 0.226 | 0.221 | 2.34E-09 | FIB |
| TIMP1 | 1.66E-12 | -1.197932093 | 0.218 | 0.052 | 3.32E-09 | FIB |
| HPSE2 | 1.82E-12 | -0.485182705 | 0.903 | 0.13 | 3.65E-09 | FIB |
| CYTOR | 1.91E-12 | 0.375939045 | 1 | 0.247 | 3.83E-09 | FIB |
| NKD1 | 2.35E-12 | 0.571162726 | 0.194 | 0.039 | 4.71E-09 | FIB |
| PHACTR1 | 2.46E-12 | 0.604151973 | 0.218 | 0.052 | 4.92E-09 | FIB |
| WT1 | 3.66E-12 | -0.44669286 | 0.984 | 0.208 | 7.32E-09 | FIB |
| KCNAB1 | 3.83E-12 | -0.919547594 | 0.992 | 0.208 | 7.65E-09 | FIB |
| LINC01619 | 3.91E-12 | 0.255044263 | 0.226 | 0.078 | 7.81E-09 | FIB |
| MET | 4.62E-12 | -0.319393696 | 0.911 | 0.143 | 9.23E-09 | FIB |
| TMEM178B | 4.62E-12 | 0.645274287 | 0.226 | 0.065 | 9.24E-09 | FIB |
| SEMA3A | 5.08E-12 | 0.436886513 | 0.823 | 0.052 | 1.02E-08 | FIB |
| ADAM12 | 5.66E-12 | 0.359097984 | 0.226 | 0.052 | 1.13E-08 | FIB |
| KDR | 7.24E-12 | -0.604397128 | 0.927 | 0.156 | 1.45E-08 | FIB |
| LINC01060 | 7.72E-12 | -0.274467249 | 0.234 | 0.078 | 1.54E-08 | FIB |
| DLGAP1 | 7.79E-12 | 0.302897291 | 0.234 | 0.117 | 1.56E-08 | FIB |
| COL25A1 | 8.54E-12 | 1.045300931 | 0.226 | 0.052 | 1.71E-08 | FIB |
| VCAM1 | 8.57E-12 | 1.109906138 | 0.226 | 0.039 | 1.71E-08 | FIB |
| FILIP1 | 1.01E-11 | 0.393977104 | 0.984 | 0.247 | 2.01E-08 | FIB |
| NFKBIZ | 1.04E-11 | -0.549985372 | 0.266 | 0.247 | 2.08E-08 | FIB |
| CYP3A5 | 1.06E-11 | -0.292478071 | 1 | 0.234 | 2.13E-08 | FIB |
| NUDT4 | 1.13E-11 | -0.282580059 | 0.879 | 0.117 | 2.26E-08 | FIB |
| ITPKC | 1.19E-11 | -1.439315812 | 1 | 0.221 | 2.37E-08 | FIB |
| TCIM | 1.20E-11 | 0.365341189 | 0.226 | 0.026 | 2.41E-08 | FIB |
| SLC35F1 | 1.27E-11 | 0.323024153 | 0.234 | 0.078 | 2.54E-08 | FIB |
| RGS6 | 1.27E-11 | -1.044600948 | 0.992 | 0.221 | 2.54E-08 | FIB |
| MGAT4C | 3.80E-11 | -0.618115272 | 0.952 | 0.195 | 7.60E-08 | FIB |
| HEG1 | 4.73E-11 | -0.445389334 | 0.976 | 0.221 | 9.46E-08 | FIB |
| HECW1 | 4.85E-11 | 0.35817914 | 0.798 | 0.039 | 9.71E-08 | FIB |
| AL390334.1 | 5.26E-11 | -0.496411087 | 1 | 0.247 | 1.05E-07 | FIB |
| PGAP1 | 8.01E-11 | 0.354618356 | 0.879 | 0.143 | 1.60E-07 | FIB |
| RYR3 | 8.15E-11 | -0.904771053 | 0.952 | 0.195 | 1.63E-07 | FIB |
| NLGN4Y | 9.39E-11 | 0.539156209 | 0.242 | 0.052 | 1.88E-07 | FIB |
| CTNNA3 | 1.04E-10 | 0.284341969 | 0.25 | 0.078 | 2.08E-07 | FIB |
| BMP5 | 1.17E-10 | 0.460007919 | 0.839 | 0.104 | 2.34E-07 | FIB |
| TRDN | 1.36E-10 | -0.280486642 | 0.823 | 0.078 | 2.72E-07 | FIB |
| PACRG | 1.43E-10 | -0.308764814 | 0.952 | 0.208 | 2.87E-07 | FIB |
| VCAN | 1.46E-10 | 0.839165695 | 0.25 | 0.117 | 2.91E-07 | FIB |
| KLF6 | 1.50E-10 | -0.370432039 | 0.968 | 0.221 | 3.00E-07 | FIB |
| KCNMB2 | 1.67E-10 | -0.263304931 | 0.992 | 0.247 | 3.34E-07 | FIB |
| SLC12A3 | 2.07E-10 | 0.261722012 | 0.823 | 0.078 | 4.13E-07 | FIB |
| GPRC5A | 2.32E-10 | -0.318688085 | 0.815 | 0.078 | 4.64E-07 | FIB |
| EMP1 | 2.52E-10 | -0.272915213 | 1 | 0.247 | 5.03E-07 | FIB |
| LINC01876 | 2.65E-10 | -0.330839965 | 0.823 | 0.078 | 5.30E-07 | FIB |
| ZBTB7C | 3.97E-10 | -0.813725636 | 1 | 0.247 | 7.93E-07 | FIB |
| RGS16 | 4.10E-10 | 1.007716887 | 0.242 | 0.026 | 8.21E-07 | FIB |
| TNIK | 4.93E-10 | -0.446490282 | 0.919 | 0.182 | 9.85E-07 | FIB |
| PHACTR3 | 5.40E-10 | 0.251409079 | 0.242 | 0.039 | 1.08E-06 | FIB |
| F3 | 5.69E-10 | -0.788052428 | 0.992 | 0.247 | 1.14E-06 | FIB |
| MPPED2 | 6.09E-10 | -0.563787572 | 0.282 | 0.143 | 1.22E-06 | FIB |
| MT2A | 6.54E-10 | -1.661028215 | 0.944 | 0.208 | 1.31E-06 | FIB |
| POSTN | 7.30E-10 | -0.668234781 | 1 | 0.247 | 1.46E-06 | FIB |
| RYR2 | 7.93E-10 | 0.270831299 | 0.266 | 0.091 | 1.59E-06 | FIB |
| ENPEP | 8.71E-10 | 0.330072947 | 0.258 | 0.052 | 1.74E-06 | FIB |
| AC009975.1 | 1.11E-09 | 0.803546853 | 0.258 | 0.052 | 2.22E-06 | FIB |
| ITGA6 | 1.52E-09 | -0.656892587 | 1 | 0.26 | 3.04E-06 | FIB |
| RGCC | 1.63E-09 | -0.941686566 | 0.298 | 0.195 | 3.26E-06 | FIB |
| FBN2 | 1.73E-09 | -0.251718202 | 0.774 | 0.052 | 3.45E-06 | FIB |
| AC015574.1 | 1.83E-09 | 0.528703958 | 0.266 | 0.078 | 3.67E-06 | FIB |
| TMEM131L | 1.86E-09 | 0.579861931 | 0.976 | 0.286 | 3.72E-06 | FIB |
| ERG | 2.20E-09 | 0.256372577 | 0.855 | 0.143 | 4.40E-06 | FIB |
| CYP4Z1 | 2.75E-09 | -0.685004584 | 1 | 0.26 | 5.49E-06 | FIB |
| LINC01098 | 2.98E-09 | 0.447007411 | 0.274 | 0.078 | 5.96E-06 | FIB |
| ALCAM | 4.14E-09 | -0.291259494 | 0.927 | 0.208 | 8.29E-06 | FIB |
| ZNF521 | 4.35E-09 | -0.685596465 | 0.992 | 0.26 | 8.69E-06 | FIB |
| AC124854.1 | 5.52E-09 | 0.565999825 | 0.266 | 0.104 | 1.10E-05 | FIB |
| PTGDS | 5.52E-09 | -1.276566306 | 0.315 | 0.208 | 1.10E-05 | FIB |
| NEBL | 5.92E-09 | -0.276501235 | 0.992 | 0.273 | 1.18E-05 | FIB |
| CCDC39 | 5.94E-09 | -0.761976022 | 0.935 | 0.234 | 1.19E-05 | FIB |
| CDC14A | 6.59E-09 | -0.573891184 | 0.323 | 0.221 | 1.32E-05 | FIB |
| RSPO3 | 7.96E-09 | -0.68663744 | 0.968 | 0.247 | 1.59E-05 | FIB |
| ARHGEF26 | 8.02E-09 | -0.255562481 | 0.927 | 0.221 | 1.60E-05 | FIB |
| TENM2 | 9.15E-09 | -0.666810481 | 1 | 0.273 | 1.83E-05 | FIB |
| PLCE1 | 1.10E-08 | -0.60191784 | 0.984 | 0.26 | 2.21E-05 | FIB |
| H2AC18 | 1.17E-08 | 0.73325249 | 0.266 | 0.013 | 2.34E-05 | FIB |
| DEPP1 | 1.36E-08 | -0.784076063 | 0.839 | 0.13 | 2.72E-05 | FIB |
| ADGRG2 | 2.93E-08 | -0.459463036 | 0.347 | 0.299 | 5.87E-05 | FIB |
| FMN1 | 3.19E-08 | -0.387949847 | 0.315 | 0.156 | 6.38E-05 | FIB |
| GRID2 | 3.66E-08 | -1.008594341 | 0.944 | 0.234 | 7.32E-05 | FIB |
| MGLL | 3.96E-08 | -0.432387658 | 0.944 | 0.247 | 7.91E-05 | FIB |
| SPSB1 | 3.98E-08 | -0.359795577 | 0.952 | 0.26 | 7.95E-05 | FIB |
| TIPARP | 4.82E-08 | -0.579598723 | 1 | 0.286 | 9.65E-05 | FIB |
| CHODL | 5.09E-08 | -0.25337526 | 0.298 | 0.078 | 0.000101825 | FIB |
| NTRK2 | 5.92E-08 | -0.752338165 | 0.984 | 0.273 | 0.000118345 | FIB |
| SPOCK1 | 6.49E-08 | -0.688856672 | 1 | 0.286 | 0.000129771 | FIB |
| KCNIP1 | 6.65E-08 | 0.327854267 | 0.29 | 0.052 | 0.000133099 | FIB |
| TNFAIP2 | 7.02E-08 | -0.382578171 | 0.839 | 0.143 | 0.000140496 | FIB |
| AC114316.1 | 7.52E-08 | 0.303594033 | 0.927 | 0.26 | 0.000150312 | FIB |
| ALDH1A1 | 7.55E-08 | -0.426985802 | 0.927 | 0.234 | 0.000150919 | FIB |
| NTRK3 | 8.44E-08 | 0.503357453 | 0.306 | 0.182 | 0.000168831 | FIB |
| MAGI2-AS3 | 8.85E-08 | -0.542675036 | 0.363 | 0.299 | 0.000177015 | FIB |
| NR4A3 | 1.03E-07 | 0.485237652 | 0.306 | 0.182 | 0.000205315 | FIB |
| RUNX2 | 1.04E-07 | 0.787252508 | 0.282 | 0.052 | 0.000208068 | FIB |
| CACNB2 | 1.05E-07 | -0.438115161 | 0.935 | 0.247 | 0.000209103 | FIB |
| TRPC4 | 1.05E-07 | 0.883865929 | 0.29 | 0.065 | 0.000209318 | FIB |
| SMAD3 | 1.21E-07 | -0.304703015 | 0.355 | 0.273 | 0.000242068 | FIB |
| PAPSS2 | 1.38E-07 | -0.341200424 | 0.339 | 0.195 | 0.000276795 | FIB |
| UAP1 | 1.62E-07 | -0.908364185 | 0.992 | 0.286 | 0.000323868 | FIB |
| PALMD | 1.89E-07 | -1.007287349 | 0.944 | 0.247 | 0.000377557 | FIB |
| BTG2 | 1.93E-07 | -1.032861727 | 0.992 | 0.286 | 0.00038657 | FIB |
| LSAMP | 1.96E-07 | 0.510678917 | 0.331 | 0.286 | 0.000391852 | FIB |
| SLC7A8 | 2.02E-07 | -0.543123852 | 0.831 | 0.143 | 0.000403935 | FIB |
| ANO3 | 2.30E-07 | 0.274293293 | 0.798 | 0.117 | 0.000459736 | FIB |
| EBF2 | 2.51E-07 | -1.729559708 | 0.992 | 0.286 | 0.000501842 | FIB |
| MOB1B | 2.90E-07 | -0.294644491 | 0.331 | 0.156 | 0.000580063 | FIB |
| SLC9C1 | 2.91E-07 | -0.4737489 | 0.331 | 0.143 | 0.00058274 | FIB |
| IER3 | 2.92E-07 | 0.250651056 | 0.29 | 0.013 | 0.000583471 | FIB |
| CLIC5 | 3.01E-07 | -0.846501358 | 0.984 | 0.286 | 0.000602982 | FIB |
| GPC5 | 3.04E-07 | -0.442135487 | 0.895 | 0.221 | 0.000608265 | FIB |
| PPP2R2B | 3.32E-07 | 0.3506175 | 0.298 | 0.026 | 0.00066389 | FIB |
| LINC01482 | 4.04E-07 | -0.779191726 | 0.976 | 0.286 | 0.000807989 | FIB |
| PDLIM3 | 4.51E-07 | -0.756025995 | 0.331 | 0.156 | 0.000901978 | FIB |
| LINC01828 | 4.60E-07 | -0.76290942 | 0.96 | 0.273 | 0.000920073 | FIB |
| NTN1 | 4.61E-07 | -1.157278817 | 0.992 | 0.286 | 0.000922044 | FIB |
| NID2 | 6.24E-07 | -0.303142119 | 0.976 | 0.299 | 0.001248075 | FIB |
| LINC00278 | 6.82E-07 | 0.651186749 | 0.298 | 0.013 | 0.001364125 | FIB |
| SLC7A2 | 9.49E-07 | 0.583402282 | 0.339 | 0.247 | 0.001897053 | FIB |
| HSPB1 | 9.49E-07 | -0.509094419 | 0.331 | 0.13 | 0.001897467 | FIB |
| HGF | 1.02E-06 | 0.473325755 | 0.298 | 0.052 | 0.002035434 | FIB |
| PDE4B | 1.36E-06 | 0.432047181 | 0.315 | 0.052 | 0.002728136 | FIB |
| CTTNBP2 | 1.37E-06 | -0.571638334 | 1 | 0.312 | 0.002732183 | FIB |
| PLPP3 | 1.51E-06 | -0.415542013 | 0.952 | 0.286 | 0.00301868 | FIB |
| H3-3B | 1.61E-06 | -0.514946286 | 0.823 | 0.156 | 0.003215039 | FIB |
| AC024901.1 | 1.70E-06 | 0.789910139 | 0.315 | 0.052 | 0.00339047 | FIB |
| INSIG1 | 1.78E-06 | -0.404069813 | 0.282 | 0.039 | 0.003557516 | FIB |
| NDRG1 | 1.82E-06 | -0.727275625 | 0.387 | 0.312 | 0.003648368 | FIB |
| KIRREL1 | 2.14E-06 | 0.297988694 | 0.839 | 0.208 | 0.004277003 | FIB |
| PXDN | 2.31E-06 | 0.411708532 | 0.315 | 0.104 | 0.00461605 | FIB |
| SORCS1 | 3.29E-06 | 0.749862724 | 0.323 | 0.065 | 0.006586979 | FIB |
| STAT4 | 3.67E-06 | 0.563369516 | 0.315 | 0.026 | 0.007343251 | FIB |
| RANBP3L | 4.43E-06 | -0.257985358 | 0.726 | 0.052 | 0.008869624 | FIB |
| NUAK1 | 4.72E-06 | 0.590468238 | 0.806 | 0.182 | 0.009431144 | FIB |
| ADAMTS6 | 4.98E-06 | -1.422716989 | 1 | 0.312 | 0.009963826 | FIB |
| SLC14A2 | 5.11E-06 | 0.263537576 | 0.347 | 0.143 | 0.010228279 | FIB |
| LINC02884 | 5.34E-06 | -0.567094792 | 0.419 | 0.338 | 0.010685969 | FIB |
| P3H2 | 5.98E-06 | 0.42463097 | 0.347 | 0.143 | 0.011963467 | FIB |
| MEF2C | 6.42E-06 | 0.302333971 | 0.347 | 0.169 | 0.012836609 | FIB |
| SYT10 | 6.62E-06 | -0.445335975 | 0.734 | 0.078 | 0.013241471 | FIB |
| LDHA | 7.31E-06 | -0.754009276 | 0.347 | 0.091 | 0.014614534 | FIB |
| PDE1C | 7.38E-06 | 0.376938221 | 0.355 | 0.195 | 0.014753893 | FIB |
| NFATC2 | 7.69E-06 | -0.334361063 | 0.79 | 0.143 | 0.015375515 | FIB |
| CD9 | 8.63E-06 | -0.824057846 | 0.75 | 0.091 | 0.017265958 | FIB |
| ADAMTS1 | 9.38E-06 | 0.779503456 | 0.339 | 0.091 | 0.018758337 | FIB |
| NPSR1-AS1 | 9.38E-06 | -0.337650163 | 0.347 | 0.091 | 0.018758337 | FIB |
| ACTG1 | 9.95E-06 | 0.466816414 | 0.339 | 0.091 | 0.019898942 | FIB |
| MIR646HG | 1.01E-05 | -0.388226051 | 0.363 | 0.143 | 0.020175242 | FIB |
| GRIN2A | 1.11E-05 | 1.85677395 | 0.323 | 0.026 | 0.02218201 | FIB |
| NR4A2 | 1.16E-05 | -0.48545219 | 0.379 | 0.234 | 0.023224273 | FIB |
| COL23A1 | 1.48E-05 | 0.364959578 | 0.347 | 0.091 | 0.029604468 | FIB |
| GRK5 | 1.63E-05 | -1.001617406 | 0.532 | 0.519 | 0.032653181 | FIB |
| STIM2 | 1.69E-05 | -0.526065453 | 0.395 | 0.208 | 0.033823279 | FIB |
| FHL1 | 1.79E-05 | -0.583490684 | 0.984 | 0.338 | 0.035848774 | FIB |
