# Supplementary material for: Investigating EGF and PAG1 as necroptosis-related biomarkers for diabetic nephropathy: an in silico and in vitro validation study
Source: Aging (Albany NY). 2023 Nov 20;15(22):13176–93. doi: 10.18632/aging.205233 (PMC10713428; doi:10.18632/aging.205233)
Supplement: Supplementary Table 3 [file aging-15-205233-s003.docx]

**Supplementary Table 3. Necroptosis related gene modules obtained from weighted gene co-expression network analysis (bule, lightyellow and lightcyan modules).**

| **probes** | **moduleColor** | **GS.NECROPTOSIS** | **p.GS.NECROPTOSIS** |
| --- | --- | --- | --- |
| A1BG | blue | -0.067235999 | 0.668367666 |
| A2M | lightcyan | 0.278685879 | 0.07034926 |
| AAAS | blue | -0.098909494 | 0.528020182 |
| AADACL3 | blue | -0.101800891 | 0.515968005 |
| AADAT | blue | -0.094457346 | 0.546844059 |
| AATK | blue | -0.178773624 | 0.251372384 |
| ABCA1 | blue | -0.033661872 | 0.830319471 |
| ABCA12 | blue | 0.096863182 | 0.536632452 |
| ABCA4 | blue | -0.140045696 | 0.370410023 |
| ABCA5 | blue | -0.084148914 | 0.591616857 |
| ABCA7 | blue | 0.218940575 | 0.158373215 |
| ABCB11 | blue | 0.004324721 | 0.978042172 |
| ABCB5 | blue | 0.122456817 | 0.434043086 |
| ABCB6 | blue | -0.233650472 | 0.131564622 |
| ABCB7 | blue | -0.0547884 | 0.727128983 |
| ABCC10 | blue | 0.346485651 | 0.022838666 |
| ABCC11 | blue | 0.020363224 | 0.89687565 |
| ABCF1 | blue | 0.09439347 | 0.547116442 |
| ABCF3 | blue | -0.074420906 | 0.6352939 |
| ABCG2 | blue | -0.053934008 | 0.731224203 |
| ABHD1 | blue | -0.274313741 | 0.075057586 |
| ABHD13 | blue | 0.171604864 | 0.271194315 |
| ABHD14A | blue | -0.4267757 | 0.004317349 |
| ABHD17B | blue | -0.141965859 | 0.363819133 |
| ABHD2 | blue | 0.240229245 | 0.120736298 |
| ABHD5 | blue | 0.181892676 | 0.243059974 |
| ABI1 | blue | -0.04294242 | 0.784529861 |
| ABL1 | blue | -0.313646478 | 0.040548899 |
| ABL2 | blue | 0.190520219 | 0.221043772 |
| ABO | blue | 0.057394189 | 0.714685771 |
| ACAP1 | blue | 0.469126347 | 0.001506905 |
| ACAP2 | blue | 0.43938969 | 0.003199195 |
| ACBD4 | blue | -0.243762043 | 0.115205922 |
| ACBD5 | blue | -0.32519807 | 0.03335007 |
| ACBD7 | blue | -0.173996988 | 0.264468609 |
| ACOT9 | blue | 0.344544382 | 0.023664994 |
| ACOX3 | blue | 0.160994586 | 0.302376566 |
| ACP2 | blue | -0.000156455 | 0.999205539 |
| ACP6 | blue | -0.285471889 | 0.063506836 |
| ACR | blue | 0.174387078 | 0.263382424 |
| ACSL4 | blue | 0.223685007 | 0.14932449 |
| ACSL5 | blue | 0.22242013 | 0.151698871 |
| ACSL6 | blue | -0.156341441 | 0.316747835 |
| ACSM1 | blue | -0.003715778 | 0.981133348 |
| ACTBL2 | lightcyan | -0.498412468 | 0.000670281 |
| ACTR1A | blue | 0.23139759 | 0.135434528 |
| ACTR3B | blue | -0.119261289 | 0.44622201 |
| ACTR8 | blue | 0.059073455 | 0.706705196 |
| ACTRT1 | lightyellow | 0.127666906 | 0.41458862 |
| ACTRT3 | blue | 0.081441352 | 0.603639647 |
| ACVR1C | blue | -0.320192966 | 0.036328017 |
| ACVR2B | lightcyan | -0.435569993 | 0.003507328 |
| ACYP1 | blue | 0.075602809 | 0.629917657 |
| ADAL | blue | -0.247665533 | 0.109320678 |
| ADAM11 | blue | -0.079975829 | 0.610190996 |
| ADAM12 | blue | 0.08783991 | 0.575400205 |
| ADAM17 | lightcyan | 0.378374285 | 0.012354849 |
| ADAM20 | blue | 0.032795541 | 0.834624852 |
| ADAM21 | blue | 0.385463942 | 0.010690191 |
| ADAM32 | blue | -0.199294176 | 0.2001066 |
| ADAM33 | blue | 0.082357033 | 0.599561784 |
| ADAM7 | blue | -0.091893586 | 0.557826897 |
| ADAM8 | blue | -0.082340468 | 0.599635449 |
| ADAM9 | blue | 0.336535946 | 0.027342544 |
| ADAMDEC1 | blue | 0.094232945 | 0.547801233 |
| ADAMTS10 | blue | 0.15081716 | 0.334360104 |
| ADAMTS2 | blue | 0.041310406 | 0.792534913 |
| ADAMTS4 | blue | -0.078421287 | 0.617173299 |
| ADAMTS8 | blue | -0.021701538 | 0.890138227 |
| ADAR | lightyellow | 0.303631055 | 0.047773496 |
| ADARB2 | blue | 0.149418896 | 0.338912533 |
| ADCK1 | blue | -0.196146743 | 0.207450513 |
| ADCK5 | blue | -0.299534918 | 0.051013413 |
| ADCY3 | blue | 0.392757573 | 0.009181803 |
| ADCY4 | lightcyan | 0.502035466 | 0.000603284 |
| ADCY6 | blue | 0.267430765 | 0.08296779 |
| ADCY7 | blue | 0.261326008 | 0.090514678 |
| ADCYAP1 | blue | -0.21375374 | 0.168715998 |
| ADCYAP1R1 | blue | -0.322239048 | 0.035085296 |
| ADD1 | blue | 0.316438504 | 0.03870223 |
| ADD2 | blue | -0.031249012 | 0.842322395 |
| ADGB | blue | 0.054485178 | 0.728581517 |
| ADH5 | blue | -0.347351989 | 0.022477762 |
| ADHFE1 | blue | -0.016122814 | 0.918268101 |
| ADK | blue | -0.458845622 | 0.001969799 |
| ADNP | blue | 0.017882445 | 0.909383149 |
| ADNP2 | blue | -0.034979322 | 0.823781611 |
| ADORA2B | blue | -0.416317034 | 0.005489257 |
| ADORA3 | blue | 0.118056459 | 0.450862061 |
| ADPGK | lightcyan | 0.390237223 | 0.009680917 |
| ADPRHL1 | blue | 0.080940589 | 0.605874791 |
| ADRA1A | blue | 0.00880848 | 0.955293968 |
| ADRA1B | blue | -0.227919555 | 0.141574262 |
| ADRB2 | blue | 0.31098099 | 0.042378363 |
| ADRB3 | blue | 0.176897612 | 0.256462943 |
| ADSL | blue | 0.203191799 | 0.191268102 |
| AEN | blue | 0.300442721 | 0.050280509 |
| AFAP1L1 | lightcyan | 0.438422751 | 0.003274863 |
| AFF1 | blue | -0.191281226 | 0.219170151 |
| AFF2 | lightcyan | -0.276387285 | 0.072794629 |
| AGA | blue | -0.22683046 | 0.14353852 |
| AGAP1 | blue | -0.138357247 | 0.376264144 |
| AGAP3 | blue | -0.025288241 | 0.872120731 |
| AGBL4 | blue | -0.447558234 | 0.002618775 |
| AGBL5 | blue | -0.123005547 | 0.431970524 |
| AGER | blue | 0.292005923 | 0.05742932 |
| AGFG1 | blue | 0.217892765 | 0.160424399 |
| AGK | blue | -0.196916354 | 0.205637633 |
| AGL | blue | -0.062411857 | 0.690932307 |
| AGPAT1 | blue | -0.151325194 | 0.332715508 |
| AGPAT2 | blue | -0.127617114 | 0.414772159 |
| AGPAT4 | blue | 0.317074959 | 0.038291052 |
| AGR3 | blue | 0.201259746 | 0.19561413 |
| AHI1 | blue | 0.220391862 | 0.155563863 |
| AHSA1 | blue | -0.067286919 | 0.668130981 |
| AIMP2 | blue | -0.054558196 | 0.728231647 |
| AJUBA | lightcyan | -0.301487368 | 0.049447664 |
| AK3 | blue | -0.000280664 | 0.998574817 |
| AK8 | blue | 0.020902297 | 0.894160928 |
| AKAP1 | blue | -0.183267178 | 0.23945661 |
| AKAP10 | blue | 0.157091994 | 0.31440104 |
| AKAP12 | blue | -0.147727321 | 0.344470834 |
| AKAP13 | blue | 0.106871718 | 0.49516839 |
| AKAP14 | blue | -0.049050607 | 0.754769653 |
| AKAP17A | blue | -0.040878734 | 0.794655828 |
| AKAP3 | blue | -0.259044656 | 0.093467597 |
| AKAP8L | blue | -0.032908819 | 0.834061623 |
| AKNA | blue | 0.311261573 | 0.042182675 |
| AKR1D1 | blue | 0.090918205 | 0.562032265 |
| AKR1E2 | blue | 0.126917931 | 0.417354319 |
| AKT1S1 | blue | -0.08248446 | 0.59899526 |
| AKTIP | blue | -0.007196535 | 0.963469014 |
| ALAS1 | blue | 0.071575033 | 0.648314974 |
| ALCAM | blue | -0.41837107 | 0.00523944 |
| ALDH1A3 | blue | -0.115427575 | 0.461077136 |
| ALDH1L2 | blue | 0.004585822 | 0.976716841 |
| ALDH7A1 | blue | -0.283313896 | 0.065622792 |
| ALG10 | blue | -0.117739616 | 0.452086644 |
| ALG12 | blue | -0.355686707 | 0.01924304 |
| ALG1L2 | blue | 0.03957432 | 0.801073588 |
| ALG9 | blue | 0.083488398 | 0.594540063 |
| ALK | blue | 0.040404907 | 0.796985549 |
| ALKBH2 | blue | -0.294688057 | 0.055073495 |
| ALKBH3 | blue | 0.111942273 | 0.474809786 |
| ALKBH4 | blue | -0.382379111 | 0.011389283 |
| ALOX15 | blue | -0.08204871 | 0.600933518 |
| ALOX15B | blue | -0.001463002 | 0.992571126 |
| ALOXE3 | blue | 0.084246438 | 0.591185789 |
| ALPK1 | blue | 0.051047375 | 0.745114276 |
| ALPP | blue | -0.024622291 | 0.875461523 |
| ALS2CL | blue | 0.092956843 | 0.553259514 |
| AMBP | blue | -0.036435345 | 0.816569766 |
| AMBRA1 | blue | 0.172001636 | 0.270071018 |
| AMD1 | blue | 0.02003703 | 0.898518902 |
| AMELY | blue | -0.143270394 | 0.35938192 |
| AMER1 | blue | -0.147271491 | 0.345978159 |
| AMER2 | blue | -0.011252425 | 0.942908309 |
| AMER3 | blue | -0.178300994 | 0.252648422 |
| AMHR2 | blue | -0.038928501 | 0.804255861 |
| AMOTL1 | blue | 0.396461246 | 0.00848853 |
| AMPD3 | blue | -0.311771831 | 0.04182869 |
| AMTN | blue | -0.061274221 | 0.696293178 |
| AMZ1 | blue | 0.06680325 | 0.670380445 |
| AMZ2 | blue | -0.292963015 | 0.056579582 |
| ANAPC1 | blue | 0.105472808 | 0.500863033 |
| ANAPC10 | blue | 0.028721709 | 0.854932513 |
| ANAPC13 | blue | 0.014421051 | 0.926870226 |
| ANAPC15 | blue | -0.187765955 | 0.227917137 |
| ANGPT1 | blue | 0.016660638 | 0.915551357 |
| ANHX | blue | 0.196281315 | 0.207132714 |
| ANKDD1A | blue | 0.2177987 | 0.16060948 |
| ANKFY1 | blue | -0.027136219 | 0.862861862 |
| ANKH | blue | 0.057543626 | 0.713974349 |
| ANKK1 | blue | 0.068859533 | 0.660836913 |
| ANKLE2 | blue | 0.387075774 | 0.010339756 |
| ANKRD12 | blue | -0.212456306 | 0.171377742 |
| ANKRD13A | blue | 0.055477308 | 0.723832401 |
| ANKRD13B | blue | -0.163174184 | 0.295790886 |
| ANKRD16 | blue | 0.180611416 | 0.246451781 |
| ANKRD23 | blue | -0.053555937 | 0.733038713 |
| ANKRD34C | blue | -0.134446106 | 0.390034197 |
| ANKRD35 | blue | 0.081900974 | 0.60159128 |
| ANKRD39 | blue | 0.020718873 | 0.895084501 |
| ANKRD40 | blue | 0.069149705 | 0.6594944 |
| ANKRD42 | blue | -0.172690719 | 0.268127469 |
| ANKRD45 | blue | -0.011277553 | 0.942781024 |
| ANKRD46 | blue | 0.067804726 | 0.665725922 |
| ANKRD49 | blue | 0.131357465 | 0.401113946 |
| ANKRD52 | blue | -0.225163643 | 0.146583601 |
| ANKRD53 | blue | 0.086986329 | 0.579132446 |
| ANKRD55 | blue | -0.024617017 | 0.875487987 |
| ANKRD62 | blue | 0.008727685 | 0.955703623 |
| ANKRD63 | blue | 0.068757956 | 0.661307117 |
| ANKRD66 | blue | -0.114375315 | 0.465200529 |
| ANKS3 | lightcyan | -0.172285774 | 0.269268486 |
| ANKUB1 | blue | 0.344041882 | 0.023882912 |
| ANO2 | blue | -0.043118979 | 0.783665135 |
| ANO3 | blue | -0.138945364 | 0.374218837 |
| ANO6 | blue | 0.133981511 | 0.391689269 |
| ANO7 | blue | -0.13910666 | 0.373659057 |
| ANP32D | blue | 0.18817844 | 0.22687854 |
| ANTXR1 | blue | 0.17119123 | 0.272368629 |
| ANTXR2 | blue | 0.277987036 | 0.071085761 |
| ANXA2R | blue | 0.10625261 | 0.497684519 |
| ANXA6 | blue | 0.141736934 | 0.364601177 |
| AOC3 | blue | 0.216632172 | 0.162917657 |
| AP1B1 | blue | -0.273426277 | 0.076042858 |
| AP1M2 | blue | -0.376956975 | 0.012712884 |
| AP1S1 | blue | 0.131467485 | 0.400716181 |
| AP1S2 | blue | 0.234785076 | 0.129647179 |
| AP3B2 | blue | -0.07815189 | 0.618386724 |
| AP3S2 | blue | -0.250193962 | 0.105632567 |
| AP4E1 | blue | 0.333748397 | 0.028729068 |
| AP4M1 | blue | -0.072436753 | 0.644361069 |
| AP4S1 | blue | -0.026337518 | 0.866861416 |
| AP5M1 | blue | -0.001849438 | 0.990608948 |
| APAF1 | blue | 0.155560028 | 0.319202849 |
| APBA3 | blue | -0.00239022 | 0.987863153 |
| APBB1 | blue | -0.225288348 | 0.146354147 |
| APBB3 | blue | 0.316461858 | 0.038687079 |
| APEX1 | blue | -0.07693812 | 0.623866232 |
| APOA1 | blue | -0.021659313 | 0.890350687 |
| APOA5 | blue | 0.164483746 | 0.291878843 |
| APOBEC1 | blue | -0.089189597 | 0.569521204 |
| APOBEC3H | blue | 0.150987023 | 0.333809665 |
| APOL4 | blue | 0.502401078 | 0.000596868 |
| APOL6 | lightyellow | 0.147982167 | 0.343629879 |
| APOO | blue | -0.316073399 | 0.03893973 |
| APPL2 | blue | -0.071126758 | 0.650375629 |
| APRT | blue | -0.195399489 | 0.209221381 |
| AQP12A | blue | -0.172493937 | 0.268681542 |
| AQP5 | blue | -0.126326027 | 0.419547402 |
| AQP6 | blue | -0.336139585 | 0.027536236 |
| AQP8 | blue | 0.057634608 | 0.713541331 |
| ARC | blue | 0.012284616 | 0.937681092 |
| AREG | lightyellow | 0.349648691 | 0.021544016 |
| ARFGEF1 | blue | 0.065023773 | 0.678680886 |
| ARFGEF2 | blue | -0.122822755 | 0.432660318 |
| ARFIP2 | blue | -0.068555365 | 0.662245307 |
| ARG1 | blue | -0.020901375 | 0.894165572 |
| ARHGAP10 | blue | -0.073174506 | 0.640983646 |
| ARHGAP11B | blue | 0.22782435 | 0.141745174 |
| ARHGAP15 | blue | 0.284568667 | 0.064385762 |
| ARHGAP17 | blue | 0.323390697 | 0.034401331 |
| ARHGAP24 | blue | -0.510664171 | 0.000467236 |
| ARHGAP27 | blue | 0.235961267 | 0.127681587 |
| ARHGAP31 | lightcyan | 0.541695131 | 0.000175824 |
| ARHGAP35 | blue | -0.353136431 | 0.020188338 |
| ARHGAP40 | blue | -0.025650882 | 0.870302427 |
| ARHGAP42 | blue | -0.054759772 | 0.727266081 |
| ARHGDIG | blue | -0.19022293 | 0.2217787 |
| ARHGEF10L | blue | 0.052506477 | 0.738082996 |
| ARHGEF15 | blue | 0.468764263 | 0.001521404 |
| ARHGEF19 | blue | -0.233883751 | 0.13116867 |
| ARHGEF2 | blue | 0.35823485 | 0.018336006 |
| ARHGEF25 | blue | 0.166884907 | 0.284793237 |
| ARHGEF33 | blue | -0.038439787 | 0.806666076 |
| ARHGEF6 | blue | 0.292509095 | 0.056981323 |
| ARHGEF9 | blue | -0.170592267 | 0.274075025 |
| ARID1B | blue | -0.057974278 | 0.711925497 |
| ARID3B | blue | 0.022926383 | 0.883978716 |
| ARID3C | blue | 0.271992243 | 0.077656374 |
| ARID4A | blue | 0.187883248 | 0.227621473 |
| ARID4B | blue | 0.143254048 | 0.359437317 |
| ARID5A | blue | 0.216303513 | 0.163572294 |
| ARL13B | blue | -0.130637408 | 0.40372288 |
| ARL14 | lightcyan | -0.025751164 | 0.869799726 |
| ARL16 | blue | -0.282281487 | 0.066654686 |
| ARL17A | blue | 0.114942778 | 0.462974415 |
| ARL17B | blue | 0.036916949 | 0.814187587 |
| ARL4A | blue | 0.014005958 | 0.928969735 |
| ARL6IP4 | blue | -0.325111747 | 0.033399671 |
| ARL6IP6 | blue | 0.024438219 | 0.876385317 |
| ARL9 | blue | 0.084810803 | 0.588693969 |
| ARMC12 | blue | -0.011001987 | 0.944176942 |
| ARMC2 | blue | 0.097673823 | 0.533212602 |
| ARMC8 | blue | 0.256685801 | 0.096598516 |
| ARPC1B | blue | 0.286471319 | 0.062545441 |
| ARPC2 | lightcyan | 0.23794826 | 0.124411842 |
| ARRDC5 | blue | -0.100806653 | 0.52009672 |
| ARSH | blue | -0.136511438 | 0.38272635 |
| ART4 | blue | -0.31385531 | 0.040408333 |
| ARTN | blue | -0.032579918 | 0.835697176 |
| ASAH2B | blue | -0.233689025 | 0.131499125 |
| ASAP1 | lightcyan | 0.517617385 | 0.000378391 |
| ASAP3 | blue | -0.225593349 | 0.145794066 |
| ASB10 | blue | 0.146300828 | 0.349201373 |
| ASB14 | blue | 0.185761856 | 0.233009664 |
| ASB3 | blue | 0.197269153 | 0.204810303 |
| ASB5 | blue | -0.129606933 | 0.407473498 |
| ASB8 | blue | -0.251962226 | 0.103110286 |
| ASCC1 | blue | 0.060149474 | 0.701607716 |
| ASCC2 | blue | -0.070137135 | 0.654933875 |
| ASCL1 | blue | -0.207203397 | 0.182463724 |
| ASF1B | blue | 0.160071445 | 0.305193938 |
| ASGR1 | blue | 0.021661073 | 0.890341832 |
| ASIC2 | blue | -0.01487823 | 0.924558412 |
| ASIP | blue | -0.199572119 | 0.199466975 |
| ASNSD1 | blue | -0.231568656 | 0.135137741 |
| ASPH | blue | -0.360983056 | 0.017398408 |
| ASPSCR1 | blue | -0.189623572 | 0.223265493 |
| ASTE1 | blue | 0.171643611 | 0.271084483 |
| ASTN2 | blue | -0.502706883 | 0.000591548 |
| ATAD2 | blue | 0.142945528 | 0.360483837 |
| ATAT1 | blue | -0.047382301 | 0.76286521 |
| ATCAY | blue | 0.060011103 | 0.702262509 |
| ATE1 | blue | 0.075647428 | 0.629715059 |
| ATF7 | blue | 0.097058548 | 0.535807291 |
| ATF7IP | blue | 0.272540845 | 0.077035967 |
| ATF7IP2 | blue | 0.051601849 | 0.742439871 |
| ATG13 | blue | -0.201509312 | 0.195048863 |
| ATG2B | blue | 0.321381602 | 0.035601759 |
| ATG4D | blue | -0.202437549 | 0.192956526 |
| ATG9B | blue | 0.282590601 | 0.066344386 |
| ATMIN | blue | -0.126006418 | 0.420734302 |
| ATOH1 | blue | 0.009774394 | 0.950397416 |
| ATP11A | blue | 0.273202878 | 0.076292471 |
| ATP11B | blue | -0.059055497 | 0.706790378 |
| ATP11C | blue | 0.050933 | 0.745666312 |
| ATP13A4 | lightyellow | 0.271118533 | 0.07865252 |
| ATP1A2 | blue | -0.183899134 | 0.237812123 |
| ATP1A3 | blue | -0.170411913 | 0.274590215 |
| ATP1B3 | blue | 0.477733723 | 0.00119643 |
| ATP2A3 | blue | 0.221538947 | 0.153369278 |
| ATP2B1 | blue | 0.065569241 | 0.676132479 |
| ATP2B3 | blue | 0.018665087 | 0.905434775 |
| ATP2C2 | blue | -0.237024821 | 0.12592352 |
| ATP4B | blue | -0.096642252 | 0.537566328 |
| ATP6V0A1 | blue | 0.13704641 | 0.380846701 |
| ATP6V0A2 | blue | 0.38707798 | 0.010339283 |
| ATP6V0D1 | blue | -0.120442603 | 0.441698065 |
| ATP7B | blue | -0.150048976 | 0.336856418 |
| ATP8B3 | blue | -0.258284719 | 0.094467586 |
| ATP8B4 | blue | 0.083654564 | 0.593804074 |
| ATP9B | blue | 0.285586834 | 0.063395671 |
| ATPAF2 | blue | -0.272666914 | 0.076893949 |
| ATXN1 | blue | 0.219610971 | 0.157070924 |
| ATXN1L | blue | -0.149362559 | 0.339096753 |
| ATXN7 | blue | 0.178153108 | 0.253048584 |
| ATXN7L2 | blue | 0.09001912 | 0.565921696 |
| AUNIP | blue | -0.347343058 | 0.022481458 |
| AURKAIP1 | blue | -0.353015418 | 0.020234145 |
| AURKC | blue | 0.129319528 | 0.408523119 |
| AVEN | blue | -0.135768278 | 0.385346545 |
| AVP | blue | 0.12707292 | 0.416781145 |
| AVPR1B | blue | -0.006339965 | 0.967814706 |
| AVPR2 | blue | 0.078306057 | 0.6176922 |
| AWAT1 | blue | -0.06424324 | 0.68233366 |
| AXDND1 | blue | 0.342336095 | 0.024635194 |
| AZU1 | blue | -0.036986363 | 0.813844375 |
| B3GALNT2 | blue | 0.189885692 | 0.222614425 |
| B3GALT5 | blue | -0.029501727 | 0.851036614 |
| B3GALT6 | blue | 0.170105145 | 0.275467971 |
| B3GNT4 | blue | -0.180187591 | 0.247580744 |
| B3GNT5 | blue | 0.007713104 | 0.960848774 |
| B3GNT8 | blue | 0.249208876 | 0.107057999 |
| B3GNTL1 | lightcyan | -0.235089449 | 0.129136371 |
| B4GALNT1 | blue | 0.182839851 | 0.240572974 |
| B4GALNT2 | blue | -0.354078298 | 0.019834777 |
| B4GALNT4 | blue | -0.157726057 | 0.312427082 |
| B4GALT7 | blue | -0.080095012 | 0.609657076 |
| BAALC | blue | 0.042224587 | 0.788048191 |
| BAG1 | blue | -0.382897085 | 0.01126924 |
| BAG4 | blue | -0.248578547 | 0.107977763 |
| BAHCC1 | blue | -0.022439795 | 0.886424885 |
| BAHD1 | blue | 0.215447651 | 0.165285989 |
| BAIAP3 | lightcyan | -0.48433559 | 0.000998288 |
| BAMBI | blue | 0.159347781 | 0.307414202 |
| BANF2 | blue | -0.071960964 | 0.646542984 |
| BARHL2 | blue | -0.142599081 | 0.361661202 |
| BARX1 | blue | -0.288317688 | 0.06079984 |
| BATF2 | lightyellow | 0.032665848 | 0.8352698 |
| BATF3 | blue | 0.394027597 | 0.008938786 |
| BAZ1B | blue | 0.242045647 | 0.117868374 |
| BAZ2A | blue | 0.275452791 | 0.073807739 |
| BBC3 | blue | -0.152782023 | 0.328027442 |
| BBS10 | blue | -0.244511198 | 0.114058223 |
| BBS2 | blue | -0.059188652 | 0.706158858 |
| BCAN | blue | 0.147462617 | 0.345345659 |
| BCAT2 | blue | -0.354548065 | 0.019660388 |
| BCDIN3D | blue | -0.227465009 | 0.142391636 |
| BCKDHA | blue | -0.359329834 | 0.017957453 |
| BCKDK | blue | -0.229454627 | 0.138839519 |
| BCL2L1 | blue | -0.107558704 | 0.49238407 |
| BCL2L10 | blue | -0.23233216 | 0.133819016 |
| BCO2 | blue | -0.162701227 | 0.297212016 |
| BCOR | lightcyan | 0.368916966 | 0.014915604 |
| BDKRB1 | blue | -0.261048118 | 0.090870457 |
| BDNF | blue | 0.143989091 | 0.356951415 |
| BDP1 | blue | 0.367305922 | 0.01539386 |
| BEAN1 | blue | 0.021395095 | 0.891680285 |
| BECN1 | blue | 0.020437294 | 0.896502573 |
| BEND3 | blue | -0.114414166 | 0.465047939 |
| BEND4 | blue | -0.294275203 | 0.055430984 |
| BEST1 | blue | -0.039114149 | 0.803340758 |
| BEX2 | blue | -0.335602276 | 0.027800623 |
| BFSP2 | blue | 0.135310575 | 0.386965526 |
| BHLHA15 | blue | 0.020886901 | 0.894238443 |
| BHLHA9 | blue | -0.119916289 | 0.4437105 |
| BHLHE23 | blue | 0.033328479 | 0.831975754 |
| BHLHE40 | blue | 0.158599383 | 0.309721153 |
| BICD2 | blue | 0.091606089 | 0.559064911 |
| BIRC5 | blue | -0.002408325 | 0.987771229 |
| BIRC7 | blue | 0.064704938 | 0.680172115 |
| BLMH | blue | -0.039752853 | 0.800194427 |
| BMP15 | blue | -0.151509879 | 0.332118899 |
| BMP2 | blue | 0.146825023 | 0.347458439 |
| BMP2K | blue | -0.149138697 | 0.339829379 |
| BMP6 | blue | 0.063841333 | 0.68421733 |
| BMP8B | blue | 0.042138653 | 0.788469664 |
| BMPER | blue | 0.242681729 | 0.116876324 |
| BOC | blue | 0.434935858 | 0.003560924 |
| BOD1L2 | blue | -0.067526231 | 0.667019039 |
| BOLA1 | blue | -0.488486359 | 0.000889221 |
| BOLA2 | blue | -0.024361326 | 0.876771262 |
| BOLL | blue | 0.002989213 | 0.98482196 |
| BPGM | blue | -0.292725731 | 0.056789306 |
| BPI | blue | -0.221916857 | 0.152651255 |
| BPIFB2 | blue | 0.124028373 | 0.428122063 |
| BPIFB3 | blue | 0.080308155 | 0.608702733 |
| BPIFB4 | blue | -0.157670365 | 0.312600147 |
| BPNT1 | blue | -0.045997506 | 0.769604075 |
| BRAF | blue | -0.013087531 | 0.933616749 |
| BRCA1 | blue | 0.107619128 | 0.492139564 |
| BRCC3 | blue | -0.122069278 | 0.435510158 |
| BRD2 | blue | 0.317620873 | 0.037941221 |
| BRD9 | blue | -0.20559756 | 0.185952702 |
| BRDT | blue | -0.101851019 | 0.515760273 |
| BRF2 | blue | -0.006056095 | 0.969255089 |
| BRI3BP | blue | -0.42926475 | 0.004073006 |
| BRMS1 | blue | -0.26363692 | 0.087597691 |
| BRMS1L | blue | 0.04514427 | 0.773764591 |
| BRPF1 | lightcyan | 0.020964817 | 0.893846162 |
| BRPF3 | blue | -0.33266311 | 0.029284419 |
| BRSK1 | blue | -0.098791215 | 0.528516127 |
| BRSK2 | blue | 0.069321279 | 0.658701091 |
| BSCL2 | blue | -0.280036722 | 0.068942719 |
| BSG | blue | -0.364542803 | 0.016244378 |
| BSX | blue | -0.061184042 | 0.696718756 |
| BTBD11 | blue | -0.339403804 | 0.025974544 |
| BTBD18 | blue | 0.171534957 | 0.271392547 |
| BTBD19 | blue | 0.223392181 | 0.149871728 |
| BTBD6 | blue | 0.19767849 | 0.203853313 |
| BTBD9 | blue | 0.141208641 | 0.366409776 |
| BTG4 | blue | -0.050210177 | 0.749157951 |
| BTN2A1 | blue | 0.284276711 | 0.064671923 |
| BTN2A2 | blue | 0.374971469 | 0.013229296 |
| BTN3A1 | lightyellow | 0.258591646 | 0.094062717 |
| BTN3A2 | lightyellow | 0.200172645 | 0.198089907 |
| BTN3A3 | lightyellow | 0.362676487 | 0.016841068 |
| BTNL9 | blue | 0.353125525 | 0.020192462 |
| BTRC | blue | 0.207378863 | 0.182085343 |
| C10orf120 | lightcyan | -0.064347423 | 0.681845685 |
| C10orf90 | blue | -0.103104052 | 0.510581343 |
| C11orf1 | blue | -0.470261694 | 0.001462235 |
| C11orf16 | blue | 0.307751521 | 0.044684385 |
| C11orf21 | blue | 0.010936949 | 0.944506424 |
| C11orf24 | lightcyan | 0.054289314 | 0.729520268 |
| C11orf53 | blue | -0.052265939 | 0.739240691 |
| C11orf58 | blue | -0.02052676 | 0.896051974 |
| C11orf65 | blue | 0.06521763 | 0.677774786 |
| C11orf68 | blue | 0.050534786 | 0.74758929 |
| C11orf71 | blue | -0.223026223 | 0.150557704 |
| C11orf86 | blue | -0.080151542 | 0.609403904 |
| C11orf94 | blue | 0.057892793 | 0.712313015 |
| C12orf29 | blue | -0.311605472 | 0.041943832 |
| C12orf40 | blue | 0.121948248 | 0.435968895 |
| C12orf54 | blue | 0.016281401 | 0.917466921 |
| C12orf65 | blue | -0.09371008 | 0.550034585 |
| C12orf73 | blue | 0.23008491 | 0.137728081 |
| C12orf76 | blue | 0.09194841 | 0.55759096 |
| C14orf28 | blue | 0.040091019 | 0.798529843 |
| C14orf93 | blue | -0.173460141 | 0.265968286 |
| C15orf39 | blue | 0.449953259 | 0.002467191 |
| C15orf40 | blue | -0.140245126 | 0.369722183 |
| C16orf54 | blue | 0.272381788 | 0.077215442 |
| C16orf70 | blue | -0.382530317 | 0.011354128 |
| C16orf71 | blue | -0.29020512 | 0.05905581 |
| C16orf78 | blue | -0.035879149 | 0.819322935 |
| C16orf82 | blue | 0.01824805 | 0.907538426 |
| C16orf86 | blue | -0.065491054 | 0.676497552 |
| C16orf90 | blue | 0.245133789 | 0.113111029 |
| C16orf91 | blue | -0.249029154 | 0.107319634 |
| C16orf95 | blue | -0.351769318 | 0.020710937 |
| C16orf96 | blue | -0.182014049 | 0.242740316 |
| C17orf50 | blue | -0.105095485 | 0.502404729 |
| C17orf67 | blue | 0.151410953 | 0.332438386 |
| C17orf99 | blue | -0.023558698 | 0.880801539 |
| C18orf54 | blue | 0.235015997 | 0.129259501 |
| C18orf63 | blue | 0.109431283 | 0.484835849 |
| C19orf12 | blue | 0.103133299 | 0.510460777 |
| C19orf47 | blue | -0.108079306 | 0.490279495 |
| C19orf67 | blue | -0.027481475 | 0.861134008 |
| C19orf71 | blue | 0.148157498 | 0.343052046 |
| C1GALT1 | blue | -0.207897659 | 0.180969873 |
| C1orf105 | blue | -0.084993734 | 0.587887284 |
| C1orf109 | blue | 0.088486815 | 0.572578954 |
| C1orf127 | blue | -0.218647747 | 0.158944519 |
| C1orf141 | blue | -0.061557265 | 0.694958014 |
| C1orf159 | blue | 0.125704219 | 0.421858289 |
| C1orf189 | lightyellow | 0.335571897 | 0.027815634 |
| C1orf194 | blue | -0.051760178 | 0.741676745 |
| C1orf21 | blue | -0.350158711 | 0.021341135 |
| C1orf216 | blue | 0.066442791 | 0.672058735 |
| C1orf226 | blue | 0.142261795 | 0.36280966 |
| C1orf54 | lightcyan | 0.328657127 | 0.031411863 |
| C1QB | blue | 0.20251419 | 0.192784482 |
| C1QL1 | blue | -0.047805652 | 0.760808469 |
| C1QL2 | blue | -0.181631572 | 0.243748602 |
| C1QL4 | blue | -0.066002507 | 0.674110822 |
| C1QTNF3 | blue | 0.431279083 | 0.003884197 |
| C1QTNF6 | blue | 0.035595569 | 0.820727486 |
| C1QTNF9B | blue | 0.023652765 | 0.88032904 |
| C20orf202 | blue | 0.175819802 | 0.25941854 |
| C2CD2 | blue | 0.243246953 | 0.116000094 |
| C2CD3 | blue | 0.249360645 | 0.106837436 |
| C2CD4A | blue | -0.101680879 | 0.516465501 |
| C2orf15 | blue | -0.082638327 | 0.598311494 |
| C2orf50 | blue | -0.060062726 | 0.702018194 |
| C2orf66 | blue | 0.101791846 | 0.516005489 |
| C2orf80 | blue | -0.154336013 | 0.323072439 |
| C2orf88 | blue | 0.087407901 | 0.577287773 |
| C3orf18 | blue | 0.033433419 | 0.831454337 |
| C3orf22 | blue | -0.201825308 | 0.194334785 |
| C3orf70 | blue | 0.200854308 | 0.196534913 |
| C3orf80 | lightyellow | 0.27797136 | 0.071102351 |
| C4orf17 | blue | -0.181382281 | 0.244407307 |
| C4orf19 | blue | -0.167426778 | 0.283209854 |
| C4orf36 | blue | 0.045162202 | 0.773677089 |
| C4orf46 | blue | 0.080007248 | 0.610050224 |
| C4orf47 | blue | 0.267345048 | 0.083070245 |
| C4orf50 | blue | -0.014898728 | 0.924454773 |
| C5 | blue | -0.237590176 | 0.124996402 |
| C5orf22 | blue | -0.359197894 | 0.018002715 |
| C5orf24 | blue | 0.076336971 | 0.626587553 |
| C6orf136 | blue | -0.238801842 | 0.12302668 |
| C6orf52 | blue | 0.140420255 | 0.369118789 |
| C7orf50 | blue | -0.289190366 | 0.059988429 |
| C8A | blue | 0.068535241 | 0.662338531 |
| C9 | blue | 0.052491922 | 0.738153031 |
| C9orf116 | blue | -0.328583836 | 0.031451944 |
| C9orf135 | blue | -0.117706832 | 0.452213455 |
| C9orf153 | blue | 0.003260744 | 0.983443415 |
| C9orf24 | blue | -0.068526682 | 0.662378178 |
| C9orf40 | blue | 0.027996477 | 0.858557841 |
| C9orf64 | blue | -0.258131162 | 0.094670647 |
| C9orf78 | blue | -0.159570709 | 0.306729143 |
| C9orf85 | blue | -0.042870019 | 0.784884532 |
| CA1 | blue | 0.091031388 | 0.56154352 |
| CA14 | blue | -0.115602314 | 0.460394309 |
| CA3 | lightcyan | -0.221268723 | 0.153884214 |
| CA5A | blue | 0.401418647 | 0.007631409 |
| CA5B | blue | 0.142939099 | 0.360505665 |
| CA6 | blue | 0.072699791 | 0.643156067 |
| CA7 | blue | -0.035752599 | 0.81994966 |
| CAB39L | blue | -0.283776025 | 0.065165021 |
| CABIN1 | blue | -0.126874313 | 0.417515708 |
| CABLES1 | blue | -0.448365165 | 0.002566812 |
| CABLES2 | blue | 0.048459838 | 0.757633477 |
| CABP4 | blue | 0.158043467 | 0.311441885 |
| CABP5 | blue | -0.071999305 | 0.64636705 |
| CABP7 | blue | -0.219521245 | 0.157244769 |
| CACHD1 | blue | -0.293434173 | 0.056164988 |
| CACNA1C | lightcyan | 0.447413134 | 0.002628216 |
| CACNA1E | blue | 0.010995615 | 0.944209219 |
| CACNA2D1 | blue | -0.183715119 | 0.238290175 |
| CACNA2D4 | blue | -0.283461484 | 0.06547632 |
| CACNB3 | blue | 0.051880817 | 0.741095445 |
| CACNG4 | blue | -0.346509815 | 0.022828534 |
| CACNG5 | blue | -0.047470265 | 0.762437725 |
| CACUL1 | blue | 0.115286072 | 0.461630484 |
| CACYBP | blue | -0.281008039 | 0.067945172 |
| CADM4 | blue | -0.248459524 | 0.108152112 |
| CADPS2 | blue | -0.076080436 | 0.627750347 |
| CAGE1 | blue | -0.043457622 | 0.782007299 |
| CALCRL | blue | 0.199926303 | 0.198653983 |
| CALD1 | blue | 0.292028323 | 0.057409317 |
| CALHM2 | blue | 0.396438448 | 0.008492655 |
| CALM1 | blue | -0.054110994 | 0.730375272 |
| CALML5 | blue | -0.126337052 | 0.419506491 |
| CALML6 | blue | -0.250701324 | 0.104904085 |
| CALY | blue | -0.10735993 | 0.493188857 |
| CAMK1 | blue | -0.002163065 | 0.989016508 |
| CAMK1D | blue | 0.340040332 | 0.025678769 |
| CAMK2B | blue | 0.014996796 | 0.923958963 |
| CAP2 | blue | 0.103623597 | 0.508441709 |
| CAPN14 | blue | 0.158926559 | 0.308711274 |
| CAPN6 | blue | 0.070943874 | 0.651217058 |
| CAPN7 | blue | 0.23499128 | 0.129300956 |
| CAPN9 | lightyellow | 0.000668165 | 0.996607134 |
| CAPZA3 | blue | -0.09739288 | 0.534396616 |
| CARD8 | blue | 0.521367332 | 0.000337071 |
| CARM1 | blue | -0.26645644 | 0.084138193 |
| CARS2 | blue | -0.160075812 | 0.305180568 |
| CASK | blue | -0.002486633 | 0.987373633 |
| CASKIN1 | blue | -0.234241324 | 0.130563479 |
| CASP1 | lightyellow | 0.495608287 | 0.000726626 |
| CASP3 | blue | 0.378119247 | 0.012418634 |
| CASP4 | blue | 0.290329198 | 0.058942574 |
| CASP7 | blue | 0.357588677 | 0.018562536 |
| CASP9 | blue | -0.319309326 | 0.036875763 |
| CASS4 | blue | 0.268886019 | 0.081243371 |
| CATSPERG | blue | 0.038334852 | 0.807183818 |
| CBFB | blue | 0.365663996 | 0.015894589 |
| CBLC | blue | -0.387712732 | 0.01020401 |
| CBLN3 | blue | 0.219240557 | 0.15778951 |
| CBR4 | blue | -0.242318247 | 0.117442443 |
| CBS | lightcyan | -0.362973607 | 0.016744853 |
| CBWD2 | blue | -0.085414782 | 0.586032428 |
| CBX1 | blue | -0.101527803 | 0.517100407 |
| CCDC102A | blue | -0.471741159 | 0.00140579 |
| CCDC102B | blue | 0.072675993 | 0.64326505 |
| CCDC110 | blue | -0.222562534 | 0.151430181 |
| CCDC122 | blue | -0.131677864 | 0.399956208 |
| CCDC130 | blue | -0.031352218 | 0.841808248 |
| CCDC136 | blue | 0.034708782 | 0.825123221 |
| CCDC137 | blue | 0.020839279 | 0.894478219 |
| CCDC154 | blue | 0.146837404 | 0.347417337 |
| CCDC171 | blue | 0.11095015 | 0.478758054 |
| CCDC173 | blue | -0.399383152 | 0.00797385 |
| CCDC175 | blue | -0.044594589 | 0.776448254 |
| CCDC28A | blue | -0.153611307 | 0.325377337 |
| CCDC39 | blue | 0.15095078 | 0.333927066 |
| CCDC50 | blue | 0.244404903 | 0.114220535 |
| CCDC60 | blue | 0.11064108 | 0.479991551 |
| CCDC61 | blue | 0.000600129 | 0.996952614 |
| CCDC62 | blue | 0.260729377 | 0.09127987 |
| CCDC69 | lightcyan | 0.483844287 | 0.001011956 |
| CCDC71L | blue | 0.06487292 | 0.679386291 |
| CCDC80 | blue | 0.022606863 | 0.885584886 |
| CCDC81 | blue | 0.131319347 | 0.401251812 |
| CCDC82 | blue | 0.295267248 | 0.054575096 |
| CCDC83 | blue | -0.060574271 | 0.699598839 |
| CCDC9 | blue | 0.172589218 | 0.268413167 |
| CCDC96 | blue | 0.084640523 | 0.589445307 |
| CCIN | blue | 0.023677498 | 0.880204814 |
| CCKAR | blue | 0.076479202 | 0.625943248 |
| CCL1 | blue | 0.103528619 | 0.508832518 |
| CCL22 | blue | 0.134677845 | 0.389210182 |
| CCL27 | blue | -0.012419003 | 0.937000713 |
| CCL28 | blue | -0.190687315 | 0.220631434 |
| CCL7 | blue | 0.091587082 | 0.559146801 |
| CCNB1 | blue | 0.037932946 | 0.809167559 |
| CCNB2 | blue | 0.00725463 | 0.963174316 |
| CCND1 | blue | 0.361856634 | 0.017108983 |
| CCND2 | blue | 0.339779882 | 0.025799451 |
| CCNE1 | blue | 0.04260647 | 0.786175936 |
| CCNE2 | blue | 0.210388087 | 0.175683149 |
| CCNF | blue | -0.205440981 | 0.186295423 |
| CCNJ | blue | 0.182596465 | 0.241210378 |
| CCNJL | blue | -0.185673256 | 0.233236578 |
| CCNK | blue | -0.011384884 | 0.942237372 |
| CCNY | blue | 0.104203138 | 0.506060348 |
| CCP110 | blue | 0.03893629 | 0.804217461 |
| CCR1 | blue | 0.130688947 | 0.403535818 |
| CCR10 | blue | 0.072033002 | 0.646212439 |
| CCRL2 | blue | 0.219553998 | 0.157181295 |
| CCS | blue | -0.31592114 | 0.039039126 |
| CCSAP | blue | 0.024354376 | 0.87680615 |
| CCT3 | blue | -0.090484798 | 0.563905627 |
| CCT6B | blue | -0.245223672 | 0.112974778 |
| CCT8 | blue | 0.223662133 | 0.149367185 |
| CCT8L2 | blue | 0.063124677 | 0.687580862 |
| CCZ1 | blue | 0.222277587 | 0.151968173 |
| CCZ1B | blue | 0.312556069 | 0.041289356 |
| CD109 | blue | 0.409313244 | 0.006420507 |
| CD160 | blue | 0.313945115 | 0.040348007 |
| CD19 | blue | 0.211952905 | 0.172418598 |
| CD1A | blue | 0.197180809 | 0.205017256 |
| CD1C | blue | 0.20570608 | 0.185715438 |
| CD207 | blue | 0.02221528 | 0.887553918 |
| CD244 | blue | 0.053351664 | 0.734019701 |
| CD248 | blue | 0.528566568 | 0.000268943 |
| CD27 | blue | 0.165255493 | 0.289589162 |
| CD276 | blue | 0.077251378 | 0.622450111 |
| CD300LB | blue | 0.157131344 | 0.314278307 |
| CD300LF | blue | -0.030951615 | 0.843804316 |
| CD300LG | blue | -0.22930938 | 0.139096587 |
| CD40 | blue | 0.286033186 | 0.062965463 |
| CD5 | blue | 0.298982234 | 0.051463817 |
| CD5L | blue | 0.099618502 | 0.525052104 |
| CD7 | blue | 0.024438686 | 0.876382973 |
| CD72 | blue | 0.200723828 | 0.196831893 |
| CD74 | lightyellow | 0.36921784 | 0.014827698 |
| CD79A | blue | 0.04350362 | 0.781782188 |
| CD80 | blue | 0.226110389 | 0.144848223 |
| CD99L2 | blue | -0.133426222 | 0.393672808 |
| CDADC1 | blue | -0.146655505 | 0.348021497 |
| CDC14B | blue | -0.208039725 | 0.180665268 |
| CDC20B | blue | 0.027600704 | 0.860537469 |
| CDC25C | blue | -0.135554469 | 0.386102329 |
| CDC34 | blue | -0.226202222 | 0.144680704 |
| CDC37L1 | blue | -0.125797584 | 0.42151085 |
| CDC42SE1 | blue | 0.306034998 | 0.045950942 |
| CDCA2 | blue | 0.080833068 | 0.606355174 |
| CDCA4 | lightcyan | -0.279985445 | 0.068995701 |
| CDCA8 | blue | 0.057473332 | 0.714308967 |
| CDH23 | blue | -0.103465039 | 0.509094217 |
| CDH24 | blue | 0.206359671 | 0.184291 |
| CDH5 | lightcyan | 0.37932894 | 0.012118564 |
| CDH7 | blue | 0.029534183 | 0.850874583 |
| CDHR1 | blue | 0.150498406 | 0.335394538 |
| CDIP1 | blue | -0.301297423 | 0.049598263 |
| CDK15 | blue | 0.076324137 | 0.626645706 |
| CDK16 | blue | -0.286883708 | 0.062152139 |
| CDK2 | blue | 0.3087943 | 0.043928902 |
| CDK20 | blue | -0.44444633 | 0.002827936 |
| CDK7 | blue | -0.177423209 | 0.255029837 |
| CDK9 | blue | 0.283156356 | 0.065779429 |
| CDKAL1 | blue | 0.396107399 | 0.008552758 |
| CDKL1 | blue | -0.470138456 | 0.001467026 |
| CDKN2A | blue | 0.072516716 | 0.643994656 |
| CDKN2AIP | blue | -0.404094031 | 0.007200668 |
| CDKN2C | blue | -0.045851427 | 0.770315928 |
| CDKN3 | blue | 0.110478499 | 0.48064108 |
| CDO1 | blue | -0.164879884 | 0.29070209 |
| CDR1 | blue | -0.076939729 | 0.623858955 |
| CDR2 | blue | -0.030467053 | 0.846220015 |
| CDR2L | blue | 0.304033709 | 0.047464209 |
| CDRT1 | blue | 0.142655226 | 0.361470243 |
| CDSN | blue | 0.029471896 | 0.851185542 |
| CEACAM16 | blue | -0.434822503 | 0.003570579 |
| CEACAM18 | blue | 0.180008142 | 0.2480598 |
| CEACAM19 | blue | -0.149787992 | 0.337707136 |
| CEACAM20 | blue | -0.167064762 | 0.28426705 |
| CEACAM21 | blue | 0.108535304 | 0.48843992 |
| CEACAM7 | blue | 0.068370254 | 0.663102996 |
| CEBPA | blue | -0.042523143 | 0.78658436 |
| CECR2 | blue | 0.157060071 | 0.314500632 |
| CELA2A | blue | 0.107149445 | 0.494041802 |
| CELA2B | blue | 0.056814091 | 0.717449687 |
| CELA3B | blue | -0.353584042 | 0.020019658 |
| CELF1 | blue | 0.255203141 | 0.098607405 |
| CELF4 | blue | 0.009895922 | 0.949781468 |
| CEMP1 | blue | 0.065440441 | 0.676733911 |
| CENPH | blue | 0.023502047 | 0.881086116 |
| CENPI | blue | 0.059628788 | 0.704072789 |
| CENPJ | blue | -0.008337661 | 0.957681317 |
| CENPL | blue | -0.236157234 | 0.127356279 |
| CENPT | blue | 0.125788301 | 0.421545387 |
| CEP120 | blue | 0.200140859 | 0.198162626 |
| CEP135 | blue | 0.12728099 | 0.416012366 |
| CEP192 | blue | 0.078033077 | 0.618922206 |
| CEP68 | blue | 0.190286606 | 0.221621145 |
| CEP70 | blue | -0.494954337 | 0.00074036 |
| CEP85 | blue | 0.186243157 | 0.231779621 |
| CEP85L | blue | -0.022998602 | 0.883615746 |
| CEP97 | blue | 0.011949631 | 0.939377247 |
| CEPT1 | blue | 0.086023566 | 0.583355165 |
| CER1 | blue | -0.174347372 | 0.263492847 |
| CERS1 | blue | -0.272321515 | 0.077283537 |
| CES1 | blue | -0.113368193 | 0.469165458 |
| CES5A | blue | -0.225988828 | 0.145070192 |
| CFHR2 | blue | -0.103447175 | 0.509167757 |
| CFHR3 | blue | 0.02385356 | 0.87932058 |
| CFHR4 | blue | -0.180418263 | 0.24696586 |
| CFL1 | blue | 0.105949743 | 0.498917792 |
| CFL2 | blue | -0.216261641 | 0.163655833 |
| CGA | lightcyan | 0.219009189 | 0.158239568 |
| CGB1 | blue | -0.359171385 | 0.01801182 |
| CHAF1A | blue | -0.123098575 | 0.431619704 |
| CHAT | blue | -0.04942016 | 0.752979844 |
| CHCHD3 | blue | -0.320384345 | 0.03621027 |
| CHCHD4 | blue | -0.214069655 | 0.16807243 |
| CHD2 | blue | 0.32254849 | 0.03490043 |
| CHD4 | blue | 0.068012005 | 0.6647641 |
| CHD5 | blue | 0.093819054 | 0.549568762 |
| CHD9 | blue | -0.040172744 | 0.798127689 |
| CHERP | blue | -0.1496594 | 0.338126791 |
| CHGB | blue | 0.058329345 | 0.710237748 |
| CHI3L1 | blue | -0.297899065 | 0.052355827 |
| CHIC1 | blue | -0.152917077 | 0.327594941 |
| CHKA | blue | -0.054293172 | 0.729501772 |
| CHKB | blue | 0.322139694 | 0.035144823 |
| CHMP2B | blue | -0.185746782 | 0.233048258 |
| CHMP3 | blue | 0.125521343 | 0.422539295 |
| CHMP7 | blue | 0.283842077 | 0.0650998 |
| CHP1 | blue | -0.302704425 | 0.048491454 |
| CHRD | blue | 0.084659826 | 0.589360114 |
| CHRM2 | blue | 0.052382428 | 0.738679962 |
| CHRM3 | lightcyan | 0.452189179 | 0.002332707 |
| CHRNA10 | blue | 0.075980996 | 0.62820132 |
| CHRNA6 | blue | 0.031334007 | 0.841898966 |
| CHRNE | blue | -0.275084854 | 0.074209662 |
| CHST1 | blue | -0.027085289 | 0.863116797 |
| CHST10 | blue | -0.217210321 | 0.161770698 |
| CHST2 | blue | 0.114467498 | 0.464838513 |
| CHST4 | blue | -0.13567101 | 0.385690264 |
| CHST6 | blue | -0.022514871 | 0.886047397 |
| CHST9 | blue | -0.17915259 | 0.250352359 |
| CHSY1 | blue | 0.382433196 | 0.011376698 |
| CHSY3 | blue | 0.044660922 | 0.776124265 |
| CIAO1 | blue | -0.026871955 | 0.864184816 |
| CIB1 | blue | -0.210488881 | 0.175471543 |
| CIB3 | blue | -0.007250585 | 0.963194834 |
| CIC | blue | 0.306821303 | 0.045367191 |
| CIDEC | blue | -0.032013531 | 0.838515294 |
| CIITA | blue | 0.557544643 | 0.000102762 |
| CILP2 | blue | -0.261769729 | 0.089948826 |
| CKAP2 | blue | 0.150075633 | 0.336769598 |
| CLASP1 | blue | 0.103284624 | 0.509837184 |
| CLASRP | blue | -0.054945046 | 0.726378965 |
| CLCA1 | blue | 0.153643827 | 0.325273688 |
| CLCN4 | blue | -0.253632803 | 0.10076995 |
| CLCN7 | blue | 0.032126293 | 0.837954074 |
| CLDN11 | blue | 0.081846057 | 0.601835866 |
| CLDN12 | blue | -0.140661782 | 0.368287596 |
| CLDN14 | blue | -0.08045569 | 0.608042521 |
| CLDN15 | blue | 0.217169683 | 0.161851125 |
| CLDN17 | blue | 0.177610072 | 0.254521625 |
| CLDN3 | blue | -0.072846325 | 0.642485175 |
| CLDN6 | blue | 0.041321775 | 0.792479069 |
| CLDN9 | blue | -0.271105324 | 0.078667655 |
| CLEC12B | blue | -0.034219883 | 0.827548925 |
| CLEC14A | blue | 0.188109335 | 0.227052312 |
| CLEC17A | blue | 0.274057002 | 0.075341582 |
| CLEC2B | lightyellow | 0.339543791 | 0.025909254 |
| CLEC4F | blue | 0.257144633 | 0.095983263 |
| CLEC4G | blue | -0.335389085 | 0.027906107 |
| CLEC4M | blue | 0.17195664 | 0.270198251 |
| CLEC9A | blue | -0.129248732 | 0.408781911 |
| CLECL1 | blue | 0.239157037 | 0.122453712 |
| CLGN | blue | -0.217712671 | 0.160778887 |
| CLIC2 | blue | 0.258134465 | 0.094666276 |
| CLIP2 | blue | -0.104646339 | 0.504243036 |
| CLK1 | blue | 0.459975225 | 0.001913436 |
| CLMP | blue | 0.071235009 | 0.649877776 |
| CLN3 | blue | -0.036984257 | 0.813854787 |
| CLN6 | blue | -0.176462743 | 0.257652729 |
| CLNS1A | blue | -0.223234846 | 0.150166365 |
| CLP1 | blue | -0.030277315 | 0.847166303 |
| CLPSL2 | blue | 0.036776381 | 0.814882714 |
| CLPTM1 | blue | -0.26222067 | 0.089376579 |
| CLRN2 | blue | -0.204668012 | 0.187993882 |
| CLSPN | blue | 0.149962467 | 0.337138258 |
| CLSTN2 | blue | -0.228874748 | 0.139867937 |
| CLUL1 | blue | -0.071081482 | 0.650583897 |
| CLVS1 | blue | -0.099816329 | 0.524225422 |
| CMKLR1 | blue | 0.36978069 | 0.014664429 |
| CMPK2 | lightyellow | 0.315024806 | 0.039628472 |
| CMTM5 | blue | 0.133737101 | 0.392561603 |
| CMYA5 | blue | 0.163371171 | 0.29520028 |
| CNBD1 | blue | -0.189237172 | 0.224227644 |
| CNBD2 | blue | -0.108034013 | 0.490462412 |
| CNGA1 | blue | 0.041646291 | 0.79088564 |
| CNGA2 | blue | -0.191367852 | 0.218957575 |
| CNGA4 | blue | -0.090536413 | 0.563682372 |
| CNGB3 | blue | -0.183299172 | 0.239373169 |
| CNIH2 | blue | -0.006877104 | 0.965089487 |
| CNIH3 | blue | 0.032956483 | 0.833824659 |
| CNKSR3 | blue | 0.058380145 | 0.70999639 |
| CNN3 | blue | -0.090948822 | 0.561900036 |
| CNNM4 | blue | -0.24098785 | 0.119532207 |
| CNOT11 | blue | -0.241022734 | 0.119477056 |
| CNOT3 | blue | 0.264042118 | 0.087093834 |
| CNOT6L | blue | 0.032902567 | 0.834092707 |
| CNOT8 | lightcyan | 0.3167097 | 0.038526586 |
| CNP | blue | -0.196791399 | 0.205931218 |
| CNR2 | blue | 0.145796185 | 0.350884334 |
| CNRIP1 | blue | 0.115437558 | 0.46103811 |
| CNTFR | blue | 0.138238804 | 0.376676857 |
| CNTLN | lightcyan | 0.036982309 | 0.813864417 |
| CNTN2 | blue | -0.099140379 | 0.527052741 |
| CNTN3 | blue | 0.31177719 | 0.041824985 |
| CNTNAP2 | blue | -0.048115375 | 0.759304787 |
| COA4 | blue | -0.237413268 | 0.125285959 |
| COA6 | blue | 0.065405121 | 0.676898872 |
| COBL | blue | -0.195744241 | 0.20840307 |
| COG2 | blue | -0.100754804 | 0.520312479 |
| COIL | blue | -0.19479199 | 0.210668802 |
| COL24A1 | blue | -0.091605843 | 0.559065971 |
| COL25A1 | blue | 0.225876349 | 0.1452758 |
| COL9A1 | blue | -0.083335322 | 0.595218429 |
| COLEC10 | blue | 0.087359305 | 0.577500279 |
| COLQ | blue | 0.032156067 | 0.8378059 |
| COMMD1 | blue | -0.291140605 | 0.058206343 |
| COMMD10 | blue | 0.105438308 | 0.501003894 |
| COMMD3 | blue | -0.205042354 | 0.187169963 |
| COMMD8 | blue | -0.20376211 | 0.189998406 |
| COMMD9 | blue | -0.429570038 | 0.004043885 |
| COPA | blue | 0.127310939 | 0.415901778 |
| COPG2 | blue | -0.138065579 | 0.377280939 |
| COQ10A | blue | -0.256683375 | 0.096601777 |
| COQ10B | blue | 0.150836276 | 0.33429813 |
| COQ2 | blue | 0.021429037 | 0.891509468 |
| COQ5 | blue | -0.246027364 | 0.111762012 |
| CORIN | blue | -0.146398166 | 0.348877323 |
| COX10 | blue | -0.273761495 | 0.075669508 |
| COX14 | blue | -0.216088797 | 0.164001004 |
| COX20 | blue | -0.233691821 | 0.131494375 |
| COX5B | blue | -0.331691237 | 0.029789255 |
| COX7A2L | lightcyan | -0.453378031 | 0.002263867 |
| COX7B | blue | -0.312852353 | 0.041087078 |
| COX7C | blue | -0.340862115 | 0.025301063 |
| CPA1 | blue | -0.085719398 | 0.584692126 |
| CPAMD8 | blue | -0.188267625 | 0.226654407 |
| CPB1 | blue | 0.03172227 | 0.839965272 |
| CPB2 | blue | 0.147637866 | 0.344766323 |
| CPED1 | blue | 0.298070455 | 0.052213862 |
| CPNE1 | blue | 0.22566239 | 0.145667504 |
| CPNE2 | blue | 0.242873457 | 0.116578541 |
| CPNE4 | blue | -0.591350894 | 2.97E-05 |
| CPO | blue | 0.084979286 | 0.587950977 |
| CPOX | blue | 0.044168924 | 0.77852821 |
| CPPED1 | blue | -0.387521076 | 0.010244694 |
| CPSF3 | blue | -0.092514423 | 0.55515785 |
| CPSF4 | blue | -0.009994376 | 0.949282493 |
| CPT1C | blue | 0.109271282 | 0.485478435 |
| CR2 | blue | -0.277629831 | 0.071464558 |
| CREB3L1 | blue | -0.354194813 | 0.019791403 |
| CREB3L4 | blue | -0.039544626 | 0.801219836 |
| CREBL2 | blue | -0.339076371 | 0.026127793 |
| CREG1 | blue | -0.166036353 | 0.287284326 |
| CRHBP | lightyellow | 0.384177305 | 0.010977174 |
| CRHR2 | blue | -0.061390999 | 0.695742207 |
| CRIP3 | blue | -0.122953828 | 0.432165629 |
| CRISP2 | blue | -0.394173852 | 0.008911158 |
| CRISPLD2 | blue | 0.251731906 | 0.103436181 |
| CRK | lightcyan | 0.103065127 | 0.510741831 |
| CRLF3 | blue | 0.383548323 | 0.011119849 |
| CRY2 | blue | -0.084516864 | 0.589991205 |
| CRYBB2 | blue | 0.064733459 | 0.680038668 |
| CRYGB | blue | -0.026253537 | 0.867282154 |
| CSAG1 | lightyellow | 0.278098067 | 0.070968342 |
| CSAG2 | lightyellow | 0.176589243 | 0.257306251 |
| CSF1 | blue | 0.276945312 | 0.072194902 |
| CSF2RA | blue | 0.310617552 | 0.042632937 |
| CSF2RB | blue | 0.143607545 | 0.358240493 |
| CSF3 | lightcyan | -0.247901204 | 0.108972828 |
| CSGALNACT2 | blue | 0.212733444 | 0.170806654 |
| CSHL1 | blue | -0.173954098 | 0.264588215 |
| CSK | blue | 0.138481517 | 0.375831413 |
| CSMD2 | blue | 0.036836071 | 0.81458752 |
| CSN2 | lightcyan | -0.257425031 | 0.095608765 |
| CSNK1A1L | blue | -0.331185337 | 0.03005488 |
| CSNK1E | lightcyan | 0.457249514 | 0.00205194 |
| CSPG5 | blue | 0.065223957 | 0.677745224 |
| CSPP1 | blue | 0.058635683 | 0.708782724 |
| CSRNP2 | blue | 0.230608843 | 0.136809218 |
| CSRNP3 | blue | -0.320279336 | 0.036274839 |
| CSRP2 | blue | -0.060518046 | 0.699864611 |
| CSRP3 | blue | 0.114254681 | 0.465674505 |
| CST11 | blue | 0.003820325 | 0.980602613 |
| CST2 | blue | -0.131018721 | 0.402340078 |
| CST4 | blue | -0.158689915 | 0.309441502 |
| CST8 | blue | -0.123654368 | 0.429527052 |
| CST9 | blue | 0.09359165 | 0.550541045 |
| CSTF1 | blue | -0.082577099 | 0.598583544 |
| CSTF2T | blue | -0.090409015 | 0.564233486 |
| CSTF3 | blue | 0.052776095 | 0.736786015 |
| CT47A6 | blue | -0.07040825 | 0.653683867 |
| CTAGE15 | blue | -0.165295649 | 0.289470342 |
| CTAGE9 | blue | -0.190297528 | 0.221594127 |
| CTC1 | blue | 0.195177586 | 0.209749279 |
| CTDP1 | blue | -0.123790245 | 0.429016319 |
| CTDSPL2 | blue | 0.227035094 | 0.143167922 |
| CTIF | blue | 0.366169948 | 0.015738843 |
| CTLA4 | blue | -0.161827241 | 0.29984969 |
| CTNNA2 | blue | -0.073909701 | 0.637625036 |
| CTNNBL1 | blue | -0.056158885 | 0.720575741 |
| CTNND2 | blue | 0.020733671 | 0.895009988 |
| CTNS | blue | -0.088957941 | 0.570528288 |
| CTRB1 | blue | -0.027798558 | 0.859547711 |
| CTSK | blue | 0.254050723 | 0.100190904 |
| CTSZ | blue | 0.180573061 | 0.246553807 |
| CTTN | blue | 0.090588871 | 0.563455518 |
| CTTNBP2 | blue | -0.136514331 | 0.382716173 |
| CTU1 | lightyellow | -0.118326641 | 0.449819253 |
| CTXN1 | blue | -0.137871744 | 0.377957578 |
| CUL5 | blue | -0.301041993 | 0.049801366 |
| CUL7 | blue | -0.244221507 | 0.114500994 |
| CUTA | blue | -0.371108399 | 0.014285328 |
| CUX2 | blue | -0.223653371 | 0.149383542 |
| CWC15 | blue | -0.136498942 | 0.382770322 |
| CWC22 | blue | 0.15085284 | 0.334244437 |
| CXCL16 | blue | 0.199014077 | 0.200752645 |
| CXCR3 | blue | 0.036262571 | 0.817424764 |
| CXCR6 | blue | 0.271882091 | 0.077781414 |
| CXorf51A | blue | -0.38416018 | 0.010981038 |
| CXorf65 | blue | -0.061123306 | 0.697005441 |
| CXorf66 | blue | 0.119781759 | 0.444225703 |
| CXXC5 | blue | -0.271086751 | 0.078688942 |
| CYB561D1 | blue | 0.009725433 | 0.950645574 |
| CYB5B | blue | -0.224931206 | 0.147011988 |
| CYB5D2 | blue | -0.207013326 | 0.182874232 |
| CYC1 | blue | -0.31973582 | 0.036610551 |
| CYFIP1 | blue | 0.177654734 | 0.254400259 |
| CYGB | blue | 0.425495304 | 0.004447953 |
| CYLC2 | blue | 0.039807937 | 0.799923222 |
| CYP11A1 | blue | -0.144173469 | 0.356329493 |
| CYP11B2 | blue | 0.105319352 | 0.501489746 |
| CYP19A1 | blue | 0.012968757 | 0.934217878 |
| CYP1B1 | blue | 0.037360909 | 0.811993068 |
| CYP26C1 | lightcyan | -0.228669045 | 0.140234104 |
| CYP27C1 | blue | -0.067314989 | 0.668000519 |
| CYP2A13 | blue | -0.085176563 | 0.587081541 |
| CYP2C8 | blue | 0.220292737 | 0.15575458 |
| CYP2C9 | blue | 0.069799838 | 0.656490327 |
| CYP2R1 | blue | 0.021828093 | 0.889501507 |
| CYP2W1 | blue | -0.012051072 | 0.938863587 |
| CYP39A1 | blue | -0.382500445 | 0.011361066 |
| CYP3A5 | blue | -0.150782299 | 0.334473138 |
| CYP4F11 | blue | -0.022346386 | 0.88689459 |
| CYP4X1 | blue | 0.099946653 | 0.52368117 |
| CYP51A1 | blue | 0.03199284 | 0.838618279 |
| CYP7B1 | blue | -0.046320097 | 0.768032721 |
| CYTH1 | blue | 0.235621253 | 0.128247495 |
| CYTH2 | blue | 0.020619263 | 0.895586116 |
| CYTH3 | blue | 0.055042852 | 0.7259108 |
| DAAM1 | blue | 0.226848259 | 0.143506257 |
| DAAM2 | blue | 0.184070995 | 0.237366236 |
| DACH2 | blue | 0.004055595 | 0.979408299 |
| DACT3 | blue | -0.274114105 | 0.075278344 |
| DALRD3 | blue | -0.294315031 | 0.055396416 |
| DAP3 | blue | -0.113686716 | 0.467909527 |
| DAPK1 | blue | -0.134499199 | 0.389845316 |
| DAPK2 | blue | 0.276732865 | 0.072422764 |
| DAW1 | blue | 0.026946833 | 0.863809922 |
| DAZ2 | blue | 0.286012539 | 0.062985312 |
| DAZ3 | blue | 0.263041472 | 0.088342226 |
| DAZ4 | blue | 0.269146097 | 0.080938161 |
| DBF4 | blue | -0.184170762 | 0.237107655 |
| DBI | blue | -0.3761478 | 0.012921231 |
| DBN1 | blue | 0.165177393 | 0.289820344 |
| DBNDD2 | blue | 0.02554143 | 0.870851156 |
| DBX2 | blue | -0.214880841 | 0.166428053 |
| DCAF12 | blue | -0.251811635 | 0.103323278 |
| DCAF17 | blue | -0.125245614 | 0.423567244 |
| DCAF5 | blue | 0.015553011 | 0.921147383 |
| DCC | blue | -0.002089356 | 0.989390763 |
| DCD | blue | 0.165496585 | 0.288876263 |
| DCHS1 | blue | 0.405871236 | 0.006926289 |
| DCLK1 | blue | -0.066686061 | 0.670925899 |
| DCLK2 | blue | 0.239585362 | 0.121765449 |
| DCLK3 | blue | 0.064013809 | 0.683408729 |
| DCLRE1A | blue | -0.167512301 | 0.282960476 |
| DCN | blue | -0.005118337 | 0.974014026 |
| DCP1B | blue | -0.136178175 | 0.383900047 |
| DCP2 | blue | 0.081288157 | 0.604323052 |
| DCST1 | blue | -0.087664263 | 0.576167315 |
| DCST2 | blue | -0.173465658 | 0.265952845 |
| DCSTAMP | blue | 0.019760207 | 0.899913765 |
| DCTN4 | blue | -0.033378738 | 0.831726023 |
| DCTN6 | lightcyan | 0.218164305 | 0.159890989 |
| DCUN1D2 | blue | 0.152665763 | 0.328400044 |
| DCUN1D5 | blue | 0.023684906 | 0.880167604 |
| DDB1 | blue | -0.277385653 | 0.071724411 |
| DDI1 | blue | 0.083236044 | 0.595658569 |
| DDR2 | blue | 0.268537893 | 0.081653317 |
| DDTL | blue | -0.30683502 | 0.045357061 |
| DDX1 | blue | -0.224688295 | 0.147460663 |
| DDX10 | blue | -0.231879017 | 0.134600523 |
| DDX11 | blue | 0.038470439 | 0.806514857 |
| DDX19B | blue | 0.004833718 | 0.975458591 |
| DDX28 | blue | -0.201096573 | 0.195984343 |
| DDX41 | blue | -0.116491909 | 0.456926517 |
| DDX47 | blue | 0.117595668 | 0.452643592 |
| DDX51 | blue | 0.042486845 | 0.786762292 |
| DDX53 | blue | -0.008953387 | 0.954559279 |
| DDX55 | blue | 0.265822591 | 0.084906481 |
| DDX56 | blue | 0.024006662 | 0.878551783 |
| DDX58 | lightyellow | 0.404426722 | 0.007148601 |
| DDX59 | blue | 0.28365466 | 0.065284996 |
| DDX60 | lightyellow | 0.347607291 | 0.022372322 |
| DDX60L | lightyellow | 0.318469595 | 0.037402547 |
| DEF6 | blue | 0.222957439 | 0.150686895 |
| DEFA1B | blue | 0.11239709 | 0.47300556 |
| DEFA5 | blue | -0.102024119 | 0.515043275 |
| DEFB103A | blue | -0.104210864 | 0.506028639 |
| DEFB105A | blue | -0.032797762 | 0.834613809 |
| DEFB110 | blue | -0.045928279 | 0.769941398 |
| DEFB112 | blue | 0.070487243 | 0.653319835 |
| DEFB114 | blue | -0.098070253 | 0.531544057 |
| DEFB115 | blue | 0.090848859 | 0.562331812 |
| DEFB116 | blue | -0.220172399 | 0.15598634 |
| DEFB123 | blue | -0.242621762 | 0.116969579 |
| DEFB127 | blue | -0.298302826 | 0.052021879 |
| DEFB128 | blue | -0.210632628 | 0.175170079 |
| DEFB136 | blue | -0.222436919 | 0.151667175 |
| DEGS2 | blue | -0.130903198 | 0.402758725 |
| DENND1B | blue | -0.060688101 | 0.69906088 |
| DENND2A | blue | 0.007864636 | 0.960080219 |
| DENND2C | blue | 0.336512838 | 0.027353805 |
| DENND2D | blue | -0.064451592 | 0.681357902 |
| DENND5B | blue | -0.088482409 | 0.572598145 |
| DENND6A | blue | 0.142388587 | 0.362377676 |
| DEPTOR | blue | -0.307522764 | 0.04485152 |
| DERL3 | blue | 0.018671011 | 0.905404896 |
| DET1 | blue | 0.020764013 | 0.894857202 |
| DEXI | blue | -0.227190412 | 0.142887107 |
| DGCR6L | blue | -0.146048171 | 0.350043354 |
| DGKA | blue | 0.443339693 | 0.002905778 |
| DGKD | blue | 0.300481318 | 0.050249537 |
| DGKE | blue | 0.333837085 | 0.028684074 |
| DHDDS | blue | -0.15253428 | 0.32882175 |
| DHH | blue | 0.036715564 | 0.815183505 |
| DHRS1 | blue | -0.117581767 | 0.452697395 |
| DHRS11 | blue | -0.054687001 | 0.72761461 |
| DHRS9 | blue | 0.037531463 | 0.811150388 |
| DHX16 | blue | 0.277599937 | 0.071496331 |
| DHX29 | blue | -0.062371757 | 0.691121018 |
| DHX32 | blue | 0.115231903 | 0.461842406 |
| DHX33 | blue | 0.016607361 | 0.915820436 |
| DHX34 | blue | -0.026659006 | 0.865251143 |
| DHX58 | lightyellow | 0.156272727 | 0.316963239 |
| DHX8 | blue | 0.418406056 | 0.005235272 |
| DIAPH2 | lightcyan | 0.255462497 | 0.0982537 |
| DIDO1 | blue | 0.041674026 | 0.790749494 |
| DIP2A | blue | -0.014520791 | 0.926365819 |
| DIRAS1 | blue | 0.263397947 | 0.087895911 |
| DIRAS2 | blue | -0.190858695 | 0.220209074 |
| DIRAS3 | blue | -0.118645531 | 0.448590145 |
| DIS3L | blue | -0.201226025 | 0.195690597 |
| DISC1 | blue | 0.344551228 | 0.023662037 |
| DKK1 | blue | -0.484509778 | 0.000993482 |
| DKK2 | blue | 0.075131376 | 0.632059868 |
| DKK3 | blue | 0.254825503 | 0.099124171 |
| DLAT | blue | -0.397097238 | 0.008374132 |
| DLC1 | blue | -0.067422724 | 0.667499889 |
| DLG1 | blue | -0.242136267 | 0.117726652 |
| DLG5 | blue | 0.166174414 | 0.286878062 |
| DLGAP5 | blue | 0.141335702 | 0.365974295 |
| DLK2 | blue | 0.008977615 | 0.954436443 |
| DLL4 | blue | 0.057693182 | 0.713262603 |
| DLX1 | blue | -0.268201731 | 0.082050703 |
| DLX4 | blue | -0.001053284 | 0.994651567 |
| DMBT1 | blue | -0.190037067 | 0.222239027 |
| DMBX1 | blue | -0.281915859 | 0.0670232 |
| DMC1 | blue | -0.176560506 | 0.257384931 |
| DMP1 | blue | 0.167467086 | 0.283092302 |
| DMPK | blue | -0.125987432 | 0.420804867 |
| DMRTC2 | blue | -0.06294921 | 0.688405307 |
| DNAAF2 | blue | -0.125103521 | 0.424097534 |
| DNAH2 | blue | 0.026797029 | 0.864559973 |
| DNAH3 | blue | -0.147879553 | 0.343968338 |
| DNAH5 | blue | 0.084230284 | 0.591257183 |
| DNAH9 | blue | 0.04149088 | 0.791648633 |
| DNAJA1 | lightcyan | 0.300620041 | 0.050138349 |
| DNAJA2 | blue | 0.044284437 | 0.777963621 |
| DNAJA3 | blue | -0.440061107 | 0.003147559 |
| DNAJB13 | blue | -0.248529296 | 0.108049881 |
| DNAJB14 | blue | 0.297168778 | 0.052964226 |
| DNAJB5 | blue | -0.263958271 | 0.087197911 |
| DNAJC11 | blue | -0.207515285 | 0.181791545 |
| DNAJC21 | lightcyan | 0.19427409 | 0.21190824 |
| DNAJC24 | blue | 0.391464757 | 0.00943499 |
| DNAJC30 | blue | -0.249330248 | 0.106881584 |
| DNAJC5G | blue | 0.112766245 | 0.471543827 |
| DNAJC7 | blue | -0.150940855 | 0.333959217 |
| DNALI1 | blue | -0.244429973 | 0.114182238 |
| DNASE1L1 | blue | 0.441181135 | 0.003063048 |
| DNM2 | blue | -0.000944962 | 0.995201605 |
| DNMBP | blue | -0.077801989 | 0.619964259 |
| DNTTIP2 | lightcyan | 0.351304727 | 0.020891098 |
| DOC2A | blue | 0.310235279 | 0.042902045 |
| DOC2B | blue | 0.277029713 | 0.072104534 |
| DOCK1 | blue | -0.080687544 | 0.607005608 |
| DOCK10 | blue | 0.469458953 | 0.001493695 |
| DOCK3 | blue | 0.127991997 | 0.413391427 |
| DOCK6 | lightcyan | 0.542585031 | 0.000170722 |
| DOCK9 | lightcyan | 0.467210983 | 0.001585012 |
| DOHH | blue | -0.224955415 | 0.146967328 |
| DOK3 | blue | -0.288034875 | 0.06106467 |
| DOK4 | blue | -0.270028352 | 0.079909477 |
| DOT1L | blue | 0.175844072 | 0.259351737 |
| DPH1 | blue | 0.04711026 | 0.764187703 |
| DPH5 | blue | -0.135654616 | 0.385748217 |
| DPP3 | blue | -0.260654514 | 0.091376235 |
| DPPA3 | blue | -0.089112165 | 0.569857735 |
| DPPA4 | blue | 0.156178721 | 0.317258076 |
| DPRX | blue | -0.221534906 | 0.15337697 |
| DPT | blue | -0.357507234 | 0.018591254 |
| DPY30 | blue | -0.115901209 | 0.45922759 |
| DPYSL3 | blue | 0.042479047 | 0.786800522 |
| DPYSL4 | blue | -0.039986764 | 0.799042932 |
| DQX1 | blue | -0.059356433 | 0.705363394 |
| DRD2 | blue | 0.15281119 | 0.327934008 |
| DRD3 | blue | -0.15072373 | 0.334663102 |
| DRD5 | blue | -0.183030669 | 0.240074039 |
| DRG2 | blue | -0.114789736 | 0.463574225 |
| DROSHA | blue | -0.032902337 | 0.834093853 |
| DSC1 | blue | -0.148873066 | 0.340699973 |
| DSC3 | blue | 0.335664251 | 0.02777002 |
| DSCC1 | blue | 0.11283681 | 0.471264684 |
| DSE | blue | 0.120606292 | 0.441073204 |
| DSTYK | blue | 0.171166938 | 0.2724377 |
| DTD2 | blue | -0.240222249 | 0.120747444 |
| DTWD1 | blue | 0.207422741 | 0.181990811 |
| DTX1 | blue | -0.122834724 | 0.432615131 |
| DTX3L | lightyellow | 0.383089838 | 0.011224846 |
| DUSP13 | blue | 0.059024056 | 0.706939524 |
| DUSP18 | blue | 0.201599406 | 0.194845082 |
| DUSP23 | blue | -0.337094482 | 0.027071524 |
| DUSP26 | blue | -0.278736549 | 0.070296094 |
| DUXA | blue | -0.130201575 | 0.405306747 |
| DYDC2 | blue | -0.015183924 | 0.923012956 |
| DYNAP | blue | 0.00805318 | 0.959123994 |
| DYNC2H1 | blue | 0.062281331 | 0.691546632 |
| DYNC2LI1 | blue | -0.240517473 | 0.120277738 |
| DYNLL2 | blue | -0.113135004 | 0.470086057 |
| DYNLT1 | blue | 0.21106909 | 0.174257017 |
| DYSF | blue | 0.341292386 | 0.025105157 |
| DZIP1 | blue | 0.053134564 | 0.735062746 |
| E2F2 | blue | 0.040781133 | 0.795135571 |
| E2F3 | blue | 0.185104845 | 0.234695945 |
| E2F6 | blue | 0.492569927 | 0.000792423 |
| E2F7 | blue | 0.131272405 | 0.401421632 |
| EARS2 | blue | -0.147446218 | 0.345399901 |
| EBF1 | blue | 0.337044814 | 0.027095533 |
| EBF3 | blue | -0.289815888 | 0.059412158 |
| EBLN1 | blue | 0.286517364 | 0.062501428 |
| EBPL | blue | -0.085488662 | 0.585707232 |
| ECE1 | blue | 0.512343926 | 0.000444209 |
| ECEL1 | blue | 0.033821025 | 0.829529066 |
| ECH1 | blue | -0.310851493 | 0.042468928 |
| ECI1 | blue | -0.290442852 | 0.058839004 |
| ECT2 | blue | 0.261436822 | 0.090373106 |
| ECT2L | blue | -0.119756001 | 0.444324385 |
| EDA | blue | -0.245409312 | 0.112693766 |
| EDARADD | blue | -0.414567171 | 0.005710179 |
| EDC3 | blue | 0.252341456 | 0.102575402 |
| EDDM3A | blue | -0.247688629 | 0.109286551 |
| EDIL3 | lightcyan | -0.344583513 | 0.023648094 |
| EDN1 | blue | 0.232405926 | 0.133692117 |
| EDN2 | blue | -0.193347469 | 0.214138471 |
| EDN3 | blue | 0.231196207 | 0.135784533 |
| EDNRA | blue | 0.024753843 | 0.874801408 |
| EDNRB | blue | 0.247826207 | 0.109083432 |
| EEF1A1 | blue | -0.133078307 | 0.394918565 |
| EEF1G | blue | -0.14932347 | 0.339224605 |
| EEF2 | blue | -0.280036011 | 0.068943453 |
| EEF2K | lightcyan | 0.226287211 | 0.144525796 |
| EEPD1 | blue | -0.203356798 | 0.190900147 |
| EFCAB1 | blue | 0.056115557 | 0.720782625 |
| EFCAB11 | blue | 0.278813875 | 0.070215022 |
| EFCAB13 | blue | 0.163403891 | 0.295102252 |
| EFCAB2 | blue | 0.016533575 | 0.916193117 |
| EFCC1 | blue | 0.103002105 | 0.511001725 |
| EFHB | blue | 0.063658395 | 0.685075353 |
| EFHD1 | blue | -0.22780262 | 0.141784205 |
| EFNA1 | blue | -0.231859001 | 0.134635123 |
| EFNA3 | blue | -0.121825039 | 0.436436165 |
| EFR3B | blue | -0.063062079 | 0.687874943 |
| EGF | blue | 0.116031332 | 0.458720157 |
| EGFL8 | blue | 0.324603269 | 0.033693075 |
| EGFLAM | blue | 0.206259141 | 0.184509587 |
| EGLN1 | blue | -0.482014833 | 0.001064329 |
| EGLN2 | blue | 0.014512824 | 0.92640611 |
| EI24 | blue | -0.12591559 | 0.421071944 |
| EID2 | blue | -0.322517248 | 0.034919058 |
| EIF1B | blue | -0.416786131 | 0.005431311 |
| EIF2A | blue | -0.151484987 | 0.33219927 |
| EIF2AK2 | lightyellow | 0.255648005 | 0.098001306 |
| EIF2AK4 | blue | 0.317080215 | 0.038287671 |
| EIF2B2 | blue | 0.235466809 | 0.128505164 |
| EIF2D | blue | -0.008152596 | 0.958619813 |
| EIF3C | blue | -0.249740904 | 0.106286336 |
| EIF3CL | blue | -0.245480497 | 0.112586151 |
| EIF3E | blue | -0.215395714 | 0.165390401 |
| EIF3F | blue | -0.159611971 | 0.306602452 |
| EIF3G | blue | -0.275534576 | 0.073718633 |
| EIF3I | blue | -0.182374847 | 0.24179177 |
| EIF4E | blue | 0.056735916 | 0.717822427 |
| EIF4E1B | blue | 0.024294513 | 0.87710664 |
| EIF4E2 | blue | -0.155858504 | 0.318263696 |
| EIF4EBP1 | blue | -0.199312176 | 0.200065135 |
| EIF4EBP2 | blue | -0.306741873 | 0.045425885 |
| EIF5A | blue | 0.043150678 | 0.783509915 |
| ELAC2 | blue | -0.001724628 | 0.991242682 |
| ELF1 | blue | -0.061600218 | 0.694755479 |
| ELF4 | blue | 0.05305005 | 0.735468916 |
| ELFN1 | blue | 0.085159938 | 0.587154788 |
| ELK3 | lightcyan | 0.619437153 | 9.48E-06 |
| ELMO1 | lightcyan | 0.499201562 | 0.000655147 |
| ELMO3 | blue | -0.088572776 | 0.572204541 |
| ELOVL2 | blue | 0.210302954 | 0.17586202 |
| ELOVL3 | blue | -0.067540526 | 0.666952641 |
| ELP2 | blue | 0.103237526 | 0.510031227 |
| ELP4 | blue | -0.4710754 | 0.001430947 |
| ELP5 | blue | -0.140155908 | 0.370029805 |
| EMB | blue | 0.453607808 | 0.00225077 |
| EMC9 | blue | -0.041808941 | 0.790087315 |
| EMD | blue | -0.424225513 | 0.004580866 |
| EMID1 | blue | 0.053130616 | 0.735081716 |
| EMILIN1 | blue | 0.037339611 | 0.812098315 |
| EMILIN3 | blue | 0.175822151 | 0.259412073 |
| EN2 | blue | -0.035799966 | 0.819715066 |
| ENAM | blue | -0.175519859 | 0.260245075 |
| ENDOG | blue | -0.445690699 | 0.002742599 |
| ENDOV | blue | -0.407137921 | 0.00673629 |
| ENG | lightyellow | 0.174472503 | 0.263144958 |
| ENHO | blue | 0.035833842 | 0.819547302 |
| ENKD1 | blue | -0.411814192 | 0.006073371 |
| ENO3 | blue | 0.147242757 | 0.346073311 |
| ENPP5 | blue | -0.138340452 | 0.376322647 |
| ENTPD1 | blue | 0.372053469 | 0.01402058 |
| ENTPD2 | blue | -0.385898959 | 0.010594625 |
| ENTPD7 | blue | 0.142027081 | 0.363610156 |
| EPB41L4A | blue | 0.293692978 | 0.055938293 |
| EPHA10 | blue | 0.081841534 | 0.601856015 |
| EPHA2 | blue | 0.248773687 | 0.107692376 |
| EPHA4 | blue | 0.351619044 | 0.020769067 |
| EPHA7 | blue | -0.594503393 | 2.63E-05 |
| EPHA8 | blue | -0.188880307 | 0.225118784 |
| EPHB1 | blue | 0.190118934 | 0.222036186 |
| EPHB4 | lightcyan | 0.499030628 | 0.000658399 |
| EPM2A | blue | -0.43476669 | 0.003575342 |
| EPN2 | blue | 0.138724075 | 0.37498764 |
| EPN3 | blue | -0.229949214 | 0.137966807 |
| EPO | blue | 0.047097753 | 0.764248519 |
| EPPIN | blue | -0.088461316 | 0.57269004 |
| EPPK1 | blue | -0.214994808 | 0.166197964 |
| EPS8L3 | blue | 0.006507668 | 0.966963808 |
| EPSTI1 | lightyellow | 0.400853249 | 0.007725231 |
| EPX | blue | -0.111834401 | 0.475238239 |
| ERAL1 | blue | -0.211866663 | 0.172597372 |
| ERAS | blue | 0.072523247 | 0.643964732 |
| ERCC2 | blue | -0.329869902 | 0.03075474 |
| ERCC6L2 | blue | 0.336951022 | 0.027140921 |
| ERCC8 | blue | 0.018824925 | 0.904628675 |
| ERF | blue | -0.193687335 | 0.213318581 |
| ERG | lightcyan | 0.447441799 | 0.002626348 |
| ERGIC3 | blue | -0.11794356 | 0.451298204 |
| ERH | blue | 0.194767261 | 0.210727867 |
| ERI1 | blue | -0.046294625 | 0.768156766 |
| ERICH1 | blue | 0.079399072 | 0.612777591 |
| ERMAP | blue | -0.327530437 | 0.032032691 |
| ERMN | blue | 0.118146662 | 0.450513764 |
| ERN2 | blue | -0.020467895 | 0.896348446 |
| ERP27 | blue | -0.319958002 | 0.036473009 |
| ERP29 | blue | -0.143785935 | 0.357637439 |
| ERVFRD-1 | blue | -0.062159903 | 0.692118313 |
| ERVV-1 | blue | 0.004824682 | 0.975504455 |
| ESAM | lightcyan | 0.502862098 | 0.000588865 |
| ESCO1 | blue | 0.078801846 | 0.615460883 |
| ESPNL | blue | -0.103591118 | 0.508575335 |
| ESR1 | blue | -0.183825721 | 0.238002765 |
| ESRP2 | lightcyan | -0.428631048 | 0.00413404 |
| ESX1 | lightyellow | 0.055465156 | 0.723890504 |
| ETAA1 | blue | -0.129781385 | 0.406837142 |
| ETFB | blue | -0.310528918 | 0.04269521 |
| ETV3 | blue | 0.145118372 | 0.353152584 |
| ETV6 | blue | 0.328863361 | 0.031299308 |
| EVI5L | blue | -0.168521852 | 0.28002754 |
| EVX1 | blue | -0.065504674 | 0.67643395 |
| EXOC4 | blue | 0.288569188 | 0.060565104 |
| EXOC8 | blue | 0.16638927 | 0.286246559 |
| EXTL2 | blue | -0.035383409 | 0.821778659 |
| EYA1 | blue | -0.197379958 | 0.204550943 |
| F13B | blue | -0.151439851 | 0.332345039 |
| F2RL2 | blue | 0.172208305 | 0.269487137 |
| F8 | blue | 0.283675414 | 0.065264467 |
| FA2H | blue | -0.299300782 | 0.05120383 |
| FAAH | blue | -0.196020279 | 0.207749471 |
| FABP12 | blue | -0.028173023 | 0.857675047 |
| FABP5 | blue | 0.433087594 | 0.003721246 |
| FABP7 | blue | -0.011212455 | 0.943110771 |
| FAF1 | blue | -0.012680786 | 0.935675482 |
| FAF2 | blue | 0.047584623 | 0.761882076 |
| FAHD2A | blue | -0.481747879 | 0.00107217 |
| FAIM2 | blue | 0.219884989 | 0.156540883 |
| FAM102B | blue | 0.279482912 | 0.069516649 |
| FAM104B | blue | -0.402922227 | 0.007386673 |
| FAM107A | lightcyan | 0.331337725 | 0.029974662 |
| FAM110A | lightcyan | 0.291895875 | 0.057527676 |
| FAM110C | blue | -0.016508745 | 0.916318531 |
| FAM110D | blue | 0.105162101 | 0.502132372 |
| FAM111A | blue | 0.03342291 | 0.831506549 |
| FAM117A | blue | -0.03311017 | 0.833060689 |
| FAM118A | blue | 0.356734105 | 0.018865742 |
| FAM124B | blue | -0.314876464 | 0.039726705 |
| FAM126B | blue | -0.001650798 | 0.991617564 |
| FAM131A | blue | 0.115482215 | 0.460863562 |
| FAM133A | blue | 0.023582625 | 0.880681349 |
| FAM136A | blue | -0.172192271 | 0.269532404 |
| FAM156B | blue | 0.186077625 | 0.232202163 |
| FAM160A2 | blue | 0.385290446 | 0.010728511 |
| FAM160B1 | blue | 0.513244252 | 0.000432292 |
| FAM160B2 | blue | 0.15835252 | 0.310484521 |
| FAM161B | blue | 0.125383902 | 0.423051514 |
| FAM163B | blue | -0.09696628 | 0.536196923 |
| FAM170B | blue | 0.124564918 | 0.42611098 |
| FAM171A2 | blue | -0.075674234 | 0.629593359 |
| FAM177A1 | lightcyan | 0.307367274 | 0.044965414 |
| FAM181A | blue | -0.372796834 | 0.013815277 |
| FAM184B | blue | 0.150426526 | 0.335628081 |
| FAM193A | blue | 0.355795703 | 0.019203483 |
| FAM199X | blue | -0.254628165 | 0.099395038 |
| FAM20A | blue | 0.232853226 | 0.132924548 |
| FAM214A | blue | 0.210995592 | 0.174410532 |
| FAM214B | blue | 0.271448137 | 0.078275558 |
| FAM220A | blue | -0.242054497 | 0.117854527 |
| FAM221B | blue | 0.195716257 | 0.20846941 |
| FAM227B | blue | -0.13564161 | 0.385794192 |
| FAM228A | blue | 0.146410361 | 0.348836736 |
| FAM24A | blue | -0.102244943 | 0.514129319 |
| FAM32A | blue | -0.393634331 | 0.009013439 |
| FAM3A | blue | -0.340995679 | 0.025240115 |
| FAM3C | blue | 0.063457964 | 0.686015878 |
| FAM47C | blue | 0.091235323 | 0.560663388 |
| FAM50B | blue | -0.382146035 | 0.011443655 |
| FAM53B | blue | -0.168700532 | 0.279510521 |
| FAM71B | blue | -0.123765671 | 0.429108661 |
| FAM71C | blue | -0.041439721 | 0.791899839 |
| FAM71E1 | blue | -0.126919498 | 0.417348523 |
| FAM71E2 | blue | -0.081593776 | 0.602960014 |
| FAM71F2 | blue | 0.311404603 | 0.042083204 |
| FAM78B | blue | -0.024047577 | 0.878346344 |
| FAM81B | blue | -0.057727386 | 0.713099855 |
| FAM83C | blue | -0.405429868 | 0.006993573 |
| FAM83F | blue | -0.25486088 | 0.099075673 |
| FAM83G | blue | -0.135762684 | 0.385366306 |
| FAM90A10P | blue | -0.108294815 | 0.489409649 |
| FAM90A14P | lightyellow | 0.1541816 | 0.323562682 |
| FAM9C | blue | 0.019398914 | 0.901734712 |
| FANCD2OS | blue | 0.138772033 | 0.374820945 |
| FANCF | blue | -0.322212851 | 0.035100984 |
| FANCG | blue | 0.04265319 | 0.785946963 |
| FANCI | blue | 0.141124067 | 0.366699811 |
| FAR1 | blue | 0.325737707 | 0.033041369 |
| FARP2 | blue | 0.193553241 | 0.213641807 |
| FARSA | blue | -0.163244774 | 0.295579158 |
| FAS | blue | 0.426557478 | 0.004339369 |
| FASLG | blue | 0.034467794 | 0.826318699 |
| FASTK | blue | -0.087241571 | 0.578015264 |
| FAT2 | blue | -0.004872448 | 0.975262016 |
| FBLIM1 | blue | 0.366650201 | 0.015592205 |
| FBP2 | blue | -0.20473424 | 0.187847927 |
| FBXL13 | blue | 0.089540793 | 0.567995994 |
| FBXL14 | blue | 0.075980304 | 0.628204458 |
| FBXL16 | blue | -0.147946518 | 0.343747438 |
| FBXL19 | blue | -0.062597518 | 0.690058828 |
| FBXL5 | blue | -0.226641793 | 0.143880828 |
| FBXO11 | blue | 0.31197832 | 0.041686129 |
| FBXO15 | blue | -0.160655476 | 0.303409567 |
| FBXO17 | blue | -0.46763444 | 0.001567442 |
| FBXO22 | blue | 0.125069497 | 0.424224567 |
| FBXO4 | blue | -0.050097287 | 0.749703723 |
| FBXO40 | blue | 0.14214331 | 0.363213622 |
| FBXO41 | blue | 0.214653452 | 0.166887821 |
| FBXO44 | blue | -0.219760626 | 0.156781279 |
| FBXO45 | blue | -0.036578546 | 0.815861272 |
| FBXO48 | blue | -0.259967618 | 0.092264105 |
| FBXO5 | lightyellow | 0.226852835 | 0.143497964 |
| FBXO9 | blue | 0.011452694 | 0.941893913 |
| FBXW10 | blue | -0.148427764 | 0.342162508 |
| FBXW2 | blue | 0.363615732 | 0.016538503 |
| FBXW7 | blue | 0.214760571 | 0.166671118 |
| FBXW9 | blue | -0.510432556 | 0.000470494 |
| FCHSD1 | blue | 0.248275862 | 0.108421568 |
| FCRL1 | blue | 0.150130169 | 0.336592025 |
| FCRL2 | blue | -0.176656461 | 0.257122269 |
| FCRL4 | blue | -0.218904887 | 0.158442762 |
| FCRL6 | blue | 0.013395949 | 0.932055979 |
| FDXR | blue | -0.006592042 | 0.966535726 |
| FECH | blue | -0.471014413 | 0.001433271 |
| FER | blue | 0.066228653 | 0.673056502 |
| FETUB | blue | -0.167929559 | 0.281745843 |
| FEZ1 | lightcyan | 0.31996879 | 0.036466342 |
| FEZ2 | blue | 0.262332118 | 0.089235587 |
| FGA | blue | -0.128750631 | 0.410605336 |
| FGB | blue | -0.044371049 | 0.777540363 |
| FGD3 | blue | -0.06363652 | 0.685177982 |
| FGD5 | blue | 0.404157901 | 0.007190647 |
| FGF10 | blue | 0.058323055 | 0.710267637 |
| FGF11 | blue | -0.190755392 | 0.220463592 |
| FGF14 | blue | -0.111829633 | 0.475257182 |
| FGF18 | blue | -0.0226387 | 0.885424825 |
| FGF2 | blue | 0.019757252 | 0.899928661 |
| FGF21 | blue | -0.069200999 | 0.659257196 |
| FGF22 | blue | 0.127122587 | 0.41659756 |
| FGF23 | blue | -0.286646825 | 0.062377816 |
| FGF7 | blue | -0.016038483 | 0.918694169 |
| FGF9 | blue | -0.397317229 | 0.008334872 |
| FGFBP3 | blue | -0.356239081 | 0.019043278 |
| FGFR1OP2 | blue | 0.215066692 | 0.166052955 |
| FGFR3 | blue | -0.004890768 | 0.975169031 |
| FGFRL1 | blue | -0.36689003 | 0.015519411 |
| FGL1 | blue | 0.062700834 | 0.689572926 |
| FHL5 | blue | 0.30342148 | 0.047935118 |
| FHOD1 | blue | 0.437914284 | 0.00331528 |
| FIBCD1 | blue | -0.16470427 | 0.291223383 |
| FIBP | blue | 0.004690565 | 0.976185189 |
| FIGLA | blue | 0.081960472 | 0.60132634 |
| FIGNL2 | blue | -0.091578945 | 0.559181865 |
| FITM1 | blue | -0.008982211 | 0.95441314 |
| FKBP11 | lightcyan | 0.349860463 | 0.02145958 |
| FKBP1A | lightcyan | 0.459204497 | 0.001951736 |
| FKBP1B | blue | 0.009660911 | 0.95097261 |
| FKBP4 | blue | -0.279916726 | 0.069066756 |
| FLAD1 | blue | 0.206286696 | 0.184449655 |
| FLCN | blue | -0.15510924 | 0.320624553 |
| FLG | blue | -0.058117729 | 0.711243466 |
| FLI1 | lightcyan | 0.56447785 | 8.05E-05 |
| FLOT2 | blue | 0.120272512 | 0.442347878 |
| FLVCR1 | blue | 0.372630896 | 0.013860883 |
| FMNL3 | lightcyan | 0.561423156 | 8.97E-05 |
| FMO2 | blue | -0.225112877 | 0.146677086 |
| FNDC3A | lightcyan | 0.344207796 | 0.023810776 |
| FNDC5 | blue | -0.244843429 | 0.11355203 |
| FNDC7 | blue | 0.002415361 | 0.987735506 |
| FNDC9 | blue | -0.078656547 | 0.616114455 |
| FOXD4L1 | blue | -0.23153076 | 0.135203447 |
| FOXD4L6 | blue | 0.161872776 | 0.299711896 |
| FOXI2 | blue | -0.207215838 | 0.182436877 |
| FOXM1 | blue | 0.373737811 | 0.013559067 |
| FOXN1 | blue | -0.179770982 | 0.248693878 |
| FOXN4 | blue | 0.100738585 | 0.520379978 |
| FOXO6 | blue | -0.363796083 | 0.016480934 |
| FOXP2 | blue | -0.25624754 | 0.097189015 |
| FOXP3 | blue | 0.150311594 | 0.33600171 |
| FOXQ1 | blue | -0.355376136 | 0.019356128 |
| FOXR1 | blue | -0.137197484 | 0.380316886 |
| FOXRED1 | blue | -0.183850996 | 0.237937117 |
| FOXRED2 | blue | -0.13469327 | 0.389155368 |
| FRAS1 | blue | -0.214598465 | 0.166999137 |
| FRMD6 | blue | 0.394134029 | 0.008918674 |
| FSCB | blue | -0.164999847 | 0.29034634 |
| FSCN1 | blue | 0.375372221 | 0.01312365 |
| FSHB | blue | 0.041975338 | 0.789270817 |
| FSHR | blue | -0.015119907 | 0.923336573 |
| FSTL1 | blue | -0.280880933 | 0.068075056 |
| FTCDNL1 | blue | -0.062627388 | 0.689918334 |
| FTSJ3 | blue | 0.067871321 | 0.66541685 |
| FUT1 | blue | 0.304456508 | 0.047141196 |
| FUT10 | blue | 0.109238614 | 0.485609686 |
| FUT2 | blue | 0.135389956 | 0.386684455 |
| FYTTD1 | blue | 0.009041424 | 0.954112939 |
| FZD4 | blue | 0.28787773 | 0.061212219 |
| G0S2 | blue | -0.255627714 | 0.098028889 |
| G6PC2 | blue | 0.128713024 | 0.410743195 |
| GAA | blue | -0.230836515 | 0.136411353 |
| GAB3 | blue | 0.335961562 | 0.027623601 |
| GABARAPL1 | blue | -0.326842741 | 0.032416576 |
| GABPA | blue | 0.187396491 | 0.228850175 |
| GABRA3 | blue | -0.343926121 | 0.023933351 |
| GABRA4 | blue | 0.011619507 | 0.941049048 |
| GABRA5 | blue | -0.078884177 | 0.615090682 |
| GABRA6 | blue | -0.133023169 | 0.395116203 |
| GABRD | blue | 0.204491928 | 0.188382327 |
| GABRQ | blue | 0.199954909 | 0.198588423 |
| GAD1 | blue | -0.199876724 | 0.198767645 |
| GADD45G | blue | -0.409338899 | 0.006416861 |
| GADD45GIP1 | blue | 0.013543467 | 0.931309546 |
| GADL1 | blue | -0.175887336 | 0.25923268 |
| GAGE1 | blue | 0.302439048 | 0.048698669 |
| GAGE13 | blue | -0.13848005 | 0.37583652 |
| GAGE2E | blue | -0.209150072 | 0.178297192 |
| GAK | blue | -0.101551036 | 0.517004019 |
| GAL3ST2 | blue | -0.032405233 | 0.836566125 |
| GALK2 | blue | 0.013959665 | 0.929203915 |
| GALNT5 | blue | 0.066046204 | 0.673907052 |
| GALP | blue | 0.249335546 | 0.106873889 |
| GALR3 | blue | -0.217870966 | 0.160467275 |
| GALT | blue | 0.031170325 | 0.842714437 |
| GAN | blue | 0.161165595 | 0.30185649 |
| GAPDH | blue | -0.147967224 | 0.343679153 |
| GAPDHS | blue | -0.009389738 | 0.952347162 |
| GAPVD1 | blue | 0.392632228 | 0.009206093 |
| GART | blue | 0.115710593 | 0.459971464 |
| GAST | blue | -0.074836522 | 0.633401213 |
| GATA1 | blue | -0.242435774 | 0.117259171 |
| GATA3 | blue | -0.151810331 | 0.331149735 |
| GATA5 | blue | -0.024629619 | 0.875424752 |
| GATAD2A | blue | 0.079746445 | 0.611219161 |
| GBP1 | lightyellow | 0.419263163 | 0.005134059 |
| GBP2 | lightyellow | 0.428334337 | 0.00416289 |
| GBP3 | lightyellow | 0.35780191 | 0.018487523 |
| GBP4 | lightyellow | 0.127079826 | 0.416755613 |
| GBP7 | lightyellow | 0.225534243 | 0.145902479 |
| GBX2 | blue | 0.348678531 | 0.0219344 |
| GC | blue | -0.193131959 | 0.214659497 |
| GCGR | blue | -0.474374377 | 0.001310094 |
| GCH1 | blue | 0.269044477 | 0.081057309 |
| GCLM | blue | -0.262523447 | 0.08899394 |
| GCM2 | blue | -0.116095962 | 0.45846824 |
| GCNT3 | blue | -0.302724002 | 0.048476196 |
| GCNT7 | blue | 0.136710248 | 0.382027183 |
| GCSAM | blue | 0.038603219 | 0.805859871 |
| GDAP1 | blue | -0.194384877 | 0.211642679 |
| GDAP1L1 | blue | 0.162314472 | 0.298377387 |
| GDF3 | blue | -0.083082262 | 0.596340624 |
| GDI1 | blue | 0.015375756 | 0.922043276 |
| GDPGP1 | blue | 0.068669182 | 0.661718162 |
| GEM | blue | -0.178459072 | 0.252221148 |
| GEMIN6 | blue | -0.04472646 | 0.775804196 |
| GFER | blue | -0.087852438 | 0.575345506 |
| GFM2 | blue | -0.134422167 | 0.390119377 |
| GFRA2 | blue | 0.0986742 | 0.529006996 |
| GFRA3 | blue | 0.053512433 | 0.7332476 |
| GGA2 | blue | -0.059977484 | 0.70242163 |
| GGCT | blue | -0.221399139 | 0.153635536 |
| GGN | blue | -0.017038402 | 0.913643702 |
| GHITM | blue | -0.32357314 | 0.034293991 |
| GHRH | lightcyan | -0.232989125 | 0.132691997 |
| GIGYF2 | blue | 0.017547191 | 0.911075148 |
| GIMAP4 | blue | 0.420447407 | 0.004997017 |
| GIMAP6 | blue | 0.421477118 | 0.004880457 |
| GIMD1 | blue | 0.087224834 | 0.578088492 |
| GINM1 | blue | -0.061648692 | 0.694526933 |
| GINS4 | blue | -0.054297416 | 0.72948143 |
| GIPC1 | blue | -0.307366011 | 0.04496634 |
| GIPC3 | blue | 0.105633434 | 0.500207467 |
| GIT2 | blue | 0.426346157 | 0.004360786 |
| GJA10 | blue | 0.100618703 | 0.520879046 |
| GJA4 | blue | 0.475276294 | 0.001278672 |
| GJA8 | blue | 0.243419081 | 0.115734245 |
| GJB5 | blue | -0.107739382 | 0.491653135 |
| GJB6 | blue | -0.024375252 | 0.876701363 |
| GJC1 | blue | 0.345436996 | 0.023281991 |
| GJC3 | blue | -0.009290005 | 0.952852737 |
| GJD2 | blue | -0.126764028 | 0.417923925 |
| GJD3 | lightyellow | 0.442650771 | 0.002955184 |
| GJD4 | blue | -0.123904341 | 0.428587715 |
| GK2 | lightcyan | -0.19399374 | 0.212581285 |
| GLB1 | blue | -0.166348257 | 0.286367034 |
| GLB1L | blue | 0.056765472 | 0.717681496 |
| GLB1L3 | blue | -0.017613429 | 0.910740822 |
| GLCCI1 | blue | 0.219531163 | 0.157225545 |
| GLI3 | blue | -0.211311589 | 0.173751198 |
| GLRB | blue | -0.195461013 | 0.209075182 |
| GLRX3 | lightcyan | 0.345272926 | 0.023351999 |
| GLRX5 | blue | -0.353740635 | 0.019960926 |
| GLT8D2 | blue | 0.163071942 | 0.296097729 |
| GLTP | blue | -0.336576963 | 0.027322565 |
| GMCL1 | blue | -0.549070599 | 0.000137402 |
| GMNN | blue | -0.200997061 | 0.196210362 |
| GMPR | blue | 0.146297783 | 0.349211511 |
| GMPR2 | blue | -0.308860398 | 0.043881366 |
| GNAI1 | blue | -0.039869456 | 0.79962036 |
| GNAO1 | blue | 0.078636909 | 0.616202809 |
| GNAS | blue | 0.356504872 | 0.01894778 |
| GNAT1 | blue | 0.063573105 | 0.685475519 |
| GNB4 | blue | 0.540625877 | 0.000182136 |
| GNB5 | blue | -0.262638709 | 0.08884861 |
| GNG4 | blue | 0.066802715 | 0.670382936 |
| GNGT2 | blue | 0.425695364 | 0.004427322 |
| GNL1 | blue | 0.231449264 | 0.135344826 |
| GNL3 | blue | -0.241501125 | 0.118722667 |
| GNL3L | blue | 0.19809561 | 0.202881355 |
| GNPDA2 | blue | -0.125304058 | 0.42334924 |
| GNRH1 | blue | 0.140489866 | 0.368879115 |
| GNRH2 | blue | -0.156228542 | 0.317101798 |
| GNRHR | blue | 0.457111606 | 0.002059177 |
| GOLGA4 | blue | -0.119023768 | 0.447134676 |
| GOLGA7B | blue | 0.183514821 | 0.238811273 |
| GOLT1A | blue | -0.28813151 | 0.060974076 |
| GOPC | blue | 0.210597956 | 0.175242759 |
| GORAB | blue | -0.044337614 | 0.777703747 |
| GORASP1 | blue | 0.02389749 | 0.87909998 |
| GPA33 | blue | -0.079450178 | 0.612548204 |
| GPAM | blue | -0.340253673 | 0.025580265 |
| GPANK1 | blue | -0.319365887 | 0.0368405 |
| GPAT2 | blue | -0.062366095 | 0.691147665 |
| GPATCH2 | blue | 0.348070606 | 0.022182029 |
| GPATCH3 | blue | -0.129815807 | 0.406711647 |
| GPATCH8 | blue | 0.038597839 | 0.805886409 |
| GPBAR1 | blue | 0.159195111 | 0.307883917 |
| GPC5 | blue | -0.090721505 | 0.562882122 |
| GPCPD1 | blue | 0.038701157 | 0.805376842 |
| GPHN | blue | -0.275124079 | 0.074166731 |
| GPI | blue | -0.284853686 | 0.064107372 |
| GPN1 | blue | -0.071689369 | 0.647789804 |
| GPN2 | blue | -0.124994829 | 0.424503423 |
| GPR135 | blue | 0.068728441 | 0.661443768 |
| GPR137C | blue | 0.112432294 | 0.47286606 |
| GPR139 | blue | 0.215894316 | 0.164390015 |
| GPR142 | blue | 0.084481653 | 0.590146689 |
| GPR148 | blue | 0.066652468 | 0.671082291 |
| GPR149 | blue | -0.076721068 | 0.624848232 |
| GPR150 | blue | 0.028028346 | 0.858398473 |
| GPR151 | blue | 0.071692772 | 0.647774177 |
| GPR157 | blue | -0.478982438 | 0.00115647 |
| GPR161 | blue | 0.103366642 | 0.509499359 |
| GPR162 | blue | -0.060401607 | 0.700415127 |
| GPR17 | blue | 0.080906942 | 0.606025104 |
| GPR180 | blue | 0.306700222 | 0.045456688 |
| GPR182 | blue | -0.244071053 | 0.114731467 |
| GPR25 | blue | -0.247698621 | 0.109271788 |
| GPR31 | blue | -0.014250014 | 0.927735262 |
| GPR32 | blue | 0.164290471 | 0.292454098 |
| GPR42 | blue | -0.193863242 | 0.212895084 |
| GPR55 | blue | -0.095372792 | 0.542947543 |
| GPR6 | blue | 0.126797836 | 0.417798761 |
| GPR61 | blue | -0.127918322 | 0.413662572 |
| GPR62 | blue | -0.009662173 | 0.950966215 |
| GPR75 | blue | 0.18077001 | 0.246030222 |
| GPR78 | blue | -0.181169863 | 0.244969529 |
| GPR83 | blue | 0.064234795 | 0.682373223 |
| GPR85 | blue | -0.057837725 | 0.712574939 |
| GPRASP2 | blue | -0.279167379 | 0.069845331 |
| GPRC5B | blue | -0.281992014 | 0.066946311 |
| GPRC5D | blue | 0.313287176 | 0.040791681 |
| GPRIN2 | blue | 0.139725282 | 0.371516741 |
| GPRIN3 | blue | 0.059122263 | 0.706473698 |
| GPS1 | blue | -0.317966251 | 0.037721253 |
| GPSM2 | blue | 0.146546548 | 0.348383696 |
| GPX4 | blue | -0.215210633 | 0.165762863 |
| GRAMD4 | blue | -0.133114991 | 0.394787103 |
| GRAPL | blue | 0.143114155 | 0.359911615 |
| GRB7 | blue | -0.366639344 | 0.015595507 |
| GRIA4 | blue | 0.048554091 | 0.757176358 |
| GRID2IP | blue | 0.013353359 | 0.932271496 |
| GRIK1 | blue | 0.245097403 | 0.113166222 |
| GRIK2 | blue | 0.114382547 | 0.465172124 |
| GRIK4 | blue | 0.043335505 | 0.78260502 |
| GRIN2A | blue | -0.171419206 | 0.271720987 |
| GRIN2C | blue | -0.090406361 | 0.564244973 |
| GRK6 | blue | -0.16131675 | 0.301397275 |
| GRM1 | blue | -0.262569608 | 0.088935715 |
| GRM2 | lightcyan | -0.070817885 | 0.651796968 |
| GRM3 | blue | 0.058979203 | 0.707152312 |
| GRM5 | blue | 0.006769418 | 0.965635813 |
| GRM6 | blue | -0.013474693 | 0.931657531 |
| GRP | blue | -0.264270355 | 0.086811017 |
| GRPEL2 | blue | 0.422432984 | 0.004774383 |
| GRWD1 | blue | 0.04668161 | 0.766272874 |
| GRXCR1 | blue | 0.129441456 | 0.408077641 |
| GSDMC | blue | -0.010872326 | 0.944833813 |
| GSE1 | blue | 0.255133265 | 0.098702869 |
| GSTK1 | blue | -0.264547181 | 0.086468951 |
| GSTM1 | blue | -0.113999386 | 0.466678419 |
| GSTM2 | blue | -0.087406724 | 0.577292921 |
| GSTM5 | blue | -0.05754746 | 0.713956099 |
| GSTO2 | blue | -0.250955687 | 0.104540313 |
| GSTT2 | blue | -0.067914599 | 0.66521602 |
| GTF2E1 | blue | 0.356048546 | 0.019111985 |
| GTF2H2 | blue | 0.135885242 | 0.384933461 |
| GTF2H4 | blue | -0.019395414 | 0.901752351 |
| GTF3A | blue | 0.044989086 | 0.774521973 |
| GTF3C3 | lightcyan | 0.224332412 | 0.148119824 |
| GTF3C5 | blue | -0.16340669 | 0.295093868 |
| GTPBP10 | blue | -0.058867629 | 0.707681723 |
| GTPBP2 | blue | 0.26092954 | 0.091022601 |
| GTSE1 | blue | 0.126086159 | 0.420437997 |
| GTSF1 | blue | -0.13666556 | 0.382184274 |
| GTSF1L | blue | 0.030245706 | 0.847323969 |
| GUK1 | blue | -0.121227417 | 0.438706584 |
| GYG1 | blue | -0.02478663 | 0.874636903 |
| GYG2 | blue | -0.305969999 | 0.045999469 |
| GYS1 | blue | -0.13808378 | 0.377217441 |
| GZMM | blue | 0.252690503 | 0.102084968 |
| HABP4 | blue | -0.078054048 | 0.618827678 |
| HACL1 | blue | 0.147938687 | 0.343773266 |
| HADHA | blue | -0.211045731 | 0.174305795 |
| HAND1 | blue | -0.1762457 | 0.25824793 |
| HAP1 | blue | -0.168719969 | 0.279454315 |
| HAPLN3 | blue | 0.121197288 | 0.438821219 |
| HAPLN4 | blue | -0.030540052 | 0.845856005 |
| HARS2 | blue | -0.0006064 | 0.996920768 |
| HAS1 | blue | -0.183937672 | 0.237712089 |
| HBEGF | blue | 0.279108372 | 0.069906932 |
| HBP1 | blue | 0.36333174 | 0.016629498 |
| HCAR1 | blue | -0.07021911 | 0.654555821 |
| HCCS | blue | -0.290030024 | 0.059215901 |
| HCFC2 | blue | 0.320812474 | 0.035947995 |
| HCN1 | blue | -0.025915755 | 0.868974762 |
| HCN4 | blue | -0.157976379 | 0.311649953 |
| HCRTR2 | blue | -0.073823539 | 0.638018286 |
| HDAC10 | blue | 0.093700176 | 0.550076933 |
| HDAC11 | blue | -0.365502296 | 0.015944639 |
| HDAC4 | blue | 0.045967472 | 0.769750418 |
| HDAC7 | blue | 0.285407065 | 0.063569597 |
| HDDC3 | blue | -0.24571437 | 0.112233137 |
| HEATR1 | blue | 0.069374652 | 0.658454384 |
| HEATR5B | blue | 0.101884771 | 0.515620428 |
| HEATR6 | blue | -0.206539541 | 0.183900363 |
| HEBP2 | blue | -0.211082051 | 0.174229955 |
| HECA | blue | 0.005334009 | 0.972919447 |
| HEG1 | blue | 0.462189668 | 0.001807063 |
| HELB | blue | -0.199197083 | 0.20033038 |
| HELZ | blue | 0.236301573 | 0.12711707 |
| HEPACAM | blue | -0.032514313 | 0.836023498 |
| HEPH | blue | -0.000538687 | 0.997264606 |
| HEPHL1 | blue | 0.076454115 | 0.626056875 |
| HERC5 | lightyellow | 0.158069654 | 0.311360692 |
| HERC6 | lightyellow | 0.320773428 | 0.03597185 |
| HES3 | blue | -0.160964422 | 0.302468359 |
| HES5 | blue | 0.108408258 | 0.488952085 |
| HES7 | blue | -0.085647593 | 0.585007942 |
| HESX1 | blue | 0.045325599 | 0.772879874 |
| HEXIM2 | blue | -0.213201511 | 0.169845245 |
| HEY1 | blue | 0.242014846 | 0.117916572 |
| HFM1 | blue | -0.003762898 | 0.980894141 |
| HGFAC | blue | -0.347955455 | 0.022229197 |
| HGS | blue | -0.060579486 | 0.699574192 |
| HHATL | blue | 0.182729697 | 0.240861314 |
| HHIPL2 | blue | -0.221385757 | 0.153661039 |
| HIF3A | blue | 0.270638921 | 0.079203581 |
| HIGD1A | blue | 0.022861521 | 0.884304727 |
| HIGD2B | blue | -0.202065626 | 0.193792961 |
| HINFP | blue | 0.003761055 | 0.980903496 |
| HIP1 | blue | 0.306905752 | 0.045304856 |
| HIPK4 | blue | -0.011589721 | 0.941199902 |
| HIVEP3 | blue | 0.279724628 | 0.069265689 |
| HK3 | blue | 0.113186777 | 0.469881581 |
| HLA-DMA | lightyellow | 0.466559367 | 0.00161239 |
| HLA-DMB | blue | 0.375677423 | 0.013043675 |
| HLA-DOA | lightyellow | 0.289613565 | 0.059598064 |
| HLA-DOB | blue | 0.297439656 | 0.052737899 |
| HLA-DPB1 | blue | 0.315525166 | 0.039298592 |
| HLA-DQA2 | lightyellow | 0.289794236 | 0.059432031 |
| HLA-DQB1 | lightcyan | 0.566621461 | 7.46E-05 |
| HLA-DQB2 | blue | 0.278364029 | 0.070687701 |
| HLA-DRA | lightyellow | 0.349812525 | 0.021478669 |
| HLA-DRB5 | blue | -0.153055075 | 0.327153379 |
| HMBOX1 | blue | 0.168994311 | 0.278661813 |
| HMCN2 | blue | 0.177156918 | 0.255755241 |
| HMG20A | blue | 0.301019475 | 0.049819304 |
| HMGCLL1 | blue | 0.044649748 | 0.776178839 |
| HMGCR | blue | 0.203882605 | 0.189730912 |
| HMGCS2 | blue | 0.055566503 | 0.723405942 |
| HMGXB3 | blue | -0.143017577 | 0.360239278 |
| HMHB1 | blue | 0.116224928 | 0.457965769 |
| HMSD | blue | -0.064881082 | 0.679348118 |
| HMX1 | blue | -0.126045637 | 0.420588555 |
| HMX2 | blue | -0.277535521 | 0.071564833 |
| HNRNPH3 | blue | 0.117156568 | 0.454344812 |
| HOXA1 | blue | 0.226007781 | 0.145035567 |
| HOXA11 | blue | 0.303060302 | 0.048214693 |
| HOXA2 | blue | 0.289011951 | 0.060153611 |
| HOXB13 | blue | -0.307096144 | 0.045164575 |
| HOXB2 | blue | -0.135349214 | 0.386828701 |
| HOXB6 | blue | 0.140677499 | 0.368233545 |
| HOXC12 | blue | -0.011877833 | 0.939740824 |
| HOXC13 | blue | 0.108593603 | 0.488204992 |
| HP | blue | 0.191003318 | 0.219853093 |
| HPCAL1 | blue | 0.024140291 | 0.877880853 |
| HPGD | blue | -0.156475684 | 0.316327281 |
| HPS1 | blue | 0.178338718 | 0.252546413 |
| HPX | blue | -0.013690642 | 0.930564905 |
| HRAS | blue | -0.324716777 | 0.033627395 |
| HRC | blue | 0.010110135 | 0.948695837 |
| HRH4 | blue | 0.17628999 | 0.258126397 |
| HS1BP3 | blue | -0.035407141 | 0.821661061 |
| HS2ST1 | blue | 0.007382843 | 0.962523944 |
| HS6ST3 | blue | 0.143623496 | 0.358186544 |
| HSD11B1 | blue | -0.057038337 | 0.716380824 |
| HSD11B1L | blue | 0.163361058 | 0.295230583 |
| HSD17B10 | blue | -0.330235445 | 0.030558919 |
| HSD17B13 | blue | -0.060834327 | 0.698370028 |
| HSD17B2 | blue | -0.427407573 | 0.004254139 |
| HSD17B8 | blue | -0.382526144 | 0.011355097 |
| HSD3B7 | blue | 0.00358745 | 0.981784811 |
| HSFX2 | blue | 0.103425587 | 0.509256638 |
| HSPA12B | lightcyan | 0.388774968 | 0.009981028 |
| HSPA14 | blue | -0.135545227 | 0.386135018 |
| HSPA1L | lightcyan | -0.464963871 | 0.001681203 |
| HSPA8 | blue | -0.064816979 | 0.679647946 |
| HSPB2 | blue | -0.236702894 | 0.126453742 |
| HSPB7 | blue | 0.266021108 | 0.084665274 |
| HSPBAP1 | blue | 0.548861275 | 0.000138378 |
| HSPBP1 | blue | -0.332149864 | 0.029550132 |
| HTR1B | blue | -0.027257603 | 0.862254316 |
| HTR1F | blue | 0.071003884 | 0.650940911 |
| HTR2C | lightcyan | -0.285457652 | 0.063520616 |
| HTR3D | blue | -0.208652189 | 0.179356279 |
| HTR7 | blue | -0.005818081 | 0.970462868 |
| HTRA4 | blue | 0.162660295 | 0.297335214 |
| HUS1 | blue | 0.257426189 | 0.09560722 |
| HVCN1 | blue | 0.228502791 | 0.140530568 |
| HYAL2 | blue | 0.475911974 | 0.00125693 |
| HYAL3 | blue | 0.025481849 | 0.87114989 |
| HYDIN | blue | -0.01229463 | 0.937630389 |
| HYLS1 | blue | 0.042961936 | 0.784434268 |
| IARS2 | blue | -0.143937038 | 0.357127113 |
| IBSP | blue | -0.145504352 | 0.351859838 |
| ICAM5 | blue | -0.447697342 | 0.002609751 |
| ID2 | blue | 0.018183612 | 0.907863521 |
| ID3 | blue | 0.051187681 | 0.744437256 |
| IDE | blue | 0.296928733 | 0.053165444 |
| IDH3A | blue | 0.205292328 | 0.186621209 |
| IDH3B | blue | -0.226543206 | 0.14405994 |
| IDH3G | blue | -0.131733079 | 0.399756887 |
| IDO1 | lightyellow | 0.413467098 | 0.005852981 |
| IER3 | blue | 0.294489106 | 0.055245534 |
| IER5L | blue | -0.209699376 | 0.177133929 |
| IFFO1 | blue | 0.381123873 | 0.011684731 |
| IFFO2 | blue | 0.056547674 | 0.71872024 |
| IFI16 | lightyellow | 0.382574589 | 0.011343852 |
| IFI27 | lightyellow | 0.265450599 | 0.08535991 |
| IFI35 | lightyellow | 0.079095113 | 0.614142633 |
| IFI44 | lightyellow | 0.474400197 | 0.001309185 |
| IFI44L | lightyellow | 0.382551797 | 0.011349141 |
| IFI6 | lightyellow | 0.174192884 | 0.263922776 |
| IFIH1 | lightyellow | 0.314193823 | 0.040181323 |
| IFIT1 | lightyellow | 0.301084715 | 0.04976735 |
| IFIT1B | blue | 0.146077254 | 0.34994637 |
| IFIT2 | lightyellow | 0.175492992 | 0.260319195 |
| IFIT3 | lightyellow | 0.277209823 | 0.071911988 |
| IFIT5 | lightyellow | 0.175888214 | 0.259230265 |
| IFITM1 | lightyellow | 0.336103389 | 0.02755398 |
| IFNA1 | blue | 0.073481226 | 0.639581584 |
| IFNA14 | blue | -0.324503562 | 0.033750857 |
| IFNA4 | blue | 0.066754851 | 0.670605696 |
| IFNA5 | blue | -0.257398553 | 0.09564408 |
| IFNB1 | blue | 0.200182293 | 0.198067839 |
| IFNE | blue | 0.322070398 | 0.035186389 |
| IFNG | blue | 0.079214425 | 0.613606666 |
| IFNK | blue | 0.198862625 | 0.201102577 |
| IFNL3 | blue | -0.418225891 | 0.005256767 |
| IFNW1 | blue | 0.122755403 | 0.432914636 |
| IFRD1 | blue | 0.171378431 | 0.271836747 |
| IFT27 | blue | -0.21254564 | 0.171193503 |
| IFT43 | blue | -0.240591087 | 0.120160831 |
| IFT57 | blue | -0.457568559 | 0.002035283 |
| IFT74 | blue | -0.247156138 | 0.11007543 |
| IFT80 | blue | -0.104621457 | 0.504344974 |
| IGF2BP2 | blue | 0.122636179 | 0.433365024 |
| IGF2BP3 | blue | 0.05239078 | 0.738639767 |
| IGFALS | blue | -0.273839629 | 0.075582694 |
| IGFBP1 | blue | 0.173033261 | 0.267164785 |
| IGFBP4 | blue | -0.235640591 | 0.128215259 |
| IGFBP5 | blue | 0.212925944 | 0.170410785 |
| IGFL4 | blue | -0.092649252 | 0.554579003 |
| IGIP | blue | 0.254981674 | 0.098910212 |
| IGLON5 | blue | 0.019539379 | 0.901026694 |
| IGSF1 | blue | 0.077903768 | 0.619505213 |
| IGSF10 | blue | 0.063215431 | 0.687154591 |
| IGSF22 | blue | -0.144121482 | 0.356504781 |
| IGSF23 | blue | -0.155827049 | 0.318362587 |
| IKBKB | lightcyan | 0.546315076 | 0.000150762 |
| IKZF1 | blue | 0.286672825 | 0.062353015 |
| IKZF2 | blue | -0.164953438 | 0.290483933 |
| IL10RB | blue | 0.065496728 | 0.676471054 |
| IL11 | blue | -0.118612729 | 0.448716489 |
| IL11RA | blue | 0.001480284 | 0.992483376 |
| IL12RB1 | blue | -0.072647069 | 0.64339752 |
| IL13RA2 | blue | 0.16207324 | 0.299105755 |
| IL15 | blue | 0.214704928 | 0.166783657 |
| IL15RA | lightyellow | 0.232419812 | 0.133668238 |
| IL16 | blue | 0.012746715 | 0.935341759 |
| IL17B | blue | 0.177951189 | 0.253595642 |
| IL17C | blue | -0.049985527 | 0.750244149 |
| IL17RC | blue | 0.044882124 | 0.775044122 |
| IL18BP | blue | 0.321665002 | 0.035430373 |
| IL19 | blue | -0.131764616 | 0.399643068 |
| IL1RAPL1 | blue | 0.013705754 | 0.930488445 |
| IL1RAPL2 | lightcyan | -0.272722032 | 0.076831923 |
| IL20RB | blue | 0.286357921 | 0.062653937 |
| IL21 | blue | 0.030923003 | 0.843946916 |
| IL21R | blue | 0.224373433 | 0.148043735 |
| IL23R | blue | 0.348534415 | 0.021992893 |
| IL24 | blue | 0.146421469 | 0.348799772 |
| IL26 | blue | 0.337003699 | 0.027115422 |
| IL31RA | blue | -0.034587894 | 0.825722865 |
| IL34 | blue | 0.14782886 | 0.34413562 |
| IL36A | blue | -0.032199258 | 0.837590966 |
| IL36RN | blue | 0.044249648 | 0.778133646 |
| IL3RA | lightcyan | 0.120969071 | 0.439690082 |
| ILK | blue | 0.215880832 | 0.164417012 |
| ILKAP | blue | 0.082235049 | 0.60010433 |
| IMMP2L | blue | -0.300902001 | 0.049912964 |
| IMMT | blue | -0.000676516 | 0.996564732 |
| IMPA1 | blue | 0.24205716 | 0.117850361 |
| IMPACT | blue | -0.280086061 | 0.06889177 |
| IMPDH2 | blue | -0.205209379 | 0.186803175 |
| IMPG2 | blue | -0.211489833 | 0.173380082 |
| ING2 | blue | -0.128081787 | 0.413061113 |
| INHA | blue | 0.261130597 | 0.090764748 |
| INIP | blue | -0.089320931 | 0.568950609 |
| INO80B | blue | -0.212748698 | 0.17077526 |
| INO80D | blue | 0.106903455 | 0.49503958 |
| INO80E | blue | 0.047817458 | 0.760751136 |
| INPP1 | lightcyan | 0.405359483 | 0.007004355 |
| INPP4A | blue | 0.379227375 | 0.012143517 |
| INPP5D | blue | 0.607023495 | 1.59E-05 |
| INPP5E | blue | 0.427507281 | 0.004244239 |
| INPP5F | blue | 0.360891457 | 0.017428993 |
| INPP5K | blue | 0.312324118 | 0.041448278 |
| INS | blue | 0.040225596 | 0.797867644 |
| INSM2 | blue | 0.076935058 | 0.623880081 |
| INSRR | blue | -0.506752225 | 0.000525069 |
| INTS10 | blue | -0.239428208 | 0.122017636 |
| INTS12 | blue | 0.094493206 | 0.546691174 |
| INTS2 | blue | -0.131129531 | 0.401938749 |
| INTS3 | blue | 0.328746329 | 0.031363139 |
| INTS9 | blue | -0.393263077 | 0.009084404 |
| INVS | blue | 0.095278084 | 0.54335004 |
| IP6K2 | blue | 0.095638756 | 0.541817988 |
| IPMK | blue | 0.410084087 | 0.006311726 |
| IPO8 | blue | -0.054812592 | 0.727013135 |
| IPO9 | blue | 0.268398707 | 0.08181767 |
| IQCA1 | blue | 0.039314757 | 0.802352205 |
| IQCC | blue | -0.003287862 | 0.98330574 |
| IQCE | blue | -0.199361486 | 0.199951569 |
| IQCF1 | blue | 0.006698677 | 0.96599471 |
| IQCF2 | blue | -0.113753017 | 0.467648327 |
| IQCF3 | blue | -0.215231487 | 0.165720866 |
| IQCF5 | blue | 0.027802368 | 0.859528651 |
| IQCJ | blue | -0.066347212 | 0.672504012 |
| IQCK | blue | -0.227945303 | 0.141528067 |
| IQGAP3 | blue | 0.038719736 | 0.805285216 |
| IRAK2 | lightcyan | 0.3955146 | 0.008661296 |
| IRAK3 | blue | 0.10199889 | 0.515147744 |
| IRF1 | blue | 0.423068597 | 0.004704964 |
| IRF2BP1 | blue | -0.263074667 | 0.088300591 |
| IRF3 | blue | 0.231273697 | 0.135649775 |
| IRF5 | blue | -0.038972204 | 0.804040413 |
| IRF7 | lightyellow | 0.266719835 | 0.08382053 |
| IRF9 | lightyellow | 0.225589256 | 0.145801572 |
| IRGC | lightcyan | -0.088777571 | 0.571312988 |
| IRS4 | blue | -0.492624693 | 0.000791191 |
| ISG15 | lightyellow | 0.267097898 | 0.083366207 |
| ISL2 | blue | -0.01172852 | 0.940496963 |
| ISM2 | blue | -0.207533829 | 0.181751636 |
| ISOC1 | blue | -0.258452589 | 0.094245981 |
| ISOC2 | blue | -0.253141677 | 0.101453706 |
| IST1 | blue | -0.059773379 | 0.703387952 |
| ITGA2 | blue | -0.036178592 | 0.817840426 |
| ITGA5 | lightcyan | 0.519980453 | 0.000351854 |
| ITGA9 | blue | 0.297622012 | 0.052585974 |
| ITGAE | blue | 0.133107728 | 0.39481313 |
| ITGB1 | blue | -0.007224701 | 0.963326134 |
| ITGB1BP2 | blue | -0.010455127 | 0.946947611 |
| ITGB3 | blue | 0.112045705 | 0.474399157 |
| ITGBL1 | blue | 0.077246174 | 0.622473622 |
| ITIH3 | blue | 0.259220137 | 0.093237851 |
| ITM2A | blue | 0.238754534 | 0.123103145 |
| ITPK1 | blue | 0.109379006 | 0.485045751 |
| ITPKA | blue | -0.217445991 | 0.161304851 |
| ITSN1 | blue | -0.147810239 | 0.344197078 |
| IVL | blue | 0.145780392 | 0.350937082 |
| IWS1 | blue | 0.365892455 | 0.015824102 |
| IZUMO1 | blue | 0.18578327 | 0.232954842 |
| IZUMO2 | blue | 0.06726858 | 0.66821622 |
| IZUMO3 | blue | 0.096938456 | 0.536314446 |
| JAKMIP2 | blue | -0.24834654 | 0.108317814 |
| JAM2 | blue | 0.401112694 | 0.007682056 |
| JDP2 | blue | 0.039006209 | 0.803872785 |
| JMJD4 | blue | 0.093211577 | 0.552167889 |
| JPH2 | blue | -0.128522435 | 0.411442255 |
| JPH3 | lightcyan | -0.472654055 | 0.001371934 |
| JPH4 | blue | 0.179581326 | 0.249201733 |
| JSRP1 | blue | 0.083070825 | 0.596391364 |
| KAAG1 | blue | -0.303575882 | 0.047816002 |
| KALRN | blue | 0.225114592 | 0.146673926 |
| KANK3 | blue | 0.262636224 | 0.088851742 |
| KANSL1L | blue | -0.195371784 | 0.209287238 |
| KAT6A | blue | 0.170005389 | 0.275753798 |
| KAZALD1 | blue | 0.133092433 | 0.394867939 |
| KAZN | blue | -0.329435752 | 0.030988659 |
| KBTBD12 | blue | 0.078783721 | 0.615542396 |
| KBTBD13 | blue | 0.187416641 | 0.228799222 |
| KBTBD2 | blue | 0.18245389 | 0.241584301 |
| KBTBD7 | blue | -0.189447791 | 0.223702841 |
| KBTBD8 | blue | -0.116635212 | 0.45636922 |
| KCNA1 | blue | 0.12985593 | 0.406565397 |
| KCNAB2 | blue | -0.098992207 | 0.527673504 |
| KCNAB3 | blue | 0.123537971 | 0.429964835 |
| KCNB1 | blue | -0.226844742 | 0.143512632 |
| KCNC3 | blue | 0.098518873 | 0.529658928 |
| KCND2 | blue | -0.05345034 | 0.733545775 |
| KCNE1 | blue | 0.112693795 | 0.471830515 |
| KCNE3 | blue | 0.078101024 | 0.618615953 |
| KCNE4 | blue | 0.263672448 | 0.087553422 |
| KCNG2 | blue | -0.179978182 | 0.248139842 |
| KCNG3 | blue | -0.014703796 | 0.9254404 |
| KCNG4 | blue | -0.047803851 | 0.760817216 |
| KCNH4 | blue | 0.006392266 | 0.967549334 |
| KCNH5 | blue | 0.010612129 | 0.946152097 |
| KCNIP2 | blue | 0.105192887 | 0.502006528 |
| KCNIP3 | blue | -0.232705126 | 0.133178323 |
| KCNJ11 | blue | -0.177898896 | 0.253737447 |
| KCNJ18 | blue | 0.136583233 | 0.382473776 |
| KCNJ2 | blue | 0.121352852 | 0.438229504 |
| KCNJ4 | blue | -0.009518701 | 0.951693443 |
| KCNJ5 | blue | 0.281670866 | 0.06727103 |
| KCNJ6 | blue | -0.032169557 | 0.837738768 |
| KCNK1 | blue | 0.153124159 | 0.326932466 |
| KCNK10 | blue | -0.181941452 | 0.242931478 |
| KCNK18 | blue | 0.060320162 | 0.700800284 |
| KCNK3 | blue | 0.322063832 | 0.03519033 |
| KCNK9 | blue | -0.130969674 | 0.40251779 |
| KCNMB1 | blue | 0.079622902 | 0.611773223 |
| KCNMB2 | blue | -0.012311031 | 0.937547354 |
| KCNMB3 | blue | 0.308339206 | 0.044257325 |
| KCNMB4 | blue | -0.174420671 | 0.263289025 |
| KCNN4 | blue | 0.221358108 | 0.153713744 |
| KCNQ5 | blue | 0.216274224 | 0.163630727 |
| KCNS2 | blue | -0.176347069 | 0.257969829 |
| KCNT2 | blue | 0.04759656 | 0.761824083 |
| KCNV2 | blue | -0.184595121 | 0.236009919 |
| KCP | blue | -5.53E-05 | 0.999719418 |
| KCTD10 | blue | 0.123118876 | 0.431543165 |
| KCTD12 | blue | 0.268211536 | 0.082039091 |
| KCTD15 | blue | 0.4180271 | 0.005280572 |
| KCTD16 | blue | -0.36603009 | 0.015781765 |
| KCTD18 | blue | 0.187451385 | 0.228711382 |
| KCTD20 | blue | 0.313949262 | 0.040345224 |
| KCTD3 | blue | -0.065011002 | 0.678740595 |
| KCTD5 | blue | 0.200512217 | 0.197314203 |
| KCTD6 | blue | 0.104002724 | 0.50688322 |
| KDM1B | blue | 0.088533024 | 0.572377672 |
| KDM2A | blue | 0.233648405 | 0.131568135 |
| KDM2B | blue | -0.109298275 | 0.485369995 |
| KDM4A | blue | -0.033497361 | 0.831136665 |
| KDM4E | blue | -0.075963206 | 0.628282011 |
| KEAP1 | blue | -0.154169094 | 0.323602406 |
| KEL | blue | 0.064191434 | 0.682576359 |
| KERA | blue | -0.182184128 | 0.24229286 |
| KHDC1L | blue | -0.018939691 | 0.904049944 |
| KIAA0100 | blue | 0.188056577 | 0.22718504 |
| KIAA0319 | blue | -0.15243035 | 0.329155328 |
| KIAA0586 | blue | -0.014609184 | 0.925918824 |
| KIAA0895 | blue | -0.033342175 | 0.831907699 |
| KIAA0895L | blue | 0.185970914 | 0.232474835 |
| KIAA0930 | blue | 0.172448136 | 0.268810611 |
| KIAA1522 | blue | 0.068935305 | 0.660486246 |
| KIAA1549 | blue | -0.268517435 | 0.081677457 |
| KIAA1586 | blue | 0.054508537 | 0.728469584 |
| KIAA1671 | blue | 0.287959942 | 0.061134992 |
| KIAA1841 | lightcyan | 0.301833991 | 0.049173793 |
| KIAA2026 | blue | 0.152512154 | 0.328892749 |
| KIF11 | blue | 0.198513894 | 0.201909953 |
| KIF18A | lightcyan | -0.185660626 | 0.233268939 |
| KIF18B | blue | 0.020017799 | 0.898615792 |
| KIF1A | blue | 0.091902339 | 0.557789227 |
| KIF20B | blue | -0.093963919 | 0.548949806 |
| KIF24 | blue | -0.188541347 | 0.225967465 |
| KIF25 | blue | 0.07974938 | 0.611205999 |
| KIF26B | blue | -0.222956046 | 0.150689511 |
| KIF3A | blue | -0.117306477 | 0.453763626 |
| KIF3C | blue | 0.110448873 | 0.480759488 |
| KIF4A | blue | 0.049799404 | 0.751144427 |
| KIF5A | blue | -0.066172795 | 0.673316858 |
| KIF7 | blue | 0.112846006 | 0.471228314 |
| KIFC1 | blue | -0.069714227 | 0.656885605 |
| KIN | blue | 0.368501939 | 0.015037588 |
| KISS1 | blue | -0.24713582 | 0.110105616 |
| KLC2 | blue | -0.128830116 | 0.410314048 |
| KLC4 | blue | -0.099084818 | 0.52728547 |
| KLF1 | blue | 0.017554234 | 0.9110396 |
| KLF13 | blue | -0.297141889 | 0.052986735 |
| KLF15 | blue | -0.402650341 | 0.007430419 |
| KLHDC3 | blue | -0.152284229 | 0.329624677 |
| KLHDC4 | blue | 0.065750569 | 0.675286116 |
| KLHDC7B | blue | -0.318437564 | 0.037422763 |
| KLHDC8B | blue | -0.101187576 | 0.518512944 |
| KLHDC9 | blue | -0.333900824 | 0.028651773 |
| KLHL14 | blue | 0.218497916 | 0.159237415 |
| KLHL20 | lightcyan | 0.202889594 | 0.191943342 |
| KLHL22 | lightcyan | -0.212355752 | 0.171585289 |
| KLHL23 | blue | -0.225701258 | 0.145596289 |
| KLHL25 | lightcyan | -0.312667353 | 0.041213285 |
| KLHL28 | blue | 0.136469145 | 0.382875183 |
| KLHL31 | blue | -0.075179328 | 0.631841836 |
| KLHL32 | blue | -0.413785924 | 0.005811279 |
| KLHL34 | blue | 0.156542316 | 0.316118667 |
| KLHL38 | blue | 0.018971554 | 0.903889276 |
| KLHL40 | blue | -0.127885036 | 0.413785108 |
| KLHL41 | blue | 0.362913576 | 0.016764255 |
| KLHL5 | blue | 0.604833838 | 1.74E-05 |
| KLHL6 | blue | 0.243227011 | 0.116030924 |
| KLHL8 | blue | -0.250087068 | 0.105786538 |
| KLK14 | blue | 0.016260439 | 0.917572817 |
| KLK2 | blue | -0.014854831 | 0.924676721 |
| KLK9 | blue | 0.025535665 | 0.87088006 |
| KLLN | blue | -0.167821457 | 0.282060198 |
| KLRF2 | blue | -0.036309075 | 0.817194614 |
| KLRG1 | blue | 0.095180167 | 0.543766327 |
| KMT2B | blue | 0.02308126 | 0.883200341 |
| KNCN | blue | 0.053832309 | 0.731712153 |
| KNDC1 | blue | 0.124653917 | 0.425777908 |
| KNTC1 | blue | 0.396739889 | 0.008438245 |
| KPNA1 | blue | 0.448094481 | 0.002584141 |
| KRAS | blue | 0.190735263 | 0.220513211 |
| KRT12 | blue | 0.051439703 | 0.743221644 |
| KRT16 | blue | -0.269238936 | 0.080829429 |
| KRT25 | blue | -0.126271445 | 0.419749961 |
| KRT27 | blue | -0.030252408 | 0.847290537 |
| KRT33A | blue | 0.085630821 | 0.585081719 |
| KRT33B | lightyellow | 0.238308854 | 0.123825261 |
| KRT36 | blue | 0.050934009 | 0.74566144 |
| KRT6A | blue | -0.071723646 | 0.647632394 |
| KRT6B | blue | -0.338857331 | 0.02623073 |
| KRT6C | blue | -0.141056985 | 0.366929958 |
| KRT71 | blue | 0.00339001 | 0.982787155 |
| KRT72 | blue | 0.01611166 | 0.918324453 |
| KRT73 | lightcyan | -0.333982232 | 0.028610563 |
| KRT83 | blue | -0.17075938 | 0.273598229 |
| KRT85 | blue | 0.049940948 | 0.750459747 |
| KRTAP10-12 | blue | 0.239537841 | 0.121841666 |
| KRTAP10-4 | blue | -0.034184495 | 0.827724567 |
| KRTAP10-6 | blue | -0.263514337 | 0.087750566 |
| KRTAP10-8 | blue | 0.206206393 | 0.184624353 |
| KRTAP10-9 | blue | 0.044058141 | 0.77906979 |
| KRTAP12-4 | blue | 0.021149554 | 0.892916164 |
| KRTAP1-3 | blue | -0.089296341 | 0.569057424 |
| KRTAP13-1 | blue | 0.090134114 | 0.565423543 |
| KRTAP13-2 | blue | 0.011046658 | 0.943950641 |
| KRTAP1-4 | blue | 0.084273271 | 0.591067211 |
| KRTAP15-1 | blue | -0.152065298 | 0.330328679 |
| KRTAP19-3 | blue | 0.182262137 | 0.242087816 |
| KRTAP19-5 | blue | -0.124395298 | 0.426746176 |
| KRTAP19-6 | blue | -0.23342524 | 0.131947762 |
| KRTAP19-8 | blue | -0.130067441 | 0.405794921 |
| KRTAP2-1 | blue | 0.101976043 | 0.515242359 |
| KRTAP2-3 | blue | -0.158323339 | 0.310574836 |
| KRTAP23-1 | lightcyan | -0.197369362 | 0.204575735 |
| KRTAP26-1 | blue | -0.011358971 | 0.942368625 |
| KRTAP3-1 | blue | -0.191510666 | 0.218607423 |
| KRTAP3-3 | blue | 0.024868977 | 0.874223749 |
| KRTAP4-1 | blue | 0.180045963 | 0.247958783 |
| KRTAP4-11 | blue | 0.099414396 | 0.525905695 |
| KRTAP5-1 | blue | 0.154649558 | 0.322078408 |
| KRTAP5-10 | blue | -0.213867324 | 0.168484405 |
| KRTAP5-2 | blue | 0.098487865 | 0.529789122 |
| KRTAP5-7 | lightcyan | -0.108743876 | 0.4875997 |
| KRTAP6-1 | blue | -0.146710694 | 0.347838124 |
| KRTAP6-2 | blue | -0.313335097 | 0.040759232 |
| KRTAP9-3 | blue | -0.375252819 | 0.013155051 |
| KRTAP9-6 | blue | -0.031225477 | 0.842439651 |
| KRTAP9-7 | blue | -0.174091093 | 0.264206307 |
| KRTAP9-8 | blue | 0.100098644 | 0.523046793 |
| KSR1 | blue | 0.543775652 | 0.000164105 |
| KTI12 | blue | -0.008828737 | 0.955191266 |
| L1TD1 | blue | 0.239028169 | 0.122661357 |
| L3HYPDH | blue | 0.145012226 | 0.353508601 |
| L3MBTL1 | blue | 0.304846284 | 0.046844991 |
| L3MBTL2 | blue | 0.331812867 | 0.029725682 |
| L3MBTL4 | blue | 0.192165785 | 0.217006175 |
| LAG3 | blue | 0.087475262 | 0.576993268 |
| LAGE3 | blue | 0.203133468 | 0.191398305 |
| LALBA | blue | 0.156024865 | 0.317741002 |
| LAMP3 | blue | -0.124277945 | 0.427185954 |
| LAMTOR1 | blue | -0.276003294 | 0.07320959 |
| LAMTOR3 | blue | -0.449480245 | 0.002496501 |
| LAP3 | lightyellow | 0.355596883 | 0.01927569 |
| LAPTM4A | blue | -0.217871145 | 0.160466923 |
| LAPTM4B | blue | 0.055384655 | 0.724275478 |
| LARP1 | blue | -0.2098294 | 0.176859378 |
| LARP1B | blue | -0.26437745 | 0.086678558 |
| LARP6 | blue | -0.04042907 | 0.796866703 |
| LARP7 | blue | 0.155471914 | 0.319480432 |
| LARS2 | blue | -0.282905856 | 0.066029103 |
| LASP1 | blue | -0.338136094 | 0.026572061 |
| LAT2 | blue | 0.244629314 | 0.113878064 |
| LATS1 | blue | 0.122507741 | 0.433850512 |
| LBH | blue | 0.28258886 | 0.06634613 |
| LCE1A | blue | -0.026428795 | 0.866404174 |
| LCE1C | blue | 0.173817346 | 0.264969811 |
| LCE1F | lightcyan | -0.171820797 | 0.270582607 |
| LCE2A | blue | 0.040219481 | 0.797897732 |
| LCE2B | blue | -0.266929419 | 0.083568433 |
| LCE2C | blue | -0.249806016 | 0.106192189 |
| LCE3A | blue | 0.004197237 | 0.978689294 |
| LCE3B | blue | 0.258967352 | 0.093568946 |
| LCE3C | blue | 0.00664583 | 0.966262826 |
| LCE3D | blue | -0.142809402 | 0.360946172 |
| LCE5A | blue | -0.285609975 | 0.063373309 |
| LCE6A | blue | -0.070556249 | 0.653001895 |
| LCLAT1 | blue | -0.087265985 | 0.57790846 |
| LCMT1 | blue | -0.130543273 | 0.404064677 |
| LCMT2 | blue | -0.398433016 | 0.008138177 |
| LCN10 | blue | -0.027969979 | 0.858690357 |
| LCN15 | lightyellow | 0.252135743 | 0.102865285 |
| LCN6 | blue | -0.027345449 | 0.861814681 |
| LCN8 | blue | 0.050875915 | 0.745941884 |
| LCN9 | blue | 0.143048199 | 0.360135365 |
| LCP2 | lightyellow | 0.467858958 | 0.001558196 |
| LDB2 | blue | 0.311286088 | 0.042165612 |
| LDB3 | blue | 0.100288837 | 0.522253498 |
| LDHAL6B | blue | 0.29804978 | 0.052230971 |
| LDLR | blue | 0.245535431 | 0.112503156 |
| LDLRAD1 | blue | -0.063249016 | 0.686996864 |
| LDLRAD2 | blue | 0.197489459 | 0.20429486 |
| LDLRAD3 | blue | 0.254048348 | 0.100194187 |
| LECT2 | lightcyan | -0.338350876 | 0.02647003 |
| LEFTY2 | blue | 0.109359805 | 0.485122861 |
| LELP1 | blue | 0.035643007 | 0.820492487 |
| LENEP | blue | 0.212698688 | 0.170878198 |
| LETM1 | blue | -0.068087363 | 0.664414551 |
| LFNG | blue | 0.361371768 | 0.017269118 |
| LGALS4 | blue | -0.154064782 | 0.323933876 |
| LGALS9 | lightyellow | 0.500835466 | 0.000624778 |
| LGALS9B | lightyellow | 0.44175696 | 0.003020381 |
| LGALS9C | lightyellow | 0.50299594 | 0.000586559 |
| LGI3 | blue | 0.048555376 | 0.757170128 |
| LGR5 | lightcyan | -0.434240784 | 0.003620492 |
| LGR6 | blue | 0.143696573 | 0.357939453 |
| LGSN | blue | -0.274674969 | 0.074659432 |
| LHFPL5 | blue | -0.126317886 | 0.419577611 |
| LHX3 | blue | -0.22525572 | 0.146414157 |
| LHX8 | blue | -0.117170745 | 0.454289833 |
| LIFR | blue | 0.194501509 | 0.211363358 |
| LILRA4 | blue | 0.264444231 | 0.08659604 |
| LILRB5 | blue | 0.11767074 | 0.452353086 |
| LIMA1 | lightcyan | 0.523497198 | 0.000315455 |
| LIMCH1 | blue | 0.314092797 | 0.040248963 |
| LIMD1 | blue | 0.148323125 | 0.342506741 |
| LIN52 | blue | -0.308330569 | 0.044263578 |
| LIN9 | blue | -0.109374658 | 0.485063214 |
| LINC00514 | blue | 0.298109873 | 0.052181254 |
| LINC00634 | blue | -0.0247936 | 0.874601929 |
| LINGO2 | blue | -0.161531343 | 0.3007461 |
| LINGO4 | blue | -0.15706465 | 0.314486344 |
| LIPA | blue | 0.236970923 | 0.126012174 |
| LIPC | blue | -0.098968323 | 0.527773597 |
| LIPI | blue | -0.03591651 | 0.819137929 |
| LIPJ | blue | 0.078886262 | 0.615081308 |
| LIPM | blue | 0.014529761 | 0.92632046 |
| LIPT2 | blue | -0.343208897 | 0.024247841 |
| LLGL1 | blue | -0.052499087 | 0.738118555 |
| LLPH | lightcyan | 0.173630462 | 0.265491886 |
| LMAN2L | blue | -0.110176176 | 0.481850119 |
| LMBR1 | blue | -0.147127297 | 0.346455816 |
| LMF2 | blue | 0.232190369 | 0.13406319 |
| LMNA | blue | 0.31743739 | 0.038058507 |
| LMO3 | blue | 0.138787618 | 0.374766783 |
| LMO7 | blue | -0.195373497 | 0.209283167 |
| LMOD2 | lightcyan | 0.036168037 | 0.817892671 |
| LMOD3 | blue | 0.18455142 | 0.236122807 |
| LMTK2 | blue | -0.299198626 | 0.05128709 |
| LMTK3 | blue | -0.269110051 | 0.080980409 |
| LMX1A | blue | -0.342171634 | 0.024708756 |
| LNX1 | blue | -0.214689756 | 0.166814354 |
| LONP1 | blue | -0.278214265 | 0.070845623 |
| LONRF2 | blue | 0.010063017 | 0.948934626 |
| LPA | blue | 0.142710614 | 0.361281915 |
| LPAR5 | blue | 0.138203081 | 0.376801384 |
| LPIN3 | blue | -0.144435182 | 0.35544784 |
| LPO | blue | -0.005741645 | 0.970850749 |
| LPP | blue | 0.301306751 | 0.049590859 |
| LRCOL1 | blue | -0.094113711 | 0.54831015 |
| LRFN1 | blue | -0.009067632 | 0.953980071 |
| LRFN2 | blue | -0.098783004 | 0.528550566 |
| LRIG3 | blue | -0.213488267 | 0.169258182 |
| LRIT1 | blue | 0.217751205 | 0.160702989 |
| LRIT3 | blue | 0.089357007 | 0.568793923 |
| LRP1B | blue | -0.212669068 | 0.170939188 |
| LRP3 | blue | -0.028368125 | 0.856699664 |
| LRP8 | blue | 0.096187472 | 0.539491162 |
| LRPAP1 | blue | -0.226064688 | 0.144931643 |
| LRRC1 | blue | -0.540581831 | 0.0001824 |
| LRRC10B | blue | 0.034432288 | 0.826494871 |
| LRRC14 | blue | -0.087808072 | 0.575539215 |
| LRRC18 | blue | 0.271876582 | 0.077787671 |
| LRRC23 | blue | -0.417302949 | 0.005368085 |
| LRRC24 | blue | -0.234763336 | 0.129683721 |
| LRRC27 | blue | -0.352183106 | 0.020551576 |
| LRRC32 | blue | 0.396219833 | 0.008532304 |
| LRRC37A | blue | 0.147413627 | 0.345507713 |
| LRRC37A2 | blue | 0.148764556 | 0.341056005 |
| LRRC38 | blue | -0.072937467 | 0.642068029 |
| LRRC40 | blue | 0.039181116 | 0.803010725 |
| LRRC46 | blue | 0.014962978 | 0.924129935 |
| LRRC47 | blue | -0.25955567 | 0.092799774 |
| LRRC49 | blue | -0.003539282 | 0.982029343 |
| LRRC4B | lightyellow | 0.024138018 | 0.877892266 |
| LRRC52 | blue | -0.068675853 | 0.66168727 |
| LRRC58 | blue | -0.316291418 | 0.038797766 |
| LRRC59 | blue | 0.088330446 | 0.573260323 |
| LRRC6 | blue | -0.443218032 | 0.00291445 |
| LRRC61 | blue | -0.079568615 | 0.612016755 |
| LRRC66 | blue | 0.054407613 | 0.72895323 |
| LRRC73 | blue | -0.098521505 | 0.52964788 |
| LRRC8B | blue | 0.445865456 | 0.002730797 |
| LRRC8C | blue | 0.198535951 | 0.20185882 |
| LRRC8E | blue | -0.216195442 | 0.163787972 |
| LRRD1 | blue | -0.050690587 | 0.746836746 |
| LRRFIP2 | blue | 0.178935287 | 0.25093691 |
| LRRIQ3 | blue | -0.210562445 | 0.17531722 |
| LRRK2 | blue | 0.205665433 | 0.185804281 |
| LRRN2 | blue | 0.05111519 | 0.744787025 |
| LRRN3 | blue | 0.12313214 | 0.431493164 |
| LRRTM2 | blue | 0.116133021 | 0.45832382 |
| LRRTM3 | blue | -0.037849377 | 0.809580189 |
| LRRTM4 | blue | -0.079357304 | 0.612965088 |
| LRSAM1 | blue | 0.107018589 | 0.494572447 |
| LRTOMT | blue | -0.093277816 | 0.551884202 |
| LSM12 | blue | -0.293976961 | 0.055690388 |
| LSM3 | blue | -0.499673967 | 0.000646233 |
| LSM4 | blue | -0.328667341 | 0.031406281 |
| LSS | blue | -0.040651562 | 0.795772575 |
| LTA | blue | 0.065727335 | 0.675394541 |
| LTB | blue | 0.273738261 | 0.075695339 |
| LTN1 | blue | 0.420388626 | 0.005003744 |
| LURAP1L | blue | -0.054604496 | 0.728009831 |
| LY6E | lightyellow | 0.173804343 | 0.265006111 |
| LY6G6D | blue | -0.012040664 | 0.938916285 |
| LY6G6E | blue | -0.313688266 | 0.04052074 |
| LY75-CD302 | blue | 0.018585027 | 0.905838571 |
| LYAR | blue | 0.082753234 | 0.597801088 |
| LYNX1 | blue | -0.055246082 | 0.724938324 |
| LYPLA2 | blue | -0.206657327 | 0.183644876 |
| LYSMD2 | blue | 0.09402594 | 0.548684914 |
| LYZL1 | lightcyan | -0.263347269 | 0.087959253 |
| LYZL2 | blue | 0.088947567 | 0.570573407 |
| LYZL4 | blue | -0.058118894 | 0.711237925 |
| LZTS1 | blue | -0.038804533 | 0.80486707 |
| LZTS2 | blue | -0.022340282 | 0.886925288 |
| LZTS3 | blue | -0.015009833 | 0.923893052 |
| MAB21L2 | blue | 0.287060492 | 0.061984141 |
| MAD2L1 | blue | -0.230529025 | 0.136948905 |
| MADD | blue | 0.253526783 | 0.100917254 |
| MAGEB1 | blue | 0.085914479 | 0.583834498 |
| MAGEB10 | blue | -0.297859801 | 0.052388394 |
| MAGEB16 | blue | -0.045277175 | 0.773116109 |
| MAGEC2 | blue | 0.010996912 | 0.944202648 |
| MAGEF1 | blue | -0.308975336 | 0.043798806 |
| MAGOH | blue | -0.104994076 | 0.502819487 |
| MAML1 | blue | -0.190614811 | 0.220810284 |
| MAN1A1 | blue | -0.269095167 | 0.080997858 |
| MANSC4 | blue | 0.269454471 | 0.080577436 |
| MAP10 | blue | -0.352358495 | 0.02048434 |
| MAP1S | blue | 0.077324165 | 0.622121258 |
| MAP2 | blue | 0.235540265 | 0.128382564 |
| MAP2K1 | blue | -0.264282796 | 0.086795621 |
| MAP2K2 | blue | -0.08589415 | 0.583923843 |
| MAP2K4 | blue | -0.100965477 | 0.51943608 |
| MAP3K12 | blue | 0.414022482 | 0.005780503 |
| MAP3K2 | blue | 0.108995766 | 0.486585981 |
| MAP3K4 | blue | 0.022554698 | 0.88584715 |
| MAP3K5 | blue | -0.138644819 | 0.375263221 |
| MAP3K6 | blue | 0.256233715 | 0.097207688 |
| MAP3K8 | blue | 0.188472802 | 0.226139355 |
| MAP4K1 | blue | 0.297830985 | 0.052412305 |
| MAP6D1 | blue | -0.043942859 | 0.779633468 |
| MAP7D1 | blue | 0.358720725 | 0.018167209 |
| MAP7D3 | blue | -0.081556039 | 0.603128249 |
| MAPK11 | blue | 0.26501982 | 0.085887348 |
| MAPK14 | blue | 0.139088629 | 0.373721609 |
| MAPK15 | blue | -0.193287615 | 0.214283089 |
| MAPK6 | blue | -0.185926192 | 0.232589177 |
| MAPK7 | blue | 0.102399822 | 0.513488783 |
| MAPK8IP1 | lightcyan | -0.406451821 | 0.006838635 |
| MAPK8IP2 | blue | -0.161901835 | 0.299623982 |
| MAPKAP1 | blue | -0.389565767 | 0.00981775 |
| MAPKAPK3 | blue | -0.092390102 | 0.555691839 |
| MAPKAPK5 | blue | 0.126056031 | 0.420549932 |
| MAPKBP1 | blue | 0.342702699 | 0.024471869 |
| MAPT | blue | -0.234253946 | 0.130542153 |
| MARCO | blue | 0.14957865 | 0.33839048 |
| MARS2 | blue | -0.139696491 | 0.371616281 |
| MARVELD2 | blue | -0.404566776 | 0.00712678 |
| MAS1L | blue | 0.027300435 | 0.862039953 |
| MASP2 | blue | 0.101273152 | 0.518157475 |
| MAT2B | blue | -0.132265767 | 0.397836903 |
| MATN1 | blue | 0.048141391 | 0.759178525 |
| MATN2 | blue | 0.350530937 | 0.02119408 |
| MATN4 | blue | -0.066775671 | 0.670508797 |
| MAU2 | blue | -0.054677005 | 0.727662489 |
| MAVS | blue | -0.080235481 | 0.609028058 |
| MAX | blue | 0.093425489 | 0.55125199 |
| MBD3L1 | blue | 0.147055161 | 0.346694927 |
| MBD4 | blue | -0.138497791 | 0.375774768 |
| MBIP | blue | -0.282417499 | 0.06651801 |
| MBNL1 | lightcyan | 0.612091876 | 1.29E-05 |
| MBNL2 | blue | -0.337870542 | 0.026698662 |
| MBTPS2 | blue | 0.074515027 | 0.634865077 |
| MC5R | blue | -0.285880491 | 0.063112373 |
| MCEE | blue | -0.267600091 | 0.08276569 |
| MCF2 | blue | 0.102764777 | 0.511981019 |
| MCF2L2 | blue | 0.38737118 | 0.010276609 |
| MCHR1 | lightyellow | 0.187089622 | 0.229627126 |
| MCIDAS | blue | -0.107544451 | 0.492441756 |
| MCM10 | blue | 0.077070498 | 0.623267636 |
| MCM5 | blue | -0.229868719 | 0.138108565 |
| MCM9 | blue | 0.208849421 | 0.178936194 |
| MCMBP | blue | 0.266591236 | 0.083975507 |
| MCOLN2 | blue | 0.10060686 | 0.520928361 |
| MDFI | blue | 0.156723105 | 0.315553088 |
| MDGA2 | blue | -0.127172981 | 0.416411336 |
| MDH2 | blue | -0.357068927 | 0.018746452 |
| MECP2 | blue | -0.016409316 | 0.916820757 |
| MECR | blue | -0.249317897 | 0.106899527 |
| MED10 | blue | 0.022454695 | 0.886349964 |
| MED20 | blue | 0.219716213 | 0.156867197 |
| MED6 | blue | 0.009261422 | 0.952997637 |
| MED8 | blue | -0.138030067 | 0.37740485 |
| MED9 | blue | -0.340987679 | 0.025243762 |
| MEF2A | lightcyan | 0.511273613 | 0.000458761 |
| MEF2C | lightcyan | 0.500717933 | 0.00062692 |
| MEF2D | blue | 0.238853898 | 0.122942583 |
| MEGF10 | blue | -0.087354507 | 0.577521261 |
| MEI1 | blue | 0.124356111 | 0.426893002 |
| MEIG1 | blue | -0.128798743 | 0.410429005 |
| MEIOB | blue | -0.01156245 | 0.941338022 |
| MEIS1 | blue | 0.254442048 | 0.099651023 |
| MEIS3 | lightyellow | 0.264936369 | 0.085989815 |
| MELK | blue | 0.16623004 | 0.286714479 |
| MEOX2 | blue | -0.110515551 | 0.480493014 |
| MEP1B | blue | 0.251436684 | 0.103855063 |
| MEPE | blue | -0.208732682 | 0.179184752 |
| MESP1 | blue | -0.20660722 | 0.183753531 |
| METRNL | blue | -0.227461402 | 0.142398136 |
| METTL14 | blue | -0.171747728 | 0.2707895 |
| METTL15 | blue | 0.171001754 | 0.272907671 |
| METTL16 | blue | 0.135106336 | 0.387689247 |
| METTL18 | blue | -0.029027497 | 0.853404806 |
| METTL23 | blue | -0.109248183 | 0.485571241 |
| METTL3 | blue | 0.25971706 | 0.092589628 |
| METTL8 | blue | -0.116091985 | 0.458483738 |
| MEX3A | blue | -0.328894174 | 0.03128252 |
| MFF | blue | -0.282377803 | 0.066557877 |
| MFGE8 | blue | 0.092630582 | 0.554659138 |
| MFN1 | lightcyan | 0.543709357 | 0.000164467 |
| MFSD11 | blue | 0.123472936 | 0.430209547 |
| MFSD2B | blue | 0.040613428 | 0.795960072 |
| MFSD8 | blue | 0.352182411 | 0.020551842 |
| MGARP | blue | -0.234702383 | 0.129786217 |
| MGAT1 | blue | 0.20757328 | 0.181666751 |
| MGAT2 | blue | -0.298638531 | 0.051745526 |
| MGAT4A | blue | -0.084214906 | 0.59132515 |
| MGAT5B | blue | -0.06694377 | 0.669726613 |
| MGP | blue | -0.211684848 | 0.1729747 |
| MIA | blue | 0.03877831 | 0.804996375 |
| MICA | blue | 0.150519918 | 0.335324664 |
| MICB | blue | 0.278283431 | 0.070772655 |
| MICU2 | blue | -0.408495668 | 0.006537654 |
| MID1 | blue | 0.335065345 | 0.028066926 |
| MID1IP1 | blue | -0.133471054 | 0.393512446 |
| MIER1 | blue | -0.049956173 | 0.750386114 |
| MIER2 | blue | 0.228352051 | 0.140799768 |
| MILR1 | blue | 0.346354826 | 0.022893585 |
| MINK1 | blue | -0.035458208 | 0.821408023 |
| MINPP1 | blue | 0.006340474 | 0.96781212 |
| MIOS | blue | 0.021122383 | 0.893052938 |
| MIPOL1 | blue | -0.105581267 | 0.50042033 |
| MIS18A | blue | -0.12501819 | 0.424416167 |
| MKKS | blue | -0.049592692 | 0.752144678 |
| MKNK1 | blue | 0.264091662 | 0.087032381 |
| MKRN1 | blue | 0.193335998 | 0.214166183 |
| MKRN3 | blue | -0.216600542 | 0.162980578 |
| MKX | blue | -0.057412344 | 0.714599327 |
| MLEC | blue | -0.256897407 | 0.096314394 |
| MLF2 | blue | -0.222765656 | 0.151047532 |
| MLKL | blue | 0.650789074 | 2.30E-06 |
| MLLT3 | blue | -0.074145278 | 0.636550354 |
| MLLT6 | blue | 0.163433741 | 0.295012843 |
| MLN | blue | -0.368577213 | 0.015015401 |
| MLST8 | blue | -0.157559737 | 0.312944105 |
| MLX | blue | -0.273481927 | 0.075980777 |
| MLXIP | blue | 0.025750906 | 0.869801022 |
| MMAB | blue | -0.363398909 | 0.016607938 |
| MMACHC | blue | -0.028954177 | 0.853771063 |
| MMD | blue | 0.017405601 | 0.911789859 |
| MMD2 | blue | -0.29883112 | 0.051587522 |
| MMEL1 | blue | -0.151759356 | 0.331314042 |
| MMP10 | blue | 0.211642777 | 0.173062095 |
| MMP15 | blue | -0.104375426 | 0.505353498 |
| MMP16 | blue | 0.031245393 | 0.842340423 |
| MMP26 | blue | 0.124813221 | 0.425182088 |
| MMRN1 | blue | -0.144037688 | 0.356787428 |
| MMRN2 | lightcyan | 0.501486041 | 0.000613041 |
| MMS19 | blue | 0.085560663 | 0.585390382 |
| MN1 | lightcyan | 0.489110646 | 0.000873771 |
| MNX1 | blue | -0.331990372 | 0.029633109 |
| MOB2 | blue | -0.071762511 | 0.647453938 |
| MOB3B | blue | -0.024339016 | 0.876883248 |
| MOB3C | blue | 0.240714418 | 0.119965161 |
| MOB4 | blue | 0.373200034 | 0.013704994 |
| MOBP | blue | -0.184591341 | 0.236019682 |
| MOCS1 | blue | -0.280114948 | 0.068861954 |
| MOCS2 | blue | -0.032120444 | 0.837983184 |
| MOGAT1 | blue | 0.049954863 | 0.750392448 |
| MOGAT2 | blue | -0.10318538 | 0.510246114 |
| MOGS | blue | 0.152261166 | 0.329698795 |
| MON1A | blue | -0.013316208 | 0.93245949 |
| MORC3 | blue | 0.342694752 | 0.0244754 |
| MORF4L2 | blue | 0.171601582 | 0.271203621 |
| MORN1 | blue | -0.119081776 | 0.446911691 |
| MORN2 | blue | -0.426547966 | 0.004340331 |
| MORN3 | blue | -0.079976556 | 0.610187741 |
| MORN4 | blue | -0.287943318 | 0.061150601 |
| MORN5 | blue | -0.240095114 | 0.120950144 |
| MOSPD3 | blue | -0.175038857 | 0.2615742 |
| MOV10 | blue | 0.132167476 | 0.398190772 |
| MPDU1 | blue | -0.059071889 | 0.706712625 |
| MPHOSPH10 | blue | -0.237597086 | 0.124985101 |
| MPHOSPH9 | blue | -0.136375324 | 0.383205467 |
| MPLKIP | blue | -0.352263151 | 0.020520867 |
| MPP3 | blue | -0.183860568 | 0.237912262 |
| MPP7 | blue | -0.435960213 | 0.0034747 |
| MPPED1 | blue | 0.007647381 | 0.961182121 |
| MPV17L | blue | -0.268801969 | 0.0813422 |
| MPV17L2 | blue | -0.082478064 | 0.599023689 |
| MPZL2 | lightcyan | 0.453603972 | 0.002250988 |
| MRAS | lightcyan | 0.443890309 | 0.002866815 |
| MRGPRX2 | blue | -0.085214425 | 0.586914743 |
| MRGPRX4 | blue | 0.215941464 | 0.164295645 |
| MROH2A | blue | -0.19765473 | 0.203908775 |
| MROH2B | blue | -0.051068388 | 0.74501287 |
| MROH5 | blue | 0.083164001 | 0.595978051 |
| MRPL1 | blue | 0.045863361 | 0.770257766 |
| MRPL10 | blue | -0.130514956 | 0.404167525 |
| MRPL12 | blue | -0.410569858 | 0.006243997 |
| MRPL13 | blue | -0.247552689 | 0.109487533 |
| MRPL17 | blue | 0.003322481 | 0.983129984 |
| MRPL19 | blue | -0.224586982 | 0.147648093 |
| MRPL2 | blue | -0.259391639 | 0.093013737 |
| MRPL20 | blue | -0.204332422 | 0.188734695 |
| MRPL21 | blue | -0.219117062 | 0.158029616 |
| MRPL23 | blue | -0.405060988 | 0.007050239 |
| MRPL24 | blue | -0.281305009 | 0.067642473 |
| MRPL27 | blue | -0.303584709 | 0.047809199 |
| MRPL28 | blue | -0.305128785 | 0.046631252 |
| MRPL30 | blue | -0.491492669 | 0.000816998 |
| MRPL34 | blue | -0.136203782 | 0.383809789 |
| MRPL35 | blue | -0.32963553 | 0.030880837 |
| MRPL36 | blue | -0.367415371 | 0.015360963 |
| MRPL37 | blue | -0.172903078 | 0.267530384 |
| MRPL39 | blue | -0.123747163 | 0.429178217 |
| MRPL41 | blue | -0.371875019 | 0.014070249 |
| MRPL44 | blue | -0.150037275 | 0.336894528 |
| MRPL46 | blue | -0.15153576 | 0.332035345 |
| MRPL49 | blue | -0.18500997 | 0.234940138 |
| MRPL52 | blue | -0.084016143 | 0.592203946 |
| MRPS10 | blue | -0.336373945 | 0.027421573 |
| MRPS12 | blue | -0.347568458 | 0.022388333 |
| MRPS17 | blue | -0.245237216 | 0.112954259 |
| MRPS18B | blue | -0.298033975 | 0.052244053 |
| MRPS23 | blue | -0.264290284 | 0.086786357 |
| MRPS25 | blue | 0.035756852 | 0.819928597 |
| MRPS26 | blue | -0.257064977 | 0.096089857 |
| MRPS28 | blue | -0.147204382 | 0.346200415 |
| MRPS30 | blue | -0.164874781 | 0.290717229 |
| MRPS33 | blue | -0.3414062 | 0.025053548 |
| MRPS35 | blue | -0.318111585 | 0.037629003 |
| MRPS5 | blue | -0.239669757 | 0.121630181 |
| MRPS7 | blue | -0.30417558 | 0.047355622 |
| MRRF | blue | -0.017395607 | 0.911840307 |
| MS4A2 | blue | 0.265588014 | 0.085192193 |
| MS4A4E | blue | 0.393146416 | 0.009106803 |
| MS4A5 | lightcyan | -0.31419919 | 0.040177732 |
| MSANTD4 | blue | 0.012258224 | 0.937814714 |
| MSGN1 | blue | -0.350159302 | 0.021340901 |
| MSH6 | blue | -0.058894379 | 0.707554781 |
| MSL3 | blue | 0.226422127 | 0.14428014 |
| MSLNL | blue | 0.119954249 | 0.443565184 |
| MSMO1 | blue | 0.040178996 | 0.79809693 |
| MSN | lightyellow | 0.30602868 | 0.045955657 |
| MSX1 | blue | 0.103743703 | 0.507947724 |
| MT1A | blue | -0.249930918 | 0.106011768 |
| MT2A | blue | -0.039983266 | 0.799060148 |
| MT4 | blue | 0.05035959 | 0.748435794 |
| MTA2 | blue | 0.266995927 | 0.083488557 |
| MTA3 | blue | -0.185607976 | 0.233403866 |
| MTBP | blue | 0.394143582 | 0.008916871 |
| MTFMT | blue | -0.079459046 | 0.612508406 |
| MTFR1L | blue | -0.463235587 | 0.001758666 |
| MTG1 | blue | -0.118162614 | 0.450452185 |
| MTHFR | blue | -0.037953861 | 0.809064295 |
| MTMR11 | blue | 0.244327012 | 0.114339587 |
| MTMR4 | blue | -0.252009446 | 0.103043568 |
| MTMR8 | blue | 0.112691806 | 0.471838385 |
| MTNR1A | blue | -0.023291851 | 0.882142133 |
| MTNR1B | blue | -0.021569338 | 0.890803427 |
| MTRF1L | lightcyan | 0.125989727 | 0.420796336 |
| MTRNR2L1 | blue | -0.359551271 | 0.017881706 |
| MTRNR2L4 | blue | -0.334036174 | 0.028583283 |
| MTRNR2L6 | blue | -0.215961455 | 0.164255645 |
| MTRNR2L7 | blue | -0.329356456 | 0.031031542 |
| MTRNR2L8 | blue | -0.240898037 | 0.119674287 |
| MTX1 | blue | -0.196888674 | 0.205702642 |
| MTX2 | blue | -0.425922235 | 0.004404027 |
| MUC17 | blue | 0.21929567 | 0.157682442 |
| MUC19 | blue | 0.004233488 | 0.978505278 |
| MUC21 | blue | -0.087281498 | 0.577840594 |
| MUC22 | blue | 0.072083108 | 0.645982566 |
| MUC5B | blue | -0.127587968 | 0.414879617 |
| MUC6 | blue | -0.141617132 | 0.365010846 |
| MVK | blue | 0.014604007 | 0.925944999 |
| MVP | blue | 0.225622377 | 0.145740843 |
| MX1 | lightyellow | 0.322911042 | 0.034684854 |
| MX2 | lightyellow | 0.397897781 | 0.008232025 |
| MYBL2 | blue | 0.019806913 | 0.899678402 |
| MYBPC2 | blue | -0.016741316 | 0.915143907 |
| MYBPC3 | blue | 0.007204687 | 0.96342766 |
| MYBPHL | blue | -0.018211731 | 0.907721656 |
| MYCBPAP | blue | -0.182083518 | 0.242557484 |
| MYCN | blue | -0.334654923 | 0.028271907 |
| MYF6 | blue | -0.228821572 | 0.139962526 |
| MYH11 | blue | 0.2538565 | 0.100459689 |
| MYH13 | blue | -0.024583266 | 0.875657361 |
| MYH4 | blue | 0.049193067 | 0.754079543 |
| MYH7 | blue | 0.002026327 | 0.989710789 |
| MYL2 | blue | 0.025006617 | 0.873533253 |
| MYL7 | blue | 0.05201036 | 0.740471401 |
| MYNN | blue | 0.293610849 | 0.056010152 |
| MYO15A | blue | 0.144116631 | 0.356521142 |
| MYO1A | blue | -0.09466478 | 0.54595997 |
| MYOCD | blue | -0.388068793 | 0.010128796 |
| MYOD1 | blue | -0.249428818 | 0.106738475 |
| MYOG | blue | 0.19333525 | 0.21416799 |
| MYT1 | blue | 0.049000155 | 0.755014096 |
| MYT1L | blue | -0.12582348 | 0.421414512 |
| MZB1 | blue | 0.110002722 | 0.482544507 |
| MZT2A | blue | -0.257119959 | 0.096016273 |
| N4BP3 | blue | 0.037135727 | 0.81310598 |
| NAA11 | blue | -0.101176094 | 0.518560648 |
| NAA16 | blue | 0.115616325 | 0.460339583 |
| NAA25 | blue | 0.290174953 | 0.059083367 |
| NAA30 | blue | -0.151385259 | 0.332521401 |
| NAA35 | blue | -0.013085657 | 0.933626235 |
| NAA50 | blue | -0.095013272 | 0.54447622 |
| NAALADL2 | blue | -0.313513137 | 0.04063886 |
| NAB1 | blue | 0.375489555 | 0.013092855 |
| NABP1 | blue | 0.06572325 | 0.675413602 |
| NACA2 | blue | -0.078368476 | 0.61741109 |
| NADK2 | blue | -0.319423283 | 0.036804746 |
| NADSYN1 | blue | 0.155800879 | 0.318444877 |
| NAF1 | blue | 0.005678053 | 0.971173456 |
| NAGLU | blue | -0.00152968 | 0.992232557 |
| NAIF1 | blue | 0.016293754 | 0.917404518 |
| NALCN | blue | 0.234057568 | 0.130874225 |
| NANOGNB | blue | 0.021288975 | 0.892214389 |
| NANOS1 | blue | 0.130181992 | 0.405377998 |
| NANOS3 | blue | -0.138845392 | 0.374566044 |
| NANP | blue | -0.221492035 | 0.153458582 |
| NAP1L1 | blue | -0.026617774 | 0.865457637 |
| NAP1L3 | blue | -0.035858669 | 0.819424355 |
| NAP1L4 | blue | -0.144822782 | 0.354144542 |
| NAPB | blue | 0.14269398 | 0.361338466 |
| NARS2 | blue | -0.362578134 | 0.01687302 |
| NASP | blue | 0.360608723 | 0.017523686 |
| NAT1 | blue | 0.09544624 | 0.542635492 |
| NAT8L | blue | -0.256952527 | 0.096240491 |
| NAV1 | blue | 0.479763658 | 0.001132078 |
| NBEAL1 | blue | 0.123574575 | 0.429827137 |
| NBEAL2 | blue | 0.263517008 | 0.087747234 |
| NBN | blue | -0.04264499 | 0.785987151 |
| NBPF1 | blue | 0.159838755 | 0.30590673 |
| NBPF10 | blue | 0.145791389 | 0.350900352 |
| NBPF14 | blue | 0.155572584 | 0.319163307 |
| NBPF4 | blue | 0.057364591 | 0.714826706 |
| NBPF6 | blue | -0.156548328 | 0.316099849 |
| NBR1 | blue | -0.263311799 | 0.088003609 |
| NCAPD2 | blue | 0.048795192 | 0.756007412 |
| NCAPD3 | blue | 0.142524949 | 0.361913434 |
| NCBP2 | blue | 0.275314921 | 0.073958142 |
| NCK1 | blue | 0.4738266 | 0.00132951 |
| NCKAP1 | blue | 0.148718936 | 0.341205756 |
| NCOA5 | blue | 0.131328898 | 0.401217267 |
| NCOA6 | blue | -0.06818522 | 0.663960751 |
| NCOA7 | blue | -0.044826282 | 0.775316764 |
| NCR1 | blue | 0.121008531 | 0.439539782 |
| NDEL1 | blue | 0.292960961 | 0.056581395 |
| NDOR1 | blue | -0.063083874 | 0.687772546 |
| NDUFA10 | blue | -0.038063004 | 0.808525485 |
| NDUFA2 | blue | -0.316513941 | 0.038653307 |
| NDUFA3 | blue | -0.017607959 | 0.910768427 |
| NDUFA6 | blue | -0.324004248 | 0.034041444 |
| NDUFA9 | blue | -0.117394045 | 0.453424316 |
| NDUFAF2 | blue | -0.25271291 | 0.102053548 |
| NDUFAF5 | blue | 0.131866598 | 0.399275138 |
| NDUFAF6 | blue | 0.197757058 | 0.203669989 |
| NDUFAF7 | blue | 0.382390273 | 0.011386685 |
| NDUFB10 | blue | -0.331842561 | 0.029710179 |
| NDUFB3 | blue | -0.306545348 | 0.04557137 |
| NDUFB9 | blue | -0.299823032 | 0.05077988 |
| NDUFS2 | blue | -0.22010288 | 0.15612034 |
| NDUFS3 | blue | -0.304107552 | 0.047407666 |
| NDUFS4 | blue | -0.302174275 | 0.048906125 |
| NDUFS7 | blue | -0.405788138 | 0.006938914 |
| NDUFS8 | blue | -0.343869482 | 0.023958061 |
| NDUFV2 | blue | -0.363348512 | 0.016624112 |
| NEBL | blue | 0.102778054 | 0.511926207 |
| NECAB3 | blue | 0.030329483 | 0.846906102 |
| NEFH | blue | -0.154662883 | 0.322036206 |
| NEK11 | blue | -0.237558334 | 0.125048482 |
| NEK2 | blue | -0.066493477 | 0.671822645 |
| NEK7 | blue | 0.06819762 | 0.663903256 |
| NEK8 | blue | 0.058938995 | 0.707343081 |
| NELFCD | blue | -0.057895438 | 0.712300435 |
| NENF | blue | -0.0970589 | 0.535805806 |
| NETO1 | blue | -0.10193365 | 0.515417943 |
| NETO2 | blue | 0.147233477 | 0.346104044 |
| NEU1 | blue | -0.058979326 | 0.707151729 |
| NEURL2 | blue | -0.197648301 | 0.203923785 |
| NEXN | blue | 0.311575957 | 0.041964287 |
| NFAM1 | blue | 0.339350669 | 0.025999362 |
| NFATC1 | blue | -0.052997525 | 0.735721381 |
| NFATC2 | blue | 0.295071269 | 0.05474333 |
| NFATC2IP | blue | 0.229015869 | 0.13961714 |
| NFE2L2 | blue | 0.217860896 | 0.160487085 |
| NFE2L3 | blue | 0.081077308 | 0.605264195 |
| NFKBID | blue | 0.085274928 | 0.586648241 |
| NFKBIL1 | blue | -0.075560913 | 0.630107912 |
| NFKBIZ | blue | 0.326101956 | 0.032834334 |
| NFX1 | blue | 0.15997842 | 0.305478772 |
| NFXL1 | blue | 0.222756833 | 0.151064139 |
| NFYA | blue | 0.104782387 | 0.503685847 |
| NFYC | blue | -0.0919311 | 0.55766545 |
| NGF | blue | -0.136672923 | 0.382158389 |
| NHLH1 | blue | 0.112463201 | 0.472743608 |
| NHLRC1 | blue | -0.072808483 | 0.642658403 |
| NHSL2 | blue | 0.342187438 | 0.02470168 |
| NIF3L1 | blue | -0.315708092 | 0.039178553 |
| NIPAL2 | blue | 0.250002227 | 0.105908868 |
| NIPSNAP3A | blue | 0.073907355 | 0.637635744 |
| NKAIN3 | blue | -0.171258398 | 0.27217771 |
| NKIRAS1 | blue | -0.01156475 | 0.941326372 |
| NKRF | blue | 0.04535158 | 0.772753135 |
| NKX1-2 | blue | -0.213266856 | 0.169711338 |
| NKX2-1 | blue | 0.022717845 | 0.885026944 |
| NKX2-2 | blue | -0.047041001 | 0.764524499 |
| NKX2-5 | lightcyan | -0.055388994 | 0.724254725 |
| NKX2-8 | blue | -0.169764449 | 0.276444963 |
| NLGN4X | blue | 0.204483657 | 0.188400589 |
| NLRC5 | blue | 0.39854548 | 0.008118576 |
| NLRP11 | blue | 0.217001612 | 0.162184065 |
| NLRP7 | blue | -0.042277423 | 0.787789082 |
| NME4 | blue | -0.112826994 | 0.471303508 |
| NMI | lightyellow | 0.304053792 | 0.047448826 |
| NMNAT1 | blue | -0.115047071 | 0.462565906 |
| NMNAT3 | blue | -0.100408405 | 0.521755087 |
| NMRAL1 | blue | -0.349644961 | 0.021545506 |
| NMRK1 | blue | -0.200200922 | 0.198025231 |
| NMRK2 | blue | 0.042206408 | 0.788137348 |
| NOG | blue | -0.116754331 | 0.455906256 |
| NOL10 | blue | 0.064095801 | 0.683024461 |
| NOL11 | blue | -0.140074944 | 0.3703091 |
| NOL8 | blue | -0.047439763 | 0.76258595 |
| NOM1 | blue | 0.086155839 | 0.582774192 |
| NOP14 | blue | 0.052760382 | 0.736861581 |
| NOP16 | blue | -0.111286669 | 0.477416905 |
| NOP58 | blue | 0.034191892 | 0.827687854 |
| NOS2 | blue | 0.224956326 | 0.146965647 |
| NOS3 | lightcyan | 0.433957665 | 0.003645004 |
| NOSTRIN | blue | 0.287162854 | 0.061887032 |
| NOTCH1 | blue | 0.203403993 | 0.190794989 |
| NOTCH3 | blue | 0.191855875 | 0.217762644 |
| NOTCH4 | blue | 0.432222696 | 0.003798413 |
| NOTO | blue | -0.0918517 | 0.558007184 |
| NOTUM | blue | -0.10478026 | 0.503694554 |
| NOX3 | blue | 0.066484159 | 0.671866043 |
| NOXO1 | blue | -0.016271744 | 0.917515706 |
| NPAS1 | blue | -0.193400629 | 0.214010086 |
| NPAS2 | blue | -0.350544167 | 0.021188869 |
| NPC1 | blue | 0.09886581 | 0.528203326 |
| NPEPL1 | blue | -0.068373299 | 0.663088885 |
| NPFFR1 | blue | -0.271798539 | 0.077876364 |
| NPM2 | blue | -0.001213008 | 0.993840528 |
| NPPB | blue | -0.039879843 | 0.799569227 |
| NPPC | blue | 0.336756009 | 0.027235494 |
| NPRL2 | blue | 0.195334842 | 0.209375077 |
| NPRL3 | blue | -0.222587537 | 0.151383042 |
| NPS | blue | 0.198489165 | 0.201967292 |
| NPTXR | blue | -0.09212289 | 0.556840396 |
| NPVF | blue | -0.105021374 | 0.502707825 |
| NPW | blue | 0.229716591 | 0.138376769 |
| NPY5R | blue | 0.085426397 | 0.585981298 |
| NR0B1 | blue | -0.231040794 | 0.136055102 |
| NR1D2 | blue | 0.222051806 | 0.152395451 |
| NR2C2 | blue | -0.162702481 | 0.297208241 |
| NR2E1 | blue | 0.010977212 | 0.944302449 |
| NR2F6 | blue | 0.084254024 | 0.591152264 |
| NR3C1 | blue | 0.041521034 | 0.791500573 |
| NR5A1 | blue | -0.017784397 | 0.90987795 |
| NR6A1 | blue | -0.206440707 | 0.184114935 |
| NRAP | blue | -0.151638858 | 0.331702637 |
| NRAS | blue | 0.116260097 | 0.457828797 |
| NRBF2 | blue | -0.034604216 | 0.825641898 |
| NRBP1 | lightcyan | 0.176507446 | 0.257530253 |
| NRDE2 | blue | 0.088816878 | 0.571141939 |
| NRF1 | blue | 0.291730735 | 0.057675523 |
| NRG1 | blue | -0.23741048 | 0.125290526 |
| NRG2 | blue | 0.078750209 | 0.615693119 |
| NRG4 | blue | 0.278602969 | 0.070436322 |
| NRGN | blue | 0.103427923 | 0.509247022 |
| NRM | blue | 0.177066505 | 0.256001848 |
| NRN1L | blue | 0.115330071 | 0.461458388 |
| NRSN1 | blue | 0.204773791 | 0.187760804 |
| NRXN2 | blue | -0.110217553 | 0.48168455 |
| NSA2 | blue | -0.348043006 | 0.022193327 |
| NSDHL | blue | 0.100791261 | 0.520160763 |
| NSMCE1 | blue | -0.368463785 | 0.015048844 |
| NSUN2 | blue | 0.217165531 | 0.161859344 |
| NSUN4 | blue | 0.222452348 | 0.151638051 |
| NT5C3B | blue | -0.080377186 | 0.608393784 |
| NT5DC1 | blue | -0.192847834 | 0.215347754 |
| NT5DC4 | blue | 0.071436905 | 0.648949653 |
| NT5E | blue | 0.355478243 | 0.019318886 |
| NTF3 | blue | 0.146131557 | 0.349765333 |
| NTMT1 | blue | -0.276820975 | 0.072328191 |
| NTN3 | blue | -0.224436338 | 0.14792711 |
| NTNG2 | blue | 0.001729574 | 0.991217566 |
| NTPCR | blue | -0.321792939 | 0.035353226 |
| NUAK2 | blue | -0.361361528 | 0.017272513 |
| NUB1 | lightyellow | 0.275491274 | 0.073765801 |
| NUDC | blue | -0.279915775 | 0.069067739 |
| NUDCD3 | blue | -0.040557204 | 0.796236541 |
| NUDT10 | blue | -0.18970291 | 0.22306829 |
| NUDT14 | blue | -0.105126748 | 0.502276902 |
| NUDT19 | blue | -0.016385408 | 0.916941529 |
| NUDT6 | blue | 0.063705813 | 0.684852916 |
| NUFIP1 | blue | -0.228984343 | 0.139673138 |
| NUMB | blue | 0.255164648 | 0.098659985 |
| NUP107 | blue | 0.092903493 | 0.553488265 |
| NUP155 | blue | -0.142504487 | 0.361983075 |
| NUP188 | blue | 0.214329327 | 0.167544769 |
| NUP210 | blue | -0.187755755 | 0.22794286 |
| NUP214 | blue | 0.126829804 | 0.41768043 |
| NUP43 | blue | 0.038129018 | 0.808199631 |
| NUP50 | lightcyan | 0.181821682 | 0.24324708 |
| NUP54 | blue | 0.10405026 | 0.506687983 |
| NUP85 | blue | 0.024706498 | 0.875038974 |
| NUP88 | blue | 0.108281907 | 0.489461725 |
| NUP93 | blue | 0.271653946 | 0.078040896 |
| NUS1 | blue | -0.068436722 | 0.662794977 |
| NUTM2F | blue | 0.340773082 | 0.025341759 |
| NUTM2G | blue | 0.3283746 | 0.0315666 |
| NXF2B | blue | -0.079657793 | 0.611616723 |
| NXPE4 | blue | -0.277586107 | 0.071511034 |
| NXT1 | blue | 0.011635423 | 0.940968445 |
| NYAP1 | blue | 0.073777242 | 0.638229626 |
| NYAP2 | blue | 0.057162466 | 0.715789401 |
| NYNRIN | blue | 0.389951093 | 0.009739025 |
| OAS1 | lightyellow | 0.329853413 | 0.030763597 |
| OAS2 | lightyellow | 0.413877876 | 0.005799299 |
| OAS3 | lightyellow | 0.340267667 | 0.025573815 |
| OASL | lightyellow | 0.226476157 | 0.144181847 |
| OAT | blue | -0.393020273 | 0.009131076 |
| OBP2A | blue | -0.029767928 | 0.849707832 |
| OBP2B | blue | 0.285173555 | 0.063796083 |
| OCEL1 | blue | -0.291928726 | 0.057498301 |
| ODF1 | lightcyan | -0.060321806 | 0.700792509 |
| ODF3L2 | blue | 0.140456309 | 0.368994641 |
| OFD1 | blue | 0.207323026 | 0.18220569 |
| OGFOD2 | blue | -0.092670284 | 0.554488735 |
| OGFR | blue | -0.102240525 | 0.514147594 |
| OGFRL1 | blue | 0.097252346 | 0.534989363 |
| OGN | blue | 0.291491286 | 0.057890438 |
| OIT3 | blue | -0.050126946 | 0.749560323 |
| OLA1 | blue | -0.111503756 | 0.476552787 |
| OLFM1 | blue | -0.270780728 | 0.079040335 |
| OLIG1 | blue | -0.273407871 | 0.076063399 |
| OLIG3 | blue | -0.092541241 | 0.555042693 |
| OMA1 | blue | -0.099392713 | 0.525996419 |
| OMD | blue | 0.074396751 | 0.635403971 |
| ONECUT2 | blue | -0.249108375 | 0.107204246 |
| OOEP | blue | 0.049451356 | 0.752828812 |
| OPA1 | blue | -0.157061875 | 0.314495002 |
| OPALIN | blue | -0.059150251 | 0.706340965 |
| OPLAH | blue | -0.163877893 | 0.293684522 |
| OPN1LW | blue | -0.513349366 | 0.000430919 |
| OPN1SW | blue | 0.052745583 | 0.736932754 |
| OPN3 | blue | -0.149137643 | 0.339832831 |
| OPRK1 | blue | -0.138252385 | 0.376629519 |
| OPRL1 | blue | -0.091919391 | 0.557715841 |
| OPRM1 | lightcyan | -0.179162327 | 0.250326189 |
| OPTC | blue | -0.274286408 | 0.07508778 |
| OR10A4 | blue | 0.109266047 | 0.485499467 |
| OR10A7 | blue | 0.237587318 | 0.125001075 |
| OR10AD1 | blue | 0.080564263 | 0.607556858 |
| OR10D3 | blue | -0.041920541 | 0.78953968 |
| OR10H4 | blue | 0.185697094 | 0.233175511 |
| OR10H5 | blue | -0.062820483 | 0.689010363 |
| OR10P1 | blue | 0.066992956 | 0.669497814 |
| OR10Q1 | blue | -0.185073722 | 0.234776032 |
| OR10S1 | blue | -0.080994357 | 0.605634631 |
| OR10T2 | blue | -0.053866416 | 0.7315485 |
| OR10V1 | blue | 0.09232883 | 0.555955105 |
| OR11H12 | blue | 0.002121189 | 0.989229128 |
| OR12D2 | blue | -0.184766035 | 0.235568778 |
| OR13C2 | blue | -0.016080282 | 0.918482986 |
| OR13C3 | blue | 0.000144428 | 0.999266609 |
| OR13C5 | blue | 0.040283693 | 0.797581819 |
| OR13C9 | blue | 0.340980574 | 0.025247001 |
| OR13G1 | blue | -0.01953927 | 0.901027245 |
| OR14J1 | blue | 0.033502214 | 0.831112555 |
| OR14K1 | lightyellow | 0.047191964 | 0.76379044 |
| OR1A1 | blue | -0.20257739 | 0.192642691 |
| OR1C1 | blue | 0.014083756 | 0.928576205 |
| OR1D2 | lightcyan | -0.32864954 | 0.031416011 |
| OR1D5 | blue | 0.00630671 | 0.967983438 |
| OR1G1 | blue | -0.199245463 | 0.200218851 |
| OR1I1 | blue | 0.017656473 | 0.910523569 |
| OR1K1 | blue | -0.054192884 | 0.72998259 |
| OR1L3 | blue | 0.1576836 | 0.312559012 |
| OR1L6 | blue | 0.16988278 | 0.276105375 |
| OR1S1 | blue | -0.205129195 | 0.186979197 |
| OR2A12 | blue | 0.074611607 | 0.634425175 |
| OR2A25 | blue | -0.067641156 | 0.6664853 |
| OR2A7 | blue | -0.171559852 | 0.271321942 |
| OR2D2 | blue | 0.336159252 | 0.027526598 |
| OR2G3 | blue | -0.072979559 | 0.641875417 |
| OR2J2 | lightcyan | -0.146142597 | 0.349728533 |
| OR2J3 | blue | -0.077965973 | 0.619224725 |
| OR2L13 | blue | 0.023435847 | 0.881418684 |
| OR2L5 | blue | -0.039781194 | 0.80005489 |
| OR2M7 | blue | -0.153075068 | 0.327089434 |
| OR2T12 | blue | -0.279785962 | 0.069202123 |
| OR2T29 | blue | -0.456903322 | 0.00207015 |
| OR2T34 | blue | -0.306444945 | 0.045645843 |
| OR2T5 | blue | -0.392811573 | 0.009171356 |
| OR2T8 | blue | -0.084558667 | 0.589806641 |
| OR2V1 | blue | -0.07693689 | 0.623871796 |
| OR2W1 | blue | 0.117960113 | 0.451234244 |
| OR2Y1 | blue | 0.031152163 | 0.842804932 |
| OR3A3 | blue | 0.060042958 | 0.702111744 |
| OR4A47 | blue | 0.099435706 | 0.525816544 |
| OR4A5 | blue | -0.252255831 | 0.102695985 |
| OR4C13 | blue | 0.069472111 | 0.658003992 |
| OR4C15 | blue | 0.025847058 | 0.869319068 |
| OR4C16 | blue | -0.027486564 | 0.861108545 |
| OR4C46 | blue | -0.048081053 | 0.759471378 |
| OR4D10 | blue | -0.051476479 | 0.743044309 |
| OR4D11 | blue | -0.103119811 | 0.510516378 |
| OR4D2 | blue | -0.032287424 | 0.83715226 |
| OR4F15 | blue | 0.038317571 | 0.80726909 |
| OR4F21 | blue | 0.200379562 | 0.19761698 |
| OR4F29 | blue | 0.250812494 | 0.104744979 |
| OR4F6 | blue | 0.213598564 | 0.169032767 |
| OR4K1 | blue | -0.073615151 | 0.638969783 |
| OR4K15 | blue | 0.08005418 | 0.609839976 |
| OR4K2 | blue | 0.205493933 | 0.186179469 |
| OR4M1 | blue | -0.069013385 | 0.660124971 |
| OR4N2 | blue | -0.096250357 | 0.539224804 |
| OR4P4 | blue | -0.035317975 | 0.822102921 |
| OR4S1 | blue | 0.036377224 | 0.816857363 |
| OR4S2 | blue | -0.065936451 | 0.6744189 |
| OR51A4 | blue | -0.042293729 | 0.787709123 |
| OR51A7 | blue | 0.040495426 | 0.796540348 |
| OR51C1P | blue | 0.012206571 | 0.93807624 |
| OR51E1 | blue | 0.345370878 | 0.023310182 |
| OR51E2 | blue | 0.354980438 | 0.019501023 |
| OR51F1 | blue | 0.077080478 | 0.623222517 |
| OR51G1 | blue | 0.107535699 | 0.492477179 |
| OR51G2 | blue | -0.001152675 | 0.994146882 |
| OR51I1 | blue | 0.109489837 | 0.484600802 |
| OR51L1 | blue | -0.160281073 | 0.304552698 |
| OR51M1 | blue | -0.072945418 | 0.642031644 |
| OR51T1 | blue | -0.148050723 | 0.34340387 |
| OR52B2 | blue | -0.116924312 | 0.455246054 |
| OR52E2 | blue | -0.295614195 | 0.054278284 |
| OR52E6 | blue | 0.021550688 | 0.890897276 |
| OR52H1 | blue | -0.127103286 | 0.416668898 |
| OR52I2 | blue | -0.132707607 | 0.396248429 |
| OR52K2 | blue | -0.11457593 | 0.46441288 |
| OR52L1 | blue | -0.216552115 | 0.163076943 |
| OR52N1 | blue | 0.270208655 | 0.079700514 |
| OR52N5 | blue | -0.028336081 | 0.856859851 |
| OR56B1 | lightyellow | 0.467876066 | 0.001557493 |
| OR5A1 | blue | 0.137798804 | 0.378212383 |
| OR5A2 | lightcyan | -0.138781341 | 0.374788596 |
| OR5AK2 | blue | 0.213370882 | 0.169498321 |
| OR5AN1 | blue | 0.079443793 | 0.612576861 |
| OR5AR1 | blue | -0.263791883 | 0.08740473 |
| OR5AS1 | blue | 0.118354074 | 0.449713446 |
| OR5B2 | lightcyan | -0.133520715 | 0.393334859 |
| OR5C1 | blue | -0.13484421 | 0.38861925 |
| OR5D18 | blue | -0.392497575 | 0.009232247 |
| OR5H1 | blue | -0.052257122 | 0.739283139 |
| OR5H15 | blue | 0.196906213 | 0.205661447 |
| OR5I1 | blue | 0.008609312 | 0.95630383 |
| OR5K1 | blue | -0.113541447 | 0.468482097 |
| OR5K4 | blue | 0.030365789 | 0.846725028 |
| OR5M8 | blue | 0.039530351 | 0.801290144 |
| OR5P3 | blue | -0.465732655 | 0.001647728 |
| OR5W2 | blue | 0.015336571 | 0.922241341 |
| OR6C3 | blue | -0.07842273 | 0.617166799 |
| OR6C6 | blue | -0.368778722 | 0.014956143 |
| OR6C68 | blue | -0.092838441 | 0.553767251 |
| OR6C70 | blue | -0.05156026 | 0.742640364 |
| OR6C74 | blue | -0.11457213 | 0.464427791 |
| OR6F1 | blue | -0.134624604 | 0.389399403 |
| OR6K6 | blue | 0.154310411 | 0.32315369 |
| OR6N1 | blue | 0.054271131 | 0.729607435 |
| OR6T1 | blue | 0.205690308 | 0.185749907 |
| OR6V1 | blue | -0.006339401 | 0.967817565 |
| OR6X1 | blue | -0.171877155 | 0.270423102 |
| OR6Y1 | blue | -0.315147601 | 0.039547306 |
| OR7A17 | blue | 0.252759608 | 0.101988086 |
| OR7C1 | blue | 0.282253806 | 0.066682529 |
| OR7G2 | lightcyan | -0.33221522 | 0.029516186 |
| OR7G3 | blue | -0.216010324 | 0.16415789 |
| OR8B4 | blue | 0.035531744 | 0.82104368 |
| OR8D1 | blue | 0.012513165 | 0.936524019 |
| OR8D4 | blue | -0.134755318 | 0.388934931 |
| OR8G5 | blue | 0.155678754 | 0.318829068 |
| OR8H3 | blue | -0.164257272 | 0.292552983 |
| OR8J3 | blue | -0.01196487 | 0.939300079 |
| OR9A4 | blue | 0.112991638 | 0.470652524 |
| OR9G1 | blue | -0.099093525 | 0.527248993 |
| OR9I1 | blue | 0.07773602 | 0.620261872 |
| OR9Q2 | blue | -0.247089096 | 0.110175058 |
| ORC5 | blue | 0.075947886 | 0.628351505 |
| OSBP2 | blue | 0.224086458 | 0.148576641 |
| OSBPL10 | blue | -0.171633735 | 0.271112474 |
| OSBPL5 | blue | -0.040173406 | 0.798124433 |
| OSBPL7 | blue | 0.178762233 | 0.251403087 |
| OSBPL9 | blue | -0.348700623 | 0.021925445 |
| OSCP1 | blue | -0.296165809 | 0.05380905 |
| OSGIN1 | blue | -0.112379617 | 0.473074807 |
| OSGIN2 | blue | -0.275365921 | 0.073902479 |
| OSM | blue | 0.136778618 | 0.381786918 |
| OSR2 | lightcyan | -0.296309645 | 0.053687233 |
| OSTM1 | blue | 0.23523917 | 0.128885657 |
| OTOF | blue | 0.014312422 | 0.927419614 |
| OTOL1 | blue | -0.080603376 | 0.607381941 |
| OTOP1 | blue | -0.314081441 | 0.040256572 |
| OTOP2 | blue | -0.235631229 | 0.128230864 |
| OTUB1 | blue | -0.286802483 | 0.062229448 |
| OTUB2 | blue | 0.248371892 | 0.108280617 |
| OTUD4 | blue | 0.257070729 | 0.096082158 |
| OTUD7B | blue | 0.094213523 | 0.547884115 |
| OXNAD1 | blue | -0.128474664 | 0.41161758 |
| OXSM | blue | 0.059546951 | 0.704460504 |
| OXT | blue | -0.361199685 | 0.017326254 |
| P2RX1 | blue | 0.188068036 | 0.227156207 |
| P2RX4 | blue | -0.030903669 | 0.844043281 |
| P2RX5 | blue | 0.03242757 | 0.836455 |
| P2RX6 | blue | 0.06391343 | 0.683879284 |
| P2RY10 | blue | 0.081125431 | 0.605049337 |
| PABPC1 | blue | -0.185834171 | 0.232824567 |
| PABPC1L2A | blue | 0.237781192 | 0.124684319 |
| PADI1 | blue | -0.12624632 | 0.419843224 |
| PADI2 | blue | -0.237617752 | 0.124951313 |
| PADI6 | blue | -0.239690123 | 0.121597556 |
| PAEP | blue | -0.102691087 | 0.512285281 |
| PAG1 | blue | 0.024983742 | 0.873648004 |
| PAGE2 | blue | -0.004934523 | 0.974946951 |
| PAGE2B | lightcyan | -0.22063604 | 0.155094795 |
| PAGE4 | blue | -0.285478602 | 0.06350034 |
| PAGE5 | blue | -0.11505743 | 0.462525343 |
| PAK4 | blue | -0.37802924 | 0.012441211 |
| PALB2 | blue | -0.283579934 | 0.065358953 |
| PALM3 | blue | -0.158125119 | 0.311188767 |
| PAN2 | blue | 0.095710834 | 0.541512068 |
| PAN3 | blue | 0.183762866 | 0.238166071 |
| PANK2 | blue | 0.21193477 | 0.172456179 |
| PAOX | blue | -0.229243785 | 0.139212797 |
| PAPOLA | blue | 0.323676157 | 0.034233503 |
| PAPOLB | blue | 0.303796925 | 0.047645891 |
| PAPOLG | blue | 0.148981811 | 0.3403434 |
| PAPSS2 | blue | -0.189862139 | 0.222672873 |
| PAQR3 | blue | 0.098360235 | 0.53032516 |
| PARD6A | blue | 0.113419694 | 0.468962271 |
| PARD6B | lightcyan | -0.490304055 | 0.000844902 |
| PARP11 | blue | 0.031043651 | 0.843345649 |
| PARP12 | lightyellow | 0.375785695 | 0.013015404 |
| PARP14 | lightyellow | 0.430861509 | 0.003922696 |
| PARP15 | blue | 0.121840089 | 0.436379072 |
| PARP3 | blue | 0.166508551 | 0.28589636 |
| PARP6 | blue | 0.315621419 | 0.039235392 |
| PARP9 | lightyellow | 0.481957395 | 0.001066012 |
| PARPBP | blue | 0.095032241 | 0.54439551 |
| PARVB | lightcyan | 0.416274731 | 0.005494509 |
| PASK | blue | 0.113301236 | 0.469429697 |
| PATE1 | blue | 0.081266898 | 0.604417914 |
| PATE2 | blue | 0.174713168 | 0.262476721 |
| PATE3 | blue | -0.052104938 | 0.740015898 |
| PATL1 | blue | 0.539450803 | 0.000189309 |
| PAWR | lightcyan | -0.338977973 | 0.026173994 |
| PAX1 | lightcyan | -0.269953112 | 0.079996804 |
| PAX3 | blue | -0.018937554 | 0.904060721 |
| PAX4 | blue | -0.083092827 | 0.596293756 |
| PAX5 | blue | -0.044943282 | 0.774745558 |
| PC | blue | -0.263503329 | 0.087764305 |
| PCBD1 | blue | -0.380836937 | 0.011753179 |
| PCBP2 | blue | -0.353604979 | 0.020011797 |
| PCBP3 | blue | 0.10551609 | 0.500686341 |
| PCDH1 | lightcyan | 0.360874439 | 0.017434681 |
| PCDH12 | lightcyan | 0.383443878 | 0.011143694 |
| PCDH7 | blue | 0.089325213 | 0.568932014 |
| PCDH8 | blue | -0.105529287 | 0.500632473 |
| PCDHA7 | blue | -0.254414409 | 0.099689081 |
| PCDHB1 | blue | -0.164694158 | 0.291253418 |
| PCDHB11 | blue | -0.253977421 | 0.100292282 |
| PCDHB6 | blue | -0.154544124 | 0.322412448 |
| PCDHB8 | blue | 0.16982636 | 0.276267255 |
| PCDHGB2 | blue | 0.024290047 | 0.877129058 |
| PCGF2 | blue | -0.046943288 | 0.764999742 |
| PCP4 | blue | -0.304683509 | 0.046968506 |
| PCSK1 | blue | 0.053329929 | 0.734124102 |
| PCSK2 | lightcyan | 0.31836936 | 0.037465837 |
| PCYOX1 | blue | -0.346951455 | 0.022644021 |
| PDCD10 | blue | 0.174404171 | 0.263334896 |
| PDCD1LG2 | lightyellow | 0.126284893 | 0.419700049 |
| PDCD6IP | blue | 0.156012731 | 0.317779106 |
| PDE11A | blue | -0.165283656 | 0.289505827 |
| PDE2A | blue | 0.291383005 | 0.057987834 |
| PDE3A | blue | 0.152010309 | 0.330505647 |
| PDE3B | blue | -0.21357943 | 0.169071855 |
| PDE4DIP | blue | 0.040550715 | 0.796268452 |
| PDE6A | blue | 0.0043352 | 0.977988978 |
| PDE6H | blue | -0.233630536 | 0.131598501 |
| PDGFA | blue | -0.047583319 | 0.761888415 |
| PDGFRA | blue | 0.088431809 | 0.572818595 |
| PDGFRL | blue | -0.259063651 | 0.093442707 |
| PDHX | blue | 0.074294885 | 0.635868244 |
| PDIK1L | blue | -0.142955771 | 0.360449063 |
| PDK1 | blue | 0.197044438 | 0.205337 |
| PDLIM3 | blue | 0.217794038 | 0.160618657 |
| PDLIM7 | blue | 0.233114708 | 0.132477367 |
| PDP1 | blue | 0.090025503 | 0.56589404 |
| PDPK1 | blue | 0.118521527 | 0.449067879 |
| PDS5A | blue | 0.355917773 | 0.019159263 |
| PDS5B | blue | 0.272379122 | 0.077218452 |
| PDX1 | blue | -0.0729031 | 0.642225309 |
| PDXDC1 | blue | -0.046341225 | 0.76792984 |
| PDXK | blue | -0.237112343 | 0.125779656 |
| PEAK1 | lightcyan | 0.39309487 | 0.009116715 |
| PEAR1 | blue | -0.075495711 | 0.630404049 |
| PEG10 | blue | -0.505640674 | 0.000542633 |
| PELP1 | blue | -0.052393225 | 0.738627997 |
| PEX11A | blue | 0.078652396 | 0.616133131 |
| PEX11G | blue | -0.612117758 | 1.29E-05 |
| PEX12 | blue | -0.312122095 | 0.041587102 |
| PEX14 | blue | -0.292912139 | 0.056624496 |
| PEX3 | blue | 0.072188008 | 0.645501419 |
| PEX5L | blue | 0.096542913 | 0.537986491 |
| PEX7 | blue | -0.243645759 | 0.115384849 |
| PF4V1 | blue | -0.10175869 | 0.516142918 |
| PFDN4 | blue | 0.092200858 | 0.556505149 |
| PFDN6 | blue | -0.088170735 | 0.573956635 |
| PFKFB4 | blue | -0.090522772 | 0.563741369 |
| PFKL | blue | -0.244120749 | 0.114655301 |
| PFKM | blue | -0.386698883 | 0.010420803 |
| PFN4 | blue | 0.289989132 | 0.059253339 |
| PGAM2 | blue | -0.220709038 | 0.154954765 |
| PGBD4 | blue | -0.180055958 | 0.24793209 |
| PGC | blue | 0.065662671 | 0.67569634 |
| PGGT1B | blue | 0.099177068 | 0.526899088 |
| PGK1 | blue | -0.280838772 | 0.068118183 |
| PGLYRP2 | blue | -0.010841603 | 0.944989461 |
| PGM2 | blue | 0.299452637 | 0.051080265 |
| PGRMC2 | blue | -0.160325049 | 0.304418289 |
| PHACTR1 | blue | 0.062380444 | 0.691080136 |
| PHB | blue | -0.32579594 | 0.033008198 |
| PHB2 | blue | -0.187909982 | 0.22755412 |
| PHC3 | blue | 0.079132796 | 0.613973335 |
| PHEX | blue | 0.18163586 | 0.243737283 |
| PHF11 | blue | 0.320102895 | 0.036383542 |
| PHF14 | blue | 0.178962595 | 0.2508634 |
| PHF20L1 | lightcyan | 0.519539893 | 0.00035667 |
| PHF5A | blue | -0.045691766 | 0.771094185 |
| PHKB | blue | -0.113308293 | 0.469401844 |
| PHLDA1 | blue | -0.313172787 | 0.040869223 |
| PHLPP1 | blue | -0.131896024 | 0.399169013 |
| PHOSPHO2 | blue | -0.005863602 | 0.970231872 |
| PHOX2B | blue | -0.160463721 | 0.30399469 |
| PHTF2 | blue | 0.215097006 | 0.165991831 |
| PI4K2B | blue | -0.153827588 | 0.324688388 |
| PI4KB | blue | 0.088976787 | 0.570446328 |
| PIANP | blue | -0.04210832 | 0.788618449 |
| PIFO | blue | -0.362637123 | 0.01685385 |
| PIGA | blue | -0.246405939 | 0.111194183 |
| PIGN | blue | 0.025587881 | 0.870618272 |
| PIGQ | blue | -0.180382292 | 0.247061678 |
| PIGV | blue | -0.173044435 | 0.267133421 |
| PIGX | blue | -0.022172604 | 0.887768549 |
| PIH1D2 | blue | -0.264746417 | 0.08622341 |
| PIK3C2A | blue | 0.416427512 | 0.005475562 |
| PIK3C3 | blue | 0.082260413 | 0.599991502 |
| PIK3CB | blue | -0.063212161 | 0.687169947 |
| PIK3R2 | blue | -0.319663325 | 0.036655521 |
| PIK3R3 | lightcyan | 0.375219393 | 0.013163853 |
| PIK3R6 | blue | 0.008990607 | 0.954370574 |
| PIKFYVE | blue | 0.35534444 | 0.019367701 |
| PIM1 | blue | -0.14413976 | 0.356443146 |
| PIP | blue | -0.104124793 | 0.506381939 |
| PIP5KL1 | blue | -0.132238441 | 0.397935262 |
| PIRT | blue | -0.393218239 | 0.009093007 |
| PITHD1 | blue | -0.341836649 | 0.024859161 |
| PITPNB | lightcyan | 0.533076034 | 0.000232869 |
| PITPNC1 | blue | 0.278337475 | 0.070715681 |
| PITRM1 | blue | -0.16154739 | 0.300697442 |
| PITX1 | lightcyan | -0.399065124 | 0.008028532 |
| PIWIL1 | blue | 0.137531886 | 0.379145691 |
| PIWIL2 | blue | -0.183769545 | 0.238148714 |
| PIWIL3 | blue | 0.059814113 | 0.703195062 |
| PKD1L2 | blue | -0.123160465 | 0.431386395 |
| PKD1L3 | blue | 0.030812165 | 0.844499379 |
| PKD2L1 | blue | 0.087341837 | 0.577576675 |
| PKD2L2 | blue | 0.201110951 | 0.195951702 |
| PKIB | blue | 0.234407255 | 0.130283349 |
| PKNOX2 | blue | 0.0387167 | 0.80530019 |
| PLA1A | lightyellow | 0.242719186 | 0.116818101 |
| PLA2G10 | blue | 0.271323668 | 0.078417744 |
| PLA2G12A | blue | -0.116078868 | 0.458534862 |
| PLA2G1B | blue | -0.182273879 | 0.242056965 |
| PLA2G2D | blue | 0.279707668 | 0.069283274 |
| PLA2G4A | blue | 0.263461498 | 0.087816527 |
| PLA2G4E | blue | -0.087296738 | 0.577773932 |
| PLAC8L1 | blue | 0.182416751 | 0.241681767 |
| PLAC9 | blue | 0.238554607 | 0.123426685 |
| PLB1 | blue | 0.150823212 | 0.334340484 |
| PLCB3 | blue | 0.17267096 | 0.26818307 |
| PLCB4 | blue | -0.098558846 | 0.529491117 |
| PLCZ1 | blue | -0.307225242 | 0.045069656 |
| PLD6 | blue | -0.084484098 | 0.590135889 |
| PLEC | blue | 0.176356729 | 0.257943338 |
| PLEKHA2 | blue | -0.092206427 | 0.556481207 |
| PLEKHA6 | blue | 0.015976361 | 0.919008048 |
| PLEKHA8 | blue | -0.036759442 | 0.814966489 |
| PLEKHD1 | blue | 0.081593347 | 0.60296193 |
| PLEKHF1 | blue | -0.042428658 | 0.787047549 |
| PLEKHG4 | blue | -0.031976425 | 0.838699989 |
| PLEKHG4B | blue | -0.26101093 | 0.090918151 |
| PLEKHG7 | blue | 0.222177583 | 0.152157316 |
| PLEKHJ1 | blue | -0.424371953 | 0.004565363 |
| PLEKHM1 | blue | 0.008223167 | 0.958261927 |
| PLEKHN1 | blue | -0.065561517 | 0.676168541 |
| PLEKHO2 | blue | 0.333605885 | 0.02880149 |
| PLIN2 | blue | 0.143478365 | 0.358677571 |
| PLIN4 | blue | -0.276184539 | 0.073013495 |
| PLIN5 | blue | -0.223534101 | 0.149606323 |
| PLK4 | blue | -0.135240245 | 0.38721465 |
| PLK5 | blue | 0.074152238 | 0.636518614 |
| PLOD1 | blue | -0.118844223 | 0.447825248 |
| PLP1 | blue | 0.07313905 | 0.641145798 |
| PLSCR1 | lightyellow | 0.476544695 | 0.001235617 |
| PLSCR3 | blue | 0.336749893 | 0.027238465 |
| PLXND1 | lightcyan | 0.39479732 | 0.008794208 |
| PMCH | blue | -0.107285255 | 0.493491377 |
| PML | lightyellow | 0.403574347 | 0.007282654 |
| PMM1 | blue | -0.346762673 | 0.022722739 |
| PMPCA | blue | -0.145992471 | 0.350229141 |
| PMS1 | blue | 0.0020228 | 0.989728696 |
| PNCK | blue | -0.080379472 | 0.608383552 |
| PNKD | blue | -0.180756775 | 0.246065382 |
| PNLIP | blue | -0.247104262 | 0.110152514 |
| PNLIPRP1 | blue | 0.279672422 | 0.06931983 |
| PNLIPRP2 | blue | -0.080030598 | 0.609945619 |
| PNLIPRP3 | blue | -0.345993118 | 0.023046 |
| PNMA5 | blue | -0.1224543 | 0.434052605 |
| PNMT | blue | -0.229680938 | 0.138439681 |
| PNN | blue | 0.201788934 | 0.194416889 |
| PNO1 | lightcyan | 0.315246306 | 0.039482162 |
| POC5 | blue | 0.011224878 | 0.943047845 |
| PODNL1 | blue | -0.005939978 | 0.969844302 |
| POLD1 | blue | -0.17363177 | 0.265488228 |
| POLD2 | blue | -0.352532393 | 0.020417858 |
| POLE | blue | 0.324777874 | 0.033592085 |
| POLH | blue | 0.01430944 | 0.927434696 |
| POLL | blue | -0.14806731 | 0.343349201 |
| POLR1C | blue | -0.097298675 | 0.534793918 |
| POLR2C | blue | -0.292218142 | 0.057240029 |
| POLR2D | blue | -0.371537995 | 0.014164461 |
| POLR2E | blue | -0.182846575 | 0.240555381 |
| POLR2H | blue | -0.141855106 | 0.364197355 |
| POLR2J | blue | -0.406936373 | 0.006766216 |
| POLR2L | blue | 0.019721217 | 0.900110254 |
| POLR3B | blue | -0.206418331 | 0.184163539 |
| POLR3D | blue | 0.2030362 | 0.191615558 |
| POLR3E | blue | 0.183694633 | 0.238343437 |
| POLRMT | blue | -0.427949458 | 0.004200576 |
| POM121L2 | blue | -0.0909046 | 0.562091027 |
| POMGNT1 | blue | -0.041557803 | 0.791320046 |
| POMT1 | blue | -0.085705322 | 0.584754029 |
| PON1 | blue | -0.06912033 | 0.659630258 |
| PON3 | blue | 0.082458215 | 0.599111922 |
| POP1 | blue | -0.18586358 | 0.232749321 |
| POP5 | blue | -0.236859624 | 0.126195395 |
| POPDC2 | blue | 0.344106132 | 0.023854956 |
| PORCN | blue | -0.357314067 | 0.018659518 |
| POT1 | blue | -0.245274694 | 0.112897491 |
| POTEB | blue | -0.081375628 | 0.603932801 |
| POTEC | blue | -0.167272983 | 0.28365867 |
| POTEE | blue | -0.10946301 | 0.484708484 |
| POTEF | blue | -0.19479611 | 0.21065896 |
| POTEG | blue | -0.181181469 | 0.24493879 |
| POTEM | blue | -0.229770895 | 0.138280986 |
| POU2AF1 | blue | -0.087552595 | 0.576655254 |
| POU2F1 | blue | 0.159308898 | 0.307533788 |
| POU2F2 | blue | 0.358034153 | 0.018406113 |
| POU2F3 | blue | -0.148239259 | 0.342782793 |
| POU3F1 | blue | 0.027587003 | 0.860606014 |
| POU3F4 | blue | -0.298062327 | 0.052220587 |
| POU4F2 | blue | 0.179123054 | 0.250431759 |
| POU5F1B | blue | -0.01458795 | 0.9260262 |
| POU5F2 | blue | 0.131470206 | 0.400706343 |
| PPCS | blue | -0.114686368 | 0.463979586 |
| PPEF1 | blue | -0.130310572 | 0.404910304 |
| PPFIA4 | blue | 0.096682276 | 0.537397085 |
| PPFIBP1 | blue | 0.143241475 | 0.359479928 |
| PPIE | blue | -0.178123975 | 0.253127465 |
| PPIF | blue | -0.214916215 | 0.166356612 |
| PPIL6 | blue | -0.223635604 | 0.149416712 |
| PPIP5K1 | blue | 0.197538695 | 0.204179787 |
| PPM1B | blue | 0.142336985 | 0.362553447 |
| PPM1K | blue | -0.19563599 | 0.208659775 |
| PPM1L | blue | -0.171541968 | 0.271372664 |
| PPM1N | blue | -0.063325018 | 0.686639986 |
| PPOX | blue | -0.130683812 | 0.403554456 |
| PPP1R12A | blue | 0.062924727 | 0.688520368 |
| PPP1R13L | blue | 0.1409911 | 0.367156085 |
| PPP1R14A | blue | 0.44492045 | 0.00279515 |
| PPP1R16A | blue | -0.356707124 | 0.018875382 |
| PPP1R1B | blue | -0.047799993 | 0.76083595 |
| PPP1R26 | blue | -0.194519846 | 0.211319466 |
| PPP1R27 | blue | -0.148563175 | 0.341717363 |
| PPP1R37 | blue | -0.457836747 | 0.002021374 |
| PPP1R3E | blue | -0.265754221 | 0.084989678 |
| PPP1R3G | blue | -0.352804967 | 0.020314016 |
| PPP2R3B | blue | -0.20193909 | 0.194078118 |
| PPP2R3C | lightcyan | 0.260238286 | 0.091913451 |
| PPP2R5A | blue | -0.22036819 | 0.155609393 |
| PPP2R5B | blue | 0.040040834 | 0.798776816 |
| PPP2R5C | blue | -0.189323656 | 0.224012048 |
| PPP3CC | blue | 0.353359837 | 0.020104 |
| PPP4R1 | blue | 0.174049191 | 0.264323081 |
| PPP4R4 | blue | 0.175463633 | 0.260400208 |
| PPP6R1 | blue | -0.001178838 | 0.994014031 |
| PPP6R3 | blue | -0.031694248 | 0.840104802 |
| PPT2 | blue | 0.343965809 | 0.023916048 |
| PRADC1 | blue | -0.151680013 | 0.331569883 |
| PRAMEF12 | lightcyan | -0.05994203 | 0.702589453 |
| PRAMEF17 | blue | -0.089535064 | 0.568020855 |
| PRAMEF20 | blue | -0.088450831 | 0.572735717 |
| PRAMEF4 | blue | -0.215656661 | 0.164866292 |
| PRAMEF5 | blue | -0.074849148 | 0.63334375 |
| PRB4 | blue | 0.081142131 | 0.604974787 |
| PRCD | blue | -0.065675624 | 0.675635881 |
| PRDM12 | blue | 0.056240922 | 0.72018409 |
| PRDM13 | blue | -0.112694849 | 0.471826345 |
| PRDM15 | blue | -0.256395956 | 0.096988733 |
| PRDM2 | blue | 0.023681986 | 0.880182269 |
| PRDX2 | blue | -0.339076488 | 0.026127738 |
| PRDX5 | blue | -0.327944059 | 0.031803612 |
| PREB | blue | -0.1052844 | 0.501632544 |
| PRELID2 | blue | 0.106227614 | 0.497786246 |
| PREPL | blue | -0.343978406 | 0.023910559 |
| PREX1 | blue | 0.407492442 | 0.006683927 |
| PREX2 | blue | 0.290242985 | 0.059021235 |
| PRG3 | blue | -0.016236921 | 0.917691626 |
| PRH1 | lightcyan | 0.322186196 | 0.035116952 |
| PRH2 | blue | 0.317301162 | 0.038145778 |
| PRIM2 | blue | -0.135828031 | 0.385135479 |
| PRKAA1 | blue | 0.231970788 | 0.134441979 |
| PRKAB1 | blue | -0.195159528 | 0.209792278 |
| PRKACG | blue | -0.068885515 | 0.66071666 |
| PRKAG3 | blue | -0.151436834 | 0.332354786 |
| PRKCG | blue | -0.175097337 | 0.261412366 |
| PRKCH | lightcyan | 0.608997052 | 1.47E-05 |
| PRKCQ | blue | -0.000699707 | 0.996446971 |
| PRKD2 | blue | 0.381663014 | 0.01155704 |
| PRKD3 | blue | 0.412729977 | 0.005950393 |
| PRKRA | blue | 0.340113286 | 0.025645049 |
| PRKX | blue | 0.377660049 | 0.012534187 |
| PRM2 | blue | -0.124720292 | 0.4255296 |
| PRMT5 | blue | -0.249058074 | 0.107277501 |
| PRMT7 | blue | 0.050545275 | 0.747538618 |
| PRND | blue | 0.071952778 | 0.646580551 |
| PROB1 | blue | -0.150018259 | 0.336956475 |
| PROCA1 | blue | 0.123766321 | 0.429106218 |
| PROCR | blue | 0.192608522 | 0.215928644 |
| PROK1 | blue | -0.085083019 | 0.587493736 |
| PROKR1 | blue | 0.019617996 | 0.900630459 |
| PROKR2 | blue | -0.108832476 | 0.487243007 |
| PROP1 | blue | -0.001075564 | 0.994538436 |
| PROX2 | blue | 0.155093044 | 0.320675708 |
| PRPF31 | blue | -0.496294871 | 0.000712452 |
| PRPF40A | lightcyan | 0.044061001 | 0.779055807 |
| PRPF40B | blue | 0.15631842 | 0.316819991 |
| PRPF4B | lightcyan | 0.554656928 | 0.000113555 |
| PRPS2 | blue | -0.351616635 | 0.020770001 |
| PRR11 | blue | 0.172048394 | 0.269938843 |
| PRR13 | blue | -0.324330118 | 0.033851564 |
| PRR14L | blue | 0.106410621 | 0.497041723 |
| PRR15L | blue | -0.332693069 | 0.029268971 |
| PRR16 | blue | -0.057783251 | 0.712834074 |
| PRRG1 | blue | -0.332531065 | 0.029352591 |
| PRRG2 | blue | -0.218308635 | 0.159607994 |
| PRRG4 | blue | -0.13198285 | 0.398855962 |
| PRRT1 | blue | 0.205869866 | 0.185357747 |
| PRSS22 | blue | -0.323504079 | 0.034334591 |
| PRSS36 | blue | -0.200649965 | 0.197000149 |
| PRSS38 | blue | 0.110302018 | 0.481346661 |
| PRSS48 | blue | 0.098513499 | 0.529681492 |
| PRSS51 | blue | 0.042935887 | 0.784561864 |
| PRSS53 | blue | 0.079698606 | 0.611433682 |
| PRSS54 | blue | -0.158139436 | 0.311144399 |
| PRSS57 | blue | 0.304475985 | 0.047126359 |
| PRUNE2 | blue | -0.25651934 | 0.096822474 |
| PSAPL1 | blue | -0.224206312 | 0.148353904 |
| PSD2 | blue | -0.276787007 | 0.072364639 |
| PSD4 | blue | 0.083035101 | 0.596549866 |
| PSG2 | blue | -0.179289493 | 0.249984556 |
| PSG3 | blue | 0.069845057 | 0.656281583 |
| PSG5 | blue | -0.401977916 | 0.007539572 |
| PSG8 | blue | -0.001443384 | 0.992670739 |
| PSG9 | blue | -0.15573859 | 0.318640797 |
| PSMB11 | blue | -0.297345477 | 0.0528165 |
| PSMB7 | blue | -0.294061471 | 0.055616784 |
| PSMB9 | lightyellow | 0.338191162 | 0.02654587 |
| PSMC4 | blue | -0.159168127 | 0.307966988 |
| PSMC5 | blue | -0.095500014 | 0.542407086 |
| PSMD10 | lightcyan | 0.199009507 | 0.2007632 |
| PSMD5 | blue | 0.197512859 | 0.204240165 |
| PSMD7 | lightcyan | 0.30850609 | 0.044136661 |
| PSMD9 | blue | -0.341306847 | 0.025098595 |
| PSMG1 | blue | -0.343413251 | 0.024157886 |
| PSMG2 | blue | -0.128551132 | 0.411336954 |
| PSMG4 | blue | -0.06532121 | 0.67729083 |
| PSTK | blue | 0.299058024 | 0.051401864 |
| PTCH2 | blue | -0.092967897 | 0.55321212 |
| PTCRA | lightyellow | -0.030646925 | 0.845323132 |
| PTEN | blue | 0.379700483 | 0.012027652 |
| PTF1A | blue | -0.17755609 | 0.25466837 |
| PTGDR | blue | 0.17727968 | 0.255420653 |
| PTGR2 | blue | -0.215352327 | 0.165477659 |
| PTGS1 | blue | 0.323913029 | 0.034094753 |
| PTHLH | blue | 0.238224407 | 0.123962445 |
| PTK2B | blue | -0.185031959 | 0.234883525 |
| PTP4A1 | blue | -0.186748577 | 0.230492724 |
| PTPDC1 | blue | 0.05634588 | 0.719683109 |
| PTPN4 | blue | -0.329759192 | 0.030814251 |
| PTPN7 | blue | 0.0500769 | 0.749802298 |
| PTPN9 | blue | 0.415354099 | 0.005609892 |
| PTPRB | blue | 0.318526617 | 0.037366582 |
| PTPRCAP | blue | -0.035024934 | 0.823555466 |
| PTPRN | blue | -0.06227217 | 0.691589757 |
| PTPRS | blue | -0.099664228 | 0.524860967 |
| PTTG2 | blue | 0.325458203 | 0.033200964 |
| PTX4 | blue | -0.256167673 | 0.097296924 |
| PURG | blue | 0.121990257 | 0.435809638 |
| PUS1 | blue | -0.101645247 | 0.516613254 |
| PUS10 | blue | -0.054499379 | 0.728513467 |
| PUS3 | blue | 0.02336306 | 0.881784362 |
| PUS7L | blue | -0.085176649 | 0.587081161 |
| PWP2 | blue | -0.151343847 | 0.332655219 |
| PXK | blue | -0.185271083 | 0.234268495 |
| PXN | blue | 0.264902372 | 0.086031588 |
| PYGM | blue | 0.201099369 | 0.195977995 |
| PZP | blue | 0.347770605 | 0.02230509 |
| QPCTL | blue | 0.28112435 | 0.067826489 |
| QRICH2 | blue | -0.174777977 | 0.262296963 |
| QRSL1 | blue | -0.062275273 | 0.69157515 |
| QSER1 | blue | -0.167961845 | 0.281652 |
| QTRT1 | blue | -0.230466717 | 0.137058023 |
| R3HCC1 | blue | -0.170971088 | 0.272994979 |
| R3HCC1L | blue | 0.252210063 | 0.102760483 |
| RAB11FIP4 | blue | -0.365281955 | 0.016013055 |
| RAB19 | blue | -0.29642045 | 0.053593542 |
| RAB22A | blue | -0.14848262 | 0.341982134 |
| RAB26 | blue | -0.124170704 | 0.427588059 |
| RAB28 | blue | 0.054500824 | 0.728506545 |
| RAB31 | blue | 0.232675737 | 0.133228726 |
| RAB32 | blue | 0.227401678 | 0.142505794 |
| RAB33A | blue | -0.337805951 | 0.026729531 |
| RAB34 | blue | 0.244569912 | 0.113968641 |
| RAB36 | blue | -0.493285382 | 0.000776468 |
| RAB38 | blue | -0.19123938 | 0.219272891 |
| RAB3A | blue | -0.245888175 | 0.111971336 |
| RAB3IL1 | blue | -0.242524079 | 0.117121608 |
| RAB42 | blue | 0.148500055 | 0.341924816 |
| RAB44 | blue | 0.00579246 | 0.970592885 |
| RAB5C | blue | -0.127662158 | 0.414606119 |
| RAB6B | blue | 0.017669711 | 0.910456753 |
| RAB9A | blue | -0.291714739 | 0.05768986 |
| RABEP2 | blue | 0.217507439 | 0.161183548 |
| RABGGTA | blue | 0.000769128 | 0.996094458 |
| RABGGTB | blue | -0.183036643 | 0.24005843 |
| RAC3 | blue | -0.351073145 | 0.020981392 |
| RACGAP1 | blue | 0.151631358 | 0.331726835 |
| RAD1 | blue | -0.16657308 | 0.285707024 |
| RAD18 | blue | 0.179409113 | 0.249663485 |
| RAD51AP2 | blue | 0.115637135 | 0.460258306 |
| RAD51B | blue | 0.249086357 | 0.107236307 |
| RAD51D | blue | 0.216029049 | 0.164120445 |
| RAD54B | blue | 0.328003233 | 0.03177095 |
| RAD54L | blue | -0.040830522 | 0.7948928 |
| RAET1L | blue | -0.016118038 | 0.91829223 |
| RALGPS2 | blue | -0.485627293 | 0.000963135 |
| RANBP1 | blue | 0.02082796 | 0.894535211 |
| RANBP10 | blue | -0.213695509 | 0.168834816 |
| RAP1A | blue | 0.415400819 | 0.005603986 |
| RAP1B | blue | 0.538803813 | 0.000193367 |
| RAP1GDS1 | blue | 0.241282678 | 0.119066694 |
| RAP2B | lightcyan | 0.193218882 | 0.214449242 |
| RAPGEF2 | blue | 0.151860081 | 0.330989429 |
| RAPGEF4 | blue | 0.247646036 | 0.109349493 |
| RAPGEF5 | lightcyan | 0.525837444 | 0.000293151 |
| RAPH1 | blue | 0.327210547 | 0.032210791 |
| RAPSN | blue | -0.113060537 | 0.470380246 |
| RARA | blue | -0.058998964 | 0.707058559 |
| RARB | blue | 0.103957295 | 0.507069839 |
| RARG | blue | 0.1853221 | 0.234137421 |
| RARS2 | blue | 0.233407581 | 0.131977837 |
| RASA3 | blue | 0.001845618 | 0.990628347 |
| RASA4B | blue | -0.098236183 | 0.530846431 |
| RASGEF1B | blue | 0.010396676 | 0.947243794 |
| RASIP1 | lightcyan | 0.38523111 | 0.010741643 |
| RASL10A | blue | -0.051486163 | 0.742997614 |
| RASL11A | blue | -0.345831098 | 0.023114546 |
| RASSF10 | blue | -0.294623186 | 0.055129543 |
| RAVER2 | blue | 0.455853112 | 0.002126268 |
| RB1 | blue | 0.173351743 | 0.266271777 |
| RBBP4 | blue | 0.248293341 | 0.108395903 |
| RBBP5 | blue | -0.16505925 | 0.290170283 |
| RBBP7 | blue | -0.073219529 | 0.640777762 |
| RBBP8NL | blue | 0.010867474 | 0.944858393 |
| RBFA | blue | -0.025452637 | 0.87129636 |
| RBFOX1 | blue | -0.006728922 | 0.965841263 |
| RBFOX3 | blue | -0.171234258 | 0.272246315 |
| RBL1 | blue | 0.114823062 | 0.463443579 |
| RBM12 | blue | -0.035785737 | 0.819785534 |
| RBM12B | blue | -0.075812804 | 0.628964389 |
| RBM14 | blue | 0.278476154 | 0.070569651 |
| RBM17 | blue | 0.220061794 | 0.156199576 |
| RBM34 | blue | 0.061008862 | 0.697545742 |
| RBM38 | blue | -0.137282029 | 0.380020577 |
| RBM7 | blue | 0.001386549 | 0.99295933 |
| RBMS3 | blue | 0.214210147 | 0.167786797 |
| RBMY1B | blue | 0.137084161 | 0.380714268 |
| RBMY1J | blue | 0.06519182 | 0.677895398 |
| RBP7 | blue | 0.318296604 | 0.037511831 |
| RBPJ | blue | 0.114048583 | 0.466484869 |
| RBPJL | blue | 0.045144613 | 0.773762918 |
| RBPMS | blue | 0.114661236 | 0.464078168 |
| RCAN2 | lightcyan | 0.127893917 | 0.413752412 |
| RCC1 | blue | -0.136132924 | 0.384059579 |
| RCC2 | blue | 0.359042429 | 0.01805617 |
| RCCD1 | blue | -0.325372827 | 0.03324984 |
| RCN3 | blue | 0.168922855 | 0.27886809 |
| RCOR2 | blue | 0.144062652 | 0.356703208 |
| RCSD1 | blue | 0.20319074 | 0.191270466 |
| RDH12 | blue | -0.065346006 | 0.677174997 |
| RDH14 | blue | -0.094203619 | 0.547926382 |
| REC8 | blue | 0.204677093 | 0.187973865 |
| RECQL | blue | 0.168147033 | 0.281114131 |
| REEP1 | blue | 0.056298986 | 0.719906924 |
| REEP2 | blue | 0.035815065 | 0.81964029 |
| REG1B | blue | 0.045169306 | 0.773642423 |
| REG4 | blue | -0.085444716 | 0.585900656 |
| RELB | blue | 0.248734835 | 0.107749151 |
| REM2 | blue | 0.019981642 | 0.898797966 |
| REN | blue | 0.447288994 | 0.002636317 |
| RENBP | blue | -0.158025238 | 0.311498412 |
| REP15 | blue | -0.003929387 | 0.980048972 |
| REPIN1 | blue | 0.013240213 | 0.93284406 |
| RESP18 | blue | 0.12172334 | 0.436822068 |
| RETN | blue | -0.154141638 | 0.323689634 |
| REV1 | blue | 0.36260743 | 0.016863497 |
| REXO1 | blue | 0.24965512 | 0.106410471 |
| REXO2 | blue | -0.228053158 | 0.141334676 |
| REXO4 | blue | -0.120453747 | 0.441655507 |
| RFC5 | blue | -0.003227519 | 0.98361209 |
| RFK | blue | -0.089443338 | 0.568419043 |
| RFNG | blue | -0.206051945 | 0.184960686 |
| RFPL2 | blue | -0.046814953 | 0.765624048 |
| RFTN2 | blue | -0.003126178 | 0.984126588 |
| RFWD3 | blue | 0.066859557 | 0.670118424 |
| RFX2 | blue | 0.072587615 | 0.643669845 |
| RFX4 | blue | 0.005236118 | 0.973416258 |
| RFX5 | blue | 0.310737334 | 0.042548897 |
| RFX8 | blue | -0.029426202 | 0.851413681 |
| RGL1 | blue | 0.109469089 | 0.48468408 |
| RGMB | blue | 0.069788821 | 0.656541189 |
| RGS14 | blue | -0.105792217 | 0.499559855 |
| RGS16 | blue | 0.198736168 | 0.201395087 |
| RGS17 | blue | 0.251937204 | 0.103145653 |
| RGS22 | blue | 0.019069279 | 0.90339653 |
| RGS9BP | blue | -0.202703937 | 0.192359004 |
| RHBDF2 | blue | 0.120322082 | 0.442158446 |
| RHBDL1 | blue | -0.02860449 | 0.855518279 |
| RHEBL1 | blue | -0.012566641 | 0.936253305 |
| RHO | blue | -0.308107282 | 0.044425462 |
| RHOT2 | blue | 0.010270218 | 0.947884595 |
| RHOV | blue | -0.608502576 | 1.50E-05 |
| RHOXF1 | blue | -0.382149442 | 0.011442858 |
| RIC8B | blue | 0.020399021 | 0.896695343 |
| RILP | blue | -0.224270184 | 0.148235304 |
| RILPL1 | blue | 0.289448853 | 0.059749755 |
| RIMBP2 | blue | -0.216789591 | 0.162604778 |
| RIMBP3 | blue | -0.169031013 | 0.278555904 |
| RIN1 | blue | 0.007369259 | 0.962592845 |
| RIN3 | lightcyan | 0.278583669 | 0.0704566 |
| RIOK1 | blue | 0.038795515 | 0.804911535 |
| RIOK3 | blue | 0.116860997 | 0.455491906 |
| RIPK1 | blue | 0.349840911 | 0.021467364 |
| RIPK2 | blue | 0.212750441 | 0.170771672 |
| RIPPLY2 | blue | -0.102000479 | 0.515141162 |
| RIPPLY3 | blue | -0.152953057 | 0.327479775 |
| RIT1 | blue | 0.103665643 | 0.508268751 |
| RLBP1 | blue | -0.091241297 | 0.560637618 |
| RLF | blue | 0.288305906 | 0.060810856 |
| RLN2 | blue | -0.108580774 | 0.488256683 |
| RMDN1 | blue | -0.12567448 | 0.421968994 |
| RMI2 | blue | -0.006259886 | 0.968221023 |
| RNASE1 | lightcyan | 0.285440666 | 0.063537059 |
| RNASE10 | blue | 0.253705331 | 0.100669276 |
| RNASE12 | blue | 0.202066742 | 0.193790448 |
| RNASE7 | blue | -0.231635244 | 0.135022347 |
| RNASEH1 | blue | 0.231459516 | 0.135327035 |
| RNASET2 | blue | 0.417289356 | 0.00536974 |
| RND1 | blue | -0.074170965 | 0.636433218 |
| RND3 | blue | -0.07279641 | 0.642713673 |
| RNF10 | blue | -0.422490782 | 0.004768033 |
| RNF112 | blue | 0.09447238 | 0.54677996 |
| RNF121 | blue | -0.01409645 | 0.928511995 |
| RNF123 | blue | -0.069109664 | 0.659679593 |
| RNF125 | blue | 0.402799588 | 0.007406378 |
| RNF126 | blue | -0.259593614 | 0.092750334 |
| RNF130 | blue | 0.103354537 | 0.509549211 |
| RNF135 | blue | -0.115098992 | 0.462362608 |
| RNF139 | blue | -0.157328948 | 0.313662436 |
| RNF144A | blue | 0.225361799 | 0.146219123 |
| RNF144B | blue | 0.195922324 | 0.207981244 |
| RNF148 | blue | 0.227459841 | 0.14240095 |
| RNF157 | blue | -0.084038537 | 0.592104906 |
| RNF165 | lightcyan | 0.401013031 | 0.007698616 |
| RNF168 | blue | -0.04209276 | 0.788694774 |
| RNF169 | blue | -0.145319981 | 0.352476982 |
| RNF170 | blue | -0.407554335 | 0.006674821 |
| RNF175 | blue | 0.094132878 | 0.548228326 |
| RNF19A | blue | 0.296418762 | 0.053594968 |
| RNF207 | blue | -0.078768542 | 0.615610665 |
| RNF208 | blue | -0.119544342 | 0.445135727 |
| RNF223 | blue | 0.127252486 | 0.416117635 |
| RNF32 | blue | -0.219700851 | 0.156896923 |
| RNF34 | blue | 0.247618259 | 0.109390556 |
| RNF39 | blue | -0.411900267 | 0.006061719 |
| RNF5 | blue | -0.336411912 | 0.027403035 |
| RNF8 | blue | 0.003186806 | 0.983818787 |
| RNFT1 | blue | 0.149860962 | 0.337469146 |
| RNPEP | blue | -0.15711548 | 0.314327785 |
| ROBO3 | blue | 0.25607317 | 0.097424727 |
| ROCK2 | blue | 0.071468419 | 0.648804831 |
| ROPN1 | blue | -0.111390815 | 0.477002248 |
| ROPN1B | blue | -0.147175439 | 0.346296297 |
| ROR2 | blue | -0.070723427 | 0.652231879 |
| RORB | blue | -0.14429915 | 0.355905935 |
| RPA1 | blue | 0.182056773 | 0.242627863 |
| RPA4 | blue | 0.238958993 | 0.12277293 |
| RPAP3 | blue | 0.130219904 | 0.405240064 |
| RPE | blue | 0.150582722 | 0.33512072 |
| RPF1 | blue | 0.139387938 | 0.372684068 |
| RPGR | blue | 0.278468531 | 0.070577671 |
| RPH3A | blue | -0.093874719 | 0.549330885 |
| RPH3AL | blue | 0.008886994 | 0.954895894 |
| RPL27 | blue | -0.101438107 | 0.517472618 |
| RPL37A | blue | -0.273455016 | 0.076010793 |
| RPL39L | blue | 0.282522408 | 0.066412742 |
| RPL4 | blue | -0.355943809 | 0.019149843 |
| RPL6 | blue | -0.348640891 | 0.021949664 |
| RPL7 | blue | -0.254041591 | 0.10020353 |
| RPLP1 | blue | -0.275131222 | 0.074158916 |
| RPP38 | blue | 0.084265891 | 0.591099822 |
| RPRM | blue | -0.195313086 | 0.209426818 |
| RPRML | blue | -0.2734628 | 0.07600211 |
| RPS20 | blue | -0.077271009 | 0.62236141 |
| RPS6KC1 | blue | 0.160634225 | 0.303474378 |
| RPSA | blue | -0.125876191 | 0.421218454 |
| RPUSD1 | blue | -0.070080392 | 0.655195617 |
| RPUSD2 | blue | -0.142992558 | 0.360324191 |
| RPUSD4 | blue | 0.063975296 | 0.683589257 |
| RRP1B | blue | 0.082967967 | 0.59684777 |
| RRP7A | blue | -0.252674247 | 0.102107769 |
| RRP8 | blue | 0.02530214 | 0.872051027 |
| RRS1 | blue | -0.056134188 | 0.720693664 |
| RSAD1 | blue | -0.056237462 | 0.720200605 |
| RSAD2 | lightyellow | 0.216800375 | 0.162583361 |
| RSPH6A | blue | 0.330792239 | 0.030262628 |
| RSRC2 | blue | 0.153620893 | 0.325346781 |
| RTCA | blue | 0.208123852 | 0.180485065 |
| RTEL1 | blue | -0.126778992 | 0.417868522 |
| RTL1 | blue | -0.133438779 | 0.393627887 |
| RTN1 | blue | 0.050674996 | 0.746912043 |
| RTN4RL1 | lightcyan | -0.154612932 | 0.322194424 |
| RTN4RL2 | blue | -0.077076814 | 0.623239083 |
| RTP2 | blue | -0.107507934 | 0.492589562 |
| RUNDC1 | blue | 0.192515253 | 0.216155331 |
| RUNDC3A | lightcyan | -0.265417722 | 0.085400075 |
| RUNX1T1 | blue | 0.234445793 | 0.130218354 |
| RUSC1 | blue | 0.048300276 | 0.758407525 |
| RUVBL2 | blue | -0.182755061 | 0.2407949 |
| RWDD2A | blue | 0.172044736 | 0.269949181 |
| RXFP1 | blue | 0.446418453 | 0.002693747 |
| RXRA | blue | -0.050215367 | 0.749132862 |
| RXRG | blue | -0.12574131 | 0.421720242 |
| S100A10 | blue | 0.104198729 | 0.506078443 |
| S100A7L2 | blue | 0.179806458 | 0.24859896 |
| S100PBP | blue | 0.190124639 | 0.222022055 |
| S100Z | blue | 0.184384995 | 0.236553043 |
| S1PR1 | lightcyan | 0.338366364 | 0.026462685 |
| S1PR5 | blue | 0.22494 | 0.146995763 |
| SAA4 | blue | -0.08534121 | 0.58635635 |
| SAFB2 | blue | 0.115072251 | 0.462467306 |
| SALL3 | blue | -0.315056204 | 0.039607705 |
| SALL4 | blue | 0.104843851 | 0.503434218 |
| SAMD13 | lightcyan | -0.105911414 | 0.49907398 |
| SAMD3 | blue | 0.248373748 | 0.108277893 |
| SAMD4B | blue | 0.085437865 | 0.585930815 |
| SAMD8 | blue | 0.179497381 | 0.249426741 |
| SAMD9 | lightyellow | 0.202110898 | 0.19369101 |
| SAMD9L | lightyellow | 0.190473241 | 0.221159795 |
| SAMHD1 | blue | 0.551560958 | 0.000126261 |
| SAPCD2 | blue | -0.055388277 | 0.724258155 |
| SAR1B | blue | 0.133417524 | 0.393703922 |
| SAT1 | blue | -0.010742837 | 0.945489849 |
| SATB1 | blue | 0.2920445 | 0.057394873 |
| SATL1 | blue | -0.421961359 | 0.004826466 |
| SBNO2 | blue | 0.153229067 | 0.326597174 |
| SCAF4 | blue | 0.316977201 | 0.038353974 |
| SCAF8 | blue | -0.07081661 | 0.651802839 |
| SCAI | blue | 0.261157207 | 0.090730664 |
| SCARF1 | blue | 0.175085928 | 0.261443933 |
| SCEL | blue | -0.028221718 | 0.857431585 |
| SCG2 | blue | -0.111598794 | 0.476174747 |
| SCGB1D1 | blue | 0.013196302 | 0.933066279 |
| SCGB3A2 | blue | 0.240898973 | 0.119672806 |
| SCLY | blue | 0.037187276 | 0.812851179 |
| SCML4 | blue | 0.190372567 | 0.221408573 |
| SCN4B | blue | 0.047469147 | 0.762443159 |
| SCN5A | blue | -0.1596381 | 0.306522243 |
| SCN7A | blue | -0.145974427 | 0.350289339 |
| SCN9A | blue | 0.068938106 | 0.660473281 |
| SCO1 | blue | 0.10185325 | 0.515751029 |
| SCRG1 | blue | 0.30046222 | 0.050264861 |
| SCTR | blue | -0.056266306 | 0.720062918 |
| SCUBE1 | blue | 0.187591956 | 0.228356226 |
| SCYL2 | blue | 0.133470376 | 0.393514872 |
| SDCBP2 | blue | -0.064870139 | 0.679399299 |
| SDF4 | blue | 0.036494458 | 0.816277282 |
| SDHAF1 | blue | -0.125662605 | 0.422013202 |
| SDHD | blue | -0.346073249 | 0.023012161 |
| SDR39U1 | blue | 0.165592154 | 0.288593987 |
| SEC13 | blue | -0.026231081 | 0.867394659 |
| SEC14L6 | blue | 0.223144784 | 0.150335214 |
| SEC16A | blue | 0.00187327 | 0.990487942 |
| SEC23B | blue | -0.062354622 | 0.691201664 |
| SEC23IP | lightcyan | 0.36109661 | 0.017360554 |
| SEMA3A | blue | 0.022398914 | 0.886630449 |
| SEMA3F | blue | 0.336091688 | 0.027559719 |
| SEMA4D | blue | -0.229932558 | 0.13799613 |
| SEMA6B | blue | 0.059298171 | 0.705639583 |
| SENP1 | blue | 0.102024302 | 0.515042514 |
| SENP5 | blue | 0.39164555 | 0.009399227 |
| SEPHS1 | blue | -0.459264975 | 0.001948706 |
| SERP2 | blue | -0.140581639 | 0.368563276 |
| SERPINA7 | blue | -0.031626234 | 0.840443481 |
| SERPINA9 | blue | -0.252032055 | 0.103011635 |
| SERPINB13 | blue | -0.153361286 | 0.326174901 |
| SERPINB5 | blue | 0.059006769 | 0.707021533 |
| SERPINB9 | blue | 0.113922657 | 0.46698037 |
| SERPINE1 | blue | 0.217317076 | 0.161559555 |
| SERPINH1 | blue | 0.170125123 | 0.275410752 |
| SETD1A | blue | 0.112984113 | 0.470682266 |
| SEZ6 | blue | 0.140635994 | 0.368376289 |
| SF1 | blue | 0.384103742 | 0.010993779 |
| SFR1 | blue | 0.081815053 | 0.601973972 |
| SFRP5 | blue | 0.154225976 | 0.323421747 |
| SFTPC | blue | 0.052823855 | 0.736556343 |
| SFXN3 | blue | 0.209018511 | 0.17857661 |
| SGCA | blue | 0.210274555 | 0.175921717 |
| SGCG | blue | 0.082740967 | 0.597855568 |
| SGCZ | lightcyan | -0.279444919 | 0.069556161 |
| SGIP1 | blue | -0.024769906 | 0.874720812 |
| SGK1 | blue | 0.279746091 | 0.06924344 |
| SGMS2 | blue | -0.082688031 | 0.598090693 |
| SGPL1 | blue | -0.107271239 | 0.493548166 |
| SH2B3 | blue | 0.51760922 | 0.000378486 |
| SH2D3C | blue | 0.422632942 | 0.004752449 |
| SH3BGR | blue | -0.236294688 | 0.127128471 |
| SH3BP1 | blue | 0.386298851 | 0.010507422 |
| SH3BP4 | blue | -0.202043366 | 0.193843105 |
| SH3D19 | blue | 0.101942818 | 0.515379968 |
| SH3D21 | blue | 0.206797408 | 0.183341363 |
| SH3GL1 | blue | 0.154705688 | 0.321900662 |
| SH3RF3 | blue | 0.446609532 | 0.002681048 |
| SHANK3 | blue | 0.398350579 | 0.008152571 |
| SHC1 | blue | 0.287001219 | 0.062040428 |
| SHC4 | blue | 0.304844635 | 0.046846241 |
| SHCBP1L | blue | -0.280683871 | 0.068276815 |
| SHE | blue | 0.345137896 | 0.023409748 |
| SHF | blue | 0.411374799 | 0.006133154 |
| SHISA3 | blue | -0.039248117 | 0.802680561 |
| SHISA5 | lightyellow | 0.324588647 | 0.033701543 |
| SHISA7 | blue | 0.079417999 | 0.612692635 |
| SHISA8 | blue | -0.337343454 | 0.026951436 |
| SHISA9 | blue | -0.045111437 | 0.773924818 |
| SHOX | blue | 0.148615443 | 0.34154563 |
| SHROOM1 | blue | 0.158738383 | 0.309291852 |
| SHROOM2 | blue | 0.062289463 | 0.691508352 |
| SHROOM4 | blue | 0.386357675 | 0.010494646 |
| SIAE | blue | -0.301035349 | 0.049806658 |
| SIGLEC11 | blue | 0.230247633 | 0.137442212 |
| SIGLEC12 | blue | 0.311779194 | 0.041823599 |
| SIGLEC15 | blue | -0.027977828 | 0.858651102 |
| SIGLEC6 | blue | 0.06157216 | 0.69488778 |
| SIGLEC7 | blue | 0.180589962 | 0.246508847 |
| SIK2 | blue | -0.036317516 | 0.817152838 |
| SIKE1 | blue | 0.083154618 | 0.596019668 |
| SIN3B | blue | 0.423136643 | 0.004697585 |
| SIPA1L3 | blue | -0.311018436 | 0.042352205 |
| SIRPB2 | blue | 0.048749104 | 0.75623082 |
| SIRPG | blue | 0.009033662 | 0.954152291 |
| SIRT1 | blue | -0.08763599 | 0.576290839 |
| SIRT3 | blue | 0.206422197 | 0.184155141 |
| SIRT4 | blue | -0.0527342 | 0.736987502 |
| SIRT6 | blue | -0.467898264 | 0.001556582 |
| SIT1 | blue | 0.311849395 | 0.041775093 |
| SIX1 | blue | -0.117357992 | 0.453563998 |
| SIX3 | blue | -0.367068035 | 0.015465568 |
| SKA1 | blue | 0.241885085 | 0.118119792 |
| SKI | blue | 0.104939229 | 0.50304388 |
| SKOR1 | blue | -0.141890389 | 0.364076839 |
| SLAMF8 | blue | 0.049372949 | 0.753208424 |
| SLAMF9 | blue | 0.177340487 | 0.255255033 |
| SLC10A1 | blue | 0.192996078 | 0.214988461 |
| SLC10A6 | blue | 0.262719974 | 0.088746257 |
| SLC12A4 | blue | -0.034929791 | 0.824027198 |
| SLC12A5 | blue | -0.032515249 | 0.83601884 |
| SLC12A8 | blue | -0.288813737 | 0.06033755 |
| SLC13A4 | blue | -0.023477239 | 0.881210739 |
| SLC15A3 | lightyellow | 0.353540958 | 0.020035842 |
| SLC16A5 | lightyellow | -0.248988944 | 0.107378239 |
| SLC16A6 | blue | 0.027158606 | 0.862749808 |
| SLC17A2 | blue | 0.228567247 | 0.140415574 |
| SLC17A6 | blue | -0.163801842 | 0.293911693 |
| SLC17A8 | blue | 0.083459593 | 0.594667691 |
| SLC18A1 | blue | 0.055147169 | 0.725411579 |
| SLC18A2 | blue | -0.338012888 | 0.026630737 |
| SLC18A3 | blue | -0.13659603 | 0.382428769 |
| SLC19A2 | blue | -0.296226594 | 0.053757543 |
| SLC20A1 | blue | 0.207532878 | 0.181753683 |
| SLC22A23 | blue | 0.084818012 | 0.588662172 |
| SLC24A1 | blue | -0.073416046 | 0.639879426 |
| SLC24A5 | blue | -0.288941706 | 0.060218745 |
| SLC25A1 | blue | -0.354399483 | 0.019715406 |
| SLC25A16 | blue | -0.280989252 | 0.067964357 |
| SLC25A17 | blue | -0.063551788 | 0.685575552 |
| SLC25A21 | blue | -0.163799132 | 0.29391979 |
| SLC25A23 | blue | -0.503068981 | 0.000585305 |
| SLC25A29 | blue | -0.054844373 | 0.726860955 |
| SLC25A31 | blue | -0.146141885 | 0.349730905 |
| SLC25A33 | blue | -0.341449235 | 0.025034057 |
| SLC25A34 | blue | -0.370564597 | 0.014439581 |
| SLC25A38 | blue | -0.204227842 | 0.188965978 |
| SLC25A41 | blue | 0.127061071 | 0.416824946 |
| SLC25A45 | blue | 0.02770804 | 0.860000496 |
| SLC25A46 | blue | -0.096575961 | 0.537846695 |
| SLC25A51 | blue | 0.110435758 | 0.480811913 |
| SLC25A6 | blue | -0.360153799 | 0.01767696 |
| SLC26A11 | blue | -0.193596777 | 0.213536829 |
| SLC26A4 | blue | -0.425660216 | 0.004430941 |
| SLC26A6 | blue | 0.034851341 | 0.824416208 |
| SLC26A9 | blue | -0.250799279 | 0.104763883 |
| SLC27A4 | blue | -0.224425365 | 0.147947449 |
| SLC27A6 | blue | -0.287500367 | 0.061567699 |
| SLC28A2 | blue | -0.130605063 | 0.403840305 |
| SLC28A3 | blue | 0.048613977 | 0.756885962 |
| SLC29A2 | blue | -0.130298008 | 0.404955992 |
| SLC2A11 | blue | 0.029415692 | 0.851466154 |
| SLC2A4 | blue | 0.202620078 | 0.192546963 |
| SLC2A8 | blue | -0.173662785 | 0.26540154 |
| SLC2A9 | blue | 0.029861598 | 0.849240364 |
| SLC30A4 | blue | -0.174245553 | 0.263776148 |
| SLC33A1 | blue | 0.035446011 | 0.821468458 |
| SLC35A3 | blue | -0.30951223 | 0.043414828 |
| SLC35B1 | blue | 0.005159711 | 0.973804036 |
| SLC35B2 | blue | -0.186051349 | 0.232269284 |
| SLC35B4 | blue | -0.149655945 | 0.338138073 |
| SLC35C1 | blue | 0.187780018 | 0.227881674 |
| SLC35D1 | blue | 0.050268168 | 0.74887764 |
| SLC35D2 | blue | -0.079248702 | 0.613452723 |
| SLC35E2B | blue | -0.042384351 | 0.787264773 |
| SLC35F3 | blue | -0.042601321 | 0.786201172 |
| SLC35F5 | blue | 0.198660771 | 0.201569629 |
| SLC35G1 | blue | -0.026907199 | 0.864008358 |
| SLC37A1 | blue | -0.111715655 | 0.475710118 |
| SLC37A3 | blue | 0.073624288 | 0.638928051 |
| SLC38A2 | blue | 0.093241291 | 0.552040624 |
| SLC38A4 | blue | -0.056025566 | 0.721212378 |
| SLC38A5 | blue | 0.089560609 | 0.567909988 |
| SLC39A11 | blue | -0.160643117 | 0.303447259 |
| SLC40A1 | blue | -0.114958373 | 0.462913321 |
| SLC41A3 | blue | 0.209839829 | 0.176837368 |
| SLC43A1 | blue | -0.298021067 | 0.052254739 |
| SLC45A4 | blue | -0.047480892 | 0.762386086 |
| SLC4A10 | blue | -0.174867914 | 0.262047645 |
| SLC4A3 | blue | -0.117909201 | 0.451430981 |
| SLC51A | blue | -0.003749396 | 0.980962682 |
| SLC5A1 | blue | -0.216526337 | 0.163128257 |
| SLC5A6 | blue | -0.106546108 | 0.496490893 |
| SLC5A7 | blue | 0.006606686 | 0.966461427 |
| SLC5A8 | blue | -0.081533041 | 0.603230784 |
| SLC6A1 | blue | -0.213518008 | 0.169197378 |
| SLC6A17 | lightcyan | -0.362881909 | 0.016774497 |
| SLC6A18 | blue | -0.018057951 | 0.908497544 |
| SLC6A3 | blue | -0.173032997 | 0.267165527 |
| SLC6A7 | blue | 0.030074238 | 0.848179353 |
| SLC6A9 | blue | 0.166202752 | 0.28679472 |
| SLC7A10 | blue | -0.388193554 | 0.010102554 |
| SLC7A13 | blue | -0.0168818 | 0.914434464 |
| SLC7A4 | blue | -0.009011573 | 0.954264279 |
| SLC7A6OS | lightcyan | 0.365257521 | 0.016020657 |
| SLC8A2 | blue | -0.133564786 | 0.393177296 |
| SLC9A9 | lightyellow | 0.026014215 | 0.868481319 |
| SLC9C2 | blue | 0.13736617 | 0.379725818 |
| SLCO1A2 | blue | 0.340820416 | 0.025320117 |
| SLCO3A1 | blue | 0.189175699 | 0.224380978 |
| SLCO4A1 | blue | 0.300350093 | 0.050354899 |
| SLCO5A1 | blue | 0.261917209 | 0.089761362 |
| SLCO6A1 | blue | 0.15359657 | 0.325424314 |
| SLFN5 | blue | 0.262127374 | 0.089494741 |
| SLIRP | blue | 0.046976092 | 0.764840186 |
| SLIT1 | blue | -0.026061165 | 0.868246046 |
| SLITRK1 | blue | -0.325908993 | 0.032943878 |
| SLITRK5 | blue | -0.368893654 | 0.014922434 |
| SLURP1 | blue | 0.201844642 | 0.194291155 |
| SLX4 | blue | 0.287454339 | 0.06161117 |
| SMAD4 | blue | 0.073239105 | 0.640688253 |
| SMAD9 | blue | 0.038370116 | 0.807009819 |
| SMARCAD1 | blue | -0.147427326 | 0.345462395 |
| SMARCC1 | blue | -0.294061415 | 0.055616833 |
| SMC2 | blue | -0.350565211 | 0.021180583 |
| SMC4 | blue | 0.25183414 | 0.103291426 |
| SMCP | blue | 0.157293933 | 0.313771513 |
| SMCR8 | blue | -0.304265055 | 0.047287243 |
| SMDT1 | blue | -0.360215155 | 0.017656223 |
| SMG5 | blue | -0.170765228 | 0.273581554 |
| SMG6 | blue | -0.091721942 | 0.558565874 |
| SMG9 | lightcyan | 0.241342496 | 0.118972413 |
| SMIM1 | blue | -0.263429745 | 0.087856184 |
| SMIM12 | blue | 0.13295243 | 0.395369849 |
| SMIM19 | blue | -0.273823693 | 0.075600394 |
| SMIM20 | blue | -0.280620507 | 0.068341789 |
| SMIM4 | blue | -0.3500365 | 0.021389603 |
| SMIM5 | blue | -0.488925352 | 0.000878332 |
| SMIM8 | blue | -0.171405022 | 0.271761252 |
| SMKR1 | blue | 0.172443082 | 0.268824858 |
| SMLR1 | blue | -0.307368335 | 0.044964636 |
| SMOX | blue | 0.122762443 | 0.432888049 |
| SMPD2 | blue | 0.091355786 | 0.560143806 |
| SMPX | blue | -0.065622072 | 0.675885849 |
| SMR3A | blue | -0.226388546 | 0.144341255 |
| SMS | blue | -0.277817679 | 0.071265157 |
| SMTN | blue | 0.109875916 | 0.483052479 |
| SMTNL1 | blue | 0.274619424 | 0.074720547 |
| SMUG1 | blue | 0.074246834 | 0.63608729 |
| SMURF1 | blue | 0.250827525 | 0.10472348 |
| SMYD2 | blue | -0.267958959 | 0.082338631 |
| SNAI2 | blue | -0.395837851 | 0.008601965 |
| SNAP25 | blue | 0.242767585 | 0.116742905 |
| SNAP47 | blue | 0.012611615 | 0.936025636 |
| SNAP91 | blue | -0.196390875 | 0.206874235 |
| SND1 | blue | 0.1577304 | 0.312413588 |
| SNIP1 | blue | 0.154992304 | 0.320993999 |
| SNPH | blue | 0.121251965 | 0.438613196 |
| SNRNP25 | blue | -0.337818406 | 0.026723577 |
| SNRNP40 | blue | -0.024772685 | 0.874706869 |
| SNRNP48 | blue | -0.228904254 | 0.139815471 |
| SNRPB2 | blue | -0.452711047 | 0.002302265 |
| SNTG1 | blue | -0.173424489 | 0.266068079 |
| SNUPN | blue | -0.114014135 | 0.466620388 |
| SNX13 | blue | 0.129280489 | 0.408665813 |
| SNX18 | blue | 0.245089664 | 0.113177962 |
| SNX2 | blue | -0.269357123 | 0.080691175 |
| SNX20 | blue | 0.207886167 | 0.180994528 |
| SNX27 | blue | -0.292258736 | 0.057203877 |
| SNX7 | blue | -0.201047527 | 0.196095716 |
| SOCS7 | blue | -0.011927858 | 0.939487504 |
| SOGA3 | blue | 0.148173224 | 0.343000247 |
| SOHLH2 | lightcyan | -0.315121544 | 0.039564518 |
| SORBS1 | blue | 0.521815714 | 0.000332413 |
| SORCS2 | blue | -0.206453375 | 0.184087422 |
| SOS1 | blue | 0.286807407 | 0.062224759 |
| SOSTDC1 | blue | -0.002914944 | 0.985199023 |
| SOWAHA | blue | -0.499813896 | 0.000643614 |
| SOX12 | blue | 0.136169046 | 0.38393223 |
| SOX13 | blue | 0.475081906 | 0.001285387 |
| SOX14 | blue | -0.136054897 | 0.384334752 |
| SOX17 | blue | 0.289795311 | 0.059431044 |
| SOX5 | blue | 0.417595705 | 0.005332555 |
| SOX6 | blue | -0.471955275 | 0.001397783 |
| SOX9 | blue | -0.140475324 | 0.368929177 |
| SP110 | lightyellow | 0.371914085 | 0.014059362 |
| SP140 | blue | 0.341373744 | 0.025068256 |
| SP140L | lightcyan | 0.286623068 | 0.062400485 |
| SP3 | blue | 0.167321817 | 0.283516109 |
| SP5 | blue | -0.179489655 | 0.249447456 |
| SP6 | lightcyan | -0.255887965 | 0.097675568 |
| SPA17 | blue | -0.199744865 | 0.199070162 |
| SPACA3 | blue | -0.049312222 | 0.753502474 |
| SPACA5B | blue | 0.09947406 | 0.525656107 |
| SPACA7 | blue | -0.231474318 | 0.13530135 |
| SPAG1 | blue | 0.10735623 | 0.493203847 |
| SPAG11A | blue | -0.100423846 | 0.52169074 |
| SPAG16 | blue | -0.184443486 | 0.236401771 |
| SPAG4 | lightcyan | -0.235019996 | 0.129252796 |
| SPAG8 | blue | -0.001837696 | 0.990668568 |
| SPANXA1 | blue | -0.129885285 | 0.406458413 |
| SPANXB1 | blue | 0.062296071 | 0.69147725 |
| SPANXN1 | blue | -0.188863905 | 0.225159801 |
| SPATA16 | blue | -0.236645685 | 0.126548141 |
| SPATA20 | blue | -0.114220312 | 0.465809588 |
| SPATA24 | blue | -0.293460031 | 0.056142306 |
| SPATA3 | blue | -0.084668604 | 0.589321377 |
| SPATA4 | blue | -0.065929087 | 0.674453246 |
| SPATA5L1 | blue | 0.046951575 | 0.764959433 |
| SPATA7 | blue | 0.058651114 | 0.708709456 |
| SPATC1 | blue | -0.091019564 | 0.561594569 |
| SPATS2 | blue | 0.464720402 | 0.00169193 |
| SPATS2L | lightcyan | -0.532847176 | 0.000234588 |
| SPC24 | blue | 0.133026257 | 0.395105134 |
| SPCS3 | blue | 0.277960712 | 0.071113623 |
| SPDEF | blue | 0.124242113 | 0.427320286 |
| SPEF2 | blue | -0.125296036 | 0.423379163 |
| SPEG | blue | -0.050538458 | 0.747571552 |
| SPESP1 | blue | -0.268988952 | 0.081122469 |
| SPG21 | blue | -0.12491251 | 0.424810972 |
| SPHK2 | blue | -0.159841077 | 0.305899612 |
| SPHKAP | blue | 0.234104622 | 0.130794599 |
| SPIB | blue | 0.020231606 | 0.897538648 |
| SPIN1 | lightcyan | -0.28859939 | 0.060536965 |
| SPINK13 | blue | -0.000128841 | 0.999345757 |
| SPINK14 | blue | -0.167425214 | 0.283214418 |
| SPINK2 | blue | -0.154870747 | 0.321378327 |
| SPINT4 | blue | -0.005492745 | 0.972113862 |
| SPN | blue | 0.057225897 | 0.715487242 |
| SPOCK3 | blue | 0.078458861 | 0.617004134 |
| SPON2 | blue | 0.027714638 | 0.859967493 |
| SPPL2B | blue | 0.024009036 | 0.878539861 |
| SPR | blue | -0.068642781 | 0.661840424 |
| SPRED3 | blue | 0.228701422 | 0.140176423 |
| SPRN | blue | 0.225821045 | 0.145376973 |
| SPRR1B | blue | 0.033936439 | 0.828955981 |
| SPRR2A | blue | -0.115021884 | 0.462664543 |
| SPRR2B | blue | -0.158298152 | 0.310652805 |
| SPRR2E | blue | -0.094701075 | 0.545805347 |
| SPRR2G | lightcyan | -0.183226911 | 0.239561654 |
| SPRR3 | blue | -0.012609799 | 0.936034829 |
| SPRTN | blue | 0.360241383 | 0.017647364 |
| SPRY2 | blue | 0.322656867 | 0.034835873 |
| SPRYD4 | blue | -0.25907807 | 0.093423817 |
| SPSB2 | blue | -0.038728171 | 0.805243623 |
| SPTBN1 | blue | 0.406704667 | 0.006800763 |
| SPTBN4 | blue | -0.115179574 | 0.462047179 |
| SPTSSB | blue | 0.020927484 | 0.894034117 |
| SPZ1 | lightcyan | -0.181752866 | 0.24342854 |
| SQLE | blue | 0.267312373 | 0.083109326 |
| SRA1 | blue | -0.097019967 | 0.535970197 |
| SRD5A2 | blue | 0.089662316 | 0.567468669 |
| SREBF1 | blue | -0.126233167 | 0.419892051 |
| SREBF2 | blue | 0.043077334 | 0.783869077 |
| SRMS | blue | -0.233038945 | 0.132606821 |
| SRPK3 | blue | -0.068622673 | 0.661933551 |
| SRRM1 | blue | -0.049909105 | 0.750613764 |
| SRRM2 | blue | 0.354853413 | 0.019547729 |
| SRRM4 | blue | -0.132245587 | 0.397909541 |
| SRRM5 | blue | -0.014536951 | 0.926284097 |
| SRSF1 | blue | 0.047558856 | 0.762007263 |
| SRSF10 | blue | 0.197748133 | 0.203690808 |
| SS18L1 | blue | -0.199502426 | 0.199627224 |
| SS18L2 | blue | -0.262719826 | 0.088746444 |
| SSBP2 | blue | 0.023447434 | 0.881360472 |
| SST | blue | -0.193175712 | 0.214553648 |
| SSTR3 | blue | 0.088082725 | 0.574340509 |
| SSX1 | blue | -0.079643089 | 0.611682674 |
| SSX2B | blue | -0.20108758 | 0.19600476 |
| SSX7 | blue | 0.047363342 | 0.762957354 |
| ST13 | blue | -0.303195462 | 0.048109917 |
| ST3GAL2 | blue | 0.35780231 | 0.018487382 |
| ST3GAL5 | blue | 0.27662733 | 0.072536167 |
| ST6GALNAC2 | blue | -0.249572385 | 0.106530299 |
| ST7L | blue | 0.092375777 | 0.555753382 |
| ST8SIA2 | lightcyan | -0.314660465 | 0.039870097 |
| ST8SIA5 | blue | -0.175899154 | 0.259200166 |
| ST8SIA6 | blue | 0.374972018 | 0.013229151 |
| STAB1 | blue | 0.493461866 | 0.000772577 |
| STAC3 | blue | 0.170098439 | 0.275487178 |
| STAMBPL1 | blue | 0.113233403 | 0.469697475 |
| STARD4 | blue | 0.268867698 | 0.081264906 |
| STARD9 | lightcyan | 0.326881143 | 0.03239504 |
| STAT1 | lightyellow | 0.319045086 | 0.037040867 |
| STAT2 | lightyellow | 0.272876203 | 0.076658637 |
| STAT3 | blue | 0.398513615 | 0.008124125 |
| STAT4 | blue | 0.235486013 | 0.128473105 |
| STAU1 | blue | -0.232679131 | 0.133222904 |
| STC2 | blue | -0.186533422 | 0.231039951 |
| STIL | blue | 0.021488394 | 0.891210751 |
| STK10 | blue | 0.482085657 | 0.001062258 |
| STK11IP | blue | 0.096081066 | 0.539941998 |
| STK17A | blue | 0.279788371 | 0.069199627 |
| STK24 | blue | -0.358773068 | 0.018149104 |
| STK3 | blue | 0.048737157 | 0.756288737 |
| STK32B | blue | -0.250305713 | 0.105471781 |
| STK38 | blue | 0.165964412 | 0.287496173 |
| STK4 | blue | 0.253656962 | 0.100736407 |
| STMN3 | blue | 0.223273754 | 0.150093464 |
| STMND1 | blue | -0.075837966 | 0.62885021 |
| STOML1 | blue | 0.016584182 | 0.915937509 |
| STOML2 | blue | -0.272205963 | 0.077414217 |
| STOX1 | blue | -0.063028202 | 0.688034112 |
| STOX2 | lightcyan | 0.390482338 | 0.009631375 |
| STRA8 | blue | -0.078171244 | 0.618299517 |
| STRIP1 | blue | 0.146689062 | 0.347909992 |
| STRN | blue | 0.216281964 | 0.163615283 |
| STRN3 | blue | 0.080260627 | 0.608915481 |
| STT3B | blue | 0.123187795 | 0.431283393 |
| STX10 | blue | -0.124234003 | 0.42735069 |
| STX17 | blue | -0.01707707 | 0.913448461 |
| STX1A | blue | -0.019268393 | 0.902392669 |
| STX1B | blue | -0.127865229 | 0.413858032 |
| STX6 | blue | -0.053178153 | 0.734853285 |
| STXBP2 | blue | -0.051306578 | 0.743863685 |
| STXBP5 | blue | 0.532094244 | 0.000240328 |
| STYX | blue | 0.100529815 | 0.52124924 |
| STYXL1 | blue | -0.222125305 | 0.152256263 |
| SUCLA2 | blue | -0.336494275 | 0.027362854 |
| SUCNR1 | blue | 0.13050638 | 0.404198676 |
| SUGP2 | blue | -0.074259425 | 0.636029889 |
| SULT1C4 | blue | -0.10218038 | 0.514396448 |
| SULT2B1 | blue | -0.145905872 | 0.35051811 |
| SULT4A1 | blue | 0.136612021 | 0.382372528 |
| SULT6B1 | blue | -0.002349355 | 0.988070638 |
| SUMF2 | blue | -0.07122259 | 0.649934883 |
| SUMO3 | blue | 0.104583215 | 0.504501666 |
| SUN1 | blue | -0.009541248 | 0.951579157 |
| SURF2 | blue | -0.075316771 | 0.631217069 |
| SUSD5 | blue | 0.022440287 | 0.88642241 |
| SV2A | blue | 0.420996703 | 0.004934541 |
| SV2B | blue | 0.297366451 | 0.052798987 |
| SVIL | blue | 0.258483279 | 0.09420551 |
| SVOPL | blue | -0.242622421 | 0.116968554 |
| SWT1 | blue | -0.02451913 | 0.875979233 |
| SYCE1 | blue | -0.182076005 | 0.242577253 |
| SYCE3 | blue | 0.054579515 | 0.728129511 |
| SYCP1 | blue | 0.007210292 | 0.96339923 |
| SYN3 | blue | 0.290114453 | 0.059138664 |
| SYNDIG1 | blue | -0.04506182 | 0.774166966 |
| SYNE3 | blue | 0.320465837 | 0.036160227 |
| SYNGR4 | blue | -0.184182332 | 0.237077679 |
| SYNPO2 | blue | 0.133146825 | 0.394673045 |
| SYNPR | blue | -0.039591407 | 0.800989435 |
| SYP | blue | 0.001298449 | 0.993406677 |
| SYPL1 | blue | -0.3386141 | 0.026345431 |
| SYT1 | blue | 0.001824725 | 0.99073443 |
| SYT10 | blue | -0.26161557 | 0.090145105 |
| SYT12 | blue | -0.16945281 | 0.277340621 |
| SYT13 | blue | -0.22632891 | 0.144449838 |
| SYT15 | blue | 0.210488312 | 0.175472738 |
| SYT2 | blue | 0.101274063 | 0.51815369 |
| SYT3 | blue | 0.085822509 | 0.58423875 |
| SYT4 | blue | 0.060354536 | 0.70063772 |
| SYT5 | blue | -0.060538939 | 0.69976585 |
| SYT7 | blue | 0.034573593 | 0.825793811 |
| SYTL5 | blue | -0.104128062 | 0.506368518 |
| TAAR2 | blue | -0.086042427 | 0.583272306 |
| TAAR5 | blue | 0.101890587 | 0.515596334 |
| TAAR8 | blue | -0.104437152 | 0.505100376 |
| TAAR9 | blue | -0.301671239 | 0.049302233 |
| TAC1 | blue | 0.175324142 | 0.260785344 |
| TAC3 | blue | 0.068967201 | 0.660338655 |
| TAC4 | blue | 0.038955257 | 0.804123958 |
| TACC1 | blue | 0.217251913 | 0.161688411 |
| TACC3 | blue | 0.264662928 | 0.086326237 |
| TACSTD2 | blue | -0.289902303 | 0.059332895 |
| TADA1 | lightcyan | -0.360423971 | 0.017585798 |
| TADA2A | blue | -0.095349083 | 0.543048286 |
| TADA2B | blue | -0.078742027 | 0.615729919 |
| TAF1 | blue | 0.118191234 | 0.450341713 |
| TAF10 | blue | -0.107536836 | 0.492472576 |
| TAF11 | blue | 0.237419956 | 0.125275003 |
| TAF1A | blue | 0.072376389 | 0.644637728 |
| TAF1B | blue | -0.314466462 | 0.039999247 |
| TAF5L | blue | 0.295799439 | 0.05412034 |
| TAF6 | blue | 0.077778271 | 0.620071251 |
| TAF6L | blue | 0.100623131 | 0.520860608 |
| TAF8 | blue | 0.106414879 | 0.497024407 |
| TAL1 | blue | 0.087756331 | 0.575765166 |
| TANGO6 | blue | 0.008582355 | 0.956440518 |
| TANK | blue | 0.475499723 | 0.001270993 |
| TAOK2 | lightcyan | 0.401996909 | 0.00753647 |
| TAP1 | lightyellow | 0.475144787 | 0.001283212 |
| TAP2 | lightyellow | 0.496826808 | 0.000701642 |
| TARBP2 | blue | -0.185523203 | 0.233621229 |
| TARDBP | blue | 0.317986428 | 0.037708434 |
| TARS2 | blue | -0.126106975 | 0.420360667 |
| TAS1R1 | blue | -0.247974597 | 0.108864672 |
| TAS1R2 | blue | -0.214541305 | 0.167114912 |
| TAS1R3 | lightcyan | -0.302201239 | 0.048884965 |
| TAS2R14 | blue | 0.164555808 | 0.29166455 |
| TAS2R16 | blue | 0.117734232 | 0.452107469 |
| TAS2R20 | blue | 0.275125335 | 0.074165358 |
| TAS2R3 | blue | 0.358672361 | 0.018183952 |
| TAS2R38 | blue | -0.158369317 | 0.310432543 |
| TAS2R39 | blue | 0.117376309 | 0.453493029 |
| TAS2R4 | blue | 0.247778553 | 0.109153755 |
| TAS2R41 | blue | -0.076650162 | 0.625169169 |
| TAS2R46 | blue | 0.260875945 | 0.091091431 |
| TBC1D19 | blue | -0.298212435 | 0.052096491 |
| TBC1D2 | blue | -0.096492976 | 0.538197762 |
| TBC1D21 | blue | -0.208810378 | 0.179019295 |
| TBC1D22A | blue | 0.497976933 | 0.000678767 |
| TBC1D26 | blue | -0.128090088 | 0.413030582 |
| TBC1D30 | blue | -0.071402229 | 0.649109021 |
| TBC1D7 | blue | 0.159891273 | 0.305745759 |
| TBC1D8 | blue | 0.42861155 | 0.00413593 |
| TBC1D8B | blue | -0.165170339 | 0.289841231 |
| TBC1D9 | blue | 0.056853165 | 0.7172634 |
| TBCE | blue | -0.158870029 | 0.308885613 |
| TBK1 | lightcyan | 0.199800971 | 0.198941402 |
| TBL1XR1 | blue | 0.187189419 | 0.229374258 |
| TBL2 | blue | 0.005122288 | 0.97399397 |
| TBPL1 | blue | -0.022939455 | 0.883913012 |
| TBX10 | blue | 0.055972364 | 0.72146648 |
| TBX15 | blue | 0.053261166 | 0.734454434 |
| TBX18 | blue | 0.02468532 | 0.875145242 |
| TBX20 | blue | 0.25694207 | 0.096254508 |
| TBX3 | blue | 0.415252498 | 0.005622753 |
| TCAIM | lightcyan | -0.439160449 | 0.003216994 |
| TCEAL5 | blue | -0.158291318 | 0.31067396 |
| TCEANC2 | blue | 0.041308679 | 0.792543392 |
| TCF25 | blue | 0.360637282 | 0.017514102 |
| TCF4 | lightcyan | 0.562443453 | 8.66E-05 |
| TCF7 | blue | 0.164352728 | 0.29226872 |
| TCF7L1 | blue | 0.052398416 | 0.738603018 |
| TCFL5 | blue | -0.141113587 | 0.366735759 |
| TCHH | blue | 0.301008819 | 0.049827793 |
| TCHHL1 | blue | -0.138502776 | 0.375757418 |
| TCHP | blue | 0.167902088 | 0.281825706 |
| TCOF1 | blue | 0.107306066 | 0.493407059 |
| TCP11 | blue | -0.001266727 | 0.993567755 |
| TDG | blue | 0.133942954 | 0.391826808 |
| TDGF1 | blue | 0.022731909 | 0.884956245 |
| TDO2 | blue | 0.103714765 | 0.508066723 |
| TDRD1 | blue | -0.173593847 | 0.265594252 |
| TEAD2 | blue | 0.417296208 | 0.005368906 |
| TEAD3 | blue | 0.297474047 | 0.05270922 |
| TECPR1 | blue | 0.12679606 | 0.417805334 |
| TECTA | blue | -0.053595776 | 0.732847447 |
| TECTB | blue | -0.049249795 | 0.75380479 |
| TEDDM1 | blue | -0.288183488 | 0.060925393 |
| TEK | lightcyan | 0.474678252 | 0.001299431 |
| TEKT1 | blue | -0.08691174 | 0.579459103 |
| TEKT3 | blue | -0.031320899 | 0.841964263 |
| TEKT4 | blue | -0.138580151 | 0.375488164 |
| TEN1 | blue | -0.071400881 | 0.649115217 |
| TEP1 | blue | 0.281765565 | 0.067175149 |
| TERF2 | blue | 0.012335956 | 0.937421164 |
| TERT | blue | -0.461129592 | 0.001857314 |
| TESPA1 | blue | 0.025867022 | 0.869219007 |
| TET2 | blue | 0.083502483 | 0.594477664 |
| TEX10 | blue | 0.250086501 | 0.105787355 |
| TEX2 | blue | 0.031961484 | 0.838774357 |
| TEX22 | blue | -0.123339914 | 0.430710325 |
| TEX264 | blue | -0.340446509 | 0.0254915 |
| TEX38 | blue | 0.165829806 | 0.287892821 |
| TF | blue | 0.022951268 | 0.883853641 |
| TFAP4 | blue | -0.110720141 | 0.47967586 |
| TFB2M | blue | -0.179899921 | 0.248349009 |
| TFCP2 | blue | -0.01642651 | 0.916733909 |
| TFDP1 | blue | 0.179252193 | 0.25008473 |
| TFDP2 | blue | -0.35531217 | 0.019379489 |
| TFEB | blue | 0.123540975 | 0.429953534 |
| TFG | blue | 0.237214915 | 0.125611212 |
| TFPI2 | lightyellow | 0.110328603 | 0.481240338 |
| TGDS | blue | -0.369414615 | 0.014770444 |
| TGFA | blue | -0.136467417 | 0.382881265 |
| TGIF1 | blue | -0.309282029 | 0.043579127 |
| TGIF2 | blue | -0.030837377 | 0.844373703 |
| TGM2 | lightyellow | 0.274454279 | 0.074902483 |
| TGM3 | blue | -0.066157428 | 0.673388495 |
| TGM5 | blue | -0.089070102 | 0.570040584 |
| TH | blue | -0.028537288 | 0.855854131 |
| THAP2 | blue | -0.325273461 | 0.0333068 |
| THAP4 | blue | -0.198877512 | 0.201068163 |
| THAP7 | blue | -0.075858552 | 0.628756799 |
| THAP9 | blue | -0.283531452 | 0.065406971 |
| THBS3 | blue | 0.284601882 | 0.06435327 |
| THBS4 | blue | -0.099328974 | 0.526263145 |
| THEM4 | blue | -0.011104463 | 0.943657814 |
| THEM5 | blue | -0.211726459 | 0.17288829 |
| THEM6 | blue | -0.261947924 | 0.089722357 |
| THEMIS | blue | 0.390066902 | 0.00971547 |
| THG1L | blue | 0.059311022 | 0.705578659 |
| THNSL1 | blue | -0.38919146 | 0.009894746 |
| THNSL2 | blue | -0.290454865 | 0.058828066 |
| THRB | blue | -0.004490814 | 0.977199084 |
| THRSP | blue | -0.083883599 | 0.592790283 |
| THSD1 | blue | 0.286756814 | 0.062272949 |
| THSD4 | blue | 0.054958305 | 0.72631549 |
| THTPA | blue | 0.017094592 | 0.913359991 |
| THUMPD1 | blue | -0.094485832 | 0.546722614 |
| TIAL1 | blue | 0.27113815 | 0.078630044 |
| TIAM2 | blue | 0.219917695 | 0.156477705 |
| TICRR | blue | 0.034957354 | 0.823890529 |
| TIE1 | lightcyan | 0.499071909 | 0.000657612 |
| TIFAB | lightyellow | 0.10457532 | 0.50453402 |
| TIGD1 | blue | -0.155437688 | 0.319588294 |
| TIMELESS | blue | -0.004115237 | 0.97910554 |
| TIMM13 | blue | -0.386663417 | 0.010428458 |
| TIMM17B | blue | -0.287633453 | 0.061442143 |
| TIMM21 | blue | -0.184389992 | 0.236540117 |
| TIMM44 | blue | -0.302404248 | 0.048725895 |
| TIMM8B | blue | -0.127094549 | 0.416701191 |
| TIMP3 | blue | 0.187872413 | 0.227648774 |
| TINAGL1 | blue | 0.202286331 | 0.193296297 |
| TINF2 | blue | 0.49852051 | 0.00066819 |
| TIRAP | blue | 0.091247645 | 0.560610233 |
| TK2 | blue | 0.194077639 | 0.212379712 |
| TKTL1 | blue | 0.050986506 | 0.745408049 |
| TLCD1 | blue | -0.200127363 | 0.198193509 |
| TLE2 | blue | 0.25421497 | 0.099964032 |
| TLE3 | blue | 0.194390867 | 0.211628328 |
| TLE6 | blue | 0.019792828 | 0.89974938 |
| TLL1 | blue | -0.005116244 | 0.974024647 |
| TLR10 | blue | 0.190272301 | 0.221656533 |
| TLX2 | blue | -0.058480205 | 0.709521076 |
| TM2D1 | blue | 0.123994998 | 0.428247334 |
| TM4SF1 | blue | 0.435968132 | 0.003474041 |
| TM4SF18 | blue | 0.389643677 | 0.009801789 |
| TM4SF20 | blue | 0.176057694 | 0.258764241 |
| TMA16 | blue | 0.106756892 | 0.495634563 |
| TMC1 | lightcyan | -0.366757947 | 0.015559466 |
| TMC5 | blue | 0.28244038 | 0.06649504 |
| TMC7 | blue | 0.295919149 | 0.054018468 |
| TMCO6 | blue | -0.399422155 | 0.007967166 |
| TMED3 | blue | -0.405129716 | 0.007039651 |
| TMED9 | blue | 0.053905587 | 0.731360559 |
| TMEFF1 | blue | 0.130877954 | 0.402850242 |
| TMEM101 | blue | -0.186768654 | 0.230441705 |
| TMEM102 | blue | -0.164862765 | 0.29075288 |
| TMEM117 | blue | 0.183630183 | 0.238511051 |
| TMEM126A | blue | -0.296394059 | 0.053615844 |
| TMEM132B | blue | -0.062408306 | 0.690949019 |
| TMEM132D | blue | -0.107557821 | 0.492387643 |
| TMEM138 | blue | 0.081366489 | 0.603973568 |
| TMEM139 | blue | -0.05274725 | 0.736924738 |
| TMEM141 | blue | -0.427494739 | 0.004245483 |
| TMEM143 | blue | -0.256624594 | 0.096680817 |
| TMEM150B | blue | 0.042916306 | 0.784657782 |
| TMEM156 | blue | 0.186349442 | 0.231508592 |
| TMEM159 | blue | -0.448216988 | 0.002576286 |
| TMEM161B | blue | 0.303900082 | 0.047566671 |
| TMEM164 | blue | -0.364404112 | 0.016288096 |
| TMEM165 | blue | 0.28077185 | 0.068186681 |
| TMEM168 | blue | 0.155953951 | 0.317963741 |
| TMEM178B | blue | -0.046640201 | 0.766474392 |
| TMEM181 | blue | 0.1099929 | 0.482583845 |
| TMEM184B | blue | 0.356483437 | 0.018955467 |
| TMEM184C | blue | -0.291991825 | 0.057441913 |
| TMEM200A | blue | -0.147345108 | 0.345734451 |
| TMEM200B | blue | 0.029325205 | 0.85191797 |
| TMEM205 | blue | -0.170670248 | 0.273852467 |
| TMEM209 | blue | -0.165600167 | 0.288570327 |
| TMEM214 | blue | 0.295270455 | 0.054572346 |
| TMEM217 | blue | 0.007488349 | 0.96198877 |
| TMEM225 | blue | 0.328884783 | 0.031287635 |
| TMEM229B | blue | -0.193594947 | 0.213541242 |
| TMEM233 | blue | 0.174292328 | 0.263645976 |
| TMEM234 | blue | -0.274214502 | 0.07516726 |
| TMEM235 | blue | -0.064073242 | 0.68313018 |
| TMEM244 | blue | -0.310719483 | 0.042561413 |
| TMEM247 | blue | -0.012729625 | 0.935428263 |
| TMEM251 | blue | -0.319313913 | 0.036872902 |
| TMEM252 | blue | -0.003100346 | 0.98425774 |
| TMEM253 | blue | 0.186107801 | 0.232125096 |
| TMEM254 | blue | -0.407033466 | 0.006751785 |
| TMEM260 | blue | 0.202460059 | 0.192905983 |
| TMEM31 | blue | -0.223263516 | 0.150112643 |
| TMEM38A | blue | -0.197343794 | 0.204635567 |
| TMEM38B | blue | -0.349196985 | 0.021725048 |
| TMEM39A | blue | 0.417953603 | 0.005289397 |
| TMEM44 | blue | 0.244613244 | 0.113902562 |
| TMEM50B | blue | 0.130999891 | 0.402408302 |
| TMEM51 | blue | -0.07994532 | 0.610327707 |
| TMEM53 | blue | -0.299317617 | 0.05119012 |
| TMEM54 | blue | -0.251559628 | 0.103680464 |
| TMEM59L | blue | -0.003514655 | 0.982154369 |
| TMEM63B | blue | 0.118919336 | 0.447536275 |
| TMEM67 | blue | 0.121919308 | 0.436078622 |
| TMEM68 | blue | -0.137149651 | 0.380484587 |
| TMEM70 | blue | -0.079913784 | 0.610469029 |
| TMEM74B | blue | 0.260217005 | 0.091940984 |
| TMEM79 | blue | -0.312972579 | 0.041005228 |
| TMEM81 | blue | 0.039324595 | 0.802303735 |
| TMEM87B | blue | 0.07777422 | 0.620089528 |
| TMEM95 | blue | -0.000450993 | 0.997709905 |
| TMLHE | blue | 0.058542917 | 0.70922323 |
| TMOD3 | blue | 0.120236016 | 0.442487377 |
| TMOD4 | blue | 0.15497511 | 0.321048346 |
| TMPRSS12 | blue | -0.052338454 | 0.738891619 |
| TMPRSS13 | blue | 0.016040698 | 0.918682978 |
| TMSB15B | blue | -0.059536195 | 0.704511465 |
| TMTC1 | blue | 0.151221778 | 0.333049876 |
| TMX1 | blue | 0.326466651 | 0.032628117 |
| TNF | blue | 0.441309061 | 0.003053524 |
| TNFAIP1 | lightcyan | 0.452033702 | 0.002341845 |
| TNFAIP8L1 | blue | -0.145947431 | 0.350379417 |
| TNFRSF10B | blue | 0.253268107 | 0.101277348 |
| TNFRSF11A | blue | -0.100390608 | 0.521829259 |
| TNFRSF18 | blue | 0.284796307 | 0.064163339 |
| TNFSF10 | blue | 0.101134183 | 0.518734795 |
| TNFSF18 | blue | 0.157072365 | 0.314462274 |
| TNFSF9 | blue | -0.081885843 | 0.601658665 |
| TNIP2 | blue | 0.039066644 | 0.803574897 |
| TNIP3 | blue | 0.141946452 | 0.363885388 |
| TNK1 | lightyellow | 0.132052723 | 0.398604142 |
| TNKS1BP1 | blue | 0.181693393 | 0.243585439 |
| TNKS2 | blue | 0.312947273 | 0.041022446 |
| TNP1 | blue | -0.197929848 | 0.203267218 |
| TNR | blue | -0.154727009 | 0.321833161 |
| TNRC6C | blue | 0.034277108 | 0.827264918 |
| TNS4 | blue | 0.130899196 | 0.402773233 |
| TOB1 | blue | -0.286392288 | 0.06262104 |
| TOM1 | blue | -0.253867969 | 0.100443803 |
| TOMM34 | blue | -0.075220055 | 0.631656681 |
| TOMM5 | blue | -0.311161396 | 0.042252457 |
| TOP1 | blue | -0.189279725 | 0.224121547 |
| TOP3A | blue | 0.000936526 | 0.995244444 |
| TOPAZ1 | lightcyan | -0.110790374 | 0.47939551 |
| TOPBP1 | blue | 0.295723366 | 0.054185157 |
| TOR4A | blue | 0.188339876 | 0.226472944 |
| TOX | blue | 0.238864445 | 0.122925548 |
| TOX2 | blue | -0.004655697 | 0.97636217 |
| TP53AIP1 | blue | -0.032696282 | 0.835118447 |
| TP53I3 | blue | -0.01962176 | 0.900611489 |
| TP53TG3B | blue | -0.175086258 | 0.261443019 |
| TP73 | blue | 0.017644014 | 0.910586449 |
| TPH1 | blue | 0.093421736 | 0.551268053 |
| TPH2 | blue | -0.044419641 | 0.777302927 |
| TPI1 | blue | -0.375627933 | 0.013056615 |
| TPO | blue | -0.046442723 | 0.76743565 |
| TPP1 | blue | -0.142401279 | 0.362334452 |
| TPP2 | blue | 0.32526886 | 0.033309439 |
| TPRKB | blue | 0.240211832 | 0.120764044 |
| TPRN | blue | -0.236740623 | 0.126391514 |
| TPRX1 | blue | 0.163441835 | 0.294988601 |
| TPSD1 | blue | -0.006417476 | 0.96742142 |
| TPST2 | blue | 0.299526995 | 0.051019847 |
| TPT1 | blue | 0.108376141 | 0.489081603 |
| TPTE2 | blue | 0.002859596 | 0.98548003 |
| TRABD2A | blue | 0.122964422 | 0.432125662 |
| TRADD | blue | -0.453598825 | 0.002251281 |
| TRAF3IP2 | blue | 0.062207946 | 0.691892105 |
| TRAF4 | blue | -0.256228791 | 0.097214339 |
| TRAF6 | blue | 0.223940032 | 0.148849093 |
| TRAF7 | blue | 0.068002525 | 0.66480808 |
| TRAK1 | blue | 0.298812159 | 0.051603061 |
| TRANK1 | blue | 0.300968627 | 0.049859826 |
| TRAPPC11 | blue | 0.35198068 | 0.020629406 |
| TRAPPC12 | blue | 0.158508281 | 0.310002728 |
| TRAPPC6A | blue | -0.335635903 | 0.027784015 |
| TRDMT1 | blue | 0.145360395 | 0.35234165 |
| TREX1 | blue | 0.073508588 | 0.63945657 |
| TREX2 | blue | -0.075774719 | 0.62913723 |
| TRIB1 | blue | -0.281176645 | 0.067773182 |
| TRIM14 | blue | 0.070856559 | 0.651618935 |
| TRIM21 | lightyellow | 0.115858592 | 0.459393845 |
| TRIM23 | blue | 0.010152819 | 0.948479525 |
| TRIM24 | blue | 0.057703727 | 0.713212429 |
| TRIM27 | blue | 0.356347096 | 0.01900442 |
| TRIM31 | blue | -0.079840836 | 0.610795986 |
| TRIM34 | blue | 0.491248878 | 0.000822654 |
| TRIM38 | lightcyan | 0.399992645 | 0.007869949 |
| TRIM43 | lightcyan | -0.284754817 | 0.064203833 |
| TRIM47 | blue | 0.23141118 | 0.135410932 |
| TRIM49 | blue | -0.282738804 | 0.066196021 |
| TRIM49B | blue | -0.304191246 | 0.047343644 |
| TRIM56 | blue | -0.040668144 | 0.795691047 |
| TRIM61 | blue | 0.105333867 | 0.501430448 |
| TRIM62 | blue | 0.054648258 | 0.72780019 |
| TRIM63 | blue | -0.294415766 | 0.055309062 |
| TRIM64 | blue | 0.130017293 | 0.40597752 |
| TRIM64C | blue | 0.06135997 | 0.695888591 |
| TRIM65 | blue | -0.099158009 | 0.526978904 |
| TRIM67 | blue | -0.18066124 | 0.246319291 |
| TRIM69 | lightyellow | 0.10645551 | 0.496859187 |
| TRIM7 | blue | 0.240414337 | 0.120441673 |
| TRIM71 | blue | -0.172300751 | 0.269226228 |
| TRIM72 | blue | -0.160896681 | 0.302674573 |
| TRIM77 | blue | -0.000355371 | 0.998195461 |
| TRIM9 | blue | 0.065449584 | 0.676691211 |
| TRIML2 | blue | 0.085302909 | 0.586525009 |
| TRIOBP | blue | 0.050347319 | 0.748495096 |
| TRIP10 | blue | 0.225697623 | 0.145602947 |
| TRIP13 | blue | 0.206299466 | 0.184421884 |
| TRIP4 | blue | -0.234060087 | 0.130869961 |
| TRIP6 | blue | -0.191821011 | 0.217847861 |
| TRMT10A | blue | -0.254092497 | 0.100133164 |
| TRMT11 | blue | 0.042976293 | 0.784363942 |
| TRMT44 | blue | 0.254431607 | 0.0996654 |
| TRMT5 | blue | -0.276170489 | 0.073028681 |
| TRMT61A | blue | -0.232322017 | 0.133836472 |
| TRMU | blue | -0.150441635 | 0.335578981 |
| TRNT1 | blue | 0.243324195 | 0.115880739 |
| TRPC4AP | blue | 0.038533421 | 0.806204157 |
| TRPC5OS | blue | -0.266991319 | 0.08349409 |
| TRPM2 | blue | -0.051473514 | 0.743058607 |
| TRPS1 | blue | -0.059913799 | 0.702723095 |
| TRPT1 | blue | -0.286790525 | 0.062240836 |
| TRPV2 | blue | 0.382580769 | 0.011342419 |
| TSC22D4 | blue | -0.112573953 | 0.472304942 |
| TSEN54 | blue | -0.113301774 | 0.469427572 |
| TSGA10 | blue | -0.22143048 | 0.15357582 |
| TSHZ2 | blue | 0.099254838 | 0.526573462 |
| TSKS | blue | -0.146882581 | 0.347267388 |
| TSPAN14 | blue | 0.131535433 | 0.400470632 |
| TSPAN19 | blue | 0.125347566 | 0.423186991 |
| TSPAN32 | blue | 0.261400063 | 0.09042005 |
| TSPAN5 | blue | 0.021882536 | 0.889227611 |
| TSPAN8 | blue | -0.272908941 | 0.076621879 |
| TSPYL2 | blue | 0.093185094 | 0.552281332 |
| TSPYL6 | blue | -0.030772597 | 0.844696618 |
| TSR1 | blue | 0.027429636 | 0.861393401 |
| TSSK1B | blue | -0.229185271 | 0.139316523 |
| TSSK2 | blue | -0.188508222 | 0.226050522 |
| TSSK3 | blue | 0.316335299 | 0.038769244 |
| TSSK4 | blue | 0.097779183 | 0.532768904 |
| TSTD1 | blue | -0.367137067 | 0.01544473 |
| TTBK1 | lightcyan | -0.414189431 | 0.005758869 |
| TTBK2 | blue | 0.104846338 | 0.503424038 |
| TTC13 | blue | 0.359173523 | 0.018011085 |
| TTC16 | blue | 0.031150692 | 0.84281226 |
| TTC17 | blue | 0.185437423 | 0.233841315 |
| TTC22 | blue | -0.267877825 | 0.082435031 |
| TTC23 | blue | -0.237422129 | 0.125271442 |
| TTC30A | blue | -0.222032744 | 0.152431565 |
| TTC30B | blue | -0.325074494 | 0.033421095 |
| TTC31 | blue | 0.072030835 | 0.646222379 |
| TTC32 | blue | -0.171191801 | 0.272367007 |
| TTC33 | blue | -0.318139026 | 0.037611606 |
| TTC37 | blue | 0.146099239 | 0.349873069 |
| TTF2 | blue | 0.032896952 | 0.834120625 |
| TTI2 | blue | -0.016952316 | 0.914078385 |
| TTK | blue | -0.030353911 | 0.846784269 |
| TTL | blue | 0.333523915 | 0.028843215 |
| TTLL10 | blue | -0.050092588 | 0.749726444 |
| TTLL12 | blue | -0.12852756 | 0.411423447 |
| TTLL4 | blue | 0.313872126 | 0.040397031 |
| TTPA | blue | -0.138852546 | 0.374541191 |
| TTYH2 | blue | -0.11238293 | 0.473061679 |
| TUBA1A | blue | -0.05580179 | 0.722281381 |
| TUBA3D | blue | -0.239728132 | 0.121536686 |
| TUBA4A | blue | -0.242803658 | 0.116686883 |
| TUBA4B | blue | -0.226318371 | 0.144469032 |
| TUBA8 | blue | 0.045919688 | 0.769983267 |
| TUBB | blue | -0.172227703 | 0.269432375 |
| TUBB1 | blue | -0.071100729 | 0.650495359 |
| TUBD1 | blue | 0.271646152 | 0.078049773 |
| TUBGCP3 | blue | -0.239560079 | 0.121805995 |
| TUFM | blue | -0.318507145 | 0.037378861 |
| TULP2 | blue | -0.05049508 | 0.747781113 |
| TULP3 | blue | -0.013705224 | 0.930491126 |
| TUSC2 | blue | -0.225762906 | 0.145483388 |
| TVP23C | blue | 0.155880697 | 0.318193935 |
| TWF1 | blue | 0.086662178 | 0.58055264 |
| TWIST1 | blue | -0.113921335 | 0.466985575 |
| TXLNB | blue | 0.161876197 | 0.299701545 |
| TXNDC11 | blue | -0.313082249 | 0.040930682 |
| TXNDC17 | blue | -0.233370872 | 0.132040371 |
| TYR | blue | 0.009958886 | 0.949462361 |
| TYSND1 | blue | -0.322378949 | 0.035001618 |
| TYW1 | blue | 0.21379615 | 0.168629498 |
| U2SURP | blue | -0.074505307 | 0.634909358 |
| UAP1L1 | blue | -0.164537159 | 0.291719997 |
| UBA2 | blue | 0.114994027 | 0.462773653 |
| UBA7 | blue | 0.463417299 | 0.001750375 |
| UBAC1 | blue | -0.09896627 | 0.527782201 |
| UBALD1 | blue | -0.295662318 | 0.054237217 |
| UBAP2L | blue | 0.190009994 | 0.222306134 |
| UBASH3A | blue | 0.133681591 | 0.392759883 |
| UBASH3B | blue | 0.267963656 | 0.082333052 |
| UBE2E1 | blue | -0.076361471 | 0.626476549 |
| UBE2G1 | blue | -0.285481021 | 0.063497999 |
| UBE2G2 | blue | 0.083144412 | 0.596064934 |
| UBE2L6 | lightyellow | 0.032289013 | 0.837144354 |
| UBE2M | blue | -0.142333494 | 0.362565343 |
| UBE2Q1 | blue | -0.198457327 | 0.20204113 |
| UBE2QL1 | blue | -0.318622439 | 0.037306208 |
| UBE2V1 | blue | 0.163611675 | 0.294480234 |
| UBE2Z | blue | -0.211685906 | 0.172972501 |
| UBE3B | blue | -0.307486766 | 0.044877867 |
| UBE3C | blue | 0.208067483 | 0.180605795 |
| UBL4A | blue | -0.057238939 | 0.715425118 |
| UBL4B | blue | 0.054546434 | 0.728288003 |
| UBOX5 | blue | 0.030359403 | 0.846756878 |
| UBR5 | blue | 0.310474463 | 0.042733505 |
| UBTD1 | blue | -0.011927357 | 0.939490038 |
| UBTD2 | blue | 0.248165875 | 0.10858318 |
| UBXN10 | blue | -0.257143291 | 0.095985058 |
| UBXN2A | blue | 0.024781822 | 0.874661024 |
| UBXN7 | blue | 0.1658244 | 0.287908759 |
| UBXN8 | blue | -0.089718561 | 0.567224682 |
| UCK2 | blue | 0.243806958 | 0.115136867 |
| UCN | blue | -0.299619652 | 0.050944641 |
| UCP1 | blue | 0.21928241 | 0.157708198 |
| UCP3 | blue | 0.440874781 | 0.003085964 |
| UGGT1 | blue | 0.215522418 | 0.165135767 |
| UGP2 | blue | -0.385259714 | 0.010735311 |
| UGT2A2 | blue | -0.266480036 | 0.084109697 |
| UGT8 | blue | -0.11844344 | 0.449368859 |
| UHRF1BP1 | blue | 0.343172079 | 0.024264078 |
| UHRF1BP1L | blue | -0.160193498 | 0.30482048 |
| ULBP2 | blue | -0.086742506 | 0.580200554 |
| ULBP3 | blue | 0.072509516 | 0.644027643 |
| UMODL1 | blue | 0.008580946 | 0.956447662 |
| UNC119 | blue | -0.380523485 | 0.011828344 |
| UNC119B | blue | -0.338291246 | 0.026498324 |
| UNC13A | blue | 0.145851111 | 0.350700918 |
| UNC13D | blue | 0.084992877 | 0.587891061 |
| UNC45B | blue | -0.244853776 | 0.113536293 |
| UNC5B | lightcyan | 0.454839659 | 0.002181691 |
| UNC79 | blue | -0.178097601 | 0.253198889 |
| UNC93A | blue | -0.021177214 | 0.89277693 |
| UPF2 | blue | -0.147527335 | 0.345131644 |
| UPF3A | blue | -0.311396267 | 0.042088997 |
| UPK1A | lightcyan | -0.321441709 | 0.035565353 |
| UPRT | blue | 0.117846936 | 0.451671656 |
| UQCRB | blue | -0.339136125 | 0.02609977 |
| UQCRC2 | blue | -0.326012498 | 0.032885081 |
| UQCRH | blue | -0.292438751 | 0.057043785 |
| UQCRHL | blue | -0.407269833 | 0.006716765 |
| UQCRQ | blue | -0.329668456 | 0.030863096 |
| URB1 | blue | 0.095195398 | 0.543701561 |
| URGCP | blue | 0.083499205 | 0.594492187 |
| USH1G | blue | 0.142688039 | 0.361358665 |
| USP15 | lightcyan | 0.334764933 | 0.028216842 |
| USP17L10 | blue | 0.295416633 | 0.054447138 |
| USP17L15 | blue | 0.325932693 | 0.032930407 |
| USP17L23 | blue | 0.264812322 | 0.086142307 |
| USP17L30 | blue | 0.123945732 | 0.42843229 |
| USP17L4 | blue | 0.021742998 | 0.889929627 |
| USP18 | lightyellow | 0.384876923 | 0.010820318 |
| USP19 | blue | 0.139973665 | 0.37065865 |
| USP21 | blue | -0.185580806 | 0.233473517 |
| USP26 | blue | 0.091400521 | 0.559950911 |
| USP27X | blue | 0.012212386 | 0.938046799 |
| USP31 | blue | 0.004725374 | 0.976008506 |
| USP33 | blue | 0.388461028 | 0.010046491 |
| USP35 | blue | 0.198422769 | 0.202121299 |
| USP38 | blue | 0.062076076 | 0.692513066 |
| USP41 | lightyellow | 0.402057639 | 0.007526559 |
| USP48 | blue | 0.18864222 | 0.225714672 |
| USP49 | blue | 0.343156643 | 0.024270888 |
| USP51 | blue | -0.262516372 | 0.089002867 |
| USP6 | blue | 0.377528454 | 0.01256747 |
| USPL1 | blue | 0.254483257 | 0.099594302 |
| UTP15 | blue | -0.181524028 | 0.244032619 |
| UTP23 | blue | 0.035764712 | 0.819889666 |
| UTP3 | blue | -0.106961537 | 0.494803898 |
| UTS2 | blue | 0.1730308 | 0.267171695 |
| UTS2R | blue | -0.046555067 | 0.766888753 |
| VAC14 | blue | -0.278935715 | 0.070087429 |
| VAMP1 | blue | 0.165661781 | 0.288388445 |
| VAMP7 | blue | -0.18515021 | 0.234579245 |
| VANGL1 | blue | 0.052061681 | 0.740224223 |
| VANGL2 | blue | 0.08171639 | 0.602413542 |
| VAPB | blue | 0.148042069 | 0.343432393 |
| VARS2 | lightcyan | -0.418200719 | 0.005259776 |
| VASH1 | lightcyan | 0.497774528 | 0.000682744 |
| VEGFB | blue | -0.103494459 | 0.508973115 |
| VEZT | blue | 0.08122077 | 0.604623769 |
| VGLL3 | blue | -0.156716557 | 0.31557356 |
| VGLL4 | blue | 0.08360976 | 0.594002482 |
| VHLL | blue | 0.297227805 | 0.05291484 |
| VIM | blue | 0.271974589 | 0.077676403 |
| VIP | blue | 0.304487981 | 0.047117223 |
| VLDLR | blue | -0.347419536 | 0.022449824 |
| VMO1 | blue | 0.191581792 | 0.218433184 |
| VPS18 | blue | -0.255842831 | 0.097736771 |
| VPS36 | blue | 0.115518476 | 0.460721854 |
| VPS37B | blue | 0.036245513 | 0.817509191 |
| VPS37D | blue | 0.019265971 | 0.902404877 |
| VPS4A | blue | -0.156140297 | 0.31737864 |
| VPS53 | blue | -0.076115937 | 0.627589379 |
| VPS54 | blue | 0.233103196 | 0.132497031 |
| VPS8 | blue | 0.426131544 | 0.00438263 |
| VSTM2B | lightcyan | -0.319099798 | 0.037006632 |
| VSTM2L | blue | 0.185113673 | 0.234673232 |
| VTA1 | blue | -0.392602859 | 0.009211792 |
| VWA3B | blue | 0.028009923 | 0.858490599 |
| VWC2L | blue | -0.079642319 | 0.611686129 |
| VWF | blue | 0.148471337 | 0.34201923 |
| WASF1 | blue | 0.128408456 | 0.411860647 |
| WBP1L | blue | -0.070996925 | 0.650972929 |
| WBP2NL | blue | 0.147334384 | 0.345769947 |
| WBP4 | blue | 0.088576126 | 0.572189952 |
| WDFY1 | blue | 0.33975622 | 0.025810438 |
| WDHD1 | lightcyan | 0.286327931 | 0.062682655 |
| WDPCP | blue | 0.135056824 | 0.387864812 |
| WDR1 | lightcyan | 0.215002669 | 0.166182102 |
| WDR11 | blue | 0.275306508 | 0.073967328 |
| WDR25 | blue | -0.156492765 | 0.316273794 |
| WDR3 | blue | -0.040668135 | 0.795691089 |
| WDR38 | blue | 0.017388539 | 0.911875988 |
| WDR43 | blue | 0.049080459 | 0.754625025 |
| WDR46 | blue | -0.178622968 | 0.251778665 |
| WDR48 | blue | -0.144004121 | 0.356900693 |
| WDR5 | blue | -0.239713658 | 0.121559862 |
| WDR54 | blue | -0.163918452 | 0.293563416 |
| WDR59 | blue | -0.037467615 | 0.811465825 |
| WDR7 | blue | 0.528735036 | 0.00026751 |
| WDR74 | blue | -0.009962892 | 0.949442057 |
| WDR76 | blue | 0.087410639 | 0.577275804 |
| WDR77 | blue | 0.101421513 | 0.517541489 |
| WDR86 | blue | -0.017389273 | 0.911872281 |
| WDR88 | blue | -0.289902181 | 0.059333007 |
| WDR89 | blue | -0.266969932 | 0.083519771 |
| WDR90 | blue | -0.203295022 | 0.191037851 |
| WDSUB1 | blue | -0.406282051 | 0.006864167 |
| WFDC1 | blue | 0.441931093 | 0.003007581 |
| WFDC10A | blue | 0.012098197 | 0.938624967 |
| WFDC10B | blue | -0.293224482 | 0.056349203 |
| WFDC13 | blue | -0.364489975 | 0.016261019 |
| WFDC6 | blue | -0.028191027 | 0.85758503 |
| WFIKKN1 | blue | -0.132401636 | 0.397348043 |
| WFIKKN2 | blue | -0.099063004 | 0.527376855 |
| WHAMM | blue | 0.201975249 | 0.193996601 |
| WIPF1 | blue | 0.531878989 | 0.000241992 |
| WIPI1 | blue | -0.113045361 | 0.47044021 |
| WIPI2 | blue | 0.069524611 | 0.657761423 |
| WNK2 | blue | 0.081639321 | 0.602757002 |
| WNT10B | blue | 0.044957255 | 0.774677351 |
| WNT11 | blue | 0.010842317 | 0.944985843 |
| WNT16 | blue | -0.228874538 | 0.139868309 |
| WNT5B | blue | 0.297102003 | 0.053020139 |
| WNT7A | blue | -0.057028497 | 0.716427716 |
| WNT8A | blue | -0.252965147 | 0.101700342 |
| WNT9A | blue | -0.312735931 | 0.041166464 |
| WRAP73 | blue | 0.078322662 | 0.617617412 |
| WSB2 | blue | -0.086012444 | 0.583404029 |
| WWOX | blue | -0.165395533 | 0.289174931 |
| XAF1 | lightyellow | 0.441077659 | 0.003070772 |
| XAGE1A | blue | 0.180237957 | 0.247446399 |
| XAGE5 | blue | 0.064246253 | 0.682319545 |
| XCL1 | blue | 0.062794881 | 0.689130723 |
| XCR1 | blue | 0.056993743 | 0.716593339 |
| XG | blue | -0.053272573 | 0.734399636 |
| XIRP1 | blue | -0.097595091 | 0.533544287 |
| XIRP2 | blue | -0.438718006 | 0.003251593 |
| XKR3 | blue | -0.156412533 | 0.316525075 |
| XKR4 | blue | 0.009775194 | 0.950393359 |
| XKR6 | blue | 0.034987508 | 0.823741022 |
| XKRX | lightcyan | -0.228789168 | 0.14002019 |
| XPC | blue | -0.366538926 | 0.015626078 |
| XPNPEP1 | blue | 0.392598572 | 0.009212624 |
| XPO1 | blue | 0.211990549 | 0.172340606 |
| XPO5 | blue | 0.157118167 | 0.314319405 |
| XPO7 | blue | -0.255700337 | 0.097930195 |
| XPR1 | blue | -0.000959262 | 0.995128991 |
| XRCC2 | blue | 0.266346908 | 0.084270568 |
| XRRA1 | blue | 0.279064933 | 0.069952309 |
| YARS2 | blue | 0.082049411 | 0.6009304 |
| YBEY | blue | -0.388924993 | 0.009949874 |
| YDJC | blue | -0.260530773 | 0.091535691 |
| YEATS4 | blue | -0.395251952 | 0.008709763 |
| YIF1A | blue | -0.282255907 | 0.066680415 |
| YIPF7 | blue | 0.067561372 | 0.666855817 |
| YOD1 | blue | -0.270128155 | 0.079793757 |
| YPEL4 | blue | 0.046041721 | 0.769388649 |
| YTHDC1 | blue | 0.021628593 | 0.890505263 |
| YWHAH | blue | 0.493192989 | 0.000778512 |
| YY2 | blue | 0.162289327 | 0.298453257 |
| ZAN | blue | 0.095994066 | 0.54031075 |
| ZAR1 | blue | 0.274211162 | 0.075170955 |
| ZBBX | blue | -0.080229364 | 0.609055445 |
| ZBED1 | blue | -0.060231911 | 0.701217708 |
| ZBED6 | blue | 0.249372322 | 0.106820481 |
| ZBED6CL | blue | -0.120556599 | 0.441262847 |
| ZBP1 | blue | 0.204061601 | 0.18933404 |
| ZBTB18 | blue | -0.296388467 | 0.053620571 |
| ZBTB2 | blue | -0.041800266 | 0.790129889 |
| ZBTB32 | blue | 0.075948662 | 0.628347987 |
| ZBTB34 | lightcyan | 0.27649793 | 0.072675404 |
| ZBTB44 | blue | -0.209395772 | 0.177776199 |
| ZBTB46 | blue | 0.363342864 | 0.016625926 |
| ZBTB47 | blue | 0.11430864 | 0.465462464 |
| ZBTB48 | blue | -0.051745589 | 0.741747056 |
| ZBTB6 | blue | -0.09475878 | 0.54555956 |
| ZBTB8B | blue | -0.124907167 | 0.424830936 |
| ZBTB8OS | blue | -0.027448797 | 0.861297517 |
| ZBTB9 | blue | 0.023514791 | 0.881022097 |
| ZC2HC1B | blue | 0.155693391 | 0.318783007 |
| ZC2HC1C | blue | -0.218876752 | 0.158497606 |
| ZC3H12A | blue | 0.145929899 | 0.350437921 |
| ZC3H15 | blue | -0.067812426 | 0.665690183 |
| ZC3H6 | blue | -0.360436484 | 0.017581585 |
| ZC3H7A | blue | 0.034529172 | 0.826014185 |
| ZC3H8 | blue | -0.34543066 | 0.023284691 |
| ZC3HAV1 | lightyellow | 0.257857188 | 0.095033784 |
| ZCCHC2 | blue | 0.342121255 | 0.024731328 |
| ZCCHC24 | lightcyan | 0.39616268 | 0.008542696 |
| ZCCHC9 | blue | -0.055195027 | 0.725182584 |
| ZCWPW1 | blue | -0.171332097 | 0.27196833 |
| ZCWPW2 | blue | -0.160379382 | 0.304252274 |
| ZDHHC11 | blue | -0.018492502 | 0.906305264 |
| ZDHHC11B | blue | -0.006672282 | 0.966128625 |
| ZDHHC13 | blue | -0.071192379 | 0.650073816 |
| ZDHHC16 | blue | 0.025729453 | 0.869908557 |
| ZDHHC17 | blue | 0.177928459 | 0.253657274 |
| ZDHHC2 | blue | -0.250763608 | 0.104814922 |
| ZDHHC21 | blue | 0.099732763 | 0.524574548 |
| ZDHHC3 | blue | 0.222402 | 0.151733104 |
| ZEB1 | lightcyan | 0.516230406 | 0.000394792 |
| ZFAND1 | blue | 0.13222534 | 0.397982427 |
| ZFAND2B | blue | -0.033762423 | 0.829820083 |
| ZFP14 | blue | -0.125059668 | 0.424261267 |
| ZFP2 | blue | -0.066094667 | 0.673681087 |
| ZFP28 | blue | -0.258095635 | 0.094717676 |
| ZFP36 | blue | -0.311321222 | 0.042141168 |
| ZFP42 | blue | -0.150613771 | 0.335019923 |
| ZFP69B | blue | 0.113887713 | 0.467117922 |
| ZFP82 | blue | -0.20120647 | 0.19573495 |
| ZFP92 | blue | 0.074812321 | 0.633511359 |
| ZFYVE1 | blue | 0.153434721 | 0.325940518 |
| ZFYVE16 | blue | 0.110895876 | 0.478974539 |
| ZFYVE19 | blue | -0.379276581 | 0.012131423 |
| ZFYVE26 | blue | 0.498278585 | 0.000672879 |
| ZIC4 | lightcyan | -0.401766679 | 0.007574146 |
| ZIC5 | blue | 0.00233121 | 0.98816277 |
| ZIM3 | blue | -0.007122821 | 0.963842952 |
| ZKSCAN4 | blue | 0.246752913 | 0.11067568 |
| ZMAT3 | blue | -0.145900799 | 0.350535045 |
| ZMAT4 | blue | 0.079452666 | 0.612537038 |
| ZMIZ2 | blue | 0.063588191 | 0.685404735 |
| ZMYM1 | blue | 0.053457469 | 0.733511537 |
| ZMYM2 | blue | 0.251853777 | 0.10326364 |
| ZMYM3 | blue | -0.335654402 | 0.027774882 |
| ZMYND10 | blue | -0.264332625 | 0.08673398 |
| ZMYND15 | blue | -0.19955241 | 0.199512284 |
| ZMYND8 | blue | 0.17166812 | 0.271015027 |
| ZNF107 | blue | 0.20177608 | 0.194445908 |
| ZNF12 | blue | -0.091176658 | 0.560916505 |
| ZNF132 | blue | 0.018084011 | 0.908366054 |
| ZNF136 | blue | 0.093930746 | 0.549091512 |
| ZNF146 | blue | -0.125909747 | 0.421093672 |
| ZNF148 | blue | -0.073089331 | 0.64137321 |
| ZNF154 | blue | 0.339287211 | 0.026029027 |
| ZNF155 | blue | 0.314577592 | 0.039925225 |
| ZNF175 | blue | -0.264109437 | 0.087010341 |
| ZNF177 | blue | 0.123385823 | 0.430537457 |
| ZNF180 | blue | -0.349584764 | 0.02156956 |
| ZNF184 | blue | -0.336092121 | 0.027559506 |
| ZNF197 | blue | -0.036440279 | 0.816545354 |
| ZNF208 | blue | -0.086897887 | 0.579519779 |
| ZNF211 | blue | 0.152783418 | 0.328022973 |
| ZNF213 | blue | -0.099235874 | 0.526652855 |
| ZNF214 | blue | -0.295187773 | 0.054643269 |
| ZNF215 | blue | 0.010293634 | 0.947765937 |
| ZNF221 | blue | 0.215644906 | 0.164889877 |
| ZNF222 | blue | 0.201226488 | 0.195689548 |
| ZNF226 | blue | 0.140020542 | 0.370496833 |
| ZNF227 | blue | 0.189153536 | 0.224436278 |
| ZNF23 | blue | 0.036804686 | 0.814742732 |
| ZNF234 | blue | 0.157089058 | 0.3144102 |
| ZNF235 | blue | 0.125484387 | 0.422676991 |
| ZNF24 | blue | 0.098479 | 0.529826345 |
| ZNF253 | blue | -0.203960784 | 0.1895575 |
| ZNF256 | blue | -0.508609678 | 0.000496854 |
| ZNF263 | blue | -0.05321614 | 0.734670761 |
| ZNF266 | blue | 0.271279236 | 0.078468549 |
| ZNF273 | blue | -0.110868206 | 0.47908493 |
| ZNF280B | blue | 0.170478351 | 0.274400357 |
| ZNF284 | lightcyan | 0.313963908 | 0.040335393 |
| ZNF3 | blue | -0.23576258 | 0.128012047 |
| ZNF317 | blue | 0.11976469 | 0.444291095 |
| ZNF318 | blue | 0.193841237 | 0.212948028 |
| ZNF324B | blue | 0.24981168 | 0.106184002 |
| ZNF333 | lightcyan | 0.058634625 | 0.708787749 |
| ZNF334 | blue | -0.224202894 | 0.148360253 |
| ZNF335 | blue | 0.251813864 | 0.103320123 |
| ZNF33B | blue | -0.387488363 | 0.010251652 |
| ZNF347 | blue | -0.083955936 | 0.59247025 |
| ZNF354A | blue | -0.280682467 | 0.068278254 |
| ZNF365 | blue | 0.09228735 | 0.556133366 |
| ZNF366 | blue | 0.416155921 | 0.005509283 |
| ZNF367 | blue | -0.160202958 | 0.304791544 |
| ZNF384 | blue | -0.231509877 | 0.135239663 |
| ZNF391 | blue | -0.003322126 | 0.983131788 |
| ZNF395 | blue | -0.460168619 | 0.00190393 |
| ZNF396 | blue | -0.213593314 | 0.169043491 |
| ZNF404 | blue | -0.116482919 | 0.45696149 |
| ZNF407 | blue | -0.198365502 | 0.202254197 |
| ZNF410 | blue | 0.169550865 | 0.277058603 |
| ZNF418 | blue | 0.157878158 | 0.311954735 |
| ZNF420 | blue | -0.056570718 | 0.718610315 |
| ZNF423 | blue | -0.152734632 | 0.328179294 |
| ZNF43 | blue | 0.140301661 | 0.369527332 |
| ZNF436 | blue | -0.059385526 | 0.705225492 |
| ZNF438 | blue | 0.505374933 | 0.000546909 |
| ZNF44 | blue | 0.063156969 | 0.687429176 |
| ZNF449 | blue | 0.3313961 | 0.02994398 |
| ZNF454 | blue | -0.072507304 | 0.644037779 |
| ZNF460 | blue | 0.081460638 | 0.603553635 |
| ZNF470 | blue | -0.255236712 | 0.098561567 |
| ZNF484 | blue | 0.299545712 | 0.051004648 |
| ZNF485 | blue | 0.197813472 | 0.203538427 |
| ZNF488 | blue | -0.194583352 | 0.211167505 |
| ZNF490 | blue | 0.206588662 | 0.183793784 |
| ZNF497 | blue | 0.078721203 | 0.615823589 |
| ZNF501 | blue | -0.384218987 | 0.010967775 |
| ZNF502 | blue | -0.220818944 | 0.154744111 |
| ZNF503 | blue | -0.359557234 | 0.01787967 |
| ZNF507 | blue | 0.30545937 | 0.046382137 |
| ZNF511 | blue | -0.130519772 | 0.404150032 |
| ZNF512B | blue | 0.030892856 | 0.844097174 |
| ZNF516 | blue | 0.202600989 | 0.192589767 |
| ZNF517 | blue | 0.080534598 | 0.607689536 |
| ZNF518A | blue | -0.049494221 | 0.752621309 |
| ZNF519 | blue | -0.080321967 | 0.608640909 |
| ZNF525 | blue | -0.047609729 | 0.761760108 |
| ZNF526 | blue | 0.093704923 | 0.550056635 |
| ZNF528 | blue | -0.166862148 | 0.284859865 |
| ZNF529 | blue | -0.33320494 | 0.029006058 |
| ZNF530 | blue | -0.113232738 | 0.4697001 |
| ZNF532 | blue | 0.017829285 | 0.909651417 |
| ZNF536 | blue | -0.133536653 | 0.393277872 |
| ZNF543 | blue | -0.068881996 | 0.660732948 |
| ZNF544 | blue | 0.093731266 | 0.549944007 |
| ZNF548 | blue | 0.027746859 | 0.859806313 |
| ZNF549 | blue | 0.195079515 | 0.20998288 |
| ZNF550 | blue | 0.080718248 | 0.606868347 |
| ZNF563 | blue | 0.070542334 | 0.653066002 |
| ZNF565 | blue | 0.215449395 | 0.165282485 |
| ZNF566 | blue | -0.018042419 | 0.908575912 |
| ZNF568 | blue | -0.197015149 | 0.205405719 |
| ZNF570 | blue | -0.057091549 | 0.716127272 |
| ZNF572 | blue | -0.400649519 | 0.007759281 |
| ZNF574 | blue | -0.280537328 | 0.068427158 |
| ZNF578 | blue | -0.204995149 | 0.187273719 |
| ZNF579 | blue | -0.070297528 | 0.654194251 |
| ZNF580 | blue | -0.154709141 | 0.321889727 |
| ZNF584 | blue | 0.109175262 | 0.485864276 |
| ZNF585B | blue | -0.057937329 | 0.712101208 |
| ZNF589 | blue | 0.135623881 | 0.385856873 |
| ZNF592 | blue | 0.041077062 | 0.793681209 |
| ZNF593 | blue | -0.360870882 | 0.01743587 |
| ZNF595 | blue | -0.051063251 | 0.745037658 |
| ZNF597 | blue | -0.132359887 | 0.397498219 |
| ZNF600 | blue | 0.186377265 | 0.231437677 |
| ZNF606 | blue | -0.10020219 | 0.522614827 |
| ZNF608 | blue | -0.306735715 | 0.045430438 |
| ZNF611 | blue | -0.110532591 | 0.480424926 |
| ZNF613 | blue | 0.255858005 | 0.097716191 |
| ZNF630 | blue | -0.06188785 | 0.693399746 |
| ZNF641 | blue | -0.16379177 | 0.293941788 |
| ZNF644 | blue | -0.093942863 | 0.549039749 |
| ZNF649 | blue | -0.202233723 | 0.193414602 |
| ZNF660 | blue | 0.081869077 | 0.601733336 |
| ZNF675 | lightyellow | -0.149559527 | 0.338452945 |
| ZNF680 | blue | -0.252223364 | 0.102741736 |
| ZNF684 | blue | -0.035604597 | 0.820682761 |
| ZNF705B | blue | 0.058907997 | 0.707490162 |
| ZNF705D | blue | -0.112659569 | 0.471965983 |
| ZNF708 | blue | -0.098168162 | 0.531132361 |
| ZNF711 | blue | -0.250496137 | 0.105198233 |
| ZNF713 | blue | 0.205725377 | 0.185673269 |
| ZNF716 | blue | -0.247919196 | 0.108946305 |
| ZNF718 | blue | -0.285296658 | 0.063676603 |
| ZNF732 | blue | -0.065114118 | 0.678258551 |
| ZNF738 | blue | -0.058393335 | 0.709933729 |
| ZNF746 | blue | 0.067139421 | 0.668816665 |
| ZNF747 | blue | -0.107259957 | 0.493593881 |
| ZNF75A | blue | -0.282781826 | 0.066153002 |
| ZNF75D | blue | -0.032558178 | 0.835805308 |
| ZNF76 | blue | -0.072023524 | 0.646255922 |
| ZNF761 | blue | 0.21209986 | 0.172114276 |
| ZNF763 | blue | -0.630535059 | 5.85E-06 |
| ZNF766 | blue | -0.047972547 | 0.759998098 |
| ZNF768 | blue | -0.344527139 | 0.023672444 |
| ZNF770 | blue | -0.39331151 | 0.009075119 |
| ZNF773 | blue | -0.066807246 | 0.670361848 |
| ZNF774 | blue | 0.239520258 | 0.121869874 |
| ZNF776 | blue | 0.352849649 | 0.020297036 |
| ZNF781 | blue | 0.220216732 | 0.155900928 |
| ZNF783 | blue | 0.235774383 | 0.127992398 |
| ZNF787 | blue | -0.250232562 | 0.105577008 |
| ZNF792 | lightcyan | 0.438219133 | 0.003290996 |
| ZNF8 | blue | 0.151673057 | 0.331592318 |
| ZNF80 | blue | 0.175278174 | 0.260912344 |
| ZNF805 | blue | 0.024280705 | 0.877175953 |
| ZNF808 | blue | 0.097613261 | 0.53346773 |
| ZNF813 | blue | 0.021160089 | 0.892863133 |
| ZNF821 | blue | 0.109377474 | 0.485051903 |
| ZNF823 | blue | -0.18933326 | 0.223988116 |
| ZNF830 | blue | 0.036627209 | 0.815620541 |
| ZNF836 | blue | 0.117296211 | 0.453803413 |
| ZNF84 | blue | -0.104867717 | 0.503336534 |
| ZNF841 | blue | 0.018499865 | 0.906268124 |
| ZNF844 | blue | -0.35281003 | 0.020312092 |
| ZNF845 | blue | -0.016525815 | 0.916232312 |
| ZNF85 | blue | 0.100611009 | 0.520911085 |
| ZNF853 | blue | 0.299061906 | 0.051398692 |
| ZNF862 | blue | 0.360855116 | 0.01744114 |
| ZNF878 | lightcyan | -0.421189156 | 0.004912813 |
| ZNF891 | blue | 0.300948485 | 0.049875886 |
| ZNFX1 | blue | 0.340668679 | 0.025389551 |
| ZNRF1 | blue | -0.217012327 | 0.162162826 |
| ZRANB3 | blue | 0.089076247 | 0.570013873 |
| ZSCAN1 | blue | -0.20531079 | 0.186580725 |
| ZSCAN10 | blue | -0.160310539 | 0.304462631 |
| ZSCAN2 | blue | -0.128808175 | 0.410394444 |
| ZSCAN30 | blue | 0.113768269 | 0.467588252 |
| ZSCAN31 | blue | -0.001592227 | 0.991914964 |
| ZSWIM4 | blue | -0.352808688 | 0.020312602 |
| ZSWIM7 | blue | -0.268405283 | 0.081809899 |
| ZSWIM8 | blue | 0.020229755 | 0.897547972 |
| ZWINT | blue | 0.058842284 | 0.707802002 |
| ZXDA | blue | -0.187105714 | 0.229586339 |
| ZXDC | blue | 0.008877629 | 0.954943376 |
