# Supplementary material for: Investigating EGF and PAG1 as necroptosis-related biomarkers for diabetic nephropathy: an in silico and in vitro validation study
Source: Aging (Albany NY). 2023 Nov 20;15(22):13176–93. doi: 10.18632/aging.205233 (PMC10713428; doi:10.18632/aging.205233)
Supplement: Supplementary Table 4 [file aging-15-205233-s004.docx]

**Supplementary Table 4. Differentially expressed genes in bulk RNA-seq dataset (log2|fold change|>1 and an adjusted p-value<0.05).**

| **gene** | **Mean(control)** | **Mean(DN)** | **logFC** | **P-Value** | **adjusted P-value** |
| --- | --- | --- | --- | --- | --- |
| A1CF | 4.933002 | 6.492285 | -1.55928 | 1.29E-08 | 3.27E-07 |
| ABAT | 6.901317 | 8.197305 | -1.29599 | 1.12E-06 | 1.24E-05 |
| ABCC2 | 5.32817 | 6.400728 | -1.07256 | 5.66E-05 | 0.000319924 |
| ABCC9 | 5.501065 | 4.155665 | 1.345401 | 1.23E-06 | 1.34E-05 |
| ABI3BP | 9.141272 | 7.819932 | 1.32134 | 0.00018148 | 0.000852832 |
| ABRACL | 8.131205 | 9.148141 | -1.01694 | 5.51E-11 | 4.58E-09 |
| ACAA2 | 11.07073 | 12.19424 | -1.12351 | 9.47E-06 | 7.23E-05 |
| ACADSB | 9.925212 | 11.13071 | -1.20549 | 2.20E-05 | 0.000144659 |
| ACAP2-IT1 | 4.865473 | 3.471366 | 1.394108 | 1.37E-10 | 8.95E-09 |
| ACE2 | 7.438649 | 8.450494 | -1.01184 | 0.00488321 | 0.013372061 |
| ACMSD | 6.817857 | 8.631907 | -1.81405 | 1.68E-08 | 4.01E-07 |
| ACSM2A | 10.10532 | 11.7278 | -1.62248 | 0.00010268 | 0.000526844 |
| ACSM2B | 11.44919 | 13.20111 | -1.75191 | 6.11E-05 | 0.000340329 |
| ACY1 | 7.330637 | 8.780959 | -1.45032 | 2.32E-07 | 3.37E-06 |
| ACY3 | 6.45952 | 7.777211 | -1.31769 | 3.99E-07 | 5.31E-06 |
| ADAMTS1 | 9.183496 | 8.010065 | 1.173431 | 2.79E-06 | 2.66E-05 |
| ADAMTS5 | 8.084066 | 7.037273 | 1.046793 | 8.69E-06 | 6.72E-05 |
| ADH1B | 6.655259 | 5.012178 | 1.643081 | 2.01E-06 | 2.02E-05 |
| ADH6 | 5.565018 | 6.586194 | -1.02118 | 5.24E-05 | 0.000299255 |
| AEBP1 | 7.462414 | 6.170445 | 1.291969 | 2.94E-06 | 2.75E-05 |
| AFM | 7.482552 | 9.463995 | -1.98144 | 3.28E-05 | 0.00020171 |
| AGMAT | 8.85363 | 11.30845 | -2.45482 | 1.01E-06 | 1.14E-05 |
| AGPAT9 | 6.034474 | 7.051009 | -1.01653 | 3.23E-06 | 2.96E-05 |
| AGXT2 | 8.722707 | 10.19464 | -1.47193 | 0.00175409 | 0.005672939 |
| AK4 | 8.73033 | 10.24979 | -1.51946 | 3.17E-08 | 6.78E-07 |
| AK4P3 | 8.645237 | 11.18326 | -2.53802 | 4.34E-09 | 1.36E-07 |
| AKR7A2 | 6.978803 | 8.02076 | -1.04196 | 1.37E-10 | 8.95E-09 |
| AKR7A3 | 5.336344 | 6.811152 | -1.47481 | 3.22E-10 | 1.73E-08 |
| ALB | 6.591741 | 9.414068 | -2.82233 | 1.48E-07 | 2.34E-06 |
| ALDH2 | 8.942753 | 10.08742 | -1.14466 | 5.27E-10 | 2.58E-08 |
| ALDH4A1 | 6.921708 | 8.39443 | -1.47272 | 7.41E-08 | 1.32E-06 |
| ALDH6A1 | 9.800851 | 11.60135 | -1.8005 | 1.18E-07 | 1.93E-06 |
| ALDOB | 15.85531 | 17.83628 | -1.98097 | 7.56E-09 | 2.15E-07 |
| ALKBH7 | 9.132979 | 10.24515 | -1.11217 | 1.63E-13 | 6.70E-11 |
| ALPL | 5.488998 | 6.782604 | -1.29361 | 3.17E-08 | 6.78E-07 |
| ANGPT2 | 6.049924 | 5.007451 | 1.042473 | 9.57E-11 | 6.87E-09 |
| ANGPTL3 | 6.647558 | 8.219161 | -1.5716 | 3.55E-05 | 0.00021589 |
| ANKRD10-IT1 | 7.327883 | 6.258085 | 1.069798 | 1.58E-05 | 0.000110167 |
| ANKRD36 | 8.392613 | 7.247365 | 1.145248 | 7.98E-11 | 6.07E-09 |
| ANKRD36B | 7.939958 | 6.865365 | 1.074593 | 3.22E-10 | 1.73E-08 |
| ANKS4B | 3.796136 | 5.036582 | -1.24045 | 2.08E-07 | 3.08E-06 |
| ANPEP | 12.33055 | 13.42027 | -1.08971 | 0.00597436 | 0.015873028 |
| ANXA9 | 6.059356 | 7.11705 | -1.05769 | 4.34E-09 | 1.36E-07 |
| AOX1 | 7.833202 | 8.854905 | -1.0217 | 0.00035706 | 0.001505577 |
| APLN | 8.320649 | 9.449012 | -1.12836 | 6.07E-07 | 7.50E-06 |
| APOH | 4.990181 | 6.437291 | -1.44711 | 1.45E-05 | 0.000102838 |
| APOM | 9.281843 | 11.11505 | -1.83321 | 3.84E-05 | 0.00023039 |
| AQP7P3 | 5.955815 | 7.030302 | -1.07449 | 3.23E-06 | 2.96E-05 |
| AQP9 | 4.583091 | 5.7598 | -1.17671 | 6.58E-08 | 1.20E-06 |
| ARHGAP19 | 9.933519 | 11.16397 | -1.23045 | 2.31E-06 | 2.27E-05 |
| AS3MT | 7.611751 | 8.618652 | -1.0069 | 1.13E-08 | 2.95E-07 |
| ASPA | 7.617845 | 8.898347 | -1.2805 | 1.48E-07 | 2.34E-06 |
| ASPN | 6.806064 | 5.086278 | 1.719786 | 2.17E-08 | 4.94E-07 |
| ASS1 | 12.3591 | 13.57662 | -1.21752 | 2.59E-07 | 3.69E-06 |
| ATP13A3 | 11.67037 | 10.42963 | 1.240737 | 7.26E-10 | 3.35E-08 |
| ATP5D | 11.07805 | 12.12841 | -1.05036 | 4.56E-11 | 3.95E-09 |
| ATP6V0D2 | 5.975014 | 6.992307 | -1.01729 | 0.00063612 | 0.002434007 |
| ATP6V1G3 | 5.792491 | 7.043371 | -1.25088 | 0.00304852 | 0.008997465 |
| BCL6B | 7.538362 | 6.493221 | 1.045141 | 4.06E-08 | 8.15E-07 |
| BHMT | 11.0536 | 12.8337 | -1.7801 | 2.80E-05 | 0.000176873 |
| BHMT2 | 10.90633 | 12.231 | -1.32467 | 4.49E-05 | 0.000263836 |
| BLVRB | 7.473879 | 8.724447 | -1.25057 | 1.63E-13 | 6.70E-11 |
| BRE-AS1 | 5.195884 | 7.592276 | -2.39639 | 1.33E-07 | 2.12E-06 |
| BST1 | 8.412367 | 9.441177 | -1.02881 | 2.20E-05 | 0.000144659 |
| BTG2 | 9.977045 | 11.4427 | -1.46566 | 1.63E-10 | 1.02E-08 |
| C11orf54 | 11.72446 | 12.87754 | -1.15308 | 6.11E-06 | 5.02E-05 |
| C14orf164 | 5.07255 | 6.326712 | -1.25416 | 4.44E-07 | 5.80E-06 |
| C15orf59 | 9.359475 | 10.36696 | -1.00749 | 0.00040698 | 0.001679168 |
| C19orf77 | 12.26415 | 13.80334 | -1.53919 | 0.00067706 | 0.002568074 |
| C1QC | 8.175081 | 6.608828 | 1.566253 | 0.00018148 | 0.000852832 |
| C1orf192 | 6.958678 | 7.96521 | -1.00653 | 1.16E-09 | 4.86E-08 |
| C3 | 7.104494 | 5.140221 | 1.964273 | 0.00232153 | 0.00714774 |
| C7 | 10.99768 | 9.022721 | 1.974962 | 0.00014702 | 0.000714231 |
| C9orf66 | 4.183311 | 5.219618 | -1.03631 | 9.15E-07 | 1.05E-05 |
| CA2 | 8.453206 | 10.0094 | -1.55619 | 2.08E-11 | 2.32E-09 |
| CACNA1C-AS4 | 5.711049 | 4.578651 | 1.132398 | 9.91E-09 | 2.67E-07 |
| CALB1 | 6.653608 | 8.069743 | -1.41613 | 0.00033426 | 0.001425893 |
| CCL11 | 5.809144 | 4.586004 | 1.22314 | 0.00010268 | 0.000526844 |
| CCL18 | 5.238753 | 3.261189 | 1.977564 | 0.00011868 | 0.000596667 |
| CCL19 | 7.112508 | 5.322353 | 1.790155 | 2.20E-05 | 0.000144659 |
| CCL2 | 13.75796 | 12.26643 | 1.491532 | 0.00052643 | 0.002081037 |
| CCL21 | 8.050048 | 5.36252 | 2.687528 | 1.48E-07 | 2.34E-06 |
| CCND2 | 7.411396 | 6.408352 | 1.003044 | 4.93E-07 | 6.31E-06 |
| CD248 | 12.19904 | 11.16663 | 1.032411 | 0.00110152 | 0.003855402 |
| CDH10 | 3.998659 | 5.675494 | -1.67683 | 1.35E-09 | 5.48E-08 |
| CDH11 | 7.254828 | 6.240288 | 1.014541 | 0.00015777 | 0.000758621 |
| CDH6 | 8.844389 | 7.23235 | 1.612039 | 0.00033426 | 0.001425893 |
| CERS6 | 12.05007 | 13.24265 | -1.19258 | 2.83E-09 | 9.73E-08 |
| CETP | 6.645079 | 7.6987 | -1.05362 | 1.45E-05 | 0.000102838 |
| CFH | 11.88965 | 10.7466 | 1.143047 | 0.00061234 | 0.002364652 |
| CHI3L1 | 11.8805 | 13.25144 | -1.37094 | 0.00052643 | 0.002081037 |
| CKB | 6.043865 | 7.272801 | -1.22894 | 2.97E-12 | 5.19E-10 |
| CLDN8 | 5.29553 | 6.334237 | -1.03871 | 0.00232153 | 0.00714774 |
| CLEC4E | 3.949504 | 5.275858 | -1.32635 | 2.11E-09 | 7.92E-08 |
| CLYBL | 6.819381 | 7.858307 | -1.03893 | 5.18E-08 | 9.93E-07 |
| CMBL | 5.302153 | 6.449925 | -1.14777 | 4.93E-07 | 6.31E-06 |
| CNTN1 | 8.127153 | 9.189497 | -1.06234 | 6.74E-07 | 8.13E-06 |
| COL14A1 | 6.048872 | 4.883525 | 1.165347 | 1.53E-05 | 0.000107726 |
| COL15A1 | 5.675812 | 4.558923 | 1.116889 | 7.56E-09 | 2.15E-07 |
| COL1A2 | 5.996208 | 4.427754 | 1.568455 | 2.17E-08 | 4.94E-07 |
| COL3A1 | 6.488376 | 5.308911 | 1.179465 | 0.00052643 | 0.002081037 |
| COL6A3 | 5.431099 | 4.024918 | 1.406181 | 4.34E-09 | 1.36E-07 |
| COL8A1 | 6.202471 | 4.543055 | 1.659416 | 3.23E-06 | 2.96E-05 |
| COLEC12 | 7.198341 | 5.560787 | 1.637554 | 0.0004632 | 0.001872224 |
| CPA3 | 4.687155 | 3.516113 | 1.171043 | 0.00052643 | 0.002081037 |
| CPXM1 | 6.725712 | 8.221834 | -1.49612 | 1.33E-05 | 9.58E-05 |
| CR1 | 11.08892 | 12.63359 | -1.54467 | 4.44E-07 | 5.80E-06 |
| CRYAA | 7.052969 | 8.342552 | -1.28958 | 7.46E-07 | 8.89E-06 |
| CRYL1 | 7.26496 | 8.525748 | -1.26079 | 4.34E-09 | 1.36E-07 |
| CTH | 4.711534 | 5.805279 | -1.09374 | 1.13E-08 | 2.95E-07 |
| CTHRC1 | 5.634222 | 4.61278 | 1.021443 | 1.94E-10 | 1.16E-08 |
| CTSL2 | 6.813765 | 8.043962 | -1.2302 | 6.67E-06 | 5.40E-05 |
| CTXN3 | 6.468321 | 8.336936 | -1.86861 | 6.07E-07 | 7.50E-06 |
| CUBN | 11.13731 | 12.60504 | -1.46773 | 0.00627873 | 0.016548042 |
| CX3CL1 | 11.73246 | 12.86132 | -1.12885 | 7.56E-09 | 2.15E-07 |
| CXCR1 | 4.073198 | 6.017135 | -1.94394 | 2.19E-13 | 8.15E-11 |
| CXCR2 | 4.515519 | 6.430611 | -1.91509 | 3.75E-12 | 6.25E-10 |
| CYB5A | 13.47462 | 14.76435 | -1.28973 | 7.98E-11 | 6.07E-09 |
| CYP2B6 | 5.404723 | 7.158453 | -1.75373 | 5.84E-08 | 1.10E-06 |
| CYP4A11 | 8.413674 | 10.33986 | -1.92619 | 0.00067706 | 0.002568074 |
| CYP4A22 | 5.720901 | 7.207879 | -1.48698 | 0.00033426 | 0.001425893 |
| DACH1 | 13.30989 | 14.57785 | -1.26796 | 3.33E-08 | 7.10E-07 |
| DANCR | 5.814249 | 7.20927 | -1.39502 | 3.11E-14 | 2.31E-11 |
| DAO | 6.655529 | 7.992121 | -1.33659 | 8.87E-05 | 0.000464919 |
| DAPL1 | 9.382265 | 8.278827 | 1.103439 | 0.00259123 | 0.007827385 |
| DCXR | 11.25304 | 13.24821 | -1.99517 | 2.35E-12 | 4.51E-10 |
| DDC | 7.446999 | 8.876083 | -1.42908 | 1.72E-05 | 0.000117882 |
| DEFA1B | 6.902221 | 9.534665 | -2.63244 | 0.01278841 | 0.02980855 |
| DEFB1 | 15.78666 | 17.39946 | -1.6128 | 0.00010268 | 0.000526844 |
| DPEP1 | 6.157081 | 7.360627 | -1.20355 | 2.68E-06 | 2.56E-05 |
| DPP6 | 8.356123 | 9.723758 | -1.36764 | 3.17E-08 | 6.78E-07 |
| DPYS | 5.990849 | 7.089051 | -1.0982 | 4.85E-05 | 0.000281163 |
| DUSP1 | 6.75986 | 8.712971 | -1.95311 | 3.21E-16 | 1.48E-12 |
| ECH1 | 10.4959 | 11.86219 | -1.36629 | 3.10E-11 | 3.00E-09 |
| ECHS1 | 9.955304 | 11.49894 | -1.54363 | 2.73E-10 | 1.52E-08 |
| ECM1 | 9.294539 | 7.589863 | 1.704676 | 5.47E-07 | 6.91E-06 |
| EGF | 5.077572 | 6.584545 | -1.50697 | 1.66E-07 | 2.57E-06 |
| EGOT | 5.719349 | 6.841973 | -1.12262 | 2.58E-05 | 0.000165515 |
| EGR1 | 8.884807 | 11.81876 | -2.93396 | 6.74E-07 | 8.13E-06 |
| EHD3 | 10.76441 | 12.41442 | -1.65001 | 8.34E-08 | 1.46E-06 |
| EHHADH | 6.811338 | 7.996909 | -1.18557 | 6.67E-06 | 5.40E-05 |
| EMP1 | 10.03188 | 8.429853 | 1.602024 | 6.60E-09 | 1.92E-07 |
| ENPEP | 14.0364 | 15.06402 | -1.02762 | 3.76E-11 | 3.45E-09 |
| EPHX2 | 7.124172 | 8.22097 | -1.0968 | 1.33E-07 | 2.12E-06 |
| ERRFI1 | 9.930238 | 11.91424 | -1.984 | 2.73E-10 | 1.52E-08 |
| ESM1 | 6.966904 | 9.426317 | -2.45941 | 4.59E-08 | 8.99E-07 |
| ETFB | 9.156315 | 10.30097 | -1.14465 | 3.10E-11 | 3.00E-09 |
| EXPH5 | 11.0041 | 12.06874 | -1.06464 | 0.01309318 | 0.030432679 |
| F2RL2 | 7.215534 | 6.105809 | 1.109725 | 1.83E-06 | 1.87E-05 |
| FABP1 | 8.316268 | 10.00861 | -1.69234 | 1.05E-07 | 1.77E-06 |
| FABP3 | 8.714144 | 10.10691 | -1.39277 | 1.12E-06 | 1.24E-05 |
| FABP5P1 | 11.30037 | 9.614891 | 1.685484 | 2.80E-05 | 0.000176873 |
| FABP5P7 | 11.48685 | 9.748398 | 1.738455 | 2.80E-05 | 0.000176873 |
| FAM151A | 7.923265 | 9.726718 | -1.80345 | 1.23E-05 | 8.94E-05 |
| FAM180A | 6.993359 | 8.775552 | -1.78219 | 3.59E-08 | 7.44E-07 |
| FBLN5 | 8.990448 | 7.386775 | 1.603673 | 9.55E-05 | 0.000494487 |
| FBN1 | 7.009005 | 5.789927 | 1.219078 | 1.16E-09 | 4.86E-08 |
| FBP1 | 7.206089 | 9.099714 | -1.89362 | 1.18E-07 | 1.93E-06 |
| FCGR3B | 6.319002 | 7.839082 | -1.52008 | 1.57E-09 | 6.15E-08 |
| FMN2 | 7.465925 | 8.752308 | -1.28638 | 5.75E-09 | 1.70E-07 |
| FMO1 | 8.813158 | 10.12488 | -1.31172 | 0.00196406 | 0.006214981 |
| FMO3 | 6.251451 | 5.014549 | 1.236902 | 5.24E-05 | 0.000299255 |
| FMO4 | 7.902696 | 8.927337 | -1.02464 | 0.0020773 | 0.006509794 |
| FN1 | 10.62167 | 6.710086 | 3.911587 | 1.13E-08 | 2.95E-07 |
| FOLH1B | 5.607559 | 6.883003 | -1.27544 | 9.47E-06 | 7.23E-05 |
| FOS | 5.014739 | 8.71527 | -3.70053 | 3.21E-16 | 1.48E-12 |
| FPR1 | 5.285803 | 7.175867 | -1.89006 | 1.05E-07 | 1.77E-06 |
| FPR3 | 6.049732 | 4.242102 | 1.80763 | 2.20E-05 | 0.000144659 |
| FRY-AS1 | 6.454839 | 7.799179 | -1.34434 | 5.84E-08 | 1.10E-06 |
| FTLP3 | 16.79399 | 17.85788 | -1.06389 | 3.89E-13 | 1.18E-10 |
| FUT6 | 6.071664 | 7.124552 | -1.05289 | 8.12E-05 | 0.000434745 |
| FXYD1 | 6.996793 | 8.130083 | -1.13329 | 6.58E-08 | 1.20E-06 |
| G0S2 | 8.032186 | 9.527813 | -1.49563 | 4.06E-08 | 8.15E-07 |
| G6PC | 7.377587 | 12.07274 | -4.69516 | 6.25E-14 | 4.00E-11 |
| GADD45B | 7.488835 | 8.552148 | -1.06331 | 7.29E-06 | 5.80E-05 |
| GBA3 | 6.605976 | 8.118515 | -1.51254 | 2.68E-06 | 2.56E-05 |
| GDF15 | 4.003446 | 5.344587 | -1.34114 | 6.64E-11 | 5.31E-09 |
| GIPC2 | 7.513032 | 9.157878 | -1.64485 | 3.22E-07 | 4.45E-06 |
| GK | 7.964956 | 9.023205 | -1.05825 | 0.00027961 | 0.001229189 |
| GLYAT | 10.30158 | 12.67888 | -2.3773 | 9.15E-07 | 1.05E-05 |
| GLYATL1 | 7.963448 | 10.01052 | -2.04707 | 3.54E-06 | 3.20E-05 |
| GOLGA8A | 9.554839 | 8.438349 | 1.116491 | 9.76E-08 | 1.67E-06 |
| GOLGA8B | 9.59481 | 8.531066 | 1.063744 | 3.17E-08 | 6.78E-07 |
| GPR18 | 5.733352 | 4.661876 | 1.071476 | 1.16E-09 | 4.86E-08 |
| GPR34 | 5.691343 | 4.449629 | 1.241714 | 8.87E-05 | 0.000464919 |
| GSTA1 | 12.41498 | 15.01897 | -2.60399 | 1.13E-08 | 2.95E-07 |
| GSTA2 | 10.77065 | 13.94744 | -3.17679 | 2.73E-10 | 1.52E-08 |
| GSTA3 | 4.548634 | 5.710237 | -1.1616 | 2.30E-10 | 1.32E-08 |
| GSTA5 | 4.661308 | 5.73798 | -1.07667 | 5.94E-07 | 7.47E-06 |
| GUCY1A3 | 8.397647 | 7.341224 | 1.056423 | 2.97E-12 | 5.19E-10 |
| GUSBP2 | 8.520157 | 7.50331 | 1.016847 | 4.48E-10 | 2.25E-08 |
| GUSBP3 | 7.691457 | 6.395792 | 1.295666 | 7.98E-11 | 6.07E-09 |
| GUSBP9 | 8.319436 | 6.984728 | 1.334708 | 3.22E-10 | 1.73E-08 |
| HAO2 | 7.82165 | 10.17732 | -2.35567 | 7.96E-06 | 6.26E-05 |
| HBA1 | 11.66794 | 15.0739 | -3.40596 | 1.49E-09 | 6.01E-08 |
| HBA2 | 12.18211 | 14.40954 | -2.22743 | 4.59E-09 | 1.44E-07 |
| HBB | 10.05054 | 12.5556 | -2.50506 | 3.21E-16 | 1.48E-12 |
| HES1 | 8.949595 | 10.48515 | -1.53556 | 1.94E-10 | 1.16E-08 |
| HGD | 7.693238 | 9.103066 | -1.40983 | 8.69E-06 | 6.72E-05 |
| HIST1H1C | 10.45424 | 11.51998 | -1.06574 | 6.60E-09 | 1.92E-07 |
| HIST1H1E | 12.52024 | 14.15336 | -1.63312 | 2.24E-15 | 4.31E-12 |
| HIST1H2BD | 6.33503 | 7.875321 | -1.54029 | 2.35E-12 | 4.51E-10 |
| HIST2H2AA4 | 8.781882 | 11.12161 | -2.33973 | 4.46E-14 | 3.11E-11 |
| HIST2H2BE | 8.225912 | 9.472695 | -1.24678 | 2.83E-09 | 9.73E-08 |
| HNRNPU-AS1 | 8.525506 | 7.24559 | 1.279917 | 1.57E-09 | 6.15E-08 |
| HPD | 8.411547 | 11.38408 | -2.97253 | 8.49E-10 | 3.80E-08 |
| HPGD | 8.058551 | 9.63711 | -1.57856 | 4.85E-05 | 0.000281163 |
| HRSP12 | 9.82894 | 11.4675 | -1.63856 | 3.22E-07 | 4.45E-06 |
| HSD11B2 | 5.323188 | 6.323672 | -1.00048 | 0.00175409 | 0.005672939 |
| HSD17B14 | 5.652324 | 7.55203 | -1.89971 | 1.20E-13 | 5.62E-11 |
| HSPA1A | 7.77684 | 9.503216 | -1.72638 | 9.62E-15 | 1.23E-11 |
| HSPA1B | 8.695588 | 10.38104 | -1.68545 | 1.44E-14 | 1.58E-11 |
| HTRA1 | 14.37704 | 15.49156 | -1.11452 | 4.48E-10 | 2.25E-08 |
| IGF1 | 8.847907 | 10.36029 | -1.51239 | 0.00288865 | 0.00860504 |
| IGFBP6 | 7.13674 | 6.007035 | 1.129705 | 5.58E-06 | 4.67E-05 |
| IGHA1 | 11.11192 | 8.141677 | 2.970245 | 0.00488321 | 0.013372061 |
| IGHG4 | 8.871307 | 6.017741 | 2.853567 | 1.13E-05 | 8.34E-05 |
| IGHJ1 | 5.078483 | 3.868347 | 1.210136 | 3.27E-09 | 1.09E-07 |
| IGHJ5 | 4.663387 | 2.714298 | 1.949089 | 0.0004632 | 0.001872224 |
| IGHV3-21 | 4.142068 | 3.086212 | 1.055856 | 2.20E-05 | 0.000144659 |
| IGHV3-23 | 6.813301 | 5.514162 | 1.299139 | 0.02254376 | 0.04762962 |
| IGHV3-30 | 6.211883 | 4.87704 | 1.334844 | 0.00692878 | 0.017965205 |
| IGJ | 7.748238 | 6.217692 | 1.530546 | 0.00801499 | 0.02025377 |
| IGKC | 6.53364 | 5.153179 | 1.380462 | 0.00035706 | 0.001505577 |
| IGKV1-17 | 6.366118 | 5.205972 | 1.160146 | 0.00259123 | 0.007827385 |
| IGKV1-27 | 5.22018 | 4.142737 | 1.077443 | 0.00840889 | 0.021056192 |
| IGKV1-5 | 6.418232 | 5.323204 | 1.095028 | 0.00043426 | 0.001774396 |
| IGKV1-6 | 7.079511 | 5.58545 | 1.494061 | 0.00025577 | 0.001135873 |
| IGKV1-9 | 6.431413 | 5.277047 | 1.154366 | 0.00038127 | 0.001590491 |
| IGKV1D-16 | 6.867225 | 5.291384 | 1.575841 | 3.28E-05 | 0.00020171 |
| IGKV1D-27 | 7.196517 | 5.334986 | 1.861531 | 0.00076624 | 0.002842055 |
| IGKV1D-33 | 5.772658 | 4.410663 | 1.361996 | 1.03E-05 | 7.78E-05 |
| IGKV1D-39 | 7.468775 | 5.622141 | 1.846634 | 0.00131472 | 0.004462578 |
| IGKV1D-42 | 6.722655 | 5.560311 | 1.162345 | 0.00339216 | 0.009853839 |
| IGKV2-28 | 6.964882 | 5.231603 | 1.733279 | 0.02161588 | 0.045966174 |
| IGKV2D-28 | 6.569895 | 4.983207 | 1.586688 | 0.02161588 | 0.045966174 |
| IGKV2D-29 | 4.415436 | 3.352203 | 1.063233 | 0.01114815 | 0.026585383 |
| IGKV3-11 | 6.543482 | 5.261279 | 1.282203 | 0.00339216 | 0.009853839 |
| IGKV3-7 | 5.62461 | 4.589169 | 1.035441 | 0.00038127 | 0.001590491 |
| IGKV3D-15 | 5.841955 | 4.371334 | 1.470621 | 0.00165687 | 0.00540179 |
| IGKV3D-7 | 7.514538 | 5.770862 | 1.743677 | 0.00219637 | 0.00682811 |
| IGLC7 | 9.305722 | 6.452117 | 2.853605 | 0.00020842 | 0.000953928 |
| IGLV6-57 | 5.478789 | 4.389398 | 1.089391 | 0.00339216 | 0.009853839 |
| INHBA | 6.371392 | 4.846441 | 1.524952 | 3.54E-06 | 3.20E-05 |
| ITGA11 | 6.975607 | 5.514117 | 1.461491 | 1.13E-05 | 8.34E-05 |
| ITGB6 | 5.817834 | 4.656537 | 1.161297 | 0.00043426 | 0.001774396 |
| JUN | 5.860061 | 7.104582 | -1.24452 | 5.12E-13 | 1.49E-10 |
| JUNB | 6.562258 | 8.731012 | -2.16875 | 1.63E-13 | 6.70E-11 |
| KCNJ15 | 8.508731 | 9.771097 | -1.26237 | 8.87E-05 | 0.000464919 |
| KDR | 12.43473 | 13.61407 | -1.17934 | 2.80E-05 | 0.000176873 |
| KHK | 5.451245 | 6.54451 | -1.09326 | 6.67E-06 | 5.40E-05 |
| KIAA1191 | 11.34454 | 12.66108 | -1.31653 | 1.20E-13 | 5.62E-11 |
| KIRREL-IT1 | 7.347434 | 5.926499 | 1.420935 | 9.56E-09 | 2.62E-07 |
| KL | 10.4224 | 11.42401 | -1.00161 | 6.11E-05 | 0.000340329 |
| KLF7-IT1 | 7.623568 | 6.48951 | 1.134059 | 1.05E-07 | 1.77E-06 |
| KLK1 | 5.116716 | 6.14292 | -1.0262 | 0.00165687 | 0.00540179 |
| KLK7 | 11.48455 | 12.52602 | -1.04147 | 0.00059743 | 0.002308631 |
| KNG1 | 9.936823 | 12.02699 | -2.09017 | 9.55E-05 | 0.000494487 |
| L3MBTL3 | 7.805288 | 8.901178 | -1.09589 | 7.29E-06 | 5.80E-05 |
| LINC00052 | 5.171605 | 6.278397 | -1.10679 | 0.00012751 | 0.000634177 |
| LINC00342 | 8.31628 | 6.963268 | 1.353012 | 6.64E-11 | 5.31E-09 |
| LINC00839 | 9.410622 | 10.43598 | -1.02536 | 2.20E-05 | 0.000144659 |
| LINC00948 | 5.775588 | 7.199057 | -1.42347 | 2.94E-06 | 2.75E-05 |
| LOC100131825 | 5.258754 | 4.146743 | 1.112011 | 0.00076624 | 0.002842055 |
| LOC100190986 | 9.987563 | 7.644604 | 2.34296 | 8.70E-13 | 2.09E-10 |
| LOC100216546 | 5.123678 | 4.116114 | 1.007564 | 1.57E-09 | 6.15E-08 |
| LOC100272216 | 6.283663 | 5.180145 | 1.103518 | 1.15E-10 | 7.72E-09 |
| LOC100287497 | 7.001313 | 5.87639 | 1.124923 | 5.47E-07 | 6.91E-06 |
| LOC100303749 | 5.847701 | 4.615876 | 1.231825 | 1.18E-07 | 1.93E-06 |
| LOC100505664 | 6.059236 | 7.224851 | -1.16561 | 1.13E-05 | 8.34E-05 |
| LOC100505985 | 6.301819 | 8.311331 | -2.00951 | 2.02E-05 | 0.000135182 |
| LOC100506060 | 7.723942 | 6.444552 | 1.27939 | 6.25E-14 | 4.00E-11 |
| LOC100506123 | 7.751403 | 6.4286 | 1.322803 | 9.09E-12 | 1.25E-09 |
| LOC100996266 | 6.34314 | 8.151493 | -1.80835 | 7.46E-07 | 8.89E-06 |
| LOC100996472 | 6.315411 | 5.314635 | 1.000776 | 3.75E-12 | 6.25E-10 |
| LOC100996862 | 8.693815 | 7.541406 | 1.152409 | 2.73E-10 | 1.52E-08 |
| LOC101060277 | 4.606987 | 6.091991 | -1.485 | 0.00012751 | 0.000634177 |
| LOC440354 | 10.44888 | 9.126821 | 1.322063 | 1.20E-13 | 5.62E-11 |
| LOC550643 | 10.78751 | 11.80482 | -1.01731 | 2.93E-13 | 9.65E-11 |
| LOC595101 | 10.38535 | 9.039945 | 1.3454 | 8.70E-13 | 2.09E-10 |
| LOC613037 | 14.87886 | 13.66381 | 1.215051 | 3.76E-11 | 3.45E-09 |
| LOC642799 | 12.90908 | 11.90837 | 1.000713 | 1.49E-09 | 6.01E-08 |
| LOC645638 | 5.467641 | 4.371713 | 1.095928 | 2.08E-07 | 3.08E-06 |
| LOC727944 | 5.571655 | 7.95926 | -2.3876 | 6.19E-10 | 2.97E-08 |
| LOC728290 | 4.766659 | 5.96553 | -1.19887 | 1.03E-05 | 7.78E-05 |
| LOX | 9.380116 | 11.16416 | -1.78404 | 1.35E-09 | 5.48E-08 |
| LPL | 6.025961 | 7.332639 | -1.30668 | 1.47E-08 | 3.65E-07 |
| LRP2BP | 7.236685 | 6.057454 | 1.179232 | 7.26E-10 | 3.35E-08 |
| LRRC2 | 9.427995 | 10.77365 | -1.34565 | 5.18E-08 | 9.93E-07 |
| LTBP1 | 7.966442 | 6.822123 | 1.144319 | 4.59E-08 | 8.99E-07 |
| LUM | 9.176469 | 6.142507 | 3.033962 | 2.08E-07 | 3.08E-06 |
| LUST | 8.484071 | 7.100586 | 1.383485 | 1.94E-10 | 1.16E-08 |
| LYVE1 | 6.850931 | 5.434377 | 1.416554 | 0.00513798 | 0.013970044 |
| MAGI2-AS1 | 9.041811 | 7.225099 | 1.816712 | 3.22E-07 | 4.45E-06 |
| MAOA | 7.403848 | 8.613064 | -1.20922 | 9.57E-11 | 6.87E-09 |
| MARCKS | 10.26158 | 8.730023 | 1.531562 | 2.20E-05 | 0.000144659 |
| MED11 | 6.42158 | 7.491453 | -1.06987 | 1.12E-11 | 1.48E-09 |
| METTL7B | 7.099206 | 8.331876 | -1.23267 | 0.00056091 | 0.002195856 |
| MFAP4 | 7.846165 | 6.487207 | 1.358958 | 7.29E-06 | 5.80E-05 |
| MIOX | 9.007013 | 11.66892 | -2.66191 | 2.89E-07 | 4.06E-06 |
| MIR103A2 | 7.116396 | 5.474071 | 1.642325 | 7.98E-11 | 6.07E-09 |
| MIR103B2 | 7.676831 | 6.083844 | 1.592987 | 7.26E-10 | 3.35E-08 |
| MIR1254-1 | 9.873334 | 8.277606 | 1.595728 | 2.93E-13 | 9.65E-11 |
| MIR1256 | 5.530258 | 4.332759 | 1.197499 | 2.93E-13 | 9.65E-11 |
| MIR1285-2 | 12.20575 | 11.0652 | 1.140546 | 2.55E-11 | 2.61E-09 |
| MIR1299 | 9.640033 | 8.411429 | 1.228605 | 3.59E-07 | 4.86E-06 |
| MIR186 | 6.997476 | 5.851385 | 1.146091 | 2.59E-07 | 3.69E-06 |
| MIR297 | 6.020291 | 4.426521 | 1.59377 | 1.20E-13 | 5.62E-11 |
| MIR30A | 3.909497 | 2.658287 | 1.25121 | 3.89E-13 | 1.18E-10 |
| MIR30E | 4.101884 | 3.012693 | 1.089191 | 2.45E-09 | 8.82E-08 |
| MIR3120 | 6.161818 | 4.960829 | 1.20099 | 1.18E-07 | 1.93E-06 |
| MIR3671 | 7.631757 | 6.490018 | 1.14174 | 4.06E-08 | 8.15E-07 |
| MIR3911 | 7.769013 | 5.935885 | 1.833128 | 1.68E-08 | 4.01E-07 |
| MIR3916 | 8.002959 | 6.557277 | 1.445683 | 1.03E-05 | 7.78E-05 |
| MIR3975 | 5.782357 | 4.209106 | 1.573251 | 8.69E-06 | 6.72E-05 |
| MIR421 | 7.142993 | 5.744809 | 1.398184 | 1.86E-07 | 2.80E-06 |
| MIR4256 | 7.72837 | 5.055988 | 2.672382 | 9.62E-15 | 1.23E-11 |
| MIR4263 | 6.786758 | 5.745579 | 1.04118 | 1.66E-06 | 1.72E-05 |
| MIR4275 | 7.290993 | 6.236646 | 1.054348 | 3.84E-05 | 0.00023039 |
| MIR450B | 5.31878 | 3.849224 | 1.469556 | 1.15E-10 | 7.72E-09 |
| MIR4521 | 7.526085 | 4.215288 | 3.310797 | 3.21E-16 | 1.48E-12 |
| MIR503 | 4.543435 | 3.153635 | 1.3898 | 2.93E-13 | 9.65E-11 |
| MIR5047 | 11.65564 | 9.759536 | 1.896104 | 5.84E-08 | 1.10E-06 |
| MIR548A2 | 8.35898 | 7.317325 | 1.041655 | 4.66E-06 | 4.03E-05 |
| MIR548A3 | 7.97229 | 6.788752 | 1.183538 | 2.55E-11 | 2.61E-09 |
| MIR548AA2 | 11.79563 | 10.35417 | 1.441465 | 2.08E-11 | 2.32E-09 |
| MIR548AD | 18.38549 | 16.90617 | 1.479318 | 1.12E-11 | 1.48E-09 |
| MIR548AI | 17.0208 | 15.57439 | 1.446412 | 2.97E-12 | 5.19E-10 |
| MIR548AJ2 | 11.87672 | 10.80325 | 1.073467 | 2.97E-12 | 5.19E-10 |
| MIR548C | 5.301417 | 4.21489 | 1.086527 | 1.16E-09 | 4.86E-08 |
| MIR548D1 | 19.0053 | 17.73782 | 1.267487 | 1.85E-12 | 3.86E-10 |
| MIR548D2 | 16.59384 | 15.04144 | 1.552397 | 5.88E-12 | 8.91E-10 |
| MIR548H2 | 10.95694 | 9.675789 | 1.281153 | 1.91E-08 | 4.46E-07 |
| MIR548H3 | 9.910972 | 8.612339 | 1.298633 | 9.91E-09 | 2.67E-07 |
| MIR548K | 5.571583 | 4.293145 | 1.278439 | 9.38E-08 | 1.61E-06 |
| MIR548O2 | 12.78928 | 11.04785 | 1.741422 | 3.10E-11 | 3.00E-09 |
| MIR548T | 11.01237 | 10.00235 | 1.010017 | 2.30E-10 | 1.32E-08 |
| MIR548W | 12.24211 | 10.6867 | 1.555406 | 5.75E-09 | 1.70E-07 |
| MIR548X | 13.00496 | 11.17224 | 1.832723 | 1.63E-10 | 1.02E-08 |
| MIR570 | 6.327633 | 5.116266 | 1.211367 | 1.33E-07 | 2.12E-06 |
| MIR604 | 7.705538 | 5.602412 | 2.103126 | 4.44E-07 | 5.80E-06 |
| MIR612 | 10.77906 | 9.417222 | 1.361839 | 1.01E-06 | 1.14E-05 |
| MIR614 | 11.7216 | 12.82911 | -1.1075 | 0.00092012 | 0.003323007 |
| MIR644A | 7.476012 | 5.711971 | 1.764041 | 2.73E-10 | 1.52E-08 |
| MIR95 | 5.059444 | 3.75691 | 1.302534 | 7.41E-08 | 1.32E-06 |
| MMP2 | 6.44015 | 5.011137 | 1.429014 | 2.47E-08 | 5.48E-07 |
| MMP7 | 11.23011 | 8.935328 | 2.294778 | 0.0018564 | 0.005952847 |
| MNDA | 7.265071 | 8.458207 | -1.19314 | 0.00018148 | 0.000852832 |
| MORN2 | 7.578231 | 8.905348 | -1.32712 | 1.20E-13 | 5.62E-11 |
| MOXD1 | 6.59866 | 4.506565 | 2.092095 | 7.41E-08 | 1.32E-06 |
| MPP5 | 13.15774 | 14.25765 | -1.09991 | 2.45E-09 | 8.82E-08 |
| MRO | 4.911834 | 6.129527 | -1.21769 | 2.58E-05 | 0.000165515 |
| MS4A4A | 5.846286 | 4.829428 | 1.016858 | 0.00259123 | 0.007827385 |
| MS4A6A | 6.730323 | 5.328279 | 1.402044 | 0.00023899 | 0.00107315 |
| MT1F | 16.6153 | 17.73723 | -1.12193 | 1.68E-08 | 4.01E-07 |
| MT1G | 17.30574 | 18.51736 | -1.21162 | 4.66E-06 | 4.03E-05 |
| MT1H | 16.39894 | 17.93034 | -1.53139 | 2.94E-06 | 2.75E-05 |
| MXD1 | 6.913819 | 7.96046 | -1.04664 | 7.97E-09 | 2.25E-07 |
| NAIP | 7.163031 | 5.999139 | 1.163892 | 2.55E-11 | 2.61E-09 |
| NAP1L2 | 6.06103 | 7.098315 | -1.03728 | 6.64E-11 | 5.31E-09 |
| NAPSA | 5.469048 | 6.53034 | -1.06129 | 2.21E-06 | 2.18E-05 |
| NAT8 | 9.644603 | 11.20655 | -1.56195 | 0.00013695 | 0.000672529 |
| NAT8B | 10.73239 | 12.37245 | -1.64006 | 7.65E-05 | 0.000411215 |
| NEBL-AS1 | 4.530795 | 5.66651 | -1.13571 | 3.11E-14 | 2.31E-11 |
| NELL1 | 8.687891 | 7.219747 | 1.468145 | 0.01114815 | 0.026585383 |
| NFIL3 | 5.318461 | 6.542356 | -1.2239 | 2.39E-09 | 8.82E-08 |
| NID2 | 8.165689 | 7.086091 | 1.079599 | 1.66E-07 | 2.57E-06 |
| NKG7 | 9.242126 | 10.26553 | -1.0234 | 0.00259123 | 0.007827385 |
| NLK | 12.96075 | 14.0188 | -1.05805 | 6.58E-08 | 1.20E-06 |
| NPHS1 | 11.06366 | 12.67809 | -1.61443 | 1.66E-07 | 2.57E-06 |
| NPIPB11 | 13.75137 | 12.35064 | 1.400729 | 9.09E-12 | 1.25E-09 |
| NPIPB3 | 15.02476 | 13.82232 | 1.202443 | 1.39E-11 | 1.70E-09 |
| NPIPB5 | 14.95335 | 13.71442 | 1.238936 | 4.59E-09 | 1.44E-07 |
| NPIPL3 | 13.3082 | 12.07104 | 1.23716 | 2.08E-11 | 2.32E-09 |
| NPL | 6.778747 | 7.933596 | -1.15485 | 4.20E-06 | 3.71E-05 |
| NR4A1 | 4.952818 | 6.007276 | -1.05446 | 2.83E-09 | 9.73E-08 |
| NR4A2 | 4.546799 | 5.783552 | -1.23675 | 4.51E-06 | 3.95E-05 |
| NT5E | 9.584648 | 8.349983 | 1.234665 | 8.69E-06 | 6.72E-05 |
| OCLM | 5.558141 | 4.110079 | 1.448062 | 2.11E-09 | 7.92E-08 |
| OLFML2B | 5.796176 | 4.640324 | 1.155852 | 0.00023899 | 0.00107315 |
| OR51E1 | 5.157142 | 4.111455 | 1.045687 | 0.00056091 | 0.002195856 |
| PAH | 10.47561 | 12.64597 | -2.17036 | 5.47E-07 | 6.91E-06 |
| PAIP2B | 6.60582 | 7.656371 | -1.05055 | 1.48E-07 | 2.34E-06 |
| PAK1 | 10.97199 | 12.13705 | -1.16506 | 2.59E-07 | 3.69E-06 |
| PBLD | 7.93082 | 9.265102 | -1.33428 | 2.01E-06 | 2.02E-05 |
| PCBD1 | 9.762671 | 10.99592 | -1.23325 | 8.70E-13 | 2.09E-10 |
| PCDH18 | 6.463634 | 5.343628 | 1.120007 | 6.58E-08 | 1.20E-06 |
| PCDHGA10 | 9.277684 | 8.135053 | 1.142631 | 9.92E-10 | 4.31E-08 |
| PCK1 | 7.10611 | 9.113207 | -2.0071 | 1.33E-07 | 2.12E-06 |
| PCOLCE2 | 11.71318 | 12.9507 | -1.23752 | 2.30E-10 | 1.32E-08 |
| PDE1A | 6.913534 | 5.910258 | 1.003276 | 2.01E-06 | 2.02E-05 |
| PDK4 | 8.789624 | 12.06422 | -3.27459 | 9.62E-15 | 1.23E-11 |
| PDZK1 | 12.01476 | 13.13736 | -1.1226 | 0.00019452 | 0.00090305 |
| PDZK1IP1 | 10.97641 | 12.26281 | -1.2864 | 4.34E-09 | 1.36E-07 |
| PDZK1P1 | 11.11484 | 12.17854 | -1.0637 | 0.00040698 | 0.001679168 |
| PEPD | 10.33088 | 11.7852 | -1.45432 | 9.91E-09 | 2.67E-07 |
| PER3 | 8.02298 | 6.864633 | 1.158347 | 9.92E-07 | 1.13E-05 |
| PFN1P2 | 8.754348 | 6.994957 | 1.759391 | 1.85E-12 | 3.86E-10 |
| PI3 | 3.734087 | 5.185591 | -1.4515 | 1.83E-06 | 1.87E-05 |
| PIPOX | 6.837972 | 7.983905 | -1.14593 | 2.58E-05 | 0.000165515 |
| PLCG2 | 7.629327 | 8.826952 | -1.19762 | 0.01399485 | 0.032135955 |
| PLG | 6.741699 | 7.991118 | -1.24942 | 0.00036474 | 0.001536305 |
| PLGLA | 7.132201 | 8.36438 | -1.23218 | 8.24E-05 | 0.000436595 |
| PAG1 | 8.184182 | 7.071191 | 1.112991 | 5.75E-09 | 1.70E-07 |
| PLTP | 10.09535 | 11.1934 | -1.09805 | 2.59E-07 | 3.69E-06 |
| PLVAP | 5.674484 | 4.618283 | 1.056201 | 0.00035706 | 0.001505577 |
| POSTN | 12.19022 | 10.96775 | 1.222471 | 0.00018148 | 0.000852832 |
| PPBP | 5.788262 | 7.090866 | -1.3026 | 2.80E-05 | 0.000176873 |
| PRAP1 | 7.598243 | 8.657541 | -1.0593 | 7.10E-05 | 0.000386586 |
| PRINS | 5.216782 | 3.928023 | 1.28876 | 1.85E-12 | 3.86E-10 |
| PRKAR2B | 9.065371 | 10.24356 | -1.17819 | 5.00E-09 | 1.53E-07 |
| PRODH2 | 7.224843 | 9.028014 | -1.80317 | 1.05E-07 | 1.77E-06 |
| PSAT1 | 8.320575 | 9.935612 | -1.61504 | 0.00014702 | 0.000714231 |
| PTGDS | 10.34146 | 11.52257 | -1.18111 | 5.88E-12 | 8.91E-10 |
| PTGS2 | 4.596119 | 7.112468 | -2.51635 | 1.82E-09 | 6.97E-08 |
| PTH1R | 11.52831 | 12.81187 | -1.28356 | 6.64E-11 | 5.31E-09 |
| PTN | 7.769083 | 6.292485 | 1.476599 | 3.23E-06 | 2.96E-05 |
| PVALB | 5.872318 | 7.1555 | -1.28318 | 1.03E-05 | 7.78E-05 |
| RASD1 | 3.35953 | 4.482036 | -1.12251 | 3.99E-07 | 5.31E-06 |
| RASSF9 | 5.709186 | 4.585251 | 1.123935 | 1.01E-06 | 1.14E-05 |
| RBP5 | 11.63767 | 13.43871 | -1.80104 | 8.26E-07 | 9.67E-06 |
| RERG-IT1 | 5.111361 | 3.791852 | 1.319509 | 9.92E-10 | 4.31E-08 |
| RGS2 | 9.44226 | 11.55666 | -2.1144 | 2.94E-06 | 2.75E-05 |
| RGS4 | 5.011164 | 3.947256 | 1.063909 | 3.28E-05 | 0.00020171 |
| RHCG | 4.49993 | 5.573859 | -1.07393 | 3.03E-05 | 0.000188806 |
| RNA5SP160 | 4.541649 | 3.472755 | 1.068894 | 1.50E-06 | 1.59E-05 |
| RNA5SP166 | 6.624536 | 5.593574 | 1.030962 | 7.26E-10 | 3.35E-08 |
| RNA5SP187 | 9.720097 | 8.239355 | 1.480742 | 3.76E-11 | 3.45E-09 |
| RNA5SP195 | 10.48214 | 7.402586 | 3.079551 | 6.69E-13 | 1.83E-10 |
| RNA5SP20 | 6.02273 | 4.408953 | 1.613778 | 1.94E-10 | 1.16E-08 |
| RNA5SP217 | 6.705676 | 5.394664 | 1.311012 | 0.00097732 | 0.003491831 |
| RNA5SP229 | 5.368987 | 3.919934 | 1.449053 | 6.19E-10 | 2.97E-08 |
| RNA5SP260 | 5.42095 | 4.371836 | 1.049114 | 2.17E-08 | 4.94E-07 |
| RNA5SP268 | 6.278392 | 5.184986 | 1.093407 | 7.29E-06 | 5.80E-05 |
| RNA5SP310 | 8.379069 | 5.948107 | 2.430963 | 5.27E-10 | 2.58E-08 |
| RNA5SP311 | 8.889481 | 6.016363 | 2.873118 | 7.98E-11 | 6.07E-09 |
| RNA5SP312 | 8.852272 | 5.79744 | 3.054832 | 2.55E-11 | 2.61E-09 |
| RNA5SP313 | 8.852272 | 5.79744 | 3.054832 | 2.55E-11 | 2.61E-09 |
| RNA5SP314 | 8.889481 | 6.016363 | 2.873118 | 7.98E-11 | 6.07E-09 |
| RNA5SP315 | 8.852272 | 5.79744 | 3.054832 | 2.55E-11 | 2.61E-09 |
| RNA5SP316 | 8.379069 | 5.948107 | 2.430963 | 5.27E-10 | 2.58E-08 |
| RNA5SP317 | 8.889481 | 6.016363 | 2.873118 | 7.98E-11 | 6.07E-09 |
| RNA5SP320 | 5.394333 | 3.712113 | 1.682221 | 1.63E-10 | 1.02E-08 |
| RNA5SP343 | 6.276971 | 5.193624 | 1.083347 | 6.58E-08 | 1.20E-06 |
| RNA5SP450 | 7.614319 | 6.584442 | 1.029877 | 1.23E-06 | 1.34E-05 |
| RNA5SP82 | 6.756507 | 5.385991 | 1.370516 | 3.23E-06 | 2.96E-05 |
| RNF138P1 | 5.953298 | 4.556929 | 1.396369 | 5.84E-08 | 1.10E-06 |
| RNF152 | 8.520465 | 10.32156 | -1.8011 | 9.38E-08 | 1.61E-06 |
| RNU4-2 | 5.72859 | 6.993684 | -1.26509 | 3.54E-06 | 3.20E-05 |
| RNU5B-1 | 5.946631 | 7.355495 | -1.40886 | 8.49E-10 | 3.80E-08 |
| RNU5D-2P | 6.045298 | 7.148669 | -1.10337 | 9.15E-07 | 1.05E-05 |
| RNU5E-2P | 6.018828 | 7.307005 | -1.28818 | 4.44E-07 | 5.80E-06 |
| RNU6-21P | 6.771056 | 5.749387 | 1.021669 | 1.13E-08 | 2.95E-07 |
| RNU6-3 | 10.5919 | 9.404244 | 1.187658 | 2.11E-09 | 7.92E-08 |
| RNU6-38 | 8.783161 | 7.423432 | 1.359729 | 5.51E-11 | 4.58E-09 |
| RNU6-60 | 8.138668 | 6.935419 | 1.20325 | 1.29E-08 | 3.27E-07 |
| RNU6-79P | 6.30682 | 5.238623 | 1.068197 | 0.00116883 | 0.004049135 |
| RNU6-8 | 8.720899 | 7.285915 | 1.434984 | 7.26E-10 | 3.35E-08 |
| RNU6-80 | 6.67833 | 5.651641 | 1.02669 | 2.11E-09 | 7.92E-08 |
| RNU6-82P | 5.552857 | 4.4015 | 1.151357 | 4.93E-07 | 6.31E-06 |
| RNU6-83P | 5.498211 | 4.333675 | 1.164536 | 2.30E-10 | 1.32E-08 |
| RNU7-10P | 6.45562 | 5.05795 | 1.39767 | 3.59E-08 | 7.44E-07 |
| RNU7-13P | 9.796696 | 8.708453 | 1.088243 | 4.48E-10 | 2.25E-08 |
| RNU7-24P | 9.150898 | 8.022585 | 1.128314 | 1.16E-09 | 4.86E-08 |
| RNU7-29P | 8.564694 | 7.050582 | 1.514113 | 1.85E-12 | 3.86E-10 |
| RNU7-35P | 8.377774 | 7.205334 | 1.17244 | 9.57E-11 | 6.87E-09 |
| RNU7-40P | 7.419 | 5.764251 | 1.654749 | 6.60E-09 | 1.92E-07 |
| RNU7-45P | 7.155602 | 5.638509 | 1.517093 | 7.98E-11 | 6.07E-09 |
| RNU7-47P | 9.397236 | 8.292264 | 1.104972 | 2.55E-11 | 2.61E-09 |
| RNU7-48P | 9.174499 | 7.707984 | 1.466515 | 1.70E-11 | 1.97E-09 |
| RNU7-53P | 9.512027 | 8.145086 | 1.366942 | 1.15E-10 | 7.72E-09 |
| RNU7-61P | 6.941517 | 5.671555 | 1.269962 | 2.83E-09 | 9.73E-08 |
| RNU7-62P | 7.346252 | 6.242066 | 1.104187 | 2.83E-09 | 9.73E-08 |
| RNU7-75P | 5.578114 | 4.507557 | 1.070557 | 1.13E-05 | 8.34E-05 |
| RNU7-7P | 9.919365 | 8.615323 | 1.304042 | 3.76E-11 | 3.45E-09 |
| RNY3P6 | 7.805381 | 6.67674 | 1.128641 | 6.67E-06 | 5.40E-05 |
| RPL23AP32 | 8.154674 | 6.863789 | 1.290886 | 1.35E-09 | 5.48E-08 |
| RPL36AP33 | 6.554692 | 5.218003 | 1.336689 | 1.82E-09 | 6.97E-08 |
| RRAS | 9.48589 | 10.59662 | -1.11073 | 1.68E-08 | 4.01E-07 |
| S100A12 | 4.67707 | 7.587597 | -2.91053 | 3.77E-09 | 1.23E-07 |
| S100A8 | 5.394414 | 7.202869 | -1.80845 | 4.48E-10 | 2.25E-08 |
| S100A9 | 8.753094 | 12.04584 | -3.29274 | 7.26E-10 | 3.35E-08 |
| SAT2 | 9.520709 | 11.20599 | -1.68528 | 4.70E-12 | 7.52E-10 |
| SBSPON | 12.10085 | 13.23288 | -1.13203 | 5.58E-06 | 4.67E-05 |
| SCARNA7 | 11.01864 | 9.359771 | 1.658867 | 1.83E-06 | 1.87E-05 |
| SELL | 4.860916 | 5.949512 | -1.0886 | 3.23E-06 | 2.96E-05 |
| SERPINF1 | 5.900138 | 4.647896 | 1.252242 | 3.28E-05 | 0.00020171 |
| SFRP2 | 8.779188 | 5.170509 | 3.608679 | 6.07E-07 | 7.50E-06 |
| SFRP4 | 5.016076 | 4.015124 | 1.000953 | 0.00052643 | 0.002081037 |
| SLC12A2 | 7.80409 | 6.785613 | 1.018478 | 3.28E-05 | 0.00020171 |
| SLC12A3 | 6.291584 | 8.594919 | -2.30333 | 3.88E-06 | 3.45E-05 |
| SLC13A1 | 8.743226 | 9.879763 | -1.13654 | 0.00924792 | 0.02279511 |
| SLC13A3 | 9.349669 | 11.03756 | -1.6879 | 8.24E-05 | 0.000436595 |
| SLC17A1 | 8.74998 | 9.947145 | -1.19716 | 0.0020773 | 0.006509794 |
| SLC17A3 | 8.113053 | 9.134535 | -1.02148 | 0.01598891 | 0.035825295 |
| SLC22A6 | 9.041853 | 10.34806 | -1.3062 | 0.0046397 | 0.012799617 |
| SLC22A8 | 9.118608 | 10.56036 | -1.44175 | 0.00092012 | 0.003323007 |
| SLC27A2 | 7.829202 | 8.913987 | -1.08479 | 0.00727549 | 0.01869577 |
| SLC2A2 | 6.329718 | 7.456 | -1.12628 | 0.00035706 | 0.001505577 |
| SLC34A1 | 8.803501 | 9.965641 | -1.16214 | 0.01463417 | 0.033344657 |
| SLC36A2 | 7.45665 | 9.208755 | -1.75211 | 2.20E-05 | 0.000144659 |
| SLC47A1 | 7.859242 | 8.981759 | -1.12252 | 0.00092012 | 0.003323007 |
| SLC4A4 | 9.221327 | 10.62583 | -1.40451 | 0.00219637 | 0.00682811 |
| SLC5A12 | 10.69253 | 12.33035 | -1.63782 | 0.00175409 | 0.005672939 |
| SLC6A13 | 6.477475 | 7.572924 | -1.09545 | 2.02E-05 | 0.000135182 |
| SLC6A19 | 7.417439 | 9.147478 | -1.73004 | 2.02E-05 | 0.000135182 |
| SLC6A6 | 9.398282 | 8.136162 | 1.262121 | 1.15E-10 | 7.72E-09 |
| SLC7A5P2 | 10.83959 | 9.335009 | 1.504581 | 3.89E-13 | 1.18E-10 |
| SLC7A7 | 7.952951 | 9.512726 | -1.55977 | 4.59E-08 | 8.99E-07 |
| SLC7A8 | 12.09087 | 13.52269 | -1.43181 | 2.11E-09 | 7.92E-08 |
| SLC7A9 | 7.214666 | 8.556068 | -1.3414 | 0.00086596 | 0.003153142 |
| SLIT3 | 8.740815 | 7.403321 | 1.337495 | 2.83E-09 | 9.73E-08 |
| SMA4 | 8.455785 | 7.221768 | 1.234018 | 4.34E-09 | 1.36E-07 |
| SMA5 | 9.589468 | 8.197704 | 1.391764 | 5.27E-10 | 2.58E-08 |
| SMCO3 | 8.008979 | 9.795152 | -1.78617 | 1.15E-10 | 7.72E-09 |
| SMG1P1 | 11.18928 | 9.641713 | 1.547565 | 6.69E-13 | 1.83E-10 |
| SMIM2-AS1 | 6.606705 | 7.818931 | -1.21223 | 8.34E-08 | 1.46E-06 |
| SNHG8 | 12.94369 | 14.01693 | -1.07324 | 6.51E-06 | 5.32E-05 |
| SNORA12 | 5.226553 | 6.583864 | -1.35731 | 2.21E-06 | 2.18E-05 |
| SNORA22 | 8.204338 | 10.03534 | -1.831 | 1.13E-08 | 2.95E-07 |
| SNORA45 | 6.491616 | 5.422941 | 1.068675 | 1.23E-06 | 1.34E-05 |
| SNORA47 | 5.31595 | 6.34818 | -1.03223 | 3.99E-07 | 5.31E-06 |
| SNORA48 | 11.97061 | 13.84945 | -1.87883 | 1.66E-07 | 2.57E-06 |
| SNORA70G | 8.133006 | 6.52383 | 1.609176 | 1.91E-08 | 4.46E-07 |
| SNORD105B | 3.631018 | 4.653455 | -1.02244 | 1.35E-09 | 5.48E-08 |
| SNORD13 | 12.74239 | 13.87475 | -1.13236 | 0.00357662 | 0.010310315 |
| SNORD14D | 4.406792 | 5.617863 | -1.21107 | 0.00018148 | 0.000852832 |
| SNORD14E | 5.680263 | 7.044205 | -1.36394 | 0.00103774 | 0.003663351 |
| SNORD3A | 8.909147 | 11.95105 | -3.0419 | 6.41E-16 | 1.48E-12 |
| SNORD3B-1 | 8.314316 | 11.42131 | -3.10699 | 6.41E-16 | 1.48E-12 |
| SNORD3B-2 | 8.314316 | 11.42131 | -3.10699 | 6.41E-16 | 1.48E-12 |
| SNORD3C | 7.959047 | 10.93034 | -2.9713 | 6.41E-16 | 1.48E-12 |
| SNORD3D | 7.714239 | 10.7503 | -3.03606 | 6.41E-16 | 1.48E-12 |
| SNORD41 | 4.009978 | 5.386458 | -1.37648 | 0.00097732 | 0.003491831 |
| SNORD46 | 8.504616 | 9.52988 | -1.02526 | 0.00196406 | 0.006214981 |
| SNORD59B | 4.066256 | 5.26969 | -1.20343 | 0.00131472 | 0.004462578 |
| SNORD63 | 7.219978 | 5.999421 | 1.220557 | 8.69E-06 | 6.72E-05 |
| SNORD66 | 3.04846 | 4.942694 | -1.89423 | 9.09E-12 | 1.25E-09 |
| SNORD7 | 5.896951 | 4.519018 | 1.377933 | 3.59E-08 | 7.44E-07 |
| SNORD75 | 4.580445 | 3.499013 | 1.081432 | 4.44E-07 | 5.80E-06 |
| SNORD78 | 6.119567 | 5.039994 | 1.079573 | 0.00016924 | 0.000804703 |
| SNORD99 | 7.611702 | 6.550794 | 1.060908 | 0.00219637 | 0.00682811 |
| SORD | 6.475694 | 7.794921 | -1.31923 | 1.12E-06 | 1.24E-05 |
| SOST | 11.51514 | 12.70269 | -1.18755 | 6.11E-05 | 0.000340329 |
| SPDYE1 | 7.377673 | 6.212502 | 1.165172 | 3.10E-11 | 3.00E-09 |
| SPDYE2 | 8.061478 | 6.642871 | 1.418607 | 1.12E-11 | 1.48E-09 |
| SPDYE3 | 6.976544 | 5.802715 | 1.17383 | 8.70E-13 | 2.09E-10 |
| SPDYE5 | 7.47871 | 6.420332 | 1.058378 | 1.37E-10 | 8.95E-09 |
| SPDYE6 | 8.662965 | 7.119597 | 1.543368 | 3.10E-11 | 3.00E-09 |
| SPDYE8P | 6.981655 | 5.869983 | 1.111673 | 9.09E-12 | 1.25E-09 |
| SST | 6.656864 | 7.807084 | -1.15022 | 0.00881967 | 0.021922561 |
| STRADB | 8.124017 | 9.151743 | -1.02773 | 3.77E-09 | 1.23E-07 |
| SVEP1 | 4.874202 | 3.717059 | 1.157143 | 1.18E-07 | 1.93E-06 |
| SYT11 | 8.507588 | 7.451879 | 1.055709 | 6.11E-06 | 5.02E-05 |
| SYTL2 | 7.989231 | 6.78232 | 1.206911 | 2.47E-05 | 0.000160049 |
| TAS2R30 | 5.389876 | 4.256625 | 1.133251 | 1.91E-08 | 4.46E-07 |
| TAS2R31 | 5.311832 | 4.262915 | 1.048917 | 2.73E-10 | 1.52E-08 |
| TCF21 | 14.13634 | 15.37793 | -1.24158 | 1.64E-05 | 0.000113626 |
| TGFB2 | 7.531184 | 6.459076 | 1.072108 | 1.36E-06 | 1.46E-05 |
| TGFBI | 6.184686 | 5.0024 | 1.182286 | 7.84E-07 | 9.27E-06 |
| TGFBR3 | 12.91749 | 14.00616 | -1.08867 | 1.83E-07 | 2.80E-06 |
| THBS2 | 5.453964 | 4.388902 | 1.065062 | 5.58E-06 | 4.67E-05 |
| THRB-IT1 | 5.97339 | 4.664078 | 1.309312 | 8.66E-09 | 2.41E-07 |
| TM4SF5 | 7.76581 | 9.367063 | -1.60125 | 4.66E-06 | 4.03E-05 |
| TMEM150C | 11.92885 | 13.81338 | -1.88453 | 2.55E-11 | 2.61E-09 |
| TMEM174 | 8.721859 | 10.657 | -1.93514 | 0.0004632 | 0.001872224 |
| TMEM178A | 8.239364 | 9.277323 | -1.03796 | 0.00040698 | 0.001679168 |
| TMEM207 | 4.620074 | 5.633483 | -1.01341 | 0.00038127 | 0.001590491 |
| TMEM27 | 9.829072 | 11.11433 | -1.28526 | 0.00259123 | 0.007827385 |
| TMEM52B | 8.6312 | 10.08297 | -1.45177 | 0.0020773 | 0.006509794 |
| TNC | 7.972606 | 6.572889 | 1.399717 | 8.39E-05 | 0.000443622 |
| TNNI1 | 10.01626 | 11.64556 | -1.6293 | 1.18E-07 | 1.93E-06 |
| TPM1 | 11.10877 | 10.09496 | 1.013818 | 3.52E-07 | 4.83E-06 |
| TPPP3 | 8.60697 | 10.14664 | -1.53967 | 1.37E-10 | 8.95E-09 |
| TREM1 | 3.837047 | 4.963383 | -1.12634 | 1.66E-07 | 2.57E-06 |
| TSPYL5 | 9.464251 | 10.49666 | -1.03241 | 1.13E-08 | 2.95E-07 |
| TST | 7.844752 | 8.88499 | -1.04024 | 1.70E-11 | 1.97E-09 |
| TYRO3 | 10.75192 | 11.97482 | -1.2229 | 1.66E-06 | 1.72E-05 |
| UGT2B7 | 12.17987 | 13.82532 | -1.64545 | 0.00011041 | 0.000561262 |
| UMOD | 9.330585 | 11.18561 | -1.85502 | 0.00357662 | 0.010310315 |
| UQCR10 | 10.82782 | 11.83531 | -1.00748 | 5.12E-13 | 1.49E-10 |
| USH1C | 5.546272 | 6.554063 | -1.00779 | 3.99E-07 | 5.31E-06 |
| USP2 | 6.375542 | 7.54231 | -1.16677 | 4.85E-05 | 0.000281163 |
| USP46 | 8.675431 | 9.688701 | -1.01327 | 1.86E-07 | 2.80E-06 |
| VASN | 10.56538 | 11.89434 | -1.32896 | 2.55E-11 | 2.61E-09 |
| VCAN | 8.504943 | 6.409303 | 2.09564 | 0.00123984 | 0.004256069 |
| VCAN-AS1 | 7.531034 | 5.983818 | 1.547216 | 0.00259123 | 0.007827385 |
| VEGFC | 7.812014 | 6.548276 | 1.263739 | 5.84E-08 | 1.10E-06 |
| VNN2 | 5.892112 | 7.303211 | -1.4111 | 9.57E-11 | 6.87E-09 |
| VSIG4 | 5.837947 | 4.57359 | 1.264357 | 0.0004632 | 0.001872224 |
| VTRNA1-3 | 7.053908 | 4.375342 | 2.678566 | 3.89E-13 | 1.18E-10 |
| VTRNA2-1 | 7.132231 | 5.419242 | 1.712989 | 0.00019452 | 0.00090305 |
| WDR49 | 6.643004 | 7.988305 | -1.3453 | 0.00013695 | 0.000672529 |
| WDR72 | 8.23644 | 9.700663 | -1.46422 | 2.94E-06 | 2.75E-05 |
| XPNPEP2 | 8.245675 | 9.886516 | -1.64084 | 0.00018148 | 0.000852832 |
| ZBED5-AS1 | 8.575922 | 9.586917 | -1.011 | 1.70E-11 | 1.97E-09 |
| ZBTB20-AS2 | 7.012106 | 5.519006 | 1.4931 | 2.89E-07 | 4.06E-06 |
| ZFP36 | 9.165276 | 12.4587 | -3.29342 | 1.20E-13 | 5.62E-11 |
| ZNF638-IT1 | 7.103637 | 5.940292 | 1.163346 | 4.93E-07 | 6.31E-06 |
| ZNRF3 | 9.231843 | 10.2402 | -1.00836 | 1.16E-09 | 4.86E-08 |
