# Supplementary material for: Investigating EGF and PAG1 as necroptosis-related biomarkers for diabetic nephropathy: an in silico and in vitro validation study
Source: Aging (Albany NY). 2023 Nov 20;15(22):13176–93. doi: 10.18632/aging.205233 (PMC10713428; doi:10.18632/aging.205233)
Supplement: Supplementary Tables 5 and 6 [file aging-15-205233-s005.pdf]

Supplementary Table 5. Available clinical characteristics of DN patients and the healthy control in this study.

	GSE96804		GSE142025		GSE131882		
**Baseline characteristics**	**Control (n=20)**	**DN (n=41)**	**Control (n=9)**	**DN (n=28)**	**Baseline characteristics**	**Control (n=3)**	**DN (n=3)**
Male/female (% male)	14/6(70)	29/12(70.7)	7/2 (77.7)	18/10 (64.2)	Male sex, *n*	2	2
Age (year)	43.2±6.52	46.9±7.70	60.89±2.85	53.18 ±2.47	Age in years, mean (range)	59 (54–62)	61(52–74)
Body mass index (kg/m2)	21.6±2.52	24.8±1.82	23.38±1.39	25.57 ± 0.70	Glomerulosclerosis	none	mild
Glycated hemoglobin A1c (%)	5.21±0.58	6.78±1.82	5.68±0.29	8.455 ± 0.66	BMI in kg/m^2, mean (range)	27 (26–29)	35 (22–43)
Systolic blood pressure (mm/Hg)	123.2±18.2	134.2±15.8	128.0±1.80	141.6 ±4.38	Terminal serum creatinine, mean, mg/dL	0.89	1.3
Diastolic blood pressure (mm/Hg)	75.3±10.2	84.8±8.2	75.67±2.26	84.41 ± 2.49	Proteinuria	NA	+
Total urinary protein (g/24hr)	−	2.53±1.20	0.09±0.01	6.11 ± 0.97	Hemoglobin A1c, mean, %	5.2	8.5
eGFR (ml/min)	100.23±10.5	63.16±22.12	117.7 ±8.62	63.79 ± 5.76	eGFR, mean mL/minute	>60	>60

Supplementary Table 6. Gene primer sequences.

Gene	Forward primer (5'-3')	Reverse primer (5'-3')	bp
EGF	TGTCCACGCAATGTGTCTGAA	CATTATCGGGTGAGGAACAACC	133
PAG1	TTCCTGTGCTCTAGTTGTGACA	CACGTTCATCAGGTTCTCATGG	75
ZFP36	GACTGAGCTATGTCGGACCTT	GAGTTCCGTCTTGTATTTGGGG	124
RIP1	TTACATGGAAAAGGCGTGATACA	AGGTCTGCGATCTTAATGTGGA	86
RIP3	CATAGGAAGTGGGGCTACGAT	AATTCGTTATCCAGACTTGCCAT	95
MLKL	AGGAGGCTAATGGGGAGATAGA	TGGCTTGCTGTTAGAAACCTG	70
ACTB	CATGTACGTTGCTATCCAGGC	CTCCTTAATGTCACGCACGAT	188
